# Scope and limitations of a DMF bio-alternative within Sonogashira cross-coupling and Cacchi-type annulation

**DOI:** 10.3762/bjoc.12.187

**Published:** 2016-09-08

**Authors:** Kirsty L Wilson, Alan R Kennedy, Jane Murray, Ben Greatrex, Craig Jamieson, Allan J B Watson

**Affiliations:** 1 Department of Pure and Applied Chemistry, WestCHEM, University of Strathclyde, Thomas Graham Building, 295 Cathedral Street, Glasgow, G1 1XL, UK; 2 Sigma-Aldrich, The Old Brickyard, New Road, Gillingham, Dorset, SP8 4XT, UK; 3 School of Science and Technology, University of New England, Armidale, Australia, 2351

**Keywords:** Cacchi annulation, cross-coupling, heterocycles, Sonogashira, sustainable solvent

## Abstract

Pd-catalysed C–C bond formation is an essential tool within the pharmaceutical and agrochemical industries. Many of these reactions rely heavily on polar aprotic solvents; however, despite their utility, these solvents are incompatible with the drive towards more sustainable chemical synthesis. Herein, we describe the scope and limitations of an alternative to DMF derived from renewable sources (Cyrene^TM^) in Sonogashira cross-coupling and Cacchi-type annulations.

## Introduction

**Scheme 1:**
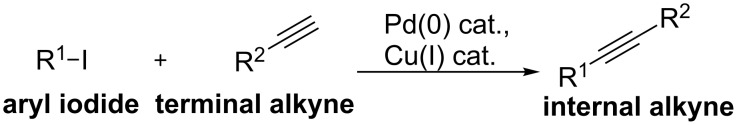
The Sonogashira reaction.

The Sonogashira reaction [1–2] (Scheme 1) is a robust and broadly applicable Pd-catalysed bond-forming process that, alongside the Suzuki–Miyaura reaction [3], has steadily become an indispensible tool for C–C bond formation in the pharmaceutical industry [4]. While the Sonogashira reaction can be effectively carried out in a variety of media [1–2], in the general sense this process clearly relies upon the use of dipolar aprotic solvents, in particular DMF. Indeed, some 41% of all Sonogashira reactions reported using aryl iodides can be linked to the use of DMF as a solvent [5].

In this context, the sustainability movement within pharmaceutical research and development strives to substitute solvents that have regulatory and environmental issues for those with a lower perceived risk. Indeed, solvent replacement has been designated a key research area with numerous pharmaceutical companies detailing their efforts towards a more sustainable solvent selection as part of their overall sustainability programmes [6–23].

Based on its associated regulatory issues [24], it is perhaps no surprise that DMF continues to be a priority solvent for replacement. With legislation surrounding the use of DMF becoming increasingly stringent [24], numerous efforts have been made towards the use of alternative media in the Sonogashira reaction [25–30]. However, notwithstanding its issues, DMF is an excellent solvent for the Sonogashira reaction and its replacement frequently occurs at the expense of increased temperature (and therefore potentially substrate compatibility), reaction time, catalyst loading or the requirement for non-commercial/expensive catalysts, and yield [25–30]. Consequently, poor choice of solvent replacement can result in one of industry’s workhorse reactions becoming rather less predictable and robust.

**Figure 1:**
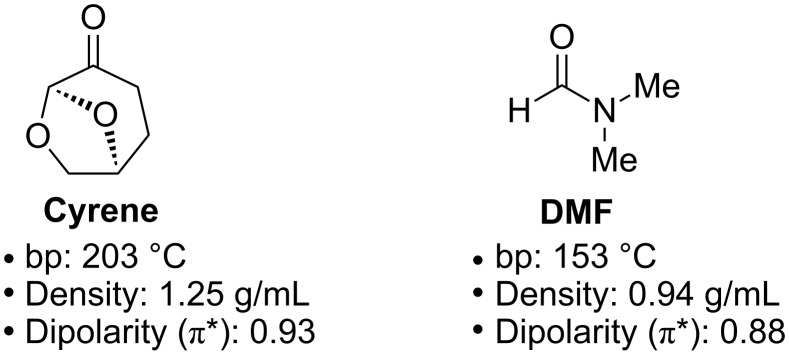
Cyrene vs. DMF – selected physical properties [31–32].

In this regard, dihydrolevoglucosenone (Cyrene, Figure 1), accessed in two steps from cellulose [31–32], has been shown to possess similar physical properties to those of DMF and other dipolar aprotic solvents [31–32]. In addition to its renewability, Cyrene, as yet, has no associated pernicious effects and could potentially represent a direct and functional replacement in many of the fundamental reactions that typically employ DMF [31–32]. The replacement of solvents with regulatory issues with bio-derived alternatives has provided a series of advances within the cross-coupling arena [33], allowing efficient C–C bond formation via cornerstone Pd-based methods including Suzuki–Miyaura [34–35], Mizoroki–Heck [36–37], Sonogashira [38], Stille [39], Hiyama reactions [40], and hydroformylation reactions [41].

In the current study, we present the use of Cyrene as an alternative solvent (direct DMF replacement) for the Sonogashira reaction, as well as related Cacchi-type annulations [42–43], with an emphasis on scope and limitations of its application.

## Results and Discussion

To explore the use of Cyrene in the context of the Sonogashira cross-coupling, we established a simple benchmark reaction using iodobenzene (**1a**) and phenylacetylene (**2a**) (Table 1). A typical literature-derived catalyst system was employed (Pd(PPh_3_)_2_Cl_2_ with CuI additive [44–45]) and conversion to diphenylacetylene (**3a**) was monitored.

**Table 1:** Reaction optimisation and comparison with existing solvents.^a^

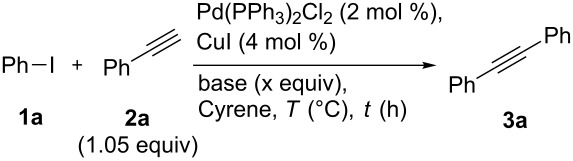		

Entry	Reaction conditions	**3a** (%)^b^

1	0.1 M, Et_3_N (3 equiv), 20 °C, 5 h	94
2	0.3 M, Et_3_N (3 equiv), 20 °C, 5 h	98
3	0.5 M, Et_3_N (3 equiv), 20 °C, 5 h	100
4	0.5 M, K_3_PO_4_ (3 equiv), 20 °C, 5 h	–^c^
5	0.5 M, Cs_2_CO_3_ (3 equiv), 20 °C, 5 h	–^c^
7	0.5 M, Et_3_N (1.1 equiv), 20 °C, 5 h	98
8	0.5 M, Et_3_N (1.1 equiv), 30 °C, 1 h	96
9^d^	0.5 M, Et_3_N (1.1 equiv), 30 °C, 1 h	81
10^e^	0.5 M, Et_3_N (1.1 equiv), 30 °C, 1 h	87

^a^**1** (1 equiv, 0.25 mmol), **2** (1.05 equiv, 0.26 mmol), Pd(PPh_3_)_2_Cl_2_ (2 mol %), CuI (4 mol %), base (see table), Cyrene, temperature (see table), time (see table), N_2_. ^b^Isolated yield. ^c^Reaction mixture solidified, product was not isolated. ^d^THF used as solvent. ^e^DMF used as solvent.

Pleasingly, high conversion to product was immediately observed at room temperature in 5 h (94%, Table 1, entry 1). This high conversion was consistent across several reaction concentrations (Table 1, entries 2 and 3) allowing for a reduction in solvent volume, commensurate with the principles of green chemistry [46–47].

**Figure 2:**
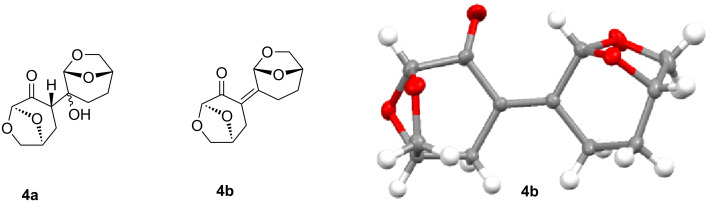
Aldol products **4a** and **4b** and single crystal X-ray structure of **4b**.

In attempts to further limit waste, we scanned a series of bases (see Supporting Information File 1); organic bases consistently performed more effectively and alternatives to Et_3_N provided no significant advantages. However, during this process we identified some potential limitations of this emerging solvent. Specifically, inorganic bases such as K_3_PO_4_ and Cs_2_CO_3_ (Table 1, entries 4 and 5) resulted in the generation of a solid reaction mixture. Further analysis revealed that the aldol products **4a** and **4b** (Figure 2) were generated under specific reaction conditions.

The manufacturers note that when using Cyrene, materials to avoid are strong acids, and strong oxidising and reducing agents. Since sensitivity to base was not specified, we surveyed a range of bases at various temperatures to evaluate the limitations of Cyrene under such conditions (Table 2).

**Table 2:** Evaluation of the base sensitivity of Cyrene.^a^

Entry	Base	Temp. (°C)	Reaction (Y/N)^b^

1	KOAc	25	N
		50	Y
		100	Y
2	Pyridine	25	N
		50	Y
		100	Y
3	K_2_CO_3_	25	Y
		50	Y
		100	Y
4	DIPEA	25	N
		50	N
		100	Y
5	Cs_2_CO_3_	25	Y
		50	Y
		100	Y
6	Et_3_N	25	N
		50	N
		100	Y
7	K_3_PO_4_	25	Y
		50	Y
		100	Y
8	DBU	25	Y
		50	Y
		100	Y
9	KOH	25	Y
		50	Y
		100	Y
10	*t*-BuOK	25	Y
		50	Y
		100	Y
11	NaH	25	Y
		50	Y
		100	Y

^a^Reaction conditions: base (0.07 mmol) and Cyrene (0.5 mL) stirred at the indicated temeperature for 24 h before analysis by TLC and ^1^H NMR . ^b^Y = reaction occurs, N = no reaction. See Supporting Information File 1.

Under these specific reaction conditions, with the exception of Et_3_N and DIPEA, there was a clear base sensitivity displayed by Cyrene in the presence of all bases when the temperature was elevated above 25 °C. Organic bases such as pyridine (Table 2, entry 2), DIPEA (Table 2, entry 4), and Et_3_N (Table 2, entry 6) were tolerated at 25 °C with DIPEA and Et_3_N also tolerated at 50 °C. DBU, however, was not tolerated at any temperature (Table 2, entry 8). With the exception of KOAc (Table 2, entry 1), all inorganic bases resulted in reaction with the solvent at room temperature (Table 2, entries 3, 5, 7, and 9–11). The extent of the reaction varied from the generation of additional components, such as **4a** and **4b**, to gelation or complete solidification of the reaction mixture. However, in a moderately basic reaction mixture (e.g., using Et_3_N) at mild reaction temperatures this issue could be entirely avoided. As such, optimisation of the Sonogashira process allowed complete conversion and 96% isolated yield in 1 h at 30 °C (Table 1, entry 8). Importantly, the Cyrene-based system compared very favourably upon comparison with standard solvents (THF and DMF; Table 1, entries 9 and 10, respectively).

**Scheme 2:**
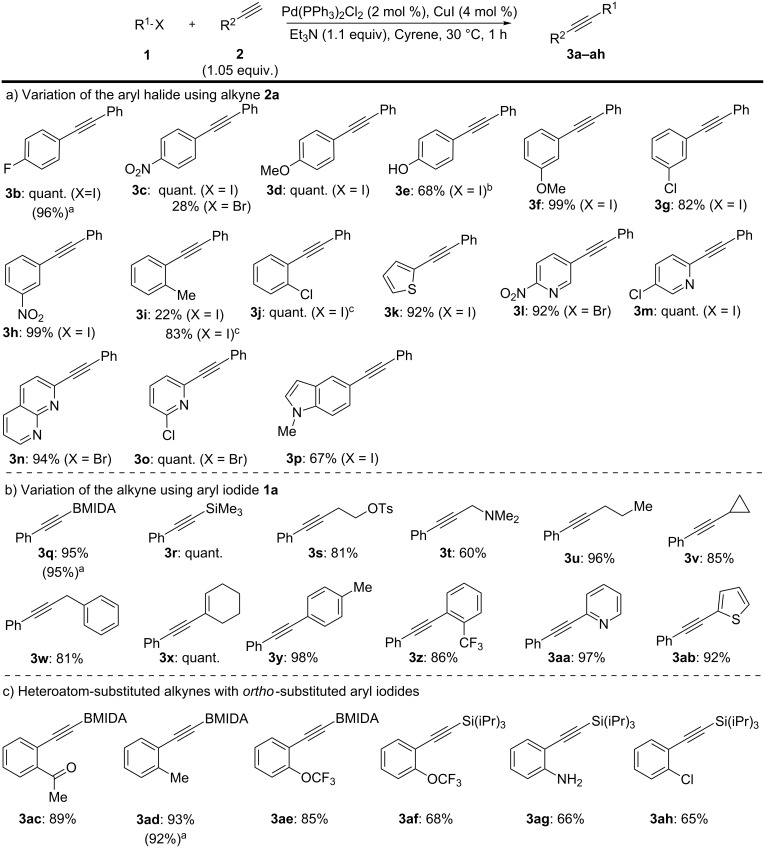
Cyrene-based Sonogashira cross-coupling: Substrate scope. Isolated yields. ^a^Yield using DMF as solvent. ^b^2 equiv of Et_3_N used. ^c^24 h reaction time.

Continuing with the primary investigation and with an optimised set of reaction conditions, we sought to explore the generality of Cyrene in the Sonogashira cross-coupling (Scheme 2). Significantly, a broad range of functionalised aryl and heteroaryl iodides were tolerated (Scheme 2a).

In addition, electron-deficient aryl bromides were accommodated, although with some variation in yield (**3c**, **3l**, **3o**, **3n**). Functionality on the alkyne component was also typically well tolerated (Scheme 2b). While **3i** and **3j** required an extended reaction time, this was a substrate-specific problem for the use of **2a** with these *ortho*-substituted aryl iodides that was not apparent for other alkyne/*ortho*-substituted iodoarene combinations (Scheme 2c).

**Scheme 3:**
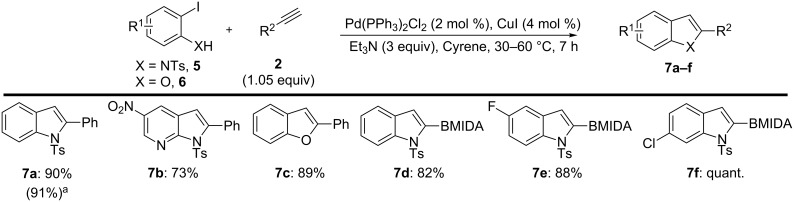
Cacchi-type annulation of *o*-amino/hydroxy iodoarenes. Isolated yields. ^a^Yield using DMF as solvent.

Judicious selection of reacting components also enabled the development of a useful Cacchi-type annulation (Scheme 3) [42–43]. Specifically, employing *ortho*-amino (**5**) or *ortho*-hydroxyaryl iodides (**6**) in the Sonogashira process generated an alkyne intermediate that, upon increasing the reaction temperature from 30 °C to 60 °C, could undergo 5-*endo*-dig cyclisation to forge functionalised and pharmaceutically relevant indole, benzofuran, and aza-indole scaffolds in a single operation (**7a**–**f**) [48–52].

Finally, with the viewpoint of generality of DMF substitution by Cyrene, the base/temperature sensitivity issue may have potential implications for further applications of Cyrene within well-used organic transformations. For example, the majority of many other standard cross-coupling processes employ inorganic or organic bases and heat (e.g., Suzuki–Miyaura, Heck). Accordingly, Cyrene may be projected to be incompatible with standard conditions for these reactions and its use would necessitate base-free or exceptionally mildly basic reaction conditions. In contrast, amide-bond formation is the most practiced reaction in the pharmaceutical industry [4] and these are routinely performed in DMF at room temperature in the presence of organic bases [53]. As such, Cyrene may offer considerable potential in this area. However, additional work will be required to validate the practicality of Cyrene as a viable DMF replacement in these applications.

## Conclusion

In summary, we have developed a mild and robust method for the Sonogashira reaction, employing the bio-derived and sustainable alternative to DMF, Cyrene. In addition, we have shown the capacity for extension of the utility of this new solvent towards enabling the cascade synthesis of functionalised indoles and benzofurans via a Cacchi-type annulation. Perhaps more importantly, we have documented some of the limitations of the use of Cyrene as a solvent, providing guidance emerging in relation to the thermal and chemical (base) stabilities of this promising green solvent.

## Supporting Information

File 1 Experimental procedures, analytical data, copies of NMR spectra, and single X-ray crystal diffraction data of **4b**.
