# Supplementary material for: Scope and limitations of a DMF bio-alternative within Sonogashira cross-coupling and Cacchi-type annulation
Source: Beilstein J Org Chem. 2016 Sep 8;12:2005–11. doi: 10.3762/bjoc.12.187 (PMC5082449; doi:10.3762/bjoc.12.187)

Supporting Information

for

Scope and limitations of a DMF bio-alternative within Sonogashira cross-coupling and Cacchi-type annulation

Kirsty L. Wilson,[1] Alan R. Kennedy,[1] Jane Murray,[2] Ben Greatrex,[3] Craig Jamieson[1] and Allan J. B. Watson*[1]

Address: [1]Department of Pure and Applied Chemistry, WestCHEM, University of Strathclyde, Thomas Graham Building, 295 Cathedral Street, Glasgow, G1 1XL, UK; [2]Sigma-Aldrich, The Old Brickyard, New Road, Gillingham, Dorset, SP8 4XT, UK and [3]School of Science and Technology, University of New England, Armidale, Australia, 2351

Email: Allan J. B. Watson* - allan.watson.100@strath.ac.uk

*Corresponding author

Experimental procedures, analytical data, copies of NMR spectra, and single X-ray crystal diffraction data of 4b

Contents

1. General
    1.1 Purification of solvents
    1.2 Purification and drying of bases
    1.3Experimental details
    1.4 Purification of products
    1.5 Analysis of products

2. General experimental procedures

3. Reaction optimization data
    3.1 Variation of concentration
    3.2 Variation of the base
    3.3 Variation of time and temperature
    3.4 Variation of solvent

4. Base sensitivity study

5. Compound characterization data
    5.1 Preparation of intermediates
    5.2 Product from Table 1
    5.3 Products from Scheme 2a
    5.4 Products from Scheme 2b
    5.5 Products from Scheme 2c
    5.6 Cyrene homo-aldol adducts, **4a** and **4b**
    5.7 Products from Scheme 3

6. Crystallographic data for compound **4a**

7. References

8. NMR spectra for intermediates and products

9. NMR evidence for the evaluation of the base sensitivity

1. General

All reagents and solvents were obtained from commercial suppliers and were used without further purification unless otherwise stated. Purification was carried out according to standard laboratory methods.[1]

1.1 Purification of solvents

Cyrene was supplied directly by Circa and used as obtained. DMF was dried by heating to reflux over previously activated 4 Å molecular sieves and distilling under vacuum before being purged with, and stored under N_2 in a septum-sealed oven-dried flask over previously activated 4 Å molecular sieves. THF was obtained from a PureSolv SPS-400-5 solvent purification system and transferred to and stored in a septum-sealed oven-dried flask over previously activated 4 Å molecular sieves and purged with and stored under N_2. CH_2Cl_2, Et_2O, EtOAc, MeCN, and petroleum ether 40–60 °C for purification purposes were used as obtained from suppliers without further purification.

1.2 Purification and drying of bases

Et_3N was dried by heating to reflux over previously activated 4 Å molecular sieves and distilling under vacuum before being purged with, and stored under N_2 in a septum-sealed oven-dried flask over previously activated 4 Å molecular sieves. Inorganic bases were dried in a Heraeus Vacutherm oven at 60 °C under vacuum for a minimum of 24 h before use.

1.3 Experimental details

Reactions were carried out using conventional glassware (preparation of **S1** and **S2**) or in sealed 5 mL microwave vials (optimization reactions and reactions for Schemes 2 and 3). The glassware was oven-dried (150 °C) and purged with N_2 before use. Purging refers to a vacuum/nitrogen-refilling procedure. Room temperature was generally ca. 18 °C. Reactions were carried out at elevated temperatures using a temperature-regulated hotplate/stirrer.

1.4 Purification of products

Thin layer chromatography was carried out using Merck silica plates coated with fluorescent indicator UV254. These were analyzed under 254 nm UV light or developed using a vanillin solution. Normal phase flash chromatography was carried out using ZEOprep 60 HYD 40-63 µm silica gel. Reverse phase flash chromatography was carried out using IST Isolute C18 cartridges. Strong cation-exchange purification was carried out using an SCX cartridge.

1.5 Analysis of products

Fourier transformed infrared (FTIR) spectra were obtained on a Shimadzu IRAffinity-1 machine. ^{1}H, ^{13}C, ^{19}F and ^{11}B NMR spectra were obtained on a Bruker DRX 500 spectrometer (Avance III HD console, Ascend 500 MHz magnet, BBO smart probe) at 500 MHz, 126 MHz, 471 MHz and 160 MHz, respectively. ^{1}H NMR for the evaluation of the base sensitivity were obtained on a Bruker AV 400 at 400 MHz. Chemical shifts are reported in ppm and coupling constants are reported in Hz with $CDCl_3$ referenced at 7.26 (^{1}H) and 77.0 ppm (^{13}C) and DMSO-d_6 referenced at 2.50 (^{1}H) and 39.5 (^{13}C). High-resolution mass spectra were obtained through analysis at the EPSRC UK National Mass Spectrometry Facility at Swansea University or at Glasgow University's School of Chemistry Mass Spectrometry Service. Crystal data was obtained at 123(2) K using an Oxford Diffraction Gemini instrument and monochromatic Mo radiation.

2. General experimental procedures

General Procedure A: Optimized conditions

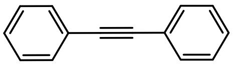

For example, synthesis of 1,2-diphenylethyne, **3a**.

To an oven-dried 5 mL microwave vessel was added $Pd(PPh_3)_2Cl_2$ (3.5 mg, 0.005 mmol, 2 mol %) and CuI (1.9 mg, 0.01 mmol, 4 mol %). The vessel was then capped and purged with N_2 before addition of Cyrene (0.5 mL, 0.5 M), Et_3N (38 µL, 0.275 mmol, 1.1 equiv), iodobenzene (27.9 µL, 0.25 mmol, 1 equiv), and phenylacetylene (28.8 µL, 0.263 mmol, 1.05 equiv). The reaction mixture was heated to 30 °C and maintained at this temperature with stirring for 1 h before the vessel was vented, and decapped. The solution was then diluted with EtOAc (10 mL), and washed with water (2 × 20 mL) and brine (2 × 20 mL). The organics were then passed through a hydrophobic frit and concentrated under reduced pressure to give a yellow oil, which was purified by flash chromatography (silica gel, 0–5% Et_2O in petroleum ether) to afford the title compound as a white solid (44.5 mg, quant.).

υ_{max} (solid): 3068, 1603, 1495, 1446 cm^{-1}.

^{1}H NMR ($CDCl_3$, 500 MHz): δ 7.55 (dd, *J* = 7.2, 1.9 Hz, 4H), 7.36 (m, 6H).

^{13}C NMR ($CDCl_3$, 126 MHz): δ 131.6, 128.4, 128.3, 123.3, 89.4.

HRMS: exact mass calculated for [M] ($C_{14}H_{10}$) requires *m/z* 178.0782, found *m/z* 178.0784.

Characterisation data is consistent with literature reported values.[2]

General Procedure B: Synthesis of indoles and benzofuran

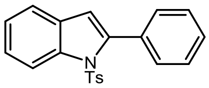

For example, synthesis of 2-phenyl-1-tosyl-1*H*-indole (**7a**).

To an oven-dried 5 mL microwave vessel was added $Pd(PPh_3)_2Cl_2$ (3.5 mg, 0.005 mmol, 2 mol %), CuI (1.9 mg, 0.01 mmol, 4 mol %), and *N*-(2-iodophenyl)-4-methylbenzenesulfonamide (93 mg, 0.25 mmol, 1 equiv). The vessel was then capped and purged with N_2 before addition of Cyrene (0.5 mL, 0.5 M), Et_3N (104 µL, 0.75 mmol, 3 equiv), and phenylacetylene (28.8 µL, 0.263 mmol, 1.05 equiv). The reaction mixture was heated to 30 °C and maintained at this temperature with stirring for 1 h. The reaction was subsequently heated to 60 °C and maintained at this temperature for 6 h before the vessel was vented and decapped. The solution was then diluted with EtOAc (10 mL), and washed with water (2 × 20 mL) and brine (2 × 20 mL). The organics were then passed through a hydrophobic frit and concentrated under reduced pressure to give a yellow oil, which was purified by flash chromatography (silica gel, 0–15% EtOAc in petroleum ether) to afford the title compound as a white solid (78.4 mg, 90%).

υ_{max} (solid): 3073, 1368, 1169 cm^{-1}.

^{1}H NMR ($CDCl_3$, 500 MHz): δ 8.33 (d, *J* = 8.4 Hz, 1H), 7.54–7.50 (m, 2H), 7.45 (t, *J* = 8.2 Hz, 4H), 7.38 (t, *J* = 7.4 Hz, 1H), 7.31–7.28 (m, 3H), 7.06 (d, *J* = 8.1 Hz, 2H), 6.56 (s, 1H), 2.31 (s, 3H).

^{13}C NMR ($CDCl_3$, 126 MHz): δ 144.5, 142.2, 138.3, 134.7, 132.4, 130.7, 130.4, 129.2, 128.7, 127.5, 126.8, 124.8, 124.3, 120.7, 116.7, 113.4, 21.5.

HRMS: exact mass calculated for $[M+H]^+$ ($C_{21}H_{18}NO_6S$) requires *m/z* 348.1058, found *m/z* 348.1061.

Characterisation data is consistent with literature reported values.[3]

3. Reaction optimization data

3.1. Variation of concentration

Reactions were carried out according to General Procedure A using $Pd(PPh_3)_2Cl_2$ (3.5 mg, 0.005 mmol, 2 mol %), CuI (1.9 mg, 0.01 mmol, 4 mol %), Cyrene (**X** M), Et_3N (104 µL, 0.75 mmol, 3 equiv), iodobenzene (27.9 µL, 0.25 mmol, 1 equiv), and phenylacetylene (28.8 µL, 0.263 mmol, 1.05 equiv). After stirring at 20 °C for 5 h, the reaction was subjected to the purification outlined in the General Procedure (silica gel, 0–5% Et_2O in petroleum ether) to afford the desired compound as a white solid.

Entry	Concentration (M)	Volume (mL)	Isolated yield (%)
**1**	0.3	0.83	98
**2**	0.1	2.5	94
**3**	0.5	0.5	100

3.2. Variation of the base

Reactions were carried out according to General Procedure A using $Pd(PPh_3)_2Cl_2$ (3.5 mg, 0.005 mmol, 2 mol %), CuI (1.9 mg, 0.01 mmol, 4 mol %), Cyrene (0.5 mL, 0.5 M), **Base** (**X** equiv), iodobenzene (27.9 µL, 0.25 mmol, 1 equiv), and phenylacetylene (28.8 µL, 0.263 mmol, 1.05 equiv). After stirring at 20 °C for 5 h, the reaction was subjected to the purification outlined in the General Procedure (silica gel, 0–5% Et_2O in petroleum ether) to afford the desired compound as a white solid.

Entry	Base (mass)	Equiv	Isolated yield (%)
**1**[a]	K_3PO_4 (159 mg)	3	-
**2**[a]	Cs_2CO_3 (245 mg)	3	-
**3**	DIPEA (97 mg)	3	85
**4**	Pyridine (59 mg)	3	0
**5**	Et_3N (28 mg)	1.1	98
**6**	Et_3N (38 mg)	1.5	94
**7**	Et_3N (51 mg)	2	92

[a] Formation of solid Cyrene dimer – product was not isolated

3.3. Variation of time and temperature

Reactions were carried out according to General Procedure A using $Pd(PPh_3)_2Cl_2$ (3.5 mg, 0.005 mmol, 2 mol %), CuI (1.9 mg, 0.01 mmol, 4 mol %), Cyrene (0.5 mL, 0.5 M), Et_3N (38 µL, 0.275 mmol, 1.1 equiv), iodobenzene (27.9 µL, 0.25 mmol, 1 equiv), and phenylacetylene (28.8 µL, 0.263 mmol, 1.05 equiv). After stirring at **X** °C for **X** h, the reaction was subjected to the purification outlined in the General Procedure (silica gel, 0–5% Et_2O in petroleum ether) to afford the desired compound as a white solid.

Entry	Temp. (°C)	Time (h)	Isolated yield (%)
**1**	20	1	86
**2**	20	3	94
**3**	20	5	98
**4**	25	1	91
**5**	30	1	96

3.4. Variation of solvent

Reactions were carried out according to General Procedure A using $Pd(PPh_3)_2Cl_2$ (3.5 mg, 0.005 mmol, 2 mol %), CuI (1.9 mg, 0.01 mmol, 4 mol %), **solvent** (0.5 mL, 0.5 M), Et_3N (38 µL, 0.275 mmol, 1.1 equiv), iodobenzene (27.9 µL, 0.25 mmol, 1 equiv), and phenylacetylene (28.8 µL, 0.263 mmol, 1.05 equiv). After stirring at 30 °C for 1 h, the reaction was subjected to the purification outlined in the General Procedure (silica gel, 0–5% Et_2O in petroleum ether) to afford the desired compound as a white solid.

Entry	Solvent	Isolated yield (%)
**1**	Cyrene	96
**2**	THF	81
**3**	DMF	87

4. Base sensitivity study

Base (0.07 mmol) was added to a test tube and Cyrene (0.5 mL) was added. The tube was then capped and the mixture stirred at **X** °C. After 24 h the reaction mixture was sampled and analysed by TLC (60% EtOAc in petroleum ether) and ^{1}H NMR and the resulting spectrum compared with that of Cyrene.

Base	Mass (mg)	Temperature (°C)	Reaction (Y/N)	Solid Formation
**KOAc**	7	25	N	x
		50	N	x
		100	N	x
**Pyridine**	6	25	N	x
		50	N	x
		100	N	x
**K_2CO_3**	10	25	N	x
		50	N	x
		100	Y	x
**DIPEA**	9	25	N	x
		50	N	x
		100	N	x
**Cs_2CO_3**	23	25	Y	√
		50	Y	√
		100	Y	√
**Et_3N**	7	25	N	x
		50	N	x
		100	N	x
**K_3PO_4**	15	25	Y	x
		50	Y	√
		100	Y	√
**DBU**	11	25	Y	√
		50	Y	√
		100	Y	√
**KOH**	4	25	Y	√

		50	Y	√
		100	Y	√
**^{t}BuOK**	8	25	Y	√
		50	Y	√
		100	Y	√
**NaH**	2	25	Y	√
		50	Y	√
		100	Y	√

5. Compound characterisation data

5.1. Preparation of intermediates

S1: *N*-(5-Chloro-2-iodophenyl)-4-methylbenzenesulfonamide

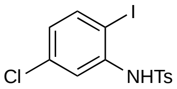

To a round-bottomed flask charged with 5-chloro-2-iodoaniline (1 g, 3.95 mmol, 1 equiv) was added a solution of 1:1 pyridine in CH_2Cl_2 (0.7 M, 40 mL) and the reaction mixture was cooled to 0 °C. 4-Methylbenzenesulfonyl chloride (750 mg, 3.95 mmol, 1 equiv) was added portionwise, and the reaction mixture was allowed to slowly warm to room temperature and then stirred for 24 h. Upon completion of the reaction, water (80 mL) and CH_2Cl_2 (80 mL) were added. The reaction mixture was separated and the organics were washed with 1 N NaOH (2 × 40 mL), 1 N HCl (2×40 mL), and brine (2 × 40 mL). The organics were then passed through a hydrophobic frit and concentrated under reduced pressure to give a crude residue, which was purified by flash chromatography (silica gel, 0–12% EtOAc in petroleum ether) to afford the title compound as an off white solid (890 mg, 52%).

^{1}H NMR ($CDCl_3$, 500 MHz): δ 7.72–7.65 (m, 3H), 7.57 (d, *J* = 8.5 Hz, 1H), 7.27 (d, *J* = 8.0 Hz, 2H), 6.88–6.80 (m, 2H), 2.42 (s, 3H).

^{13}C NMR ($CDCl_3$, 126 MHz): δ 144.1, 139.1, 138.1, 135.1, 135.1, 129.4, 127.0, 126.4, 121.4, 88.3, 21.2.

Characterisation data is consistent with literature reported values.[4]

S2: *N*-(3-Iodo-5-nitropyridin-2-yl)-4-methylbenzenesulfonamide

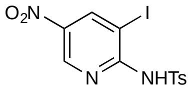

Prepared in two steps from 3-iodo-5-nitropyridin-2-amine:

Step 1: To a 25 mL three-necked flask charged with 5-nitropyridin-2-amine (1 g, 7.1 mmol, 1 equiv), was added concentrated sulfuric acid (12 mL, 0.6 M) and potassium iodate (653 mg, 2.8 mmol, 0.4 equiv) portionwise, before subsequent heating to 200 °C. Potassium iodide (1.18 g, 7.1 mmol, 1 equiv) was then added dropwise as an aqueous solution (4 mL), and the reaction mixture was stirred at 200 °C for 1.5 h. Upon completion, the reaction mixture was allowed to cool to room temperature before the slow addition of saturated sodium bicarbonate solution (20 mL) and EtOAc (20 mL). The reaction mixture was separated and the organics were washed with an aqueous solution of saturated $Na_2S_2O_3$ (2 × 30 mL). The organics were then passed through a hydrophobic frit and concentrated under reduced pressure to give a yellow solid, 3-iodo-5-nitropyridin-2-amine, which was used without further purification (1.64 g, 87 %).

Step 2: To a 100 mL round-bottomed flask charged with 3-iodo-5-nitropyridin-2-amine (1.29 g, 4.86 mmol, 1 equiv) was added THF (40 mL, 0.13 M) and the reaction mixture was cooled to 0 °C.

Sodium hydride (224 mg, 9.72 mmol, 2 equiv) was added portionwise and the reaction mixture was stirred at 0 °C for 20 minutes. 4-Methylbenzenesulfonyl chloride (1.09 g, 4.86 mmol, 1 equiv) was added portion wise, and the reaction mixture was allowed to slowly warm to room temperature and was stirred for 18 h. Upon completion of the reaction, water (50 mL) and CH_2Cl_2 (50 mL) were added and the reaction mixture was separated and the organics washed with 1 M NaOH (2 × 50 mL), 1 M HCl (2 × 50 mL), and brine (2 × 50 mL). The organics were passed through a hydrophobic frit and concentrated under reduced pressure to give a crude residue, which was purified by flash chromatography (silica gel, 0–30% EtOAc in petroleum ether) to afford the title compound as a yellow solid (1.43 g, 70%).

υ_{max} (solid): 3581, 3268, 3064, 2919, 1571, 1444, 1320 cm^{-1}.

^{1}H NMR (DMSO-d_6, 500 MHz): δ 8.66 (d, J = 2.6 Hz, 1H), 8.40 (d, J = 2.5 Hz, 1H), 7.74 (d, J = 8.1 Hz, 2H), 7.21 (d, J = 8.0 Hz, 2H), 3.35 (bs, 1H), 2.32 (s, 3H).

^{13}C NMR (DMSO-$d_{6,}$ 126 MHz): δ 161.9, 145.0, 142.3, 140.9, 140.7, 134.7, 128.9, 127.4, 86.7, 21.4.

HRMS: exact mass calculated for $[M+H]^+$ ($C_{12}H_{11}IN_3O_4S$) requires m/z 419.9509, found m/z 419.9510.

Characterisation data is consistent with literature reported values.[4]

5.2. Products from Table 1

3a: 1,2-Diphenylethyne

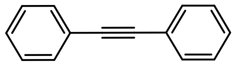

Prepared according to General Procedure A using $Pd(PPh_3)_2Cl_2$ (3.5 mg, 0.005 mmol, 2 mol %), CuI (1.9 mg, 0.01 mmol, 4 mol %), Cyrene (0.5 mL, 0.5 M), Et_3N (38 µL, 0.275 mmol, 1.1 equiv), iodobenzene (27.9 µL, 0.25 mmol, 1 equiv), and phenylacetylene (28.8 µL, 0.263 mmol, 1.05 equiv). After 1 h, the reaction mixture was subjected to the purification method outlined in the General Procedure (silica gel, 0–5% Et_2O in petroleum ether) to afford the title compound as a white solid (44.5 mg, quant.).

υ_{max} (solid): 3068, 1603, 1495, 1446 cm^{-1}.

^{1}H NMR ($CDCl_3$, 500 MHz): δ 7.55 (dd, J = 7.2, 1.9 Hz, 4H), 7.36 (m, 6H).

^{13}C NMR ($CDCl_3$, 126 MHz): δ 131.6, 128.4, 128.3, 123.3, 89.4.

HRMS: exact mass calculated for [M] ($C_{14}H_{10}$) requires m/z 178.0782, found m/z 178.0784.

Characterisation data is consistent with literature reported values.[2]

5.3. Products from Scheme 2a

3b: 1-Fluoro-4-(phenylethynyl)benzene

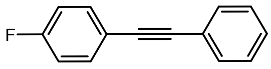

Prepared according to General Procedure A using $Pd(PPh_3)_2Cl_2$ (3.5 mg, 0.005 mmol, 2 mol %), CuI (1.9 mg, 0.01 mmol, 4 mol %), Cyrene (0.5 mL, 0.5 M), Et_3N (38 µL, 0.275 mmol, 1.1 equiv), 4-fluoro-iodobenzene (28.8 µL, 0.25 mmol, 1 equiv), and phenylacetylene (28.8 µL, 0.263 mmol, 1.05 equiv). After 1 h, the reaction mixture was subjected to the purification method outlined in the General Procedure (silica gel, 0–5% Et_2O in petroleum ether) to afford the title compound as a white solid (48.8 mg, quant.).

Prepared according to General Procedure A using $Pd(PPh_3)_2Cl_2$ (3.5 mg, 0.005 mmol, 2 mol %), CuI (1.9 mg, 0.01 mmol, 4 mol %), DMF (0.5 mL, 0.5 M), Et_3N (38 µL, 0.275 mmol, 1.1 equiv), 4-fluoro-iodobenzene (28.8 µL, 0.25 mmol, 1 equiv), and phenylacetylene (28.8 µL, 0.263 mmol, 1.05 equiv).

After 1 h, the reaction mixture was subjected to the purification method outlined in the General Procedure (silica gel, 0–5% Et_2O in petroleum ether) to afford the title compound as a white solid (46.9 mg, 96%).
υ_{max} (solid): 2921, 1595, 1508, 1217 cm^{-1}.
^{1}H NMR ($CDCl_3$, 500 MHz): δ 7.55–7.50 (m, 4H), 7.38–7.33 (m, 3H), 7.05 (t, J = 8.7 Hz, 2H).
^{13}C NMR ($CDCl_3$, 126 MHz): δ 162.5 (d, $^1J_{CF}$ = 249.6 Hz), 133.5 (d, $^3J_{CF}$ = 8.2 Hz), 131.6, 128.4, 128.4, 123.3, 119.4 (d, J_{CF} = 3.4 Hz), 115.7 (d, $^2J_{CF}$ = 22.4 Hz), 89.1, 88.3.
^{19}F NMR ($CDCl_3$, 471 MHz): δ -110.98.
HRMS: exact mass calculated for [M] ($C_{14}H_9F$) requires *m/z* 196.0688, found *m/z* 196.0689.
Characterisation data is consistent with literature reported values.[5]

3c: 1-Nitro-4-(phenylethynyl)benzene

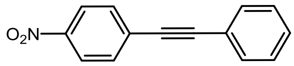

Prepared according to General Procedure A using $Pd(PPh_3)_2Cl_2$ (3.5 mg, 0.005 mmol, 2 mol %), CuI (1.9 mg, 0.01 mmol, 4 mol %), Cyrene (0.5 mL, 0.5 M), Et_3N (38 µL, 0.275 mmol, 1.1 equiv), 4-nitro-iodobenzene (62.3 mg, 0.25 mmol, 1 equiv), and phenylacetylene (28.8 µL, 0.263 mmol, 1.05 equiv). After 1 h, the reaction mixture was subjected to the purification method outlined in the General Procedure (silica gel, 0–5% Et_2O in petroleum ether) to afford the title compound as an off white solid (48.8 mg, quant.).

Prepared according to General Procedure A using $Pd(PPh_3)_2Cl_2$ (3.5 mg, 0.005 mmol, 2 mol %), CuI (1.9 mg, 0.01 mmol, 4 mol %), Cyrene (0.5 mL, 0.5 M), Et_3N (38 µL, 0.275 mmol, 1.1 equiv), 4-nitro-bromobenzene (50.5 mg, 0.25 mmol, 1 equiv), and phenylacetylene (28.8 µL, 0.263 mmol, 1.05 equiv). After 1 h, the reaction mixture was subjected to the purification method outlined in the General Procedure (silica gel, 0–5% Et_2O in petroleum ether) to afford the title compound as an off white solid (14.6 mg, 28%).
υ_{max} (solid): 3107, 2926, 2217, 1593, 1511 cm^{-1}.
^{1}H NMR ($CDCl_3$, 500 MHz): δ 8.22 (d, J = 8.9 Hz, 2H), 7.67 (d, J = 8.9 Hz, 2H), 7.58–7.54 (m, 2H), 7.39 (dd, J = 5.3, 1.8 Hz, 3H).
^{13}C NMR ($CDCl_3$, 126 MHz): δ 147.0, 132.3, 131.9, 130.4, 129.3, 128.6, 123.7, 122.1, 94.7, 87.6.
HRMS: exact mass calculated for $[M+H]^+$ ($C_{14}H_{10}NO_2$) requires *m/z* 224.0712, found *m/z* 224.0714.
Characterisation data is consistent with literature reported values.[5]

3d: 1-Methoxy-4-(phenylethynyl)benzene

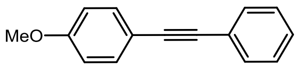

Prepared according to General Procedure A using $Pd(PPh_3)_2Cl_2$ (3.5 mg, 0.005 mmol, 2 mol %), CuI (1.9 mg, 0.01 mmol, 4 mol %), Cyrene (0.5 mL, 0.5 M), Et_3N (38 µL, 0.275 mmol, 1.1 equiv), 4-iodoanisole (58.5 mg, 0.25 mmol, 1 equiv), and phenylacetylene (28.8 µL, 0.263 mmol, 1.05 equiv). After 1 h, the reaction mixture was subjected to the purification method outlined in the General Procedure (silica gel, 0–15% Et_2O in petroleum ether) to afford the title compound as an off white solid (51.9 mg, quant.).
υ_{max} (solid): 3014, 2841, 2217, 1509 cm^{-1}.
^{1}H NMR ($CDCl_3$, 500 MHz): δ 7.51 (dt, J = 3.9, 2.1 Hz, 2H), 7.49–7.46 (m, 2H), 7.36–7.29 (m, 3H), 6.88 (d, J = 8.8 Hz, 2H), 3.83 (s, 3H).

^{13}C NMR ($CDCl_3$, 126 MHz): δ 159.6, 133.1, 131.5, 128.3, 127.9, 123.6, 115.4, 114.0, 89.4, 88.1, 55.3.
HRMS: exact mass calculated for $[2M+H]^+$ ($C_{30}H_{25}O_2$) requires *m/z* 417.1855, found *m/z* 417.1847.
Characterisation data is consistent with literature reported values.[5]

3e: 4-(Phenylethynyl)phenol

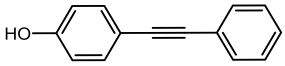

Prepared according to General Procedure A using $Pd(PPh_3)_2Cl_2$ (3.5 mg, 0.005 mmol, 2 mol %), CuI (1.9 mg, 0.01 mmol, 4 mol %), Cyrene (0.5 mL, 0.5 M), Et_3N (70 µL, 0.5 mmol, 2 equiv), 4-iodophenol (55 mg, 0.25 mmol, 1 equiv), and phenylacetylene (28.8 µL, 0.263 mmol, 1.05 equiv). After 1 h, the reaction mixture was subjected to the purification method outlined in the General Procedure (silica gel, 0–10% Et_2O in petroleum ether) to afford the title compound as an off white solid (32.6 mg, 68%).
υ_{max} (solid): 3412, 3059, 1513, 1254 cm^{-1}.
^{1}H NMR ($CDCl_3$, 500 MHz): δ 7.51 (dd, *J* = 7.7, 1.4 Hz, 2H), 7.43 (d, *J* = 8.6 Hz, 2H), 7.33 (m, 3H), 6.81 (d, *J* = 8.6 Hz, 2H).
^{13}C NMR ($CDCl_3$, 126 MHz): δ 155.7, 133.3, 131.5, 128.3, 127.9, 123.6, 115.7, 115.5, 89.2, 88.1.
HRMS: exact mass calculated for $[M+H]^+$ ($C_{15}H_{13}O$) requires *m/z* 209.0966, found *m/z* 209.1008.
Characterisation data is consistent with literature reported values.[6]

3f: 1-Methoxy-3-(phenylethynyl)benzene

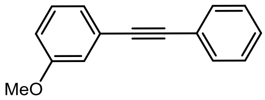

Prepared according to General Procedure A using $Pd(PPh_3)_2Cl_2$ (3.5 mg, 0.005 mmol, 2 mol %), CuI (1.9 mg, 0.01 mmol, 4 mol %), Cyrene (0.5 mL, 0.5 M), Et_3N (38 µL, 0.275 mmol, 1.1 equiv), 3-iodoanisole (29.8 µL, 0.25 mmol, 1 equiv), and phenylacetylene (28.8 µL, 0.263 mmol, 1.05 equiv). After 1 h, the reaction mixture was subjected to the purification method outlined in the General Procedure (silica gel, 0–5% Et_2O in petroleum ether) to afford the title compound as a yellow oil (51.4 mg, 99%).
υ_{max} (liquid film): 2937, 2838 cm^{-1}.
^{1}H NMR ($CDCl_3$, 500 MHz): δ 7.57–7.53 (m, 2H), 7.35 (dd, *J* = 4.9, 2.4 Hz, 3H), 7.27 (t, *J* = 7.9 Hz, 1H), 7.15 (d, *J* = 7.6 Hz, 1H), 7.08 (s, 1H), 6.91 (dd, *J* = 8.3, 2.0 Hz, 1H), 3.83 (s, 3H).
^{13}C NMR ($CDCl_3$, 126 MHz): δ 159.4, 131.7, 129.4, 128.4, 128.3, 124.3, 124.2, 123.2, 116.4, 114.9, 89.3, 89.2, 55.3.
HRMS: exact mass calculated for $[M+Na]^+$ ($C_{14}H_{11}O$) requires *m/z* 195.0810, found *m/z* 195.0813.
Characterisation data is consistent with literature reported values.[2]

3g: 1-Chloro-3-(phenylethynyl)benzene

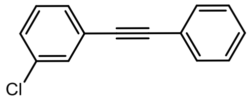

Prepared according to General Procedure A using $Pd(PPh_3)_2Cl_2$ (3.5 mg, 0.005 mmol, 2 mol %), CuI (1.9 mg, 0.01 mmol, 4 mol %), Cyrene (0.5 mL, 0.5 M), Et_3N (38 µL, 0.275 mmol, 1.1 equiv), 3-

chloro-iodobenzene (30.9 µL, 0.25 mmol, 1 equiv), and phenylacetylene (28.8 µL, 0.263 mmol, 1.05 equiv). After 1 h, the reaction mixture was subjected to the purification method outlined in the General Procedure (silica gel, 0–5% Et_2O in petroleum ether) to afford the title compound as a yellow oil (53.5 mg, 82%).
υ_{max} (liquid film): 3064, 2224, 884 cm^{-1}.
^1H NMR ($CDCl_3$, 500 MHz): δ 7.55–7.51 (m, 3H), 7.41 (dt, *J* = 7.3, 1.4 Hz, 1H), 7.36 (dd, *J* = 4.9, 1.7 Hz, 3H), 7.31 (dt, *J* = 8.0, 1.5 Hz, 1H), 7.29 (d, *J* = 7.5 Hz, 1H).
^{13}C NMR ($CDCl_3$, 126 MHz): δ 134.4, 131.9, 131.6, 129.9, 129.8, 128.8, 128.7, 128.6, 125.2, 122.9, 90.7, 88.1.
HRMS: exact mass calculated for [M] ($C_{14}H_9Cl$) requires *m/z* 212.0393, found *m/z* 212.0395.
Characterisation data is consistent with literature reported values.[7]

3h: 1-Nitro-3-(phenylethynyl)benzene

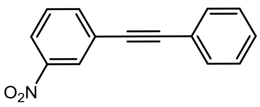

Prepared according to General Procedure A using $Pd(PPh_3)_2Cl_2$ (3.5 mg, 0.005 mmol, 2 mol %), CuI (1.9 mg, 0.01 mmol, 4 mol %), Cyrene (0.5 mL, 0.5 M), Et_3N (38 µL, 0.275 mmol, 1.1 equiv), 3-nitro-iodobenzene (62.3 mg, 0.25 mmol, 1 equiv), and phenylacetylene (28.8 µL, 0.263 mmol, 1.05 equiv). After 1 h, the reaction mixture was subjected to the purification method outlined in the General Procedure (silica gel, 0–5% Et_2O in petroleum ether) to afford the title compound as a white solid (55.2 mg, 99%).
υ_{max} (solid): 3083, 2213, 1517, 1349 cm^{-1}.
^1H NMR ($CDCl_3$, 500 MHz): δ 8.40–8.37 (m, 1H), 8.19 (m, 1H), 7.83 (d, *J* = 7.7 Hz, 1H), 7.56 (m, 3H), 7.40 (m, 3H).
^{13}C NMR ($CDCl_3$, 126 MHz): δ 148.2, 137.2, 131.8, 129.4, 129.1, 128.5, 126.4, 125.2, 122.9, 122.2, 91.9, 86.9.
HRMS: exact mass calculated for $[M+H]^+$ ($C_{14}H_{10}NO_2$) requires *m/z* 224.0712, found *m/z* 224.0710.
Characterisation data is consistent with literature reported values.[5]

3i: 1-Methyl-2-(phenylethynyl)benzene

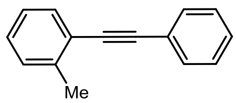

Prepared according to General Procedure A using $Pd(PPh_3)_2Cl_2$ (3.5 mg, 0.005 mmol, 2 mol %), CuI (1.9 mg, 0.01 mmol, 4 mol %), Cyrene (0.5 mL, 0.5 M), Et_3N (38 µL, 0.275 mmol, 1.1 equiv), 2-iodotoluene (31.8 µL, 0.25 mmol, 1 equiv), and phenylacetylene (28.8 µL, 0.263 mmol, 1.05 equiv). After 24 h, the reaction mixture was subjected to purification by reverse phase chromatography (C18 cartridge, 20–65% MeCN in water) to afford the title compound as a yellow oil (40 mg, 83%).
υ_{max} (liquid film): 3023, 2924, 2855, 2217, 1496 cm^{-1}.
^1H NMR ($CDCl_3$, 500 MHz): δ 7.56–7.53 (m, 2H), 7.50 (d, *J* = 7.5 Hz, 1H), 7.35 (m, 3H), 7.24 (d, *J* = 3.9 Hz, 2H), 7.17 (m, 1H), 2.52 (s, 3H).
^{13}C NMR ($CDCl_3$, 126 MHz): δ 140.3, 131.9, 131.7, 129.6, 128.5, 128.5, 128.3, 125.7, 123.7, 123.2, 93.5, 88.5, 20.9.
HRMS: exact mass calculated for [M] ($C_{15}H_{12}$) requires *m/z* 192.0939, found *m/z* 192.0935.
Characterisation data is consistent with literature reported values.[5]

3j: 1-Chloro-2-(phenylethynyl)benzene

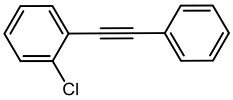

Prepared according to General Procedure A using $Pd(PPh_3)_2Cl_2$ (3.5 mg, 0.005 mmol, 2 mol %), CuI (1.9 mg, 0.01 mmol, 4 mol %), Cyrene (0.5 mL, 0.5 M), Et_3N (38 µL, 0.275 mmol, 1.1 equiv), 2-chloro-iodobenzene (30.5 µL, 0.25 mmol, 1 equiv), and phenylacetylene (28.8 µL, 0.263 mmol, 1.05 equiv). After 24 h, the reaction mixture was subjected to purification by reverse phase chromatography (C18 cartridge, 20–65% MeCN in water) to afford the title compound as a yellow oil (54.9 mg, quant.).

υ_{max} (liquid film): 3060, 2926, 2224, 1495 cm^{-1}.

^{1}H NMR (DMSO-d_6, 500 MHz): δ 7.69 (dd, *J* = 7.5, 1.7 Hz, 1H), 7.62–7.58 (m, 3H), 7.48–7.41 (m, 5H).

^{13}C NMR (DMSO-d_6, 126 MHz): δ 135.1, 133.8, 131.9, 130.9, 129.9, 129.8, 129.3, 127.9, 122.4, 122.3, 94.8, 86.4.

HRMS: exact mass calculated for [M] ($C_{14}H_9Cl$) requires *m/z* 212.0393, found *m/z* 212.0385.

Characterisation data is consistent with literature reported values.[2]

3k: 2-(Phenylethynyl)thiophene

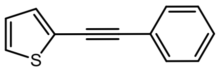

Prepared according to General Procedure A using $Pd(PPh_3)_2Cl_2$ (3.5 mg, 0.005 mmol, 2 mol %), CuI (1.9 mg, 0.01 mmol, 4 mol %), Cyrene (0.5 mL, 0.5 M), Et_3N (38 µL, 0.275 mmol, 1.1 equiv), 2-iodothiophene (27.6 µL, 0.25 mmol, 1 equiv), and phenylacetylene (28.8 µL, 0.263 mmol, 1.05 equiv). After 1 h, the reaction mixture was subjected to the purification method outlined in the General Procedure (silica gel, 0–5% Et_2O in petroleum ether) to afford the title compound as an off white solid (41.4 mg, 92%).

υ_{max} (liquid film): 3088, 2204 cm^{-1}.

^{1}H NMR ($CDCl_3$, 500 MHz): δ 7.54–7.50 (m, 2H), 7.35 (dd, *J* = 5.2, 1.9 Hz, 3H), 7.31–7.28 (m, 2H), 7.02 (dd, *J* = 5.0, 3.8 Hz, 1H).

^{13}C NMR ($CDCl_3$, 126 MHz): δ 131.9, 131.4, 128.4, 128.4, 127.3, 127.1, 123.4, 122.9, 93.0, 82.6.

HRMS: exact mass calculated for [M] ($C_{12}H_8S$) requires *m/z* 184.0347, found *m/z* 184.0348.

Characterisation data is consistent with literature reported values.[2]

3l: 2-Nitro-5-(phenylethynyl)pyridine

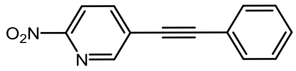

Prepared according to General Procedure A using $Pd(PPh_3)_2Cl_2$ (3.5 mg, 0.005 mmol, 2 mol %), CuI (1.9 mg, 0.01 mmol, 4 mol %), Cyrene (0.5 mL, 0.5 M), Et_3N (38 µL, 0.275 mmol, 1.1 equiv), 5-bromo-2-nitropyridine (50.8 mg, 0.25 mmol, 1 equiv), and phenylacetylene (28.8 µL, 0.263 mmol, 1.05 equiv). After 1 h, the reaction mixture was subjected to the purification method outlined in the General Procedure (silica gel, 0–15% Et_2O in petroleum ether) to afford the title compound as a white solid (51.4 mg, 92%).

υ_{max} (solid): 3058, 2219, 1532, 1348 cm^{-1}.

^{1}H NMR ($CDCl_3$, 500 MHz): δ 8.73 (d, *J* = 1.6 Hz, 1H), 8.26 (d, *J* = 8.4 Hz, 1H), 8.10 (dd, *J* = 8.4, 2.0 Hz, 1H), 7.58 (dd, *J* = 7.7, 1.4 Hz, 2H), 7.44 – 7.38 (m, 3H).

^{13}C NMR ($CDCl_3$, 126 MHz): δ 154.8, 151.1, 141.8, 131.9, 129.8, 128.7, 126.7, 121.4, 117.7, 97.9, 84.2.
HRMS: exact mass calculated for $[M+H]^+$ ($C_{13}H_9N_2O_2$) requires *m/z* 225.0664, found *m/z* 225.0670.

3m: 5-Chloro-2-(phenylethynyl)pyridine

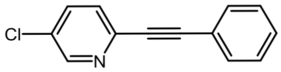

Prepared according to General Procedure A using $Pd(PPh_3)_2Cl_2$ (3.5 mg, 0.005 mmol, 2 mol %), CuI (1.9 mg, 0.01 mmol, 4 mol %), Cyrene (0.5 mL, 0.5 M), Et_3N (38 µL, 0.275 mmol, 1.1 equiv), 3-chloro-6-iodopyridine (59.8 mg, 0.25 mmol, 1 equiv), and phenylacetylene (28.8 µL, 0.263 mmol, 1.05 equiv). After 1 h, the reaction mixture was subjected to the purification method outlined in the General Procedure (silica gel, 0–5% Et_2O in petroleum ether) to afford the title compound as a white solid (54.5 mg, quant.).
υ_{max} (solid): 3040, 2221, 1493, 1459 cm^{-1}.
^1H NMR ($CDCl_3$, 500 MHz): δ 8.58 (d, *J* = 1.8 Hz, 1H), 7.67 (dd, *J* = 8.4, 2.4 Hz, 1H), 7.60 (dd, *J* = 7.5, 1.9 Hz, 2H), 7.48 (d, *J* = 8.4 Hz, 1H), 7.40–7.36 (m, 3H).
^{13}C NMR ($CDCl_3$, 126 MHz): δ 149.1, 141.5, 136.0, 132.1, 131.3, 129.2, 128.5, 127.7, 121.9, 90.4, 87.6.
HRMS: exact mass calculated for $[M+H]^+$ ($C_{13}H_9NCl$) requires *m/z* 214.0418, found *m/z* 214.0421.

3n: 2-(Phenylethynyl)-1,8-naphthyridine

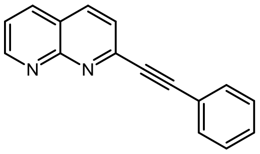

Prepared according to General Procedure A using $Pd(PPh_3)_2Cl_2$ (3.5 mg, 0.005 mmol, 2 mol %), CuI (1.9 mg, 0.01 mmol, 4 mol %), Cyrene (0.5 mL, 0.5 M), Et_3N (38 µL, 0.275 mmol, 1.1 equiv), 2-bromo-1,8-naphthyridine (52.3 mg, 0.25 mmol, 1 equiv), and phenylacetylene (28.8 µL, 0.263 mmol, 1.05 equiv). After 1 h, the reaction mixture was subjected to the purification method outlined in the General Procedure (silica gel, 0–65% Et_2O in petroleum ether) to afford the title compound as a white solid (54.3 mg, 94%).
υ_{max} (solid): 3049, 3008, 2211, 1601, 1498 cm^{-1}.
^1H NMR ($CDCl_3$, 500 MHz): δ 9.15 (s, 1H), 8.17 (d, *J* = 8.2 Hz, 2H), 7.69–7.64 (m, 3H), 7.48 (dd, *J* = 7.7, 3.9 Hz, 1H), 7.43–7.37 (m, 3H).
^{13}C NMR ($CDCl_3$, 126 MHz): δ 156.1, 154.3, 146.9, 137.2, 136.6, 132.4, 129.5, 128.5, 125.4, 122.3, 121.9, 91.6, 89.3. Quaternary carbon at ring junction not observed.
HRMS: exact mass calculated for $[M+H]^+$ ($C_{16}H_{11}N_2$) requires *m/z* 231.0922, found *m/z* 231.0923.

3o: 2-Chloro-6-(phenylethynyl)pyridine

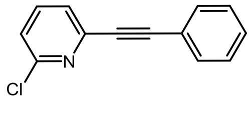

Prepared according to General Procedure A using $Pd(PPh_3)_2Cl_2$ (3.5 mg, 0.005 mmol, 2 mol %), CuI (1.9 mg, 0.01 mmol, 4 mol %), Cyrene (0.5 mL, 0.5 M), Et_3N (38 µL, 0.5 mmol, 1.1 equiv), 2-bromo-6-chloropyridine (48 mg, 0.25 mmol, 1 equiv), and phenylacetylene (28.8 µL, 0.263 mmol, 1.05 equiv). After 1 h, the reaction mixture was subjected to the purification method outlined in the

General Procedure (silica gel, 0–10% Et_2O in petroleum ether) to afford the title compound as an off white solid (52.3 mg, quant.).
υ_{max} (solid): 3059, 2960, 2226, 1577, 1435 cm^{-1}.
^1H NMR ($CDCl_3$, 500 MHz): δ 7.63 (t, *J* = 7.8 Hz, 1H), 7.60–7.56 (m, 2H), 7.44 (d, *J* = 7.6 Hz, 1H), 7.37 (q, *J* = 5.7 Hz, 3H), 7.28 (d, *J* = 8.0 Hz, 1H).
^{13}C NMR ($CDCl_3$, 126 MHz): δ 151.4, 143.6, 138.7, 132.1, 129.3, 128.5, 125.7, 123.6, 121.8, 90.7, 87.5.
HRMS: exact mass calculated for $[M+H]^+$ ($C_{13}H_9NCl$) requires *m/z* 214.0424, found *m/z* 214.0427.
^1H NMR and HRMS data is consistent with literature reported values.[8]
3p: 1-Methyl-5-(phenylethynyl)-1*H*-indole

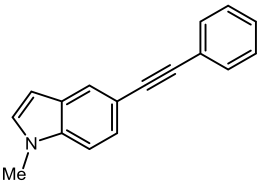

Prepared according to General Procedure A using $Pd(PPh_3)_2Cl_2$ (2.6 mg, 0.004 mmol, 2 mol %), CuI (1.4 mg, 0.007 mmol, 4 mol %), Cyrene (0.5 mL, 0.5 M), Et_3N (28 µL, 0.20 mmol, 1.1 equiv), 5-iodo-1-methyl-1*H*-indole (47 mg, 0.18 mmol, 1 equiv), and phenylacetylene (21 µL, 0.19 mmol, 1.05 equiv). After 1 h, the reaction mixture was subjected to the purification method outlined in the General Procedure (silica gel, 0–10% Et_2O in petroleum ether) to afford the title compound as an off white solid (27.7 mg, 67%).
υ_{max} (solid): 3051, 2926, 2208, 1597, 1496 cm^{-1}.
^1H NMR ($CDCl_3$, 500 MHz): δ 7.85 (s, 1H), 7.57–7.54 (m, 2H), 7.41 (dd, *J* = 8.5, 1.2 Hz, 1H), 7.35 (t, *J* = 7.2 Hz, 2H), 7.30 (t, *J* = 8.7 Hz, 2H), 7.08 (d, *J* = 3.1 Hz, 1H), 6.49 (d, *J* = 2.7 Hz, 1H), 3.80 (s, 3H).
^{13}C NMR ($CDCl_3$, 126 MHz): δ 136.4, 131.5, 129.8, 128.4, 128.3, 127.7, 125.2, 124.8, 124.1, 113.8, 109.3, 101.3, 91.2, 87.0, 32.9.
HRMS: exact mass calculated for [M] ($C_{17}H_{13}N$) requires *m/z* 231.1048, found *m/z* 231.1057.
Characterisation data is consistent with literature reported values.[9]

5.4. Products from Scheme 2b

3q: Phenylethynylboronic acid, MIDA ester

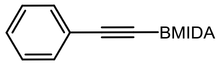

Prepared according to General Procedure A using $Pd(PPh_3)_2Cl_2$ (3.5 mg, 0.005 mmol, 2 mol %), CuI (1.9 mg, 0.01 mmol, 4 mol %), Cyrene (0.5 mL, 0.5 M), Et_3N (38 µL, 0.275 mmol, 1.1 equiv), iodobenzene (27.9 µL, 0.25 mmol, 1 equiv), and ethynyl boronic acid, MIDA ester (47.5 mg, 0.263 mmol, 1.05 equiv). After 1 h, the reaction mixture was subjected to the purification method outlined in the General Procedure (silica gel, 0–60% EtOAc in petroleum ether) to afford the title compound as an off white solid (61.3 mg. 95%).

Prepared according to General Procedure A using $Pd(PPh_3)_2Cl_2$ (3.5 mg, 0.005 mmol, 2 mol %), CuI (1.9 mg, 0.01 mmol, 4 mol %), DMF (0.5 mL, 0.5 M), Et_3N (38 µL, 0.275 mmol, 1.1 equiv), iodobenzene (27.9 µL, 0.25 mmol, 1 equiv), and ethynyl boronic acid, MIDA ester (47.5 mg, 0.263 mmol, 1.05 equiv). After 1 h, the reaction mixture was subjected to the purification method outlined

in the General Procedure (silica gel, 0-60% EtOAc in petroleum ether) to afford the title compound as an off white solid (61.2 mg. 95%).
υ_{max} (solid): 3025, 2198, 1768, 1493 cm^{-1}.
^{1}H NMR (DMSO-d_6, 500 MHz): δ 7.51–7.48 (m, 2H), 7.42–7.37 (m, 3H), 4.32 (d, *J* = 17.1 Hz, 2H), 4.15 (d, *J* = 17.1 Hz, 2H), 3.08 (s, 3H).
^{13}C NMR (DMSO-d_6, 126 MHz): δ 169.1, 132.0, 129.4, 129.1, 129.1, 122.9, 99.9, 61.9, 48.4. Carbon bearing boron not observed.
^{11}B NMR (DMSO-d_6, 160 MHz): δ 6.24.
HRMS: exact mass calculated for $[M+NH_4]^+$ ($C_{13}H_{16}BN_2O_4$) requires *m/z* 275.1202, found *m/z* 275.1198.
Characterisation data is consistent with literature reported values.[10]
3r: Trimethyl(phenylethynyl)silane

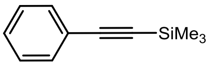

Prepared according to General Procedure A using $Pd(PPh_3)_2Cl_2$ (3.5 mg, 0.005 mmol, 2 mol %), CuI (1.9 mg, 0.01 mmol, 4 mol %), Cyrene (0.5 mL, 0.5 M), Et_3N (38 µL, 0.275 mmol, 1.1 equiv), 2-iodobenzene (27.9 µL, 0.25 mmol, 1 equiv), and ethynyltrimethylsilane (37 µL, 0.263 mmol, 1.05 equiv). After 1 h, the reaction mixture was subjected to the purification method outlined in the General Procedure (silica gel, 0–1% Et_2O in petroleum ether) to afford the title compound as a colourless oil (44 mg, quant.).
υ_{max} (liquid film): 2962, 2161, 1491, 1251 cm^{-1}.
^{1}H NMR ($CDCl_3$, 500 MHz): δ 7.50–7.47 (m, 2H), 7.34–7.29 (m, 3H), 0.27 (s, 9H).
^{13}C NMR ($CDCl_3$, 126 MHz): δ 131.9, 128.5, 128.2, 123.1, 105.1, 94.1, -0.01.
HRMS: exact mass calculated for [M] ($C_{11}H_{14}Si$) requires *m/z* 174.0865, found *m/z* 174.0866.
Characterisation data is consistent with literature reported values.[11]

3s: 4-Phenylbut-3-yn-1-yl 4-methylbenzenesulfonate

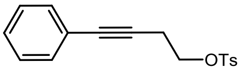

Prepared according to General Procedure A using $Pd(PPh_3)_2Cl_2$ (3.5 mg, 0.005 mmol, 2 mol %), CuI (1.9 mg, 0.01 mmol, 4 mol %), Cyrene (0.5 mL, 0.5 M), Et_3N (38 µL, 0.275 mmol, 1.1 equiv), 2-iodobenzene (27.9 µL, 0.25 mmol, 1 equiv), and 3-butynyl-*p*-toluenesulfonate (46.3 µL, 0.263 mmol, 1.05 equiv). After 1 h, the reaction mixture was subjected to the purification method outlined in the General Procedure (silica gel, 0-30% EtOAc in petroleum ether) to afford the title compound as a colourless oil (60.7 mg, 81%).
υ_{max} (liquid film): 2924, 2980, 1493, 1361, 1176 cm^{-1}.
^{1}H NMR ($CDCl_3$, 500 MHz): δ 7.79 (d, *J* = 8.3 Hz, 2H), 7.32–7.28 (m, 3H), 7.28–7.23 (m, 4H), 4.16 (t, *J* = 7.0 Hz, 2H), 2.75 (t, *J* = 7.0 Hz, 2H), 2.39 (s, 3H).
^{13}C NMR ($CDCl_3$, 126 MHz): δ 144.9, 132.9, 131.7, 129.9, 128.2, 128.2, 127.9, 122.9, 83.8, 82.7, 67.8, 21.6, 20.4.
HRMS: exact mass calculated for $[M+Na]^+$ ($C_{17}H_{16}O_3SNa$) requires *m/z* 323.0712, found *m/z* 323.0702.
Characterisation data is consistent with literature reported values.[12]

3t: *N*,*N*-Dimethyl-3-phenylprop-2-yn-1-amine

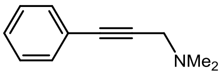

Prepared according to General Procedure A using $Pd(PPh_3)_2Cl_2$ (3.5 mg, 0.005 mmol, 2 mol %), CuI (1.9 mg, 0.01 mmol, 4 mol %), Cyrene (0.5 mL, 0.5 M), Et_3N (38 µL, 0.275 mmol, 1.1 equiv), 2-iodobenzene (27.9 µL, 0.25 mmol, 1 equiv), and dimethyl(prop-2-yne)amine (28 µL, 0.263 mmol, 1.05 equiv). After 1 h, the reaction mixture was subjected to purification by SCX (MeOH in 3M ammonium MeOH) to afford the title compound as a yellow oil (23.6 mg, 60%).

υ_{max} (liquid film): 3058, 2941, 2824, 2775, 1690, 1493 cm^{-1}.

^1H NMR ($CDCl_3$, 500 MHz): δ 7.46–7.42 (m, 2H), 7.32–7.28 (m, 3H), 3.49 (s, 2H), 2.39 (s, 6H).

^{13}C NMR ($CDCl_3$, 126 MHz): δ 131.7, 128.3, 128.1, 123.2, 85.4, 84.4, 48.6, 44.2.

HRMS: exact mass calculated for $[M+H]^+$ ($C_{11}H_{14}N$) requires *m/z* 160.1126, found *m/z* 160.1125.

Characterisation data is consistent with literature reported values.[13]

3u: Pent-1-yn-1-ylbenzene

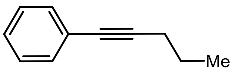

Prepared according to General Procedure A using $Pd(PPh_3)_2Cl_2$ (3.5 mg, 0.005 mmol, 2 mol %), CuI (1.9 mg, 0.01 mmol, 4 mol %), Cyrene (0.5 mL, 0.5 M), Et_3N (38 µL, 0.275 mmol, 1.1 equiv), 2-iodobenzene (27.9 µL, 0.25 mmol, 1 equiv), and 1-pentyne (25.8 µL, 0.263 mmol, 1.05 equiv). After 1 h, the reaction mixture was subjected to the purification method outlined in the General Procedure (silica gel, 0–1% Et_2O in petroleum ether) to afford the title compound as a yellow oil (34.5 mg, 96%).

υ_{max} (liquid film): 3058, 2963, 2934, 2872, 2237, 1601, 1491 cm^{-1}.

^1H NMR ($CDCl_3$, 500 MHz): δ 7.32 (dd, *J* = 7.5, 1.9 Hz, 2H), 7.22–7.17 (m, 3H), 2.31 (t, *J* = 7.0 Hz, 2H), 1.56 (h, *J* = 7.3 Hz, 2H), 0.98 (t, *J* = 7.4 Hz, 3H).

^{13}C NMR ($CDCl_3$, 126 MHz): δ 131.6, 128.2, 127.5, 124.1, 90.3, 80.7, 22.2, 21.4, 13.6.

HRMS: exact mass calculated for [M] ($C_{11}H_{12}$) requires *m/z* 144.0939, found *m/z* 144.0941.

Characterisation data is consistent with literature reported values.[14]

3v: (Cyclopropylethynyl)benzene

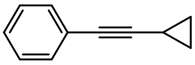

Prepared according to General Procedure A using $Pd(PPh_3)_2Cl_2$ (3.5 mg, 0.005 mmol, 2 mol %), CuI (1.9 mg, 0.01 mmol, 4 mol %), Cyrene (0.5 mL, 0.5 M), Et_3N (38 µL, 0.275 mmol, 1.1 equiv), 2-iodobenzene (27.9 µL, 0.25 mmol, 1 equiv), and ethynylcyclopropane (22 µL, 0.263 mmol, 1.05 equiv). After 1 h, the reaction mixture was subjected to the purification method outlined in the General Procedure (silica gel, 0–1% Et_2O in petroleum ether) to afford the title compound as a colourless oil (30.3 mg, 85%).

υ_{max} (liquid film): 3034, 2924, 2219, 1597, 1513 cm^{-1}.

^1H NMR ($CDCl_3$, 500 MHz): δ 7.42–7.38 (m, 2H), 7.30–7.26 (m, 3H), 1.47 (m, 1H), 0.91–0.87 (m, 2H), 0.83 (m, 2H).

^{13}C NMR ($CDCl_3$, 126 MHz): δ 131.6, 128.1, 127.4, 123.9, 93.4, 75.8, 8.6, 0.1.

Characterisation data is consistent with values reported in the literature.[15]

3w: Prop-1-yne-1,3-diyldibenzene

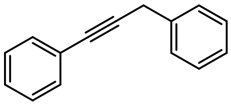

Prepared according to General Procedure A using $Pd(PPh_3)_2Cl_2$ (3.5 mg, 0.005 mmol, 2 mol %), CuI (1.9 mg, 0.01 mmol, 4 mol %), Cyrene (0.5 mL, 0.5 M), Et_3N (38 µL, 0.275 mmol, 1.1 equiv), 2-iodobenzene (27.9 µL, 0.25 mmol, 1 equiv), and 3-phenyl-1-propyne (32.6 µL, 0.263 mmol, 1.05 equiv). After 1 h, the reaction mixture was subjected to the purification method outlined in the General Procedure (silica gel, 0–1% Et_2O in petroleum ether) to afford the title compound as a colourless oil (38.7 mg, 81%).
υ_{max} (liquid film): 3064, 3032, 2924, 1601, 1493 cm^{1}.
^{1}H NMR ($CDCl_3$, 500 MHz): δ 7.46 (dd, *J* = 6.5, 3.0 Hz, 2H), 7.44 (d, *J* = 7.4 Hz, 2H), 7.36 (t, *J* = 7.6 Hz, 2H), 7.32–7.29 (m, 3H), 7.26 (dd, *J* = 8.8, 5.8 Hz, 1H), 3.85 (s, 2H).
^{13}C NMR ($CDCl_3$, 126 MHz): δ 136.8, 131.7, 128.6, 128.3, 127.9, 127.8, 126.7, 123.7, 87.5, 82.7, 25.8.
HRMS: exact mass calculated for [M] ($C_{15}H_{12}$) requires *m/z* 192.0939, found *m/z* 192.0932.
Characterisation data is consistent with literature reported values.[14]

3x: (Cyclohex-1-en-1-ylethynyl)benzene

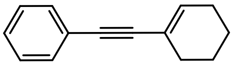

Prepared according to General Procedure A using $Pd(PPh_3)_2Cl_2$ (3.5 mg, 0.005 mmol, 2 mol %), CuI (1.9 mg, 0.01 mmol, 4 mol %), Cyrene (0.5 mL, 0.5 M), Et_3N (38 µL, 0.275 mmol, 1.1 equiv), 2-iodobenzene (27.9 µL, 0.25 mmol, 1 equiv), and 1-ethynylcyclohexene (30.8 µL, 0.263 mmol, 1.05 equiv). After 1 h, the reaction mixture was subjected to the purification method outlined in the General Procedure (silica gel, 0–1% Et_2O in petroleum ether) to afford the title compound as an off white solid (46.3 mg, quant.).
υ_{max} (liquid film): 3062, 2935, 2865, 2204, 1716, 1670 cm^{-1}.
^{1}H NMR ($CDCl_3$, 500 MHz): δ 7.39 (dd, *J* = 7.8, 1.6 Hz, 2H), 7.28–7.23 (m, 3H), 6.20–6.16 (m, 1H), 2.20 (dd, *J* = 8.1, 6.0 Hz, 2H), 2.13–2.09 (m, 2H), 1.68–1.63 (m, 2H), 1.61–1.56 (m, 2H).
^{13}C NMR ($CDCl_3$, 126 MHz): δ 135.2, 131.4, 128.2, 127.7, 123.8, 120.8, 91.3, 86.8, 29.3, 25.8, 22.4, 21.6.
HRMS: exact mass calculated for [M] ($C_{14}H_{15}$) requires *m/z* 182.1095, found *m/z* 182.1102.
Characterisation data is consistent with literature reported values.[15]

3y: 1-Methyl-4-(phenylethynyl)benzene

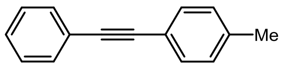

Prepared according to General Procedure A using $Pd(PPh_3)_2Cl_2$ (3.5 mg, 0.005 mmol, 2 mol %), CuI (1.9 mg, 0.01 mmol, 4 mol %), Cyrene (0.5 mL, 0.5 M), Et_3N (38 µL, 0.275 mmol, 1.1 equiv), 2-iodobenzene (27.9 µL, 0.25 mmol, 1 equiv), and *p*-tolylacetylene (33.2 µL, 0.263 mmol, 1.05 equiv). After 1 h, the reaction mixture was subjected to the purification method outlined in the General

Procedure (silica gel, 0–1% Et_2O in petroleum ether) to afford the title compound as an off white solid (46.9 mg, 98%).
υ_{max} (liquid film): 3032, 2921, 2219, 1597, 1511 cm^{-1}.
^1H NMR ($CDCl_3$, 500 MHz): δ 7.53 (dd, J = 7.7, 1.5 Hz, 2H), 7.44 (d, J = 8.0 Hz, 2H), 7.34 (d, J = 7.3 Hz, 3H), 7.16 (d, J = 7.9 Hz, 2H), 2.38 (s, 3H).
^{13}C NMR ($CDCl_3$, 126 MHz): δ 138.4, 131.6, 131.5, 129.1, 128.3, 128.1, 123.5, 120.2, 89.6, 88.7, 21.5.
HRMS: exact mass calculated for [M] ($C_{15}H_{12}$) requires *m/z* 192.0939, found *m/z* 192.0942.
Characterisation data is consistent with literature reported values.[2]

3z: 1-(Phenylethynyl)-2-(trifluoromethyl)benzene

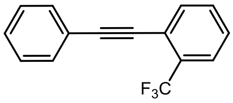

Prepared according to General Procedure A using $Pd(PPh_3)_2Cl_2$ (3.5 mg, 0.005 mmol, 2 mol %), CuI (1.9 mg, 0.01 mmol, 4 mol %), Cyrene (0.5 mL, 0.5 M), Et_3N (38 µL, 0.275 mmol, 1.1 equiv), 2-iodobenzene (27.9 µL, 0.25 mmol, 1 equiv), and 2-ethynyltrifluorotoluene (36.5 µL, 0.263 mmol, 1.05 equiv). After 1 h, the reaction mixture was subjected to the purification method outlined in the General Procedure (silica gel, 0–1% Et_2O in petroleum ether) to afford the title compound as a colourless oil (53 mg, 86%).
υ_{max} (liquid film): 3066, 2224, 1312 cm^{-1}.
^1H NMR ($CDCl_3$, 500 MHz): δ 7.69 (t, J = 8.3 Hz, 2H), 7.57 (dd, J = 6.5, 3.0 Hz, 2H), 7.53 (t, J = 7.5 Hz, 1H), 7.43 (t, J = 7.7 Hz, 1H), 7.40–7.35 (m, 3H).
^{13}C NMR ($CDCl_3$, 126 MHz): δ 133.7, 131.7, 131.4, 128.8, 128.4, 127.9, 125.9 (q, $^3J_{CF}$ = 5.2 Hz), 123.6 (q, $^1J_{CF}$ = 273.5 Hz), 122.8, 121.6, 94.9, 85.4. Carbon bearing trifluoromethyl group not observed.
^{19}F NMR ($CDCl_3$, 471 MHz): δ -62.35.
HRMS: exact mass calculated for [M] ($C_{15}H_9F_3$) requires *m/z* 246.0656, found *m/z* 246.0654.
Characterisation data is consistent with literature reported values.[16]

3aa: 2-(Phenylethynyl)pyridine

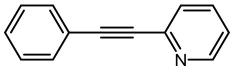

Prepared according to General Procedure A using $Pd(PPh_3)_2Cl_2$ (3.5 mg, 0.005 mmol, 2 mol %), CuI (1.9 mg, 0.01 mmol, 4 mol %), Cyrene (0.5 mL, 0.5 M), Et_3N (38 µL, 0.275 mmol, 1.1 equiv), 2-iodobenzene (27.9 µL, 0.25 mmol, 1 equiv), and 2-ethynylpyridine (26.5 µL, 0.263 mmol, 1.05 equiv). After 1 h, the reaction mixture was subjected to the purification method outlined in the General Procedure (silica gel, 0–15% EtOAc in petroleum ether) to afford the title compound as a yellow oil (43.3 mg, 97%).
υ_{max} (liquid film): 3053, 2224, 1582, 1493, 1463 cm^{-1}.
^1H NMR ($CDCl_3$, 500 MHz): δ 8.62 (d, J = 4.4 Hz, 1H), 7.67 (td, J = 7.7, 1.7 Hz, 1H), 7.60 (dd, J = 6.5, 3.1 Hz, 2H), 7.52 (d, J = 7.8 Hz, 1H), 7.38–7.35 (m, 3H), 7.26 – 7.22 (m, 1H).
^{13}C NMR ($CDCl_3$, 126 MHz): δ 150.1, 143.5, 136.2, 132.1, 128.9, 128.4, 127.2, 122.8, 122.3, 89.2, 88.6.
HRMS: exact mass calculated for $[M+Na]^+$ ($C_{21}H_{18}BF_3N_2O_6SNa$) requires *m/z* 179.0735, found *m/z* 179.0731.

Characterisation data is consistent with literature reported values.[2]

3ab: 2-(Phenylethynyl)thiophene

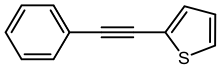

Prepared according to General Procedure A using $Pd(PPh_3)_2Cl_2$ (3.5 mg, 0.005 mmol, 2 mol %), CuI (1.9 mg, 0.01 mmol, 4 mol %), Cyrene (0.5 mL, 0.5 M), Et_3N (38 µL, 0.275 mmol, 1.1 equiv), 2-iodobenzene (27.9 µL, 0.25 mmol, 1 equiv), and 2-ethynylthiophene (24.9 µL, 0.263 mmol, 1.05 equiv). After 1 h, the reaction mixture was subjected to the purification method outlined in the General Procedure (silica gel, 0–1% Et_2O in petroleum ether) to afford the title compound as an off white solid (42.4 mg, 92%).
υ_{max} (liquid film): 3088, 2204 cm^{-1}.
^{1}H NMR ($CDCl_3$, 500 MHz): δ 7.54–7.50 (m, 2H), 7.35 (m, 3H), 7.31–7.28 (m, 2H), 7.02 (t, *J* = 4.4 Hz, 1H).
^{13}C NMR ($CDCl_3$, 126 MHz): δ 131.9, 131.4, 128.4, 128.4, 127.3, 127.1, 123.4, 122.9, 93.0, 82.6.
HRMS: exact mass calculated for [M] ($C_{12}H_8S$) requires *m/z* 184.0347, found *m/z* 184.0349.
Characterisation data is consistent with literature reported values.[2]

5.5. Products from Scheme 2c

3ac: 2-Acetyl phenylethynylboronic acid, MIDA ester

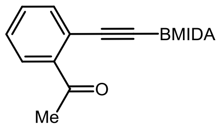

Prepared according to General Procedure A using $Pd(PPh_3)_2Cl_2$ (3.5 mg, 0.005 mmol, 2 mol %), CuI (1.9 mg, 0.01 mmol, 4 mol %), Cyrene (0.5 mL, 0.5 M), Et_3N (38 µL, 0.275 mmol, 1.1 equiv), 2-iodoacetophenone (35.8 µL, 0.25 mmol, 1 equiv), and ethynyl boronic acid, MIDA ester (47.5 mg, 0.263 mmol, 1.05 equiv). After 1 h, the reaction mixture was subjected to the purification method outlined in the General Procedure (silica gel, 0–100% EtOAc in petroleum ether) to afford the title compound as an off white solid (66.5 mg, 89%).
υ_{max} (solid): 2960, 2193, 1770, 1684 cm^{-1}.
^{1}H NMR (DMSO-d_6, 500 MHz): δ 7.79 (dd, *J* = 7.7, 0.9 Hz, 1H), 7.63 (dd, *J* = 7.6, 0.9 Hz, 1H), 7.57 (td, *J* = 7.5, 1.3 Hz, 1H), 7.52 (td, *J* = 7.6, 1.3 Hz, 1H), 4.34 (d, *J* = 17.1 Hz, 2H), 4.13 (d, *J* = 17.1 Hz, 2H), 3.11 (s, 3H), 2.63 (s, 3H).
^{13}C NMR (DMSO-d_6, 126 MHz): δ 200.1, 169.1, 141.2, 134.6, 131.9, 129.4, 129.2, 120.7, 98.6, 61.9, 48.4, 29.9. Carbon bearing boron not observed.
^{11}B NMR (DMSO-d_6, 160 MHz): δ 6.23.
HRMS: exact mass calculated for $[M+NH_4]^+$ ($C_{15}H_{18}BN_2O_5$) requires *m/z* 317.1305, found *m/z* 317.1303.

3ad: 2-Methyl-phenylethynylboronic acid, MIDA ester

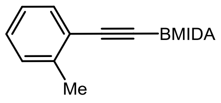

Prepared according to General Procedure A using $Pd(PPh_3)_2Cl_2$ (3.5 mg, 0.005 mmol, 2 mol %), CuI (1.9 mg, 0.01 mmol, 4 mol %), Cyrene (0.5 mL, 0.5 M), Et_3N (38 µL, 0.275 mmol, 1.1 equiv), 2-iodotoluene (31.8 µL, 0.25 mmol, 1 equiv), and ethynyl boronic acid, MIDA ester (47.5 mg, 0.263 mmol, 1.05 equiv). After 1 h, the reaction mixture was subjected to the purification method outlined in the General Procedure (silica gel, 0–60% EtOAc in petroleum ether) to afford the title compound as an off white solid (62.9 mg, 93%).

Prepared according to General Procedure A using $Pd(PPh_3)_2Cl_2$ (3.5 mg, 0.005 mmol, 2 mol %), CuI (1.9 mg, 0.01 mmol, 4 mol %), DMF (0.5 mL, 0.5 M), Et_3N (38 µL, 0.275 mmol, 1.1 equiv), 2-iodotoluene (31.8 µL, 0.25 mmol, 1 equiv), and ethynyl boronic acid, MIDA ester (47.5 mg, 0.263 mmol, 1.05 equiv). After 1 h, the reaction mixture was subjected to the purification method outlined in the General Procedure (silica gel, 0–60% EtOAc in petroleum ether) to afford the title compound as an off white solid (62.3 mg, 92%).
υ_{max} (solid): 3019, 2191, 1770, 1290, 1247 cm^{-1}.
^1H NMR (DMSO-d_6, 500 MHz): δ 7.45 (d, J = 7.4 Hz, 1H), 7.31–7.27 (m, 2H), 7.22–7.18 (m, 1H), 4.33 (d, J = 17.1 Hz, 2H), 4.15 (d, J = 17.1 Hz, 2H), 3.09 (s, 3H), 2.40 (s, 3H).
^{13}C NMR (DMSO-d_6, 126 MHz): δ 169.2, 140.3, 132.4, 129.9, 129.3, 126.3, 122.7, 98.7, 61.9, 48.4, 20.8. Carbon bearing boron not observed.
^{11}B NMR (DMSO-d_6, 160 MHz): δ 6.37.
HRMS: exact mass calculated for $[M+NH_4]^+$ ($C_{14}H_{18}BN_2O_4$) requires m/z 289.1355, found m/z 289.1354.
Characterisation data is consistent with literature reported values.[17]

3ae: 2-Trifluoromethoxy-phenylethynylboronic acid, MIDA ester

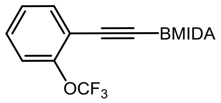

Prepared according to General Procedure A using $Pd(PPh_3)_2Cl_2$ (3.5 mg, 0.005 mmol, 2 mol %), CuI (1.9 mg, 0.01 mmol, 4 mol %), Cyrene (0.5 mL, 0.5 M), Et_3N (38 µL, 0.275 mmol, 1.1 equiv), 2-(trifluoromethoxy)iodobenzene (38.8 µL, 0.25 mmol, 1 equiv), and ethynyl boronic acid, MIDA ester (47.5 mg, 0.263 mmol, 1.05 equiv). After 1 h, the reaction mixture was subjected to the purification method outlined in the General Procedure (silica gel, 0–60% EtOAc in petroleum ether) to afford the title compound as an off white solid (71.8 mg, 85%).
υ_{max} (solid): 3016, 2922, 2965, 2198, 1772, 1217, 1024 cm^{-1}.
^1H NMR (DMSO-d_6, 500 MHz): δ 7.69 (d, J = 7.5 Hz, 1H), 7.55 (t, J = 7.4 Hz, 1H), 7.45 (dd, J = 16.7, 8.6 Hz, 2H), 4.35 (d, J = 17.2 Hz, 2H), 4.15 (d, J = 17.2 Hz, 2H), 3.09 (s, 3H).
^{13}C NMR (DMSO-d_6, 126 MHz): δ 169.0, 148.9, 134.5, 131.3, 128.3, 121.9, 120.6 (q, $^1J_{CF}$ = 257.4 Hz), 117.3, 93.5, 62.1, 48.3. Carbon bearing boron not observed.
^{11}B NMR (DMSO-d_6, 160 MHz): δ 6.29.
^{19}F NMR (DMSO-d_6, 471 MHz): δ -56.54.
HRMS: exact mass calculated for $[M+H]^+$ ($C_{14}H_{12}BF_3NO_5$) requires m/z 342.0763, found m/z 342.0767.

3af: Triisopropyl((2-(trifluoromethoxy)phenyl)ethynyl)silane

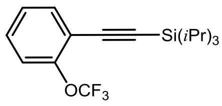

Prepared according to General Procedure A using $Pd(PPh_3)_2Cl_2$ (3.5 mg, 0.005 mmol, 2 mol %), CuI (1.9 mg, 0.01 mmol, 4 mol %), Cyrene (0.5 mL, 0.5 M), Et_3N (38 µL, 0.275 mmol, 1.1 equiv), 2-(trifluoromethoxy)iodobenzene (38.8 µL, 0.25 mmol, 1 equiv), and (triisopropylsilyl)acetylene (58.9 µL, 0.263 mmol, 1.05 equiv). After 1 h, the reaction mixture was subjected to the purification method outlined in the General Procedure (silica gel, 0–1% Et_2O in petroleum ether) to afford the title compound as a colouless oil (58.3 mg, 68%).

υ_{max} (liquid film): 2947, 2868, 2167, 1491, 1258, 1219, 1169 cm^{-1}.

^{1}H NMR ($CDCl_3$, 500 MHz): δ 7.47 (dd, J = 7.6, 1.3 Hz, 1H), 7.28–7.24 (m, 1H), 7.19–7.14 (m, 2H), 1.06 (s, 21H).

^{13}C NMR ($CDCl_3$, 126 MHz): δ 149.8, 134.1, 129.4, 126.6, 121.2, 120.6 (q, $^{1}J_{CF}$ = 258.1 Hz), 118.3, 100.4, 97.1, 18.5, 11.2.

^{19}F NMR (471 MHz, $CDCl_3$): δ -57.50.

HRMS: exact mass calculated for [M] ($C_{18}H_{25}F_3SiO$) requires m/z 342.1627, found m/z 342.1626.

3ag: 2-((Triisopropylsilyl)ethynyl)aniline

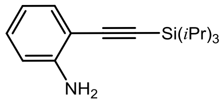

Prepared according to General Procedure A using $Pd(PPh_3)_2Cl_2$ (3.5 mg, 0.005 mmol, 2 mol %), CuI (1.9 mg, 0.01 mmol, 4 mol %), Cyrene (0.5 mL, 0.5 M), Et_3N (38 µL, 0.275 mmol, 1.1 equiv), 2-iodoaniline (54.8 mg, 0.25 mmol, 1 equiv), and (triisopropylsilyl)acetylene (58.9 µL, 0.263 mmol, 1.05 equiv). After 1 h, the reaction mixture was subjected to the purification method outlined in the General Procedure (silica gel, 0–10% Et_2O in petroleum ether) to afford the title compound as a yellow oil (45.3 mg, 66%).

υ_{max} (liquid film): 3487, 3388, 2945, 2867, 2146, 1616, 1318 cm^{-1}.

^{1}H NMR ($CDCl_3$, 500 MHz): δ 7.31 (dd, J = 7.7, 1.1 Hz, 1H), 7.14–7.09 (m, 1H), 6.71–6.64 (m, 2H), 4.25 (s, 2H), 1.14 (s, 21H).

^{13}C NMR ($CDCl_3$, 126 MHz): δ 148.3, 132.4, 129.7, 117.7, 114.1, 108.3, 103.7, 95.9, 18.7, 11.3.

HRMS: exact mass calculated for $[M+H]^+$ ($C_{17}H_{28}NSi$) requires m/z 274.1986, found m/z 274.1986.

Characterisation data is consistent with literature reported values.[18]

3ah: ((2-Chlorophenyl)ethynyl)triisopropylsilane

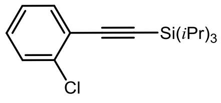

Prepared according to General Procedure A using $Pd(PPh_3)_2Cl_2$ (3.5 mg, 0.005 mmol, 2 mol %), CuI (1.9 mg, 0.01 mmol, 4 mol %), Cyrene (0.5 mL, 0.5 M), Et_3N (38 µL, 0.275 mmol, 1.1 equiv), 2-chloro-iodobenzene (30.5 µL, 0.25 mmol, 1 equiv), and (triisopropylsilyl)acetylene (58.9 µL, 0.263 mmol, 1.05 equiv). After 1 h, the reaction mixture was subjected to purification by reverse phase chromatography (C18 cartridge, 20–100% MeCN in water) to afford the title compound as a yellow oil (47.7 mg, 65%).

υ_{max} (liquid film): 2945, 2867, 2163, 1472, 1225 cm^{-1}.
^{1}H NMR ($CDCl_3$, 500 MHz): δ 7.51 (dd, *J* = 7.5, 1.6 Hz, 1H), 7.38 (dd, *J* = 7.9, 0.9 Hz, 1H), 7.23 (td, *J* = 7.7, 1.8 Hz, 1H), 7.19 (td, *J* = 7.5, 1.2 Hz, 1H), 1.15 (s, 21H).
^{13}C NMR ($CDCl_3$, 126 MHz): δ 136.5, 133.9, 129.4, 126.4, 123.6, 103.3, 96.9, 18.8, 11.5.
HRMS: exact mass calculated for [M] ($C_{17}H_{25}ClSi$) requires *m/z* 292.1414, found *m/z* 292.1431.

5.6. Cyrene homo-aldol adducts, 4a and 4b

To a stirred solution of Cyrene (256 mg, 2.0 mmol, 1 equiv) was added DBU (30 mg, 0.2 mmol, 0.1 equiv) and the mixture heated to 100 °C for 10 minutes. The resulting mixture was cooled to 20 °C giving a viscous brown oil and then kept at 20 °C for 72 hours over which time the mixture began to crystallise. The mixture was then purified by flash chromatography (30% EtOAc/hexanes to EtOAc) to give **4a** as a colourless oil (40 mg, 16%).

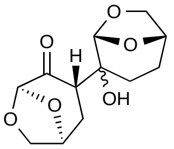

4a

υ_{max} (neat): 3470, 2962, 2895, 1731 cm^{-1}.

^{1}H NMR ($CDCl_3$, 500 MHz): δ 5.71 (s, 1H), 5.04 (s, 1H), 4.73-4.70 (m, 1H), 4.44-4.41 (m, 1H), 4.01 (br d, *J* = 7.4 Hz, 1H), 3.91 (ddd, *J* = 7.4, 4.9, 1.6 Hz, 1H), 3.83 (d, *J* = 7.1 Hz, 1H), 3.76 (ddd, *J* = 7.1, 5.1, 0.9 Hz, 1H), 3.35 (dd, *J* = 12.0, 7.4 Hz, 1H), 2.72 (s, 1H), 2.27 (dddd, *J* = 13.3, 12.0, 3.7, 1.8 Hz, 1H), 1.96 (ddd, *J* = 13.3, 7.4, 1.6 Hz, 1H), 1.64-1.59 (m, 3H), 1.50-1.46 (m, 1H).

^{13}C NMR ($CDCl_3$, 126 MHz): δ 203.7, 102.9, 101.6, 73.6, 73.2, 72.9, 68.4, 67.9, 42.7, 32.6, 26.7, 25.1.

ESI-MS: *m/z* 257 (50, [M+H]+), 279 (100, [M+Na]).

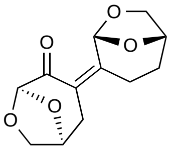

4b

To an oven-dried 5 mL microwave vessel was added K_3PO_4 (637 mg, 3 mmol, 3 equiv). The vessel was then capped and purged with N_2 before addition of THF (4 mL, 0.25 M), and Cyrene (123 μL, 1 mmol, 1 equiv). The reaction mixture was heated to 70 °C and maintained at this temperature with stirring for 8 h before the vessel was vented, and decapped. The solution was then diluted with EtOAc (20 mL), and washed with water (2 × 20 mL) and brine (2 × 20 mL). The organics were then passed through a hydrophobic frit and concentrated under reduced pressure to give an off white solid, which was purified by flash chromatography (silica gel, 0–50% EtOAc in petroleum ether) to afford the title compound as a white solid (105 mg, 88%).

υ_{max} (solid): 2898, 1703, 1621, 1098 cm^{-1}.
^{1}H NMR ($CDCl_3$, 500 MHz): δ 6.76 (s, 1H), 5.18 (s, 1H), 4.79 (t, *J* = 5.1 Hz, 1H), 4.60 (t, *J* = 4.0 Hz, 1H), 3.94 – 3.83 (m, 4H), 2.78 (dd, *J* = 16.3, 2.6 Hz, 1H), 2.56 (d, *J* = 16.3 Hz, 1H), 2.41-2.24 (m, 2H), 2.14-2.07 (m, 1H), 1.75 (dd, *J* = 13.5, 6.5 Hz, 1H).

^{13}C NMR ($CDCl_3$, 126 MHz): δ 190.7, 151.0, 123.4, 101.5, 97.2, 72.6, 72.5, 68.7, 67.8, 34.1, 28.8, 20.4.
HRMS: exact mass calculated for [M] ($C_{12}H_{14}$) requires *m/z* 238.0841, found *m/z* 238.0839.

5.7. Products from Scheme 3

7a: 2-Phenyl-1-tosyl-1*H*-indole

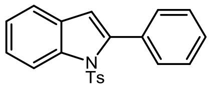

Prepared according to General Procedure B using $Pd(PPh_3)_2Cl_2$ (3.5 mg, 0.005 mmol, 2 mol %), CuI (1.9 mg, 0.01 mmol, 4 mol %), Cyrene (0.5 mL, 0.5 M), Et_3N (104 µL, 0.75 mmol, 3 equiv), *N*-(2-iodophenyl)-4-methylbenzenesulfonamide (93 mg, 0.25 mmol, 1 equiv), and phenylacetylene (28.8 µL, 0.263 mmol, 1.05 equiv). After 7 h, the reaction mixture was subjected to the purification method outlined in the General Procedure (silica gel, 0–15% EtOAc in petroleum ether) to afford the title compound as a white solid (78.4 mg, 90%).

Prepared according to General Procedure B using $Pd(PPh_3)_2Cl_2$ (3.5 mg, 0.005 mmol, 2 mol %), CuI (1.9 mg, 0.01 mmol, 4 mol %), DMF (0.5 mL, 0.5 M), Et_3N (104 µL, 0.75 mmol, 3 equiv), *N*-(2-iodophenyl)-4-methylbenzenesulfonamide (93 mg, 0.25 mmol, 1 equiv), and phenylacetylene (28.8 µL, 0.263 mmol, 1.05 equiv). After 7 h, the reaction mixture was subjected to the purification method outlined in the General Procedure (silica gel, 0–15% EtOAc in petroleum ether) to afford the title compound as a white solid (78.6 mg, 91%).
υ_{max} (solid): 3073, 1368, 1169 cm^{-1}.
^1H NMR ($CDCl_3$, 500 MHz): δ 8.33 (d, *J* = 8.4 Hz, 1H), 7.54–7.50 (m, 2H), 7.45 (t, *J* = 8.2 Hz, 4H), 7.38 (t, *J* = 7.4 Hz, 1H), 7.31–7.28 (m, 3H), 7.06 (d, *J* = 8.1 Hz, 2H), 6.56 (s, 1H), 2.31 (s, 3H).
^{13}C NMR ($CDCl_3$, 126 MHz): δ 144.5, 142.2, 138.3, 134.7, 132.4, 130.7, 130.4, 129.2, 128.7, 127.5, 126.8, 124.8, 124.3, 120.7, 116.7, 113.4, 21.5.
HRMS: exact mass calculated for $[M+H]^+$ ($C_{21}H_{18}NO_6S$) requires *m/z* 348.1058, found *m/z* 348.1061.
Characterization data is consistent with literature reported values.[3]

7b: 5-Nitro-2-phenyl-1-tosyl-1*H*-pyrrolo[2,3-b]pyridine

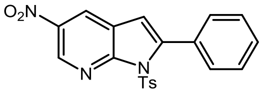

Prepared according to General Procedure B using $Pd(PPh_3)_2Cl_2$ (3.5 mg, 0.005 mmol, 2 mol %), CuI (1.9 mg, 0.01 mmol, 4 mol %), Cyrene (0.5 mL, 0.5 M), Et_3N (104 µL, 0.75 mmol, 3 equiv), *N*-(3-iodo-5-nitropyridin-2-yl)-4-methylbenzenesulfonamide (104 mg, 0.25 mmol, 1 equiv), and phenylacetylene (28.8 µL, 0.263 mmol, 1.05 equiv). After 7 h, the reaction mixture was subjected to the purification method outlined in the General Procedure (silica gel, 0-30% EtOAc in petroleum ether) to afford the title compound as an off white solid (71.4 mg, 73%).
υ_{max} (solid): 3070, 2935, 1593, 1517, 1394, 1346, 1184 cm^{-1}.
^1H NMR ($CDCl_3$, 500 MHz): δ 9.32 (d, *J* = 2.4 Hz, 1H), 8.61 (d, *J* = 2.4 Hz, 1H), 7.85 (d, *J* = 8.3 Hz, 2H), 7.55 – 7.48 (m, 5H), 7.24 (d, *J* = 8.1 Hz, 2H), 6.63 (s, 1H), 2.37 (s, 3H).
^{13}C NMR – ($CDCl_3$, 126 MHz): δ 151.3, 145.8, 145.8, 141.4, 140.3, 135.3, 131.5, 129.9, 129.6, 129.6, 128.2, 127.9, 124.3, 121.4, 108.3, 21.7.
HRMS: exact mass calculated for $[M+H]^+$ ($C_{20}H_{16}N_3O_4S$) requires *m/z* 394.0862, found *m/z* 394.0869.

7c: 2-Phenylbenzofuran

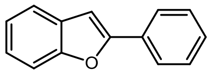

Prepared according to General Procedure B using $Pd(PPh_3)_2Cl_2$ (3.5 mg, 0.005 mmol, 2 mol %), CuI (1.9 mg, 0.01 mmol, 4 mol %), Cyrene (0.5 mL, 0.5 M), Et_3N (104 µL, 0.75 mmol, 3 equiv), 2-iodophenol (55 mg, 0.25 mmol, 1 equiv), and phenylacetylene (28.8 µL, 0.263 mmol, 1.05 equiv). After 7 h, the reaction mixture was subjected to the purification method outlined in the General Procedure (silica gel, 0–1% EtOAc in petroleum ether) to afford the title compound as a white solid (43.3 mg, 89%).

υ_{max} (solid): 3038, 2924, 2855 cm^{-1}.

^{1}H NMR ($CDCl_3$, 500 MHz): δ 7.88 (d, *J* = 7.4 Hz, 2H), 7.59 (d, *J* = 7.5 Hz, 1H), 7.53 (d, *J* = 8.1 Hz, 1H), 7.46 (t, *J* = 7.7 Hz, 2H), 7.36 (t, *J* = 7.4 Hz, 1H), 7.29 (t, *J* = 7.7 Hz, 1H), 7.23 (t, *J* = 7.4 Hz, 1H), 7.03 (s, 1H).

^{13}C NMR ($CDCl_3$, 126 MHz): δ 155.9, 154.9, 130.5, 129.2, 128.8, 128.6, 124.9, 124.3, 122.9, 120.9, 111.2, 101.3

HRMS: exact mass calculated for [M] ($C_{14}H_{10}O$) requires *m/z* 194.0732, found *m/z* 194.0737.

Characterization data is consistent with literature reported values.[19]

7d: (1-Tosyl-1*H*-indol-2-yl)boronic acid, MIDA ester

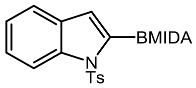

Prepared according to General Procedure B using $Pd(PPh_3)_2Cl_2$ (3.5 mg, 0.005 mmol, 2 mol %), CuI (1.9 mg, 0.01 mmol, 4 mol %), Cyrene (0.5 mL, 0.5 M), Et_3N (104 µL, 0.75 mmol, 3 equiv), *N*-(2-iodophenyl)-4-methylbenzenesulfonamide (93 mg, 0.25 mmol, 1 equiv), and ethynyl boronic acid, MIDA ester (47.5 mg, 0.263 mmol, 1.05 equiv). After 7 h, the reaction mixture was subjected to the purification method outlined in the General Procedure (silica gel, 0–80% EtOAc in petroleum ether) to afford the title compound as a white solid (87.4 mg, 82%).

υ_{max} (solid): 2928, 1763, 1450, 1176, 1038 cm^{-1}.

^{1}H NMR (DMSO-d_6, 500 MHz): δ 8.12 (d, *J* = 8.4 Hz, 1H), 7.91 (d, *J* = 8.2 Hz, 2H), 7.63 (d, *J* = 7.7 Hz, 1H), 7.37 (dd, *J* = 13.1, 8.0 Hz, 3H), 7.25 (t, *J* = 7.4 Hz, 1H), 7.06 (s, 1H), 4.47 (d, *J* = 17.5 Hz, 2H), 4.23 (d, *J* = 17.4 Hz, 2H), 2.96 (s, 3H), 2.32 (s, 3H).

^{13}C NMR (DMSO-d_6, 126 MHz): δ 169.6, 145.72, 138.9, 135.5, 130.4, 130.1, 127.08, 125.7, 123.9, 122.2, 122.0, 114.7, 64.8, 49.9, 21.5. Carbon bearing boron not observed.

^{11}B NMR (DMSO-d_6, 160 MHz): δ 10.28.

HRMS: exact mass calculated for $[M+H]^+$ ($C_{20}H_{20}BN_2O_6S$) requires *m/z* 427.1139, found *m/z* 427.1139.

Characterization data is consistent with literature reported values.[4]

7e: (5-Fluoro-1-tosyl-1*H*-indol-2-yl)boronic acid, MIDA ester

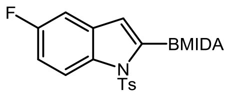

Prepared according to General Procedure B using $Pd(PPh_3)_2Cl_2$ (3.5 mg, 0.005 mmol, 2 mol %), CuI (1.9 mg, 0.01 mmol, 4 mol %), Cyrene (0.5 mL, 0.5 M), Et_3N (104 µL, 0.75 mmol, 3 equiv), *N*-(4-fluoro-2-iodophenyl)-4-methylbenzenesulfonamide (98 mg, 0.25 mmol, 1 equiv), and ethynyl boronic

acid, MIDA ester (47.5 mg, 0.263 mmol, 1.05 equiv). After 7 h, the reaction mixture was subjected to the purification method outlined in the General Procedure (silica gel, 0–80% EtOAc in petroleum ether) to afford the title compound as a white solid (98 mg, 88%).

υ_{max} (solid): 2930, 1750, 1305, 1174, 1040 cm^{-1}.

^1H NMR (DMSO-d_6, 500 MHz): δ 8.13 (dd, J = 9.2, 4.3 Hz, 1H), 7.91 (d, J = 8.4 Hz, 2H), 7.47 (dd, J = 8.8, 2.6 Hz, 1H), 7.40 (d, J = 8.2 Hz, 2H), 7.22 (td, J = 9.2, 2.6 Hz, 1H), 7.06 (s, 1H), 4.48 (d, J = 17.5 Hz, 2H), 4.24 (d, J = 17.5 Hz, 2H), 2.96 (s, 3H), 2.33 (s, 3H).

^{13}C NMR (DMSO-d_6, 126 MHz): δ 169.6, 159.3 (d, $^1J_{CF}$ = 238.2 Hz), 145.9, 135.4, 135.3, 131.2 (d, $^3J_{CF}$ = 10.4 Hz), 130.5, 127.1, 121.9, 116.1 (d, $^3J_{CF}$ = 9.4 Hz), 113.5 (d, $^2J_{CF}$ = 25.5 Hz), 107.1 (d, $^2J_{CF}$ = 23.5 Hz), 64.8, 49.9, 21.5. Carbon bearing boron not observed.

^{11}B NMR (DMSO-d_6, 160 MHz): δ 10.09.

^{19}F NMR (DMSO-d_6, 471 MHz): δ -120.04.

HRMS: exact mass calculated for [M] ($C_{21}H_{18}BF_3N_2O_6SNa$) requires m/z 444.2966, found m/z 444.0951.

Characterization data is consistent with literature reported values.[4]

7f: (6-Chloro-1-tosyl-1*H*-indol-2-yl)boronic acid, MIDA ester

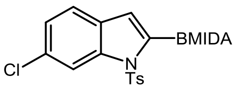

Prepared according to General Procedure B using $Pd(PPh_3)_2Cl_2$ (3.5 mg, 0.005 mmol, 2 mol %), CuI (1.9 mg, 0.01 mmol, 4 mol %), Cyrene (0.5 mL, 0.5 M), Et_3N (104 µL, 0.75 mmol, 3 equiv), *N*-(4-chloro-2-iodophenyl)-4-methylbenzenesulfonamide (102 mg, 0.25 mmol, 1 equiv), and ethynyl boronic acid, MIDA ester (47.5 mg, 0.263 mmol, 1.05 equiv). After 7 h, the reaction mixture was subjected to the purification method outlined in the General Procedure (silica gel, 0–80% EtOAc in petroleum ether) to afford the title compound as a white solid (116 mg, quant.).

υ_{max} (solid): 2922, 1763, 1455, 1267, 1173, 1038 cm^{-1}.

^1H NMR (DMSO-d_6, 500 MHz): δ 8.11 (s, 1H), 7.90 (d, J = 8.4 Hz, 2H), 7.69 (d, J = 8.4 Hz, 1H), 7.42 (d, J = 8.2 Hz, 2H), 7.34 (dd, J = 8.4, 1.7 Hz, 1H), 7.09 (s, 1H), 4.48 (d, J = 17.5 Hz, 2H), 4.23 (d, J = 17.4 Hz, 2H), 2.94 (s, 3H), 2.34 (s, 3H).

^{13}C NMR (DMSO-d_6, 126 MHz): δ 169.6, 146.1, 139.3, 135.2, 130.6, 130.4, 128.9, 127.0, 124.4, 123.4, 121.9, 114.4, 64.7, 49.9, 21.5. Carbon bearing boron not observed.

^{11}B NMR (DMSO-d_6, 160 MHz): δ 10.21.

HRMS: exact mass calculated for $[M+Na]^+$ ($C_{20}H_{18}ClN_2O_6SB$) requires m/z 460.0671, found m/z 460.0658.

Characterization data is consistent with literature reported values.[4]

6. Crystallographic Data for Compound 4b

Single crystal diffraction measurements were made with an Oxford Diffraction Gemini S instrument. Refinement was to convergence against F^2 and used all unique reflections. Programs used were from the SHELX suite.[20] Non-hydrogen atoms were refined anisotropically whereas hydrogen atoms were placed in idealized positions and refined in riding modes. Selected crystallographic and refinement parameters are given in Table 1. CCDC reference number CCDC 1485168 contains the supplementary crystallographic data for this paper. This data can be obtained free of charge from The Cambridge Crystallographic Data Centre via www.ccdc.cam.ac.uk/data_request/cif.

Table S1 Selected crystallographic data and refinement parameters for compound **4b**.

**Compound**	**4**
Formula	$C_{12}H_{14}O_5$
M_r (g mol^{-1})	238.23
Crystal system	monoclinic
Space group	$P2_1$
Temperature (K)	123(2)
a (Å)	6.4668(2)
b (Å)	9.8239(3)
c (Å)	8.5963(2)
β (°)	96.341(3)
V/Å^3	542.78(3)
Z	2
Wavelength (Å)	0.71073
Measured reflections	9884
Unique reflections	3457
R_{int}	0.03024
Observed rflns [$I > 2\sigma(I)$]	3286
μ (mm^{-1})	0.114
No. of parameters	155
2ϑmax (°)	63.8
R [on F, obs rflns only]	0.0329
wR [on F^2, all data]	0.0852
GoF	1.043
Largest diff. peak/hole/e Å^{-3}	0.242/–0.191

7. References

1. Armarego, W.; Chai, C. *Purification of laboratory chemicals*. Elsevier: Amsterdam, 2009.
2. Raja, G. C.; Irudayanathan, F. M.; Kim, H.-S.; Kim, J.; Lee, S. *J. Org. Chem.*, **2016**, *81*, 5244-5249.
3. Jang, Y. H., Youn, S. W. *Org. Lett.*, **2014**, *16*, 3720-3723.
4. Seath, C. P.; Wilson, K. L.; Campbell, A.; Mowat, J. M.; Watson, A. J. B. *Chem. Commun.*, **2016**, *52*, 8703-8706.

5. Kakusawa, N.; Yamaguchi, K.; Kurita, J. *J. Organomet. Chem.*, **2005**, *690*, 2956-2966.
6. Dickson, D. P.; Toh, C.; Lunda, M.; Yermolina, M. V.; Wardrop, D. J.; Landrie, C. L. *J. Org. Chem.*, **2009**, *74*, 9535-9538.
7. Gallop, C. W. D.; Chen, M.-T.; Navarro, O. *Org. Lett.*, **2014**, *16*, 3724-3727.
8. Goodman, A. J.; Stanforth, S. P.; Tarbit, B. *Tetrahedron*, **1999**, *55*, 15067-15070.
9. Zarwell, S.; Dietrich, S.; Schulz, C.; Dietrich, P.; Michalik, F.; Rück-Braun, K. *Eur. J. Org. Chem.*, **2009**, 2088-2095.
10. Hyodo, K.; Suetsugu, M.; Nishihara, Y. *Org. Lett.*, **2014**, *16*, 440-443.
11. Stefani, H. A.; Cella, R.; Dörr, F. A.; de Pereira, C. M. P.; Gomes, F. P.; Zeni, G. *Tetrahedron Lett.*, **2005**, *46*, 2001-2003.
12. Qian, D.; Zhang, J. *Beilstein J. Org. Chem.*, **2011**, *7*, 808-812.
13. Chahdoura, F.; Pradel, C.; Gómez, M. *Adv. Synth. Catal.*, **2013**, *355*, 3648-3660.
14. Li, X.; Liu, X.; Chen, H.; Wu, W.; Qi, C.; Jiang, H. *Angew. Chem. Int. Ed.*, **2014**, *53*, 14485-14489.
15. Cai, R.; Lu, M.; Aguilera, E. Y.; Xi, Y.; Akhmedov, N. G.; Peterson, J.; Chen, H.; Shi, X. *Angew. Chem. Int. Ed.*, **2015**, *54*, 8772-8776.
16. Strappaveccia, G.; Luciani, L.; Bartollini, E.; Marrocchi, A.; Pizzo, F.; Vaccaro, L. *Green Chem.*, **2015**, *17*, 1071-1076.
17. Struble, J. R.; Lee, S. J.; Burke, M. D. *Tetrahedron*, **2010**, *66*, 4710-4718.
18. Kanno, H.; Nakamura, K.; Noguchi, K.; Shibata, Y.; Tanaka, K. *Org. Lett.*, **2016**, *18*, 1654-1657.
19. Rubio-Marqués, P.; Rivero-Crespo, M. A.; Leyva-Pérez, A.; Corma, A. *J. Am. Chem. Soc.*, **2015**, *137*, 11832-11837.
20. Sheldrick, G. M. *Acta Cryst.*, **2008**, *64*, 112-122.

8. NMR spectra for intermediates and products

^{1}H NMR of S1

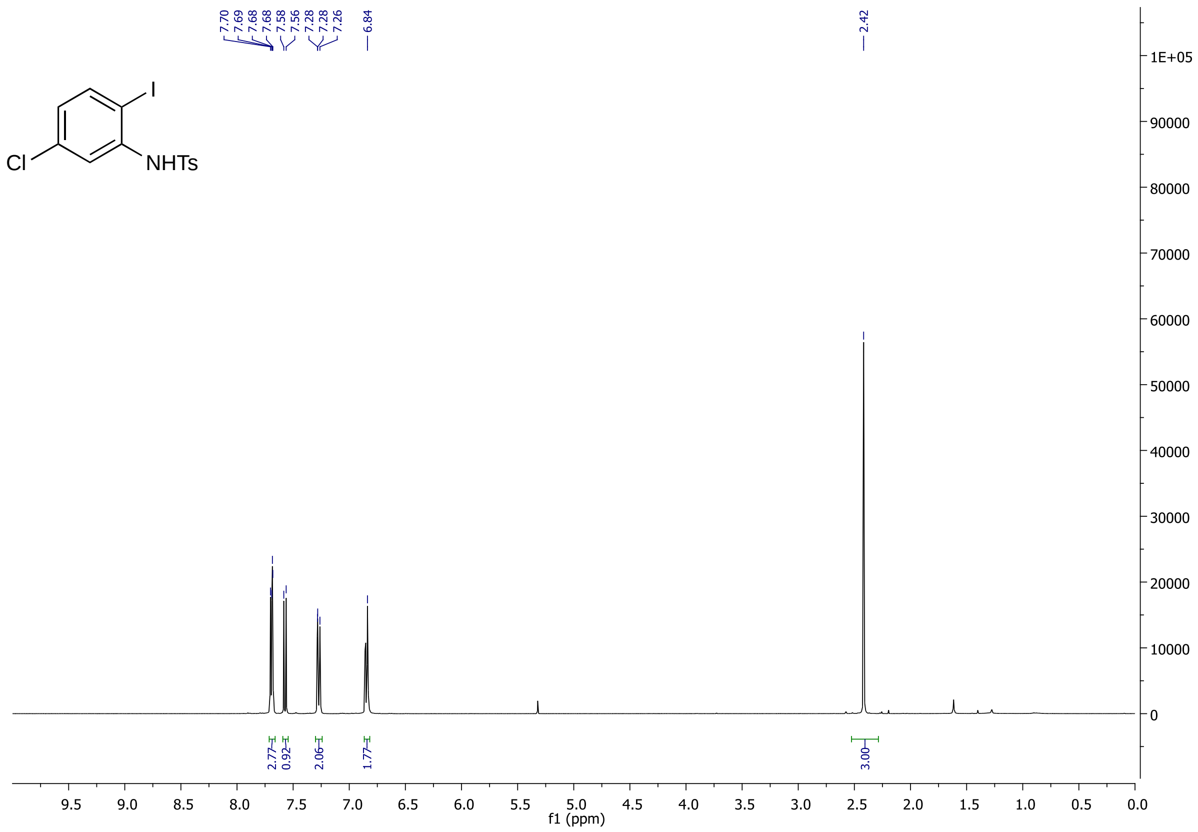

^{13}C NMR of S1

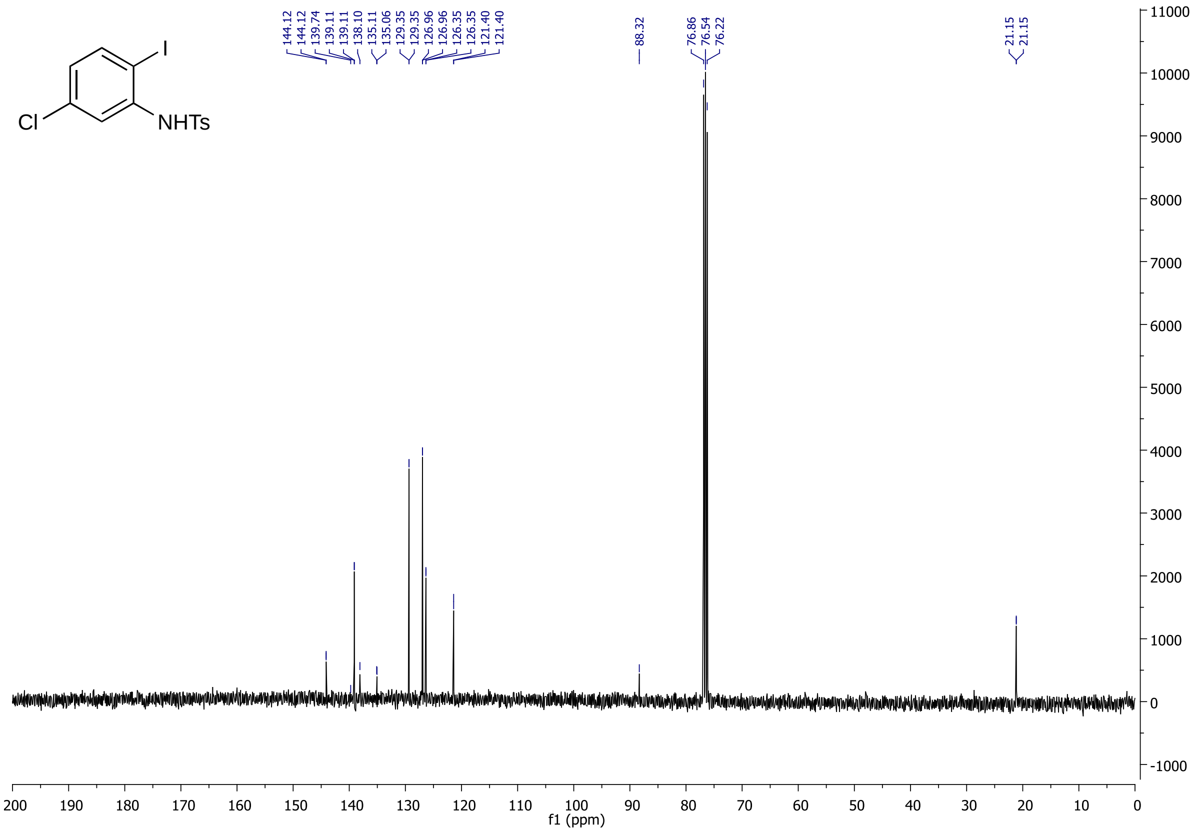

^{1}H NMR of S2

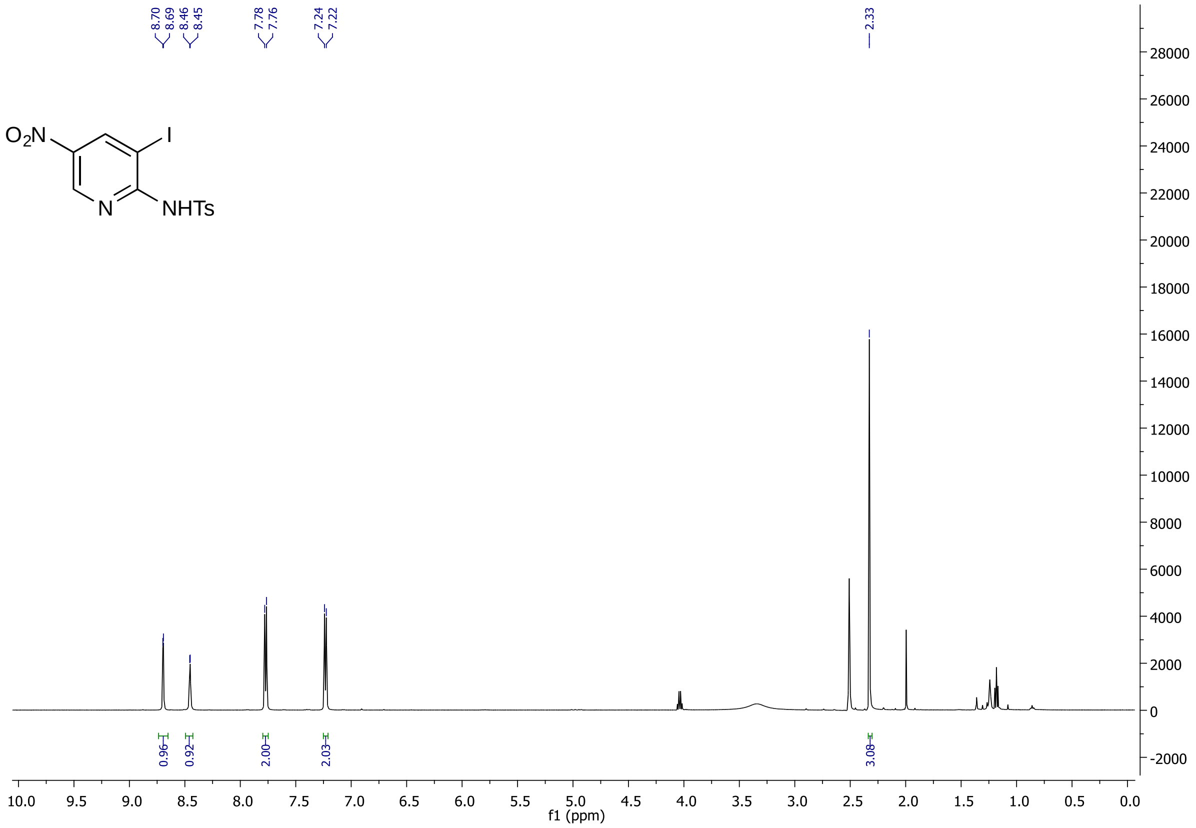

^{13}C NMR of S2

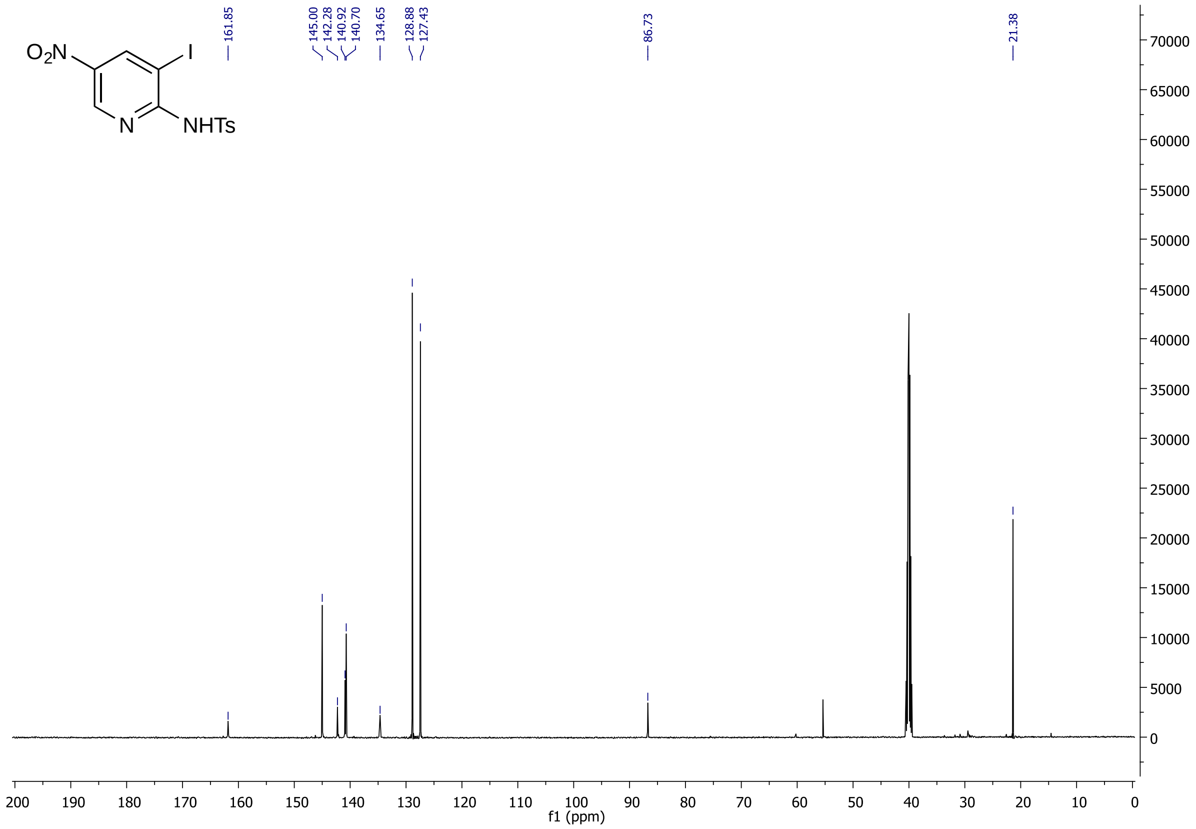

^{1}H NMR of 3a

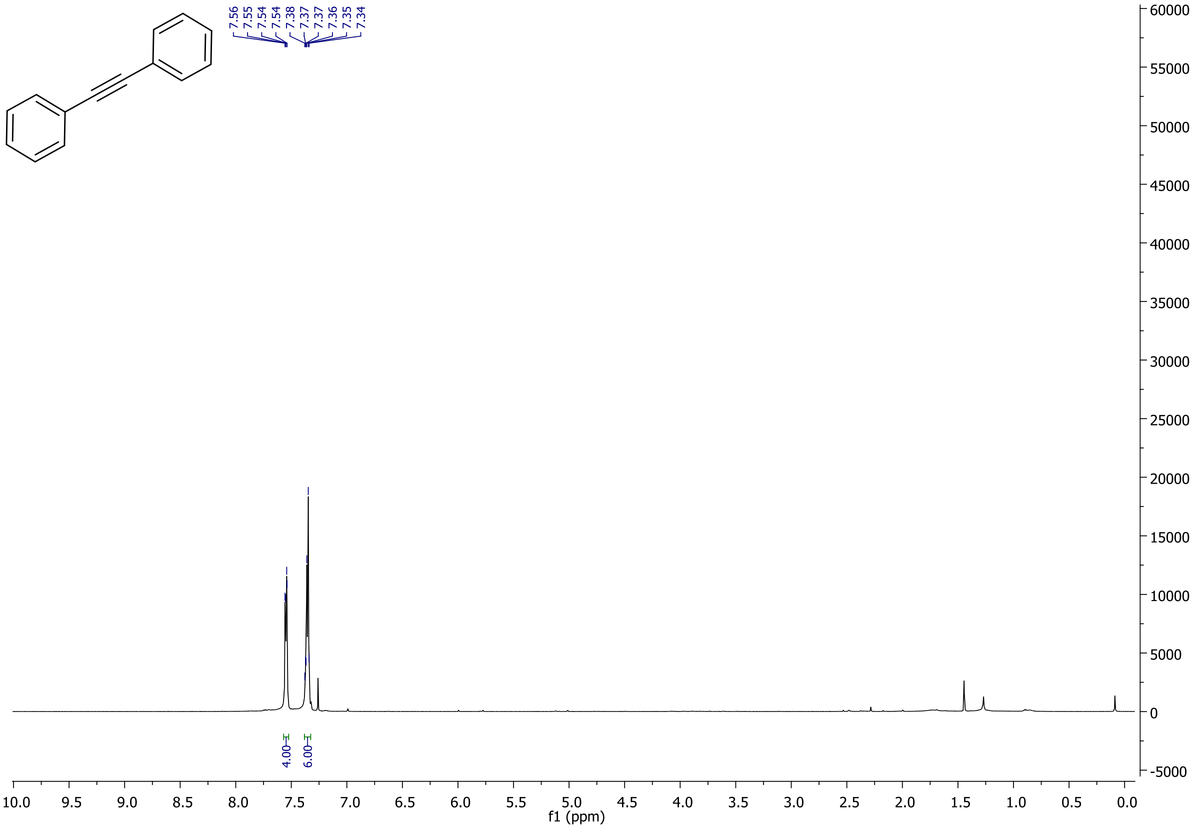

^{13}C NMR of 3a

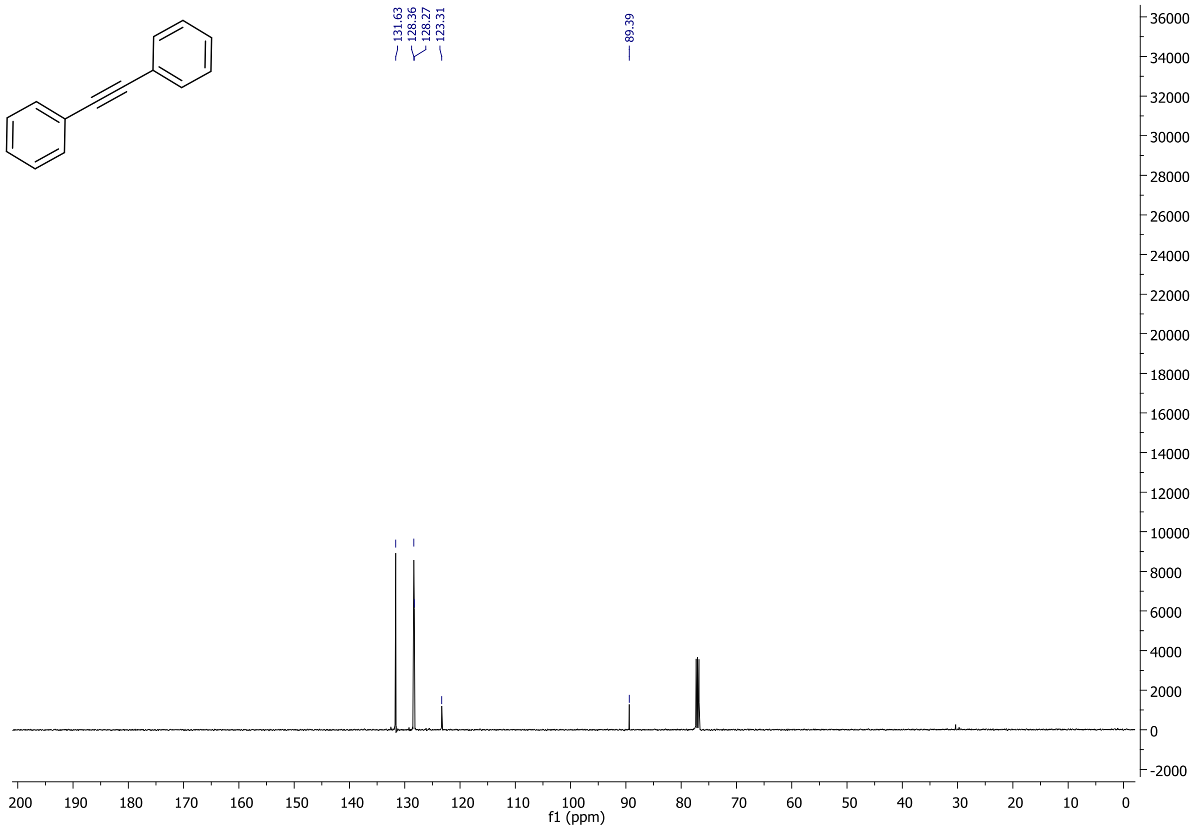

^{1}H NMR of 3b

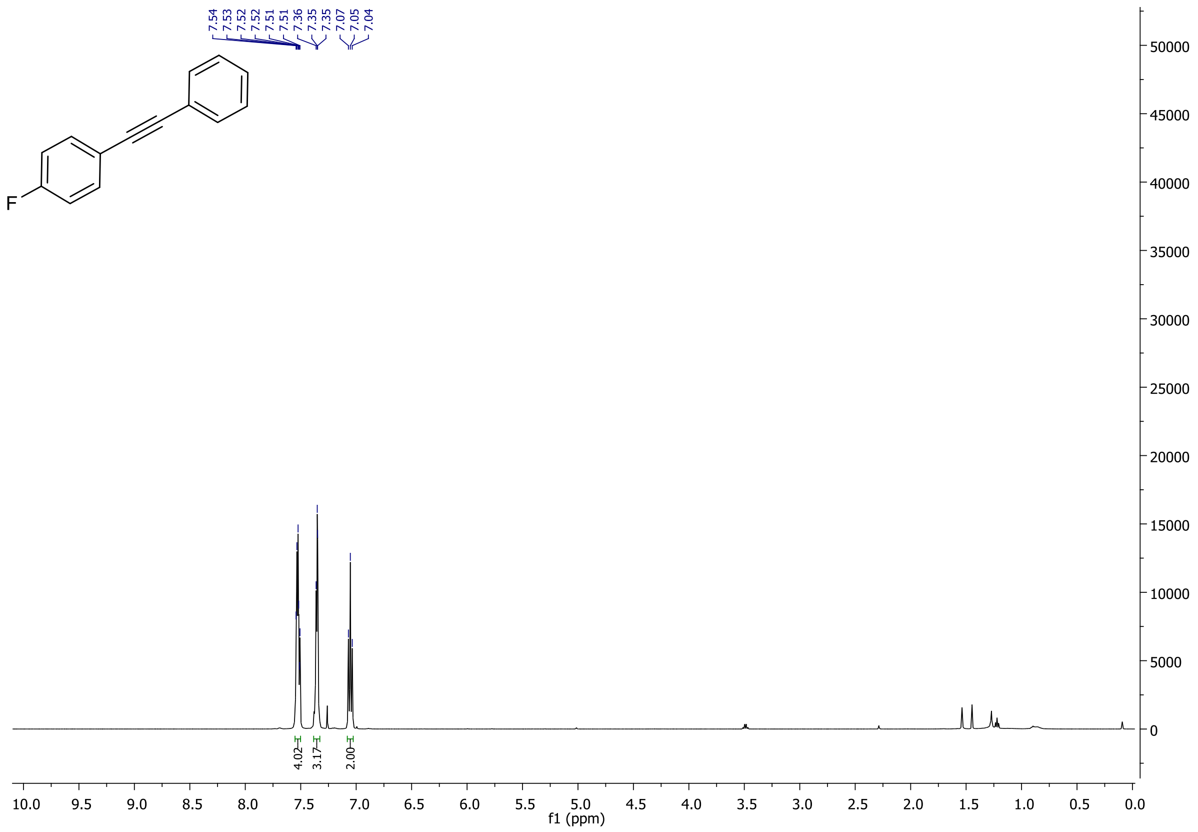

^{13}C NMR of 3b

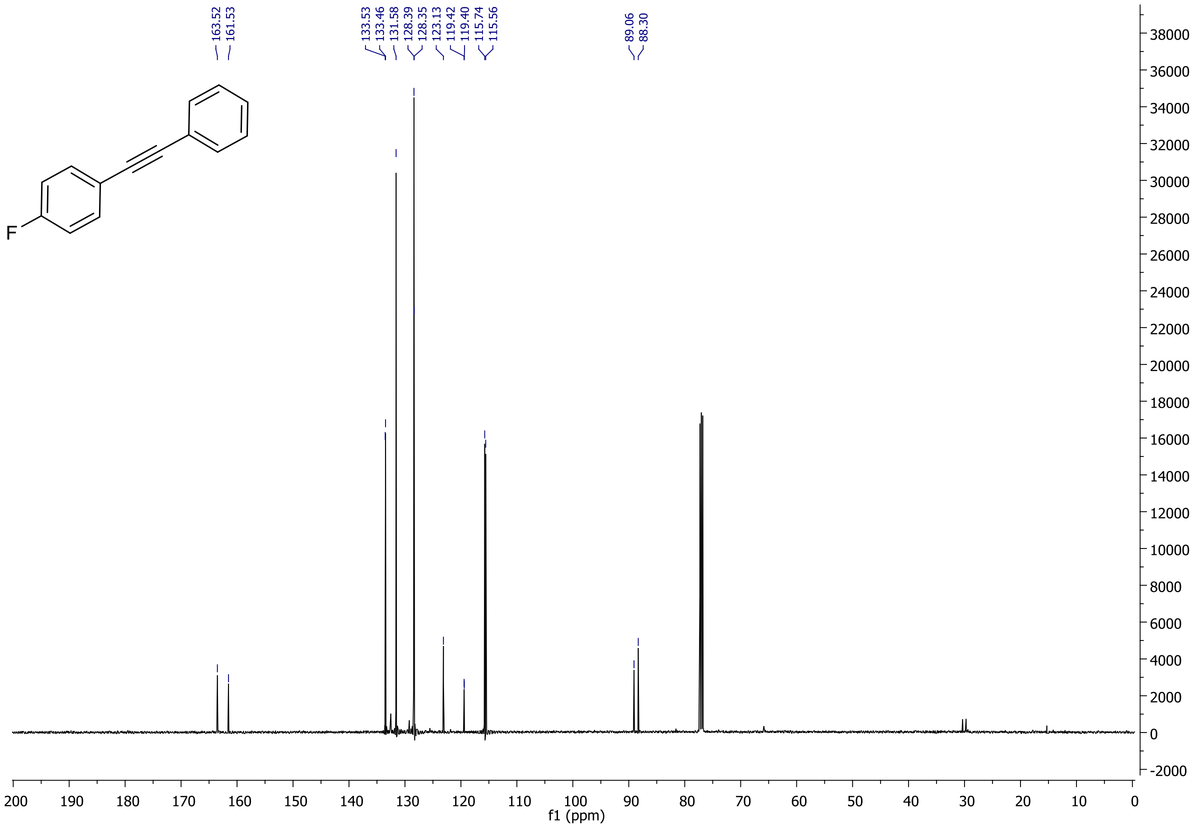

^{19}F NMR of 3b

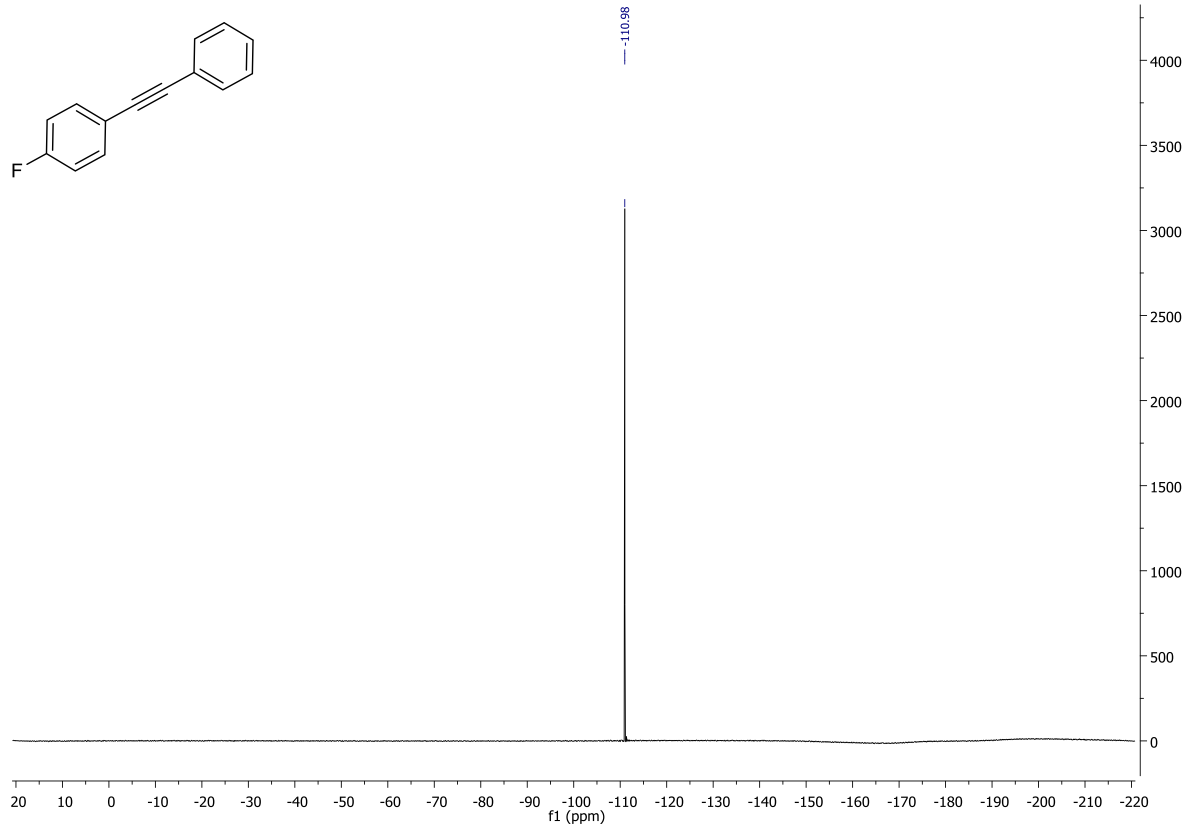
-110.98
F
f1 (ppm)

^{1}H NMR 3c

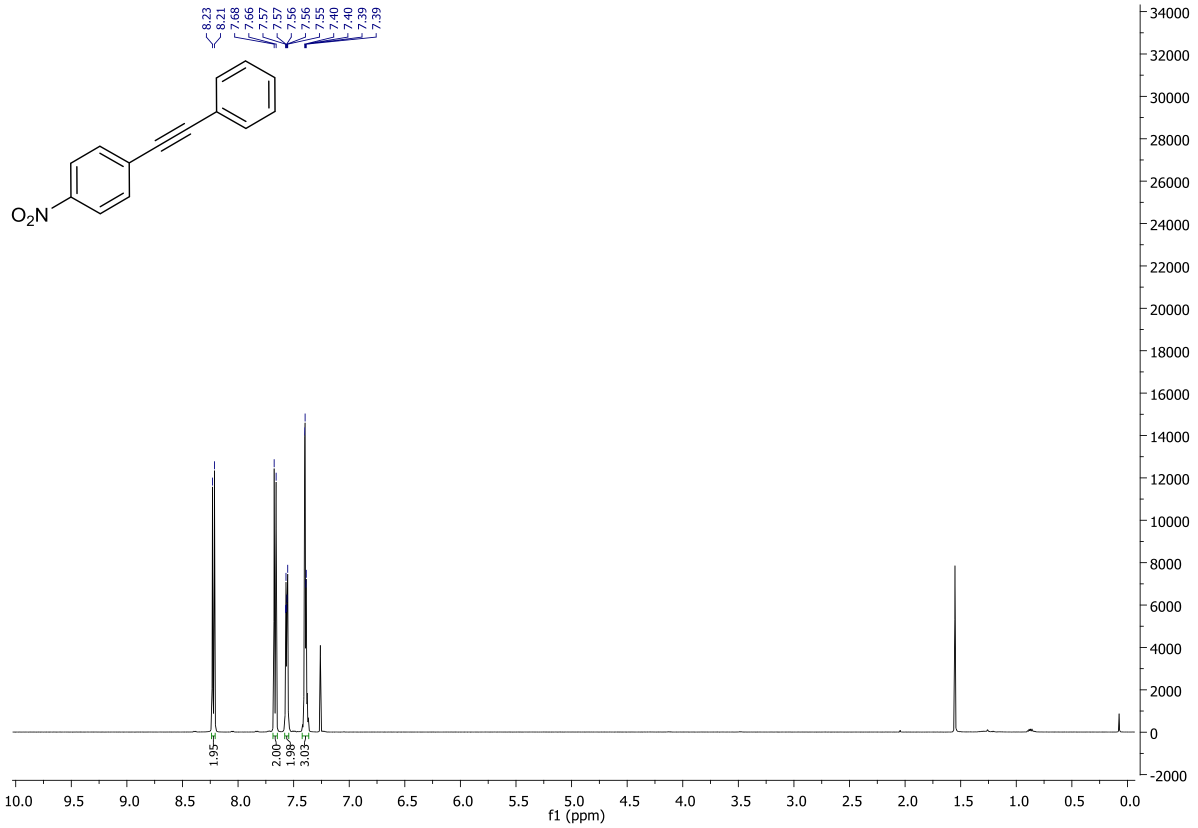
8.23
8.21
7.68
7.66
7.57
7.57
7.56
7.56
7.55
7.40
7.40
7.39
7.39
O2N
1.95
2.00
1.98
3.03
f1 (ppm)

^{13}C NMR 3c

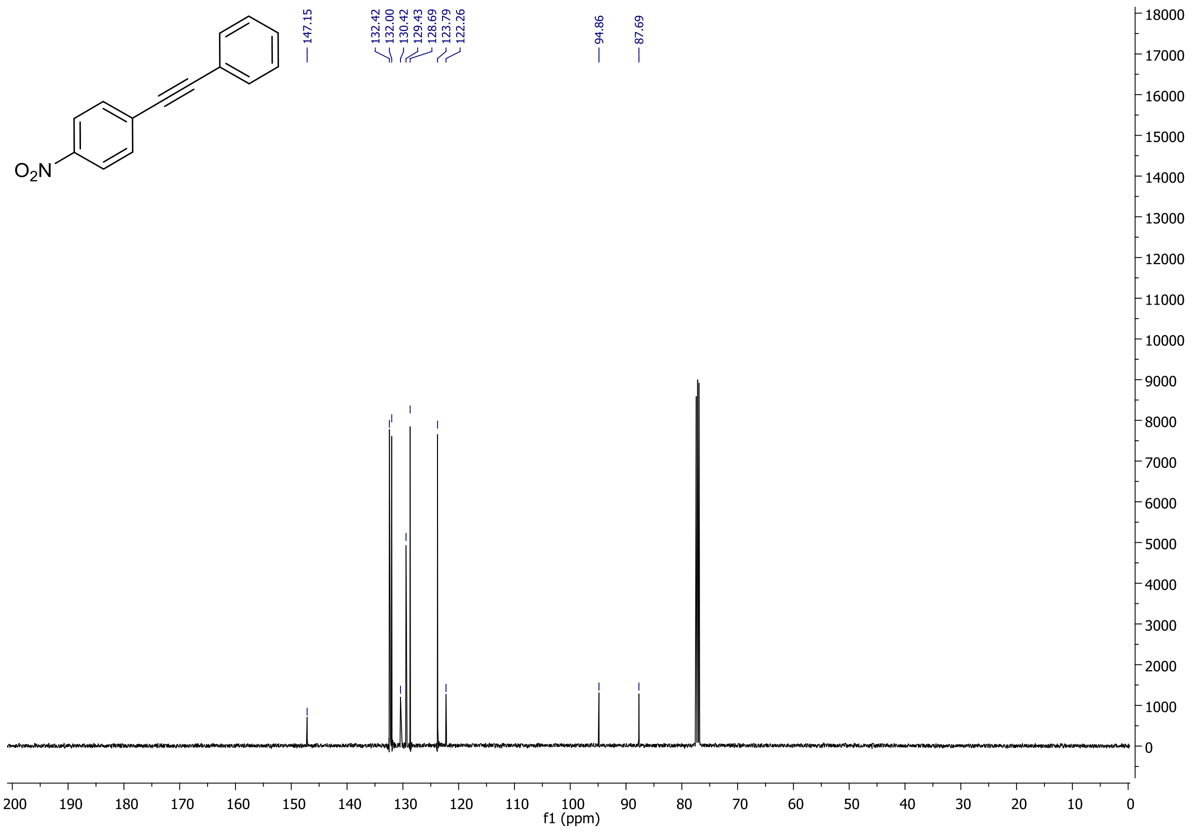

^{1}H NMR 3d

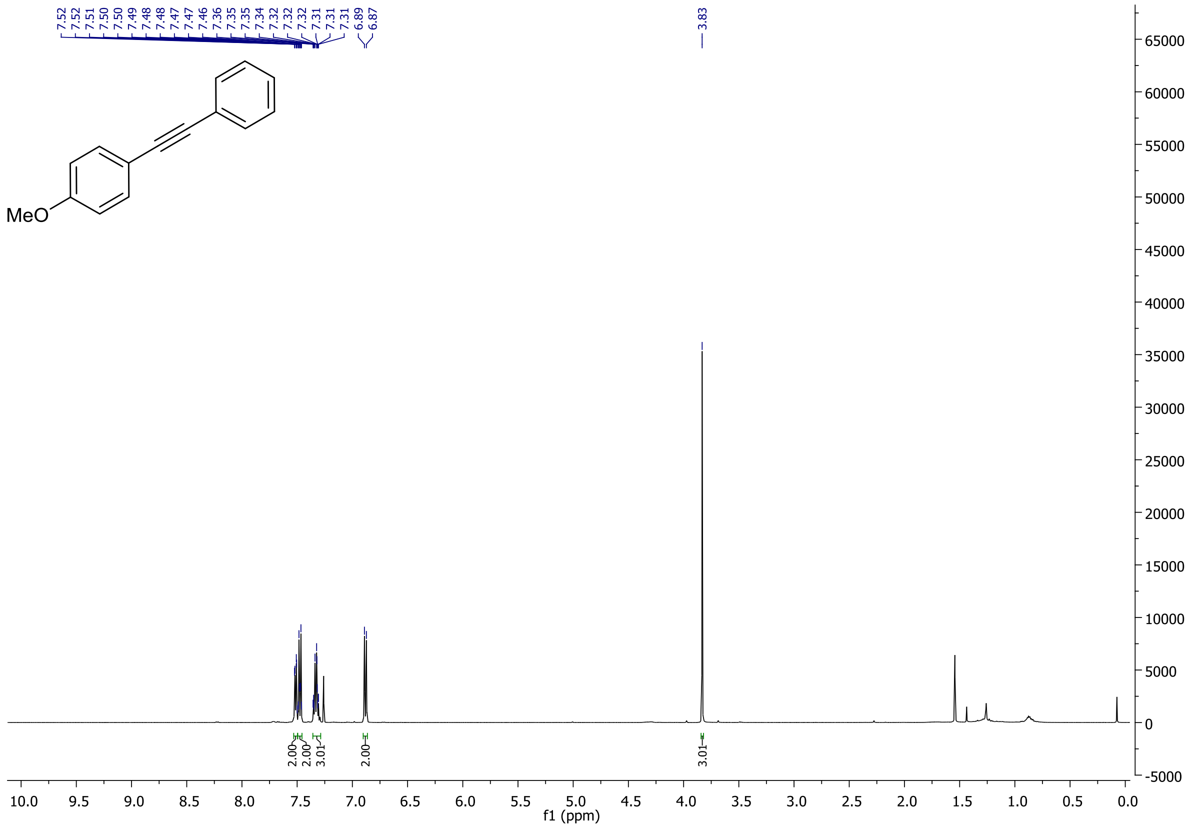

^{13}C NMR 3d

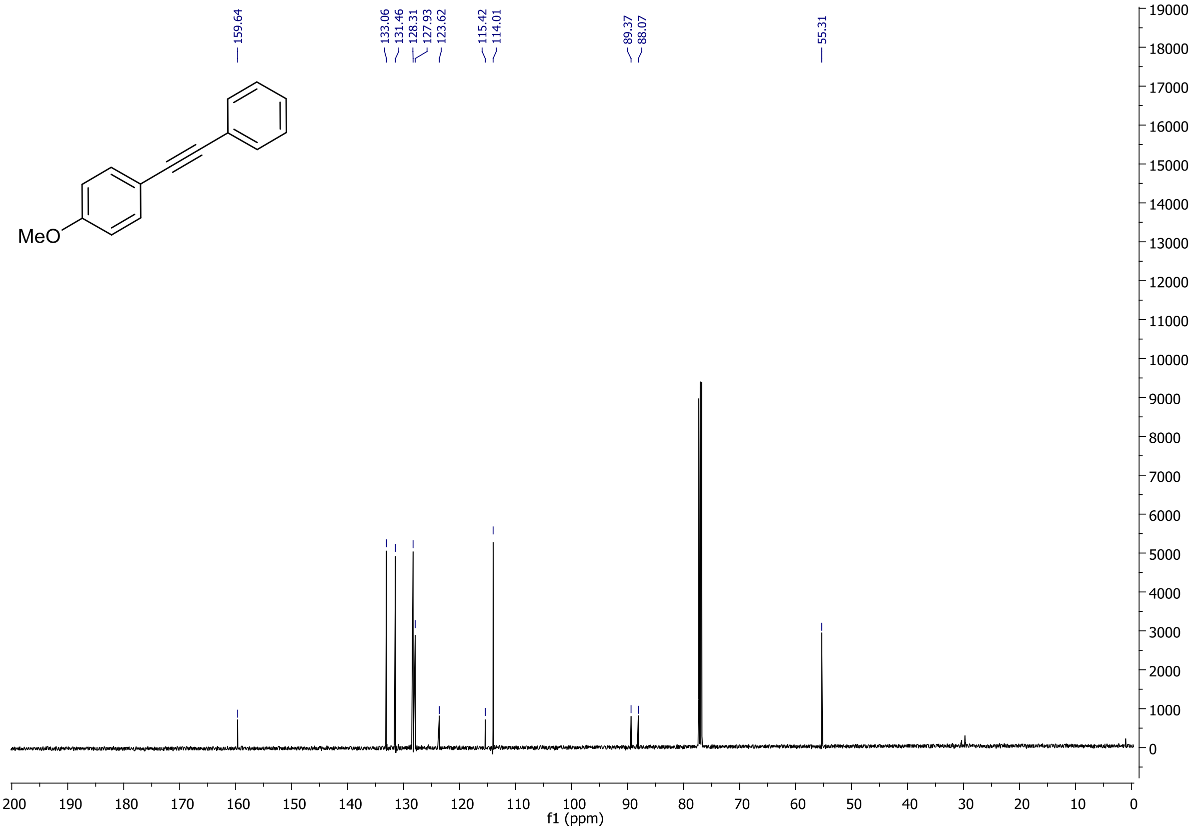

^{1}H NMR 3e

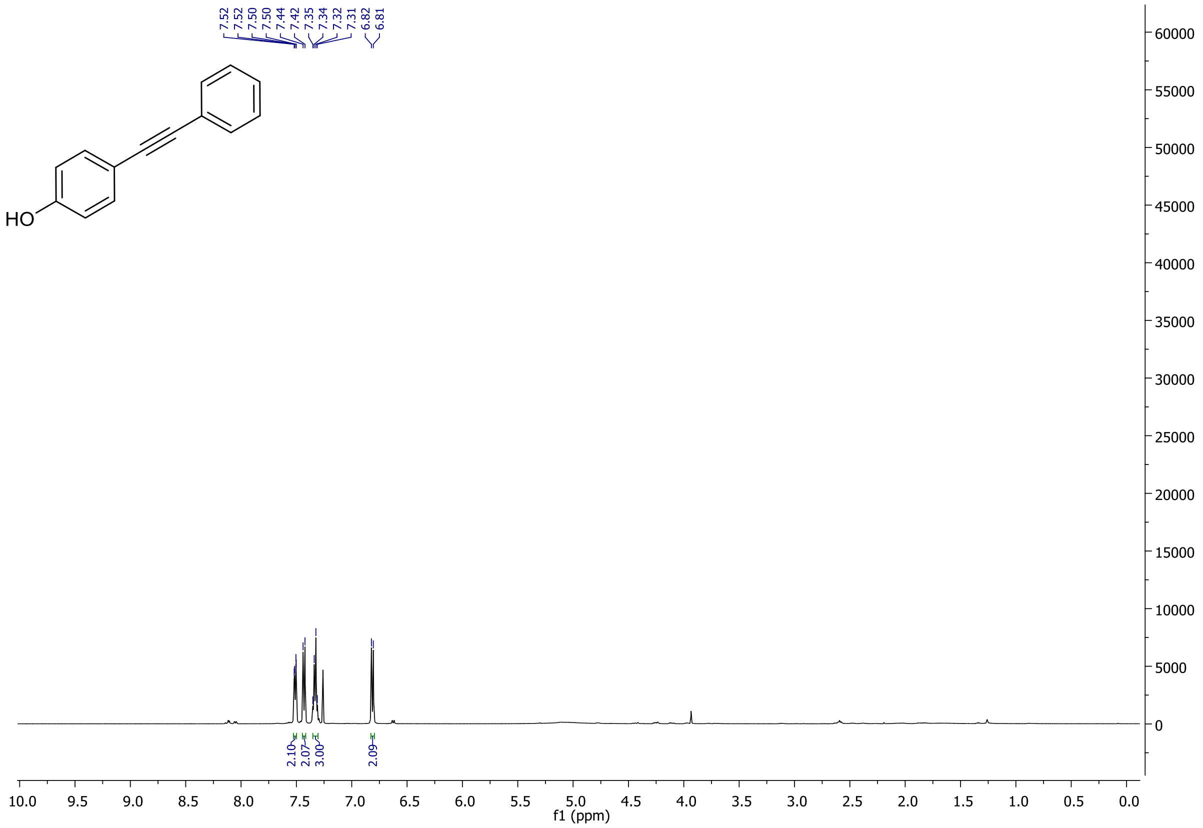

^{13}C NMR 3e

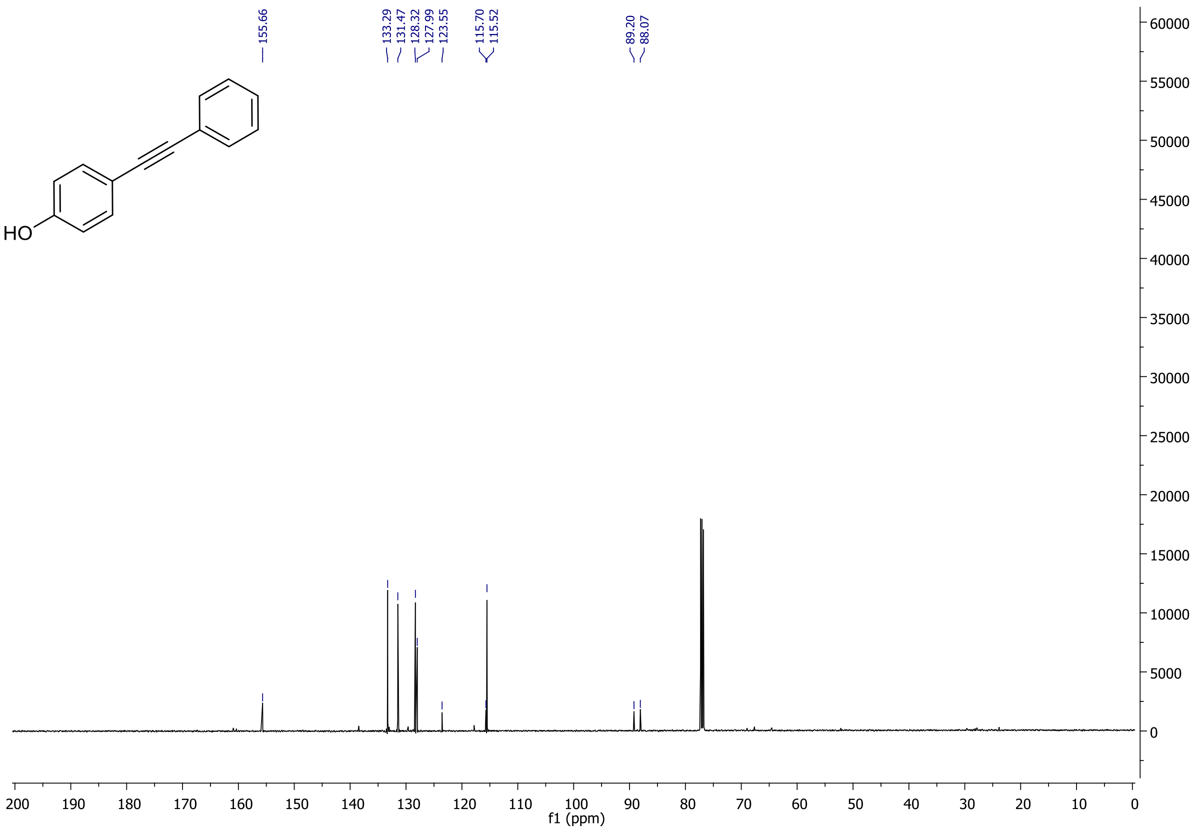

^{1}H NMR 3f

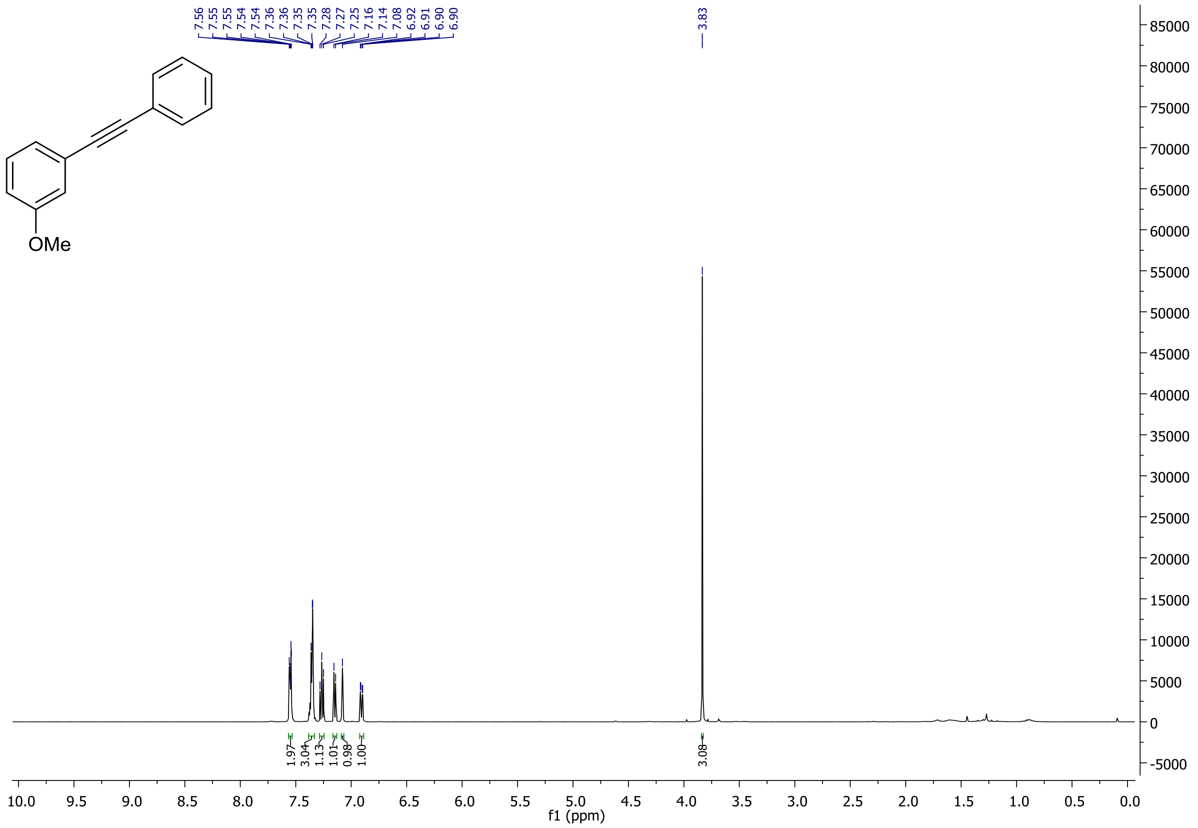

^{13}C NMR 3f

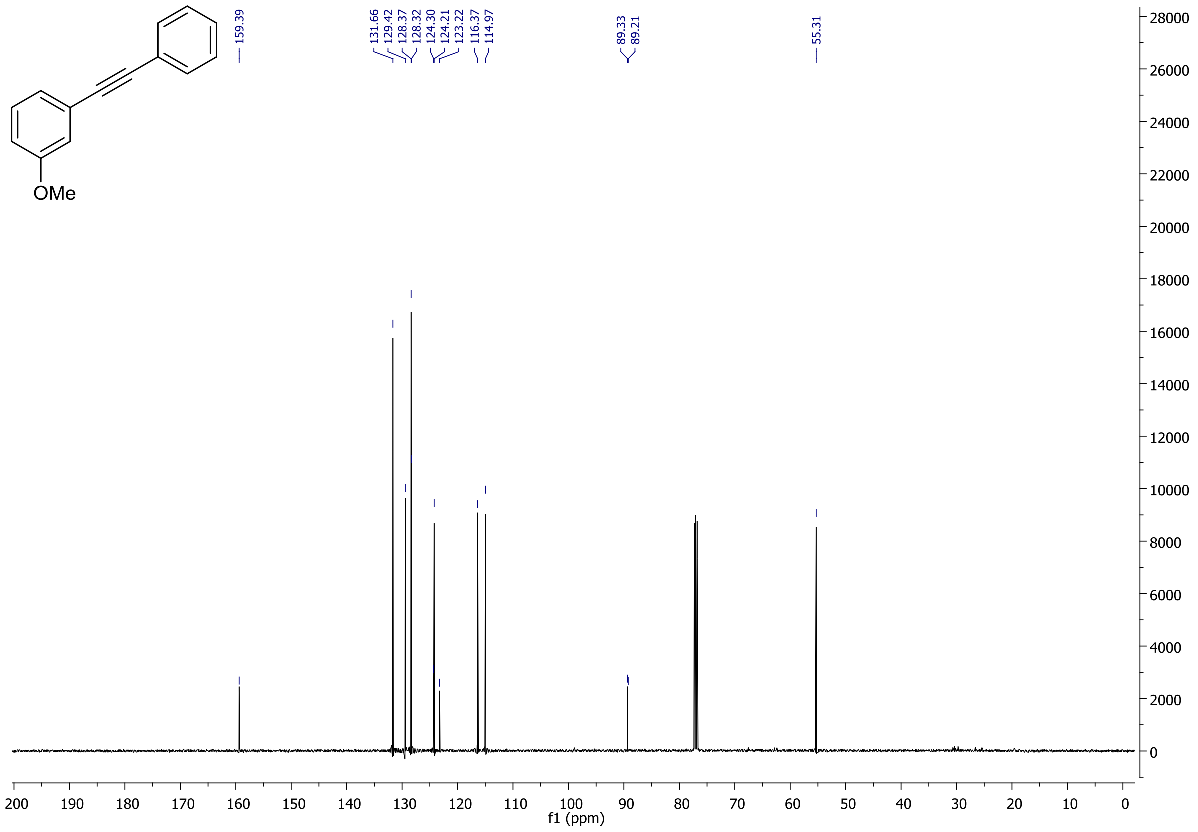

^{1}H NMR 3g

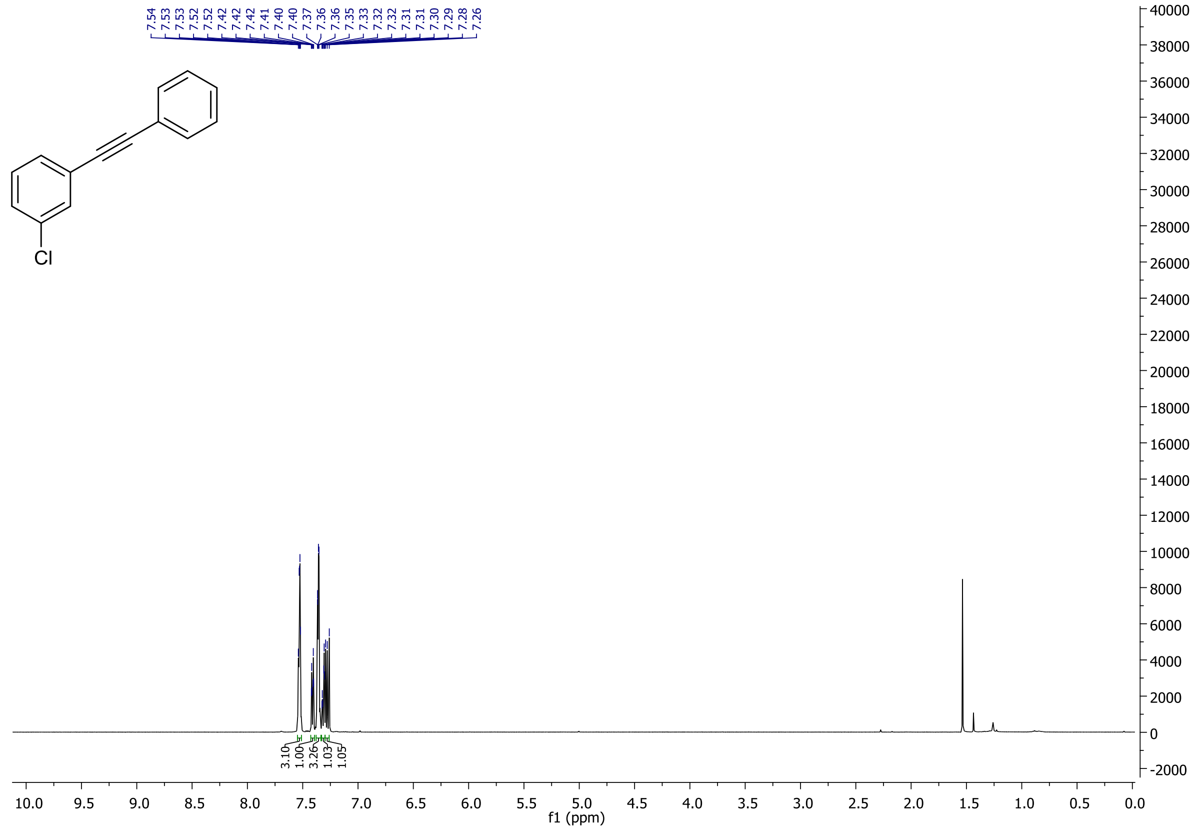

^{13}C NMR 3g

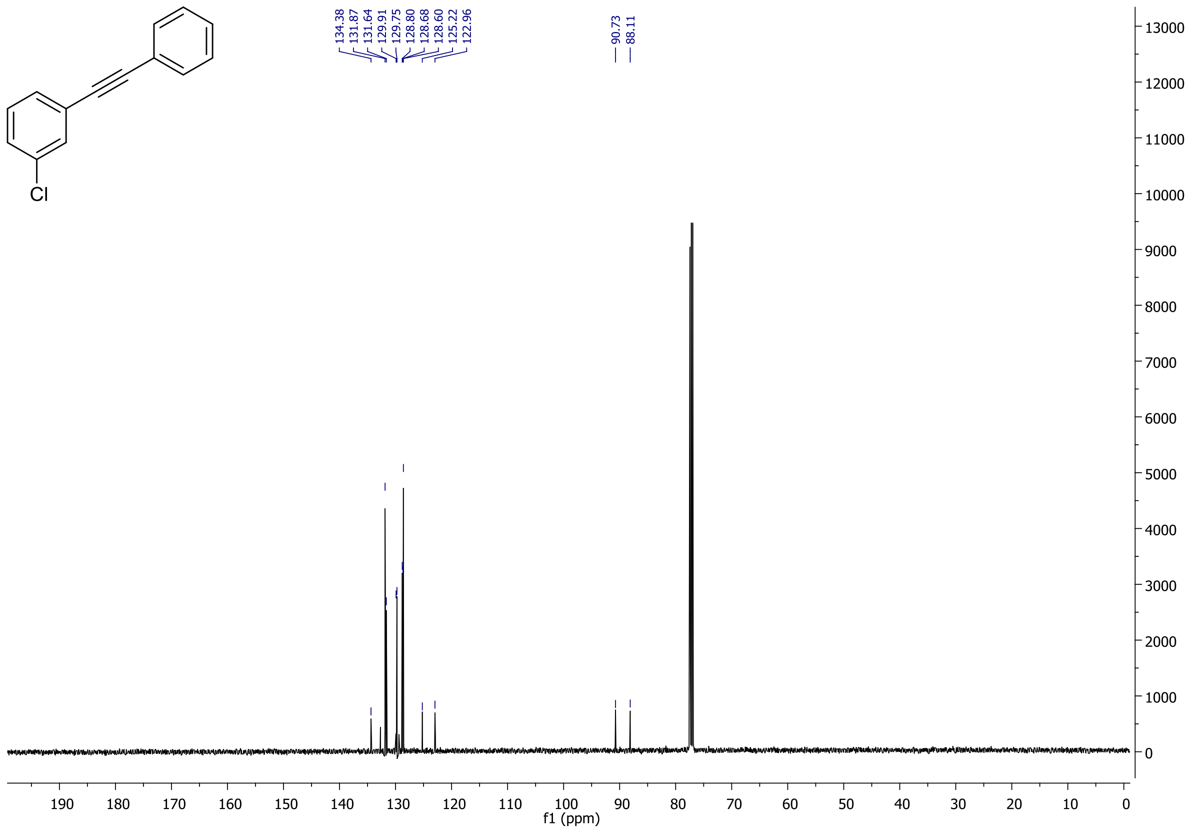

^{1}H NMR 3h

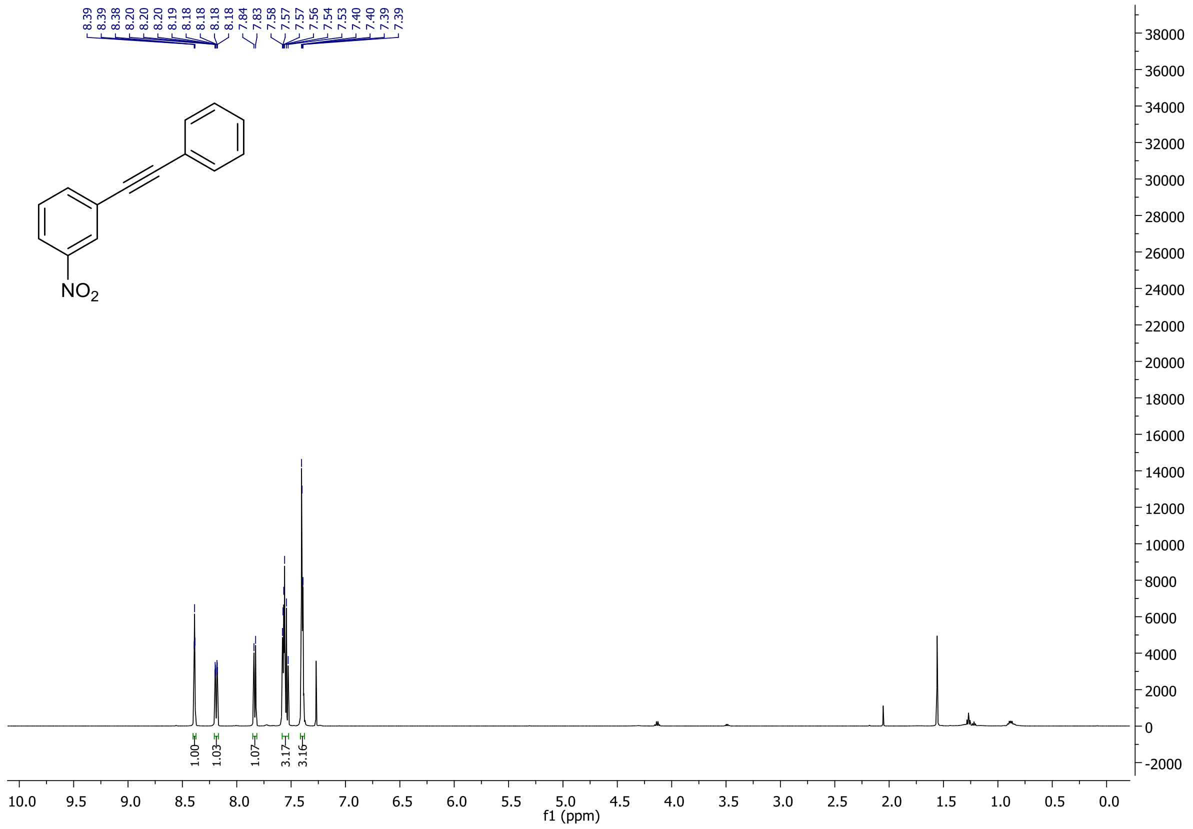

^{13}C NMR 3h

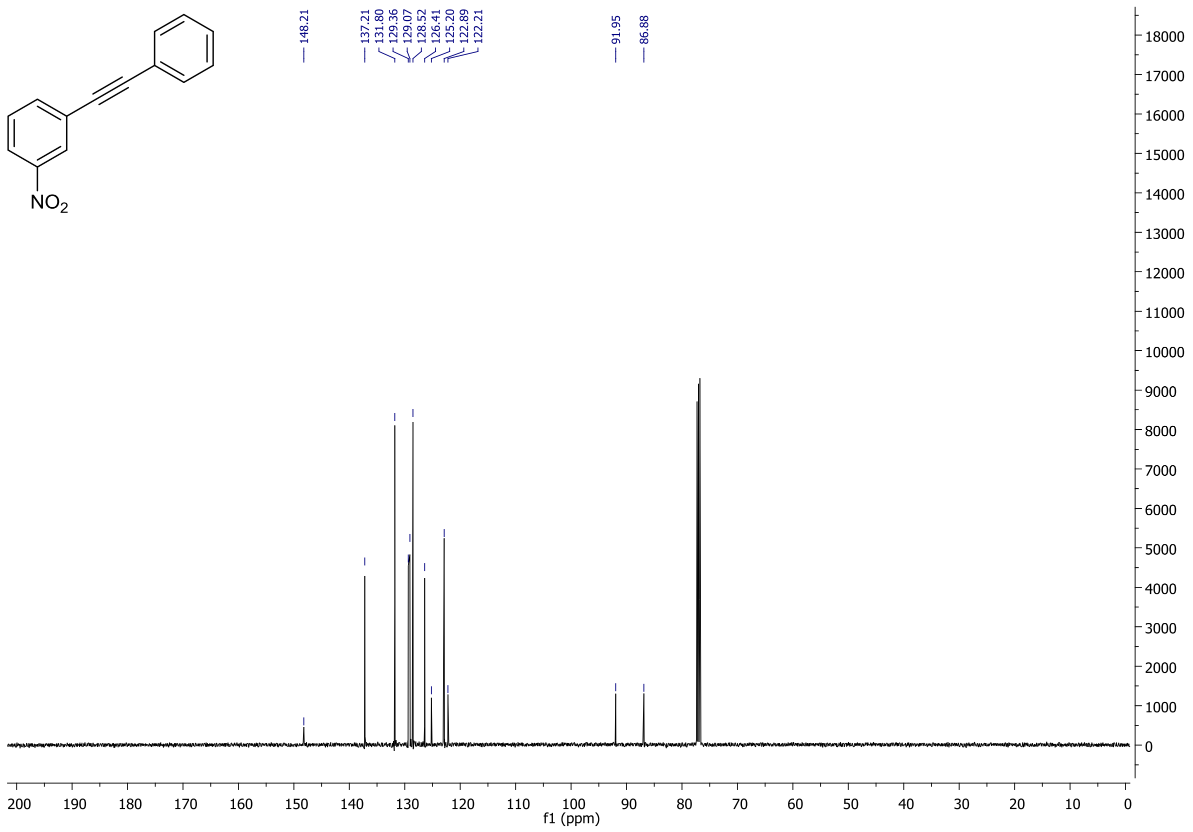

^{1}H NMR 3i

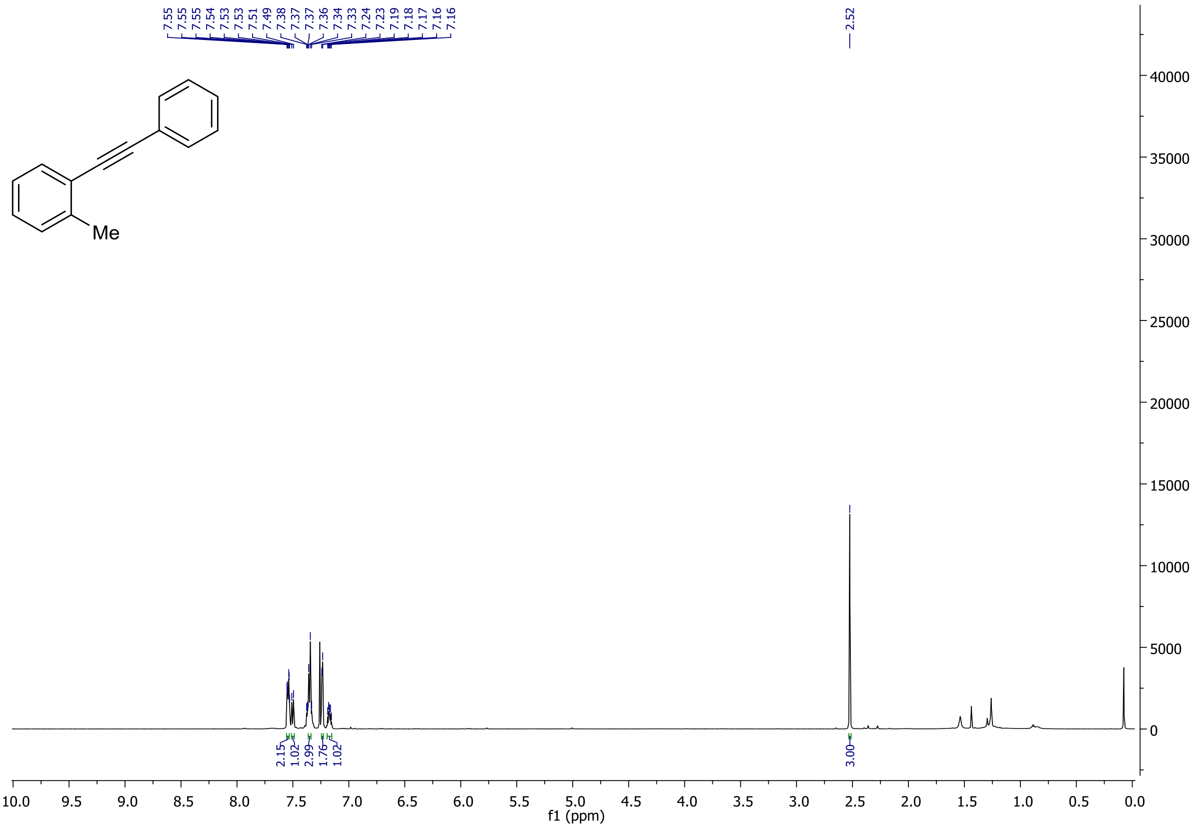

^{13}C NMR 3i

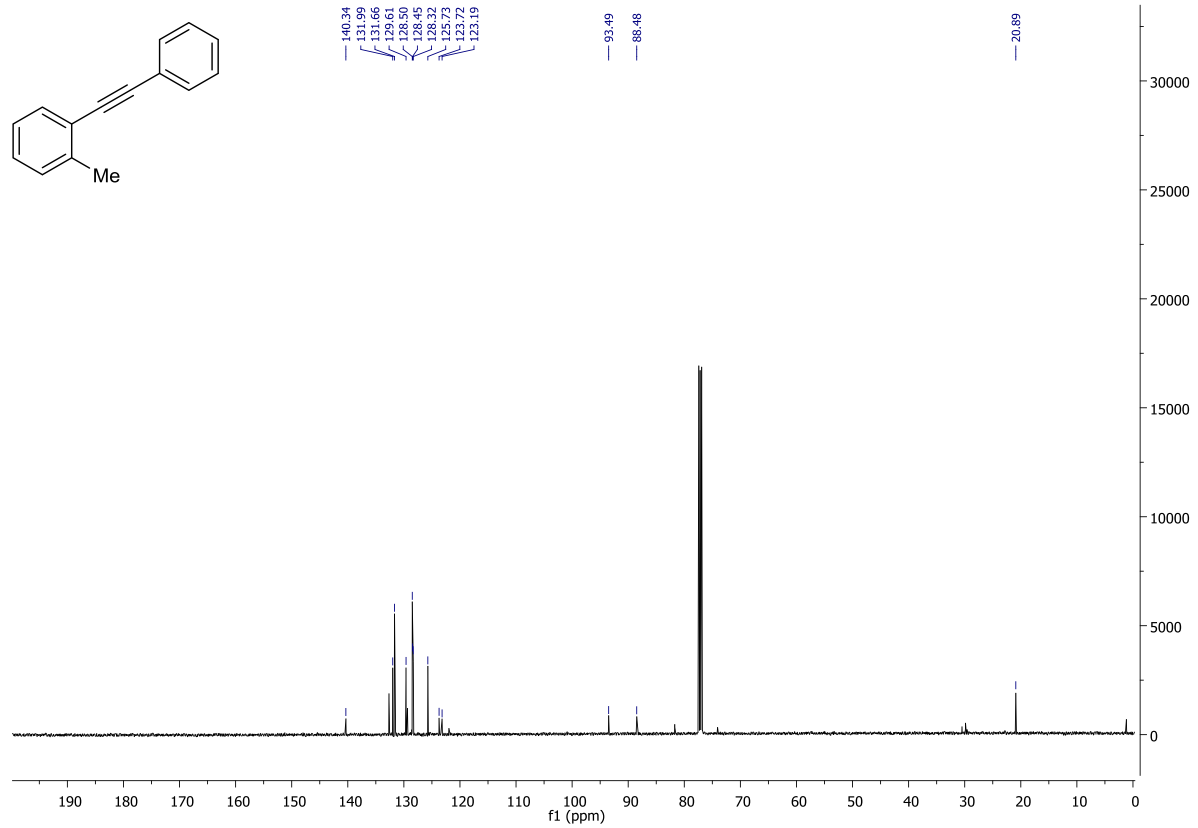

^{1}H NMR 3j

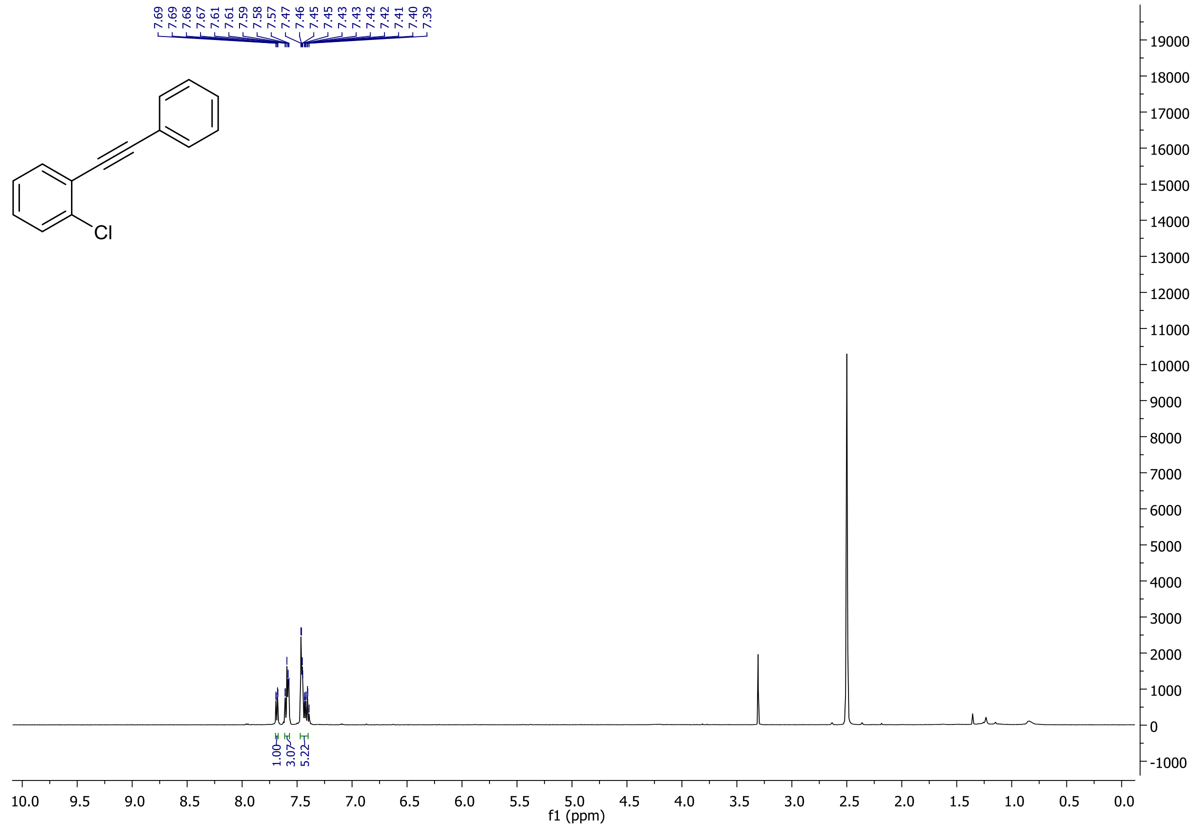

^{13}C NMR 3j

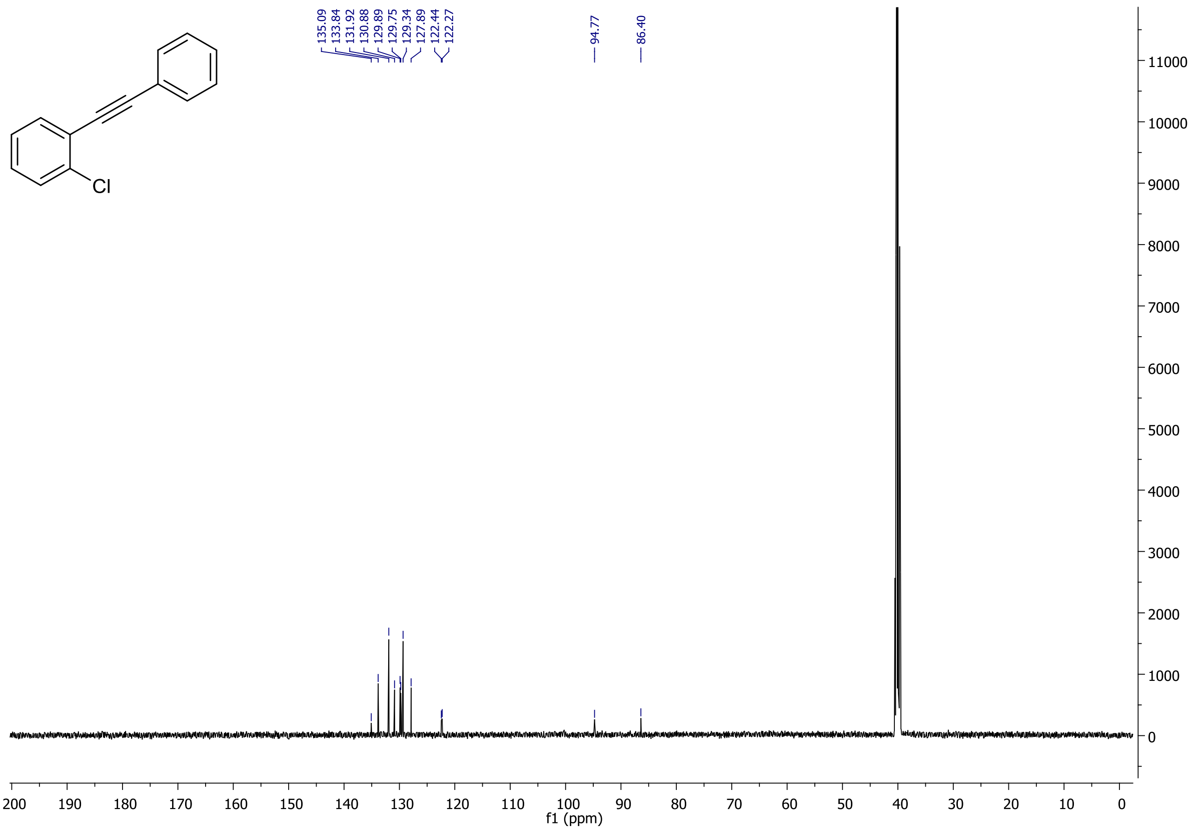

^{1}H NMR 3k

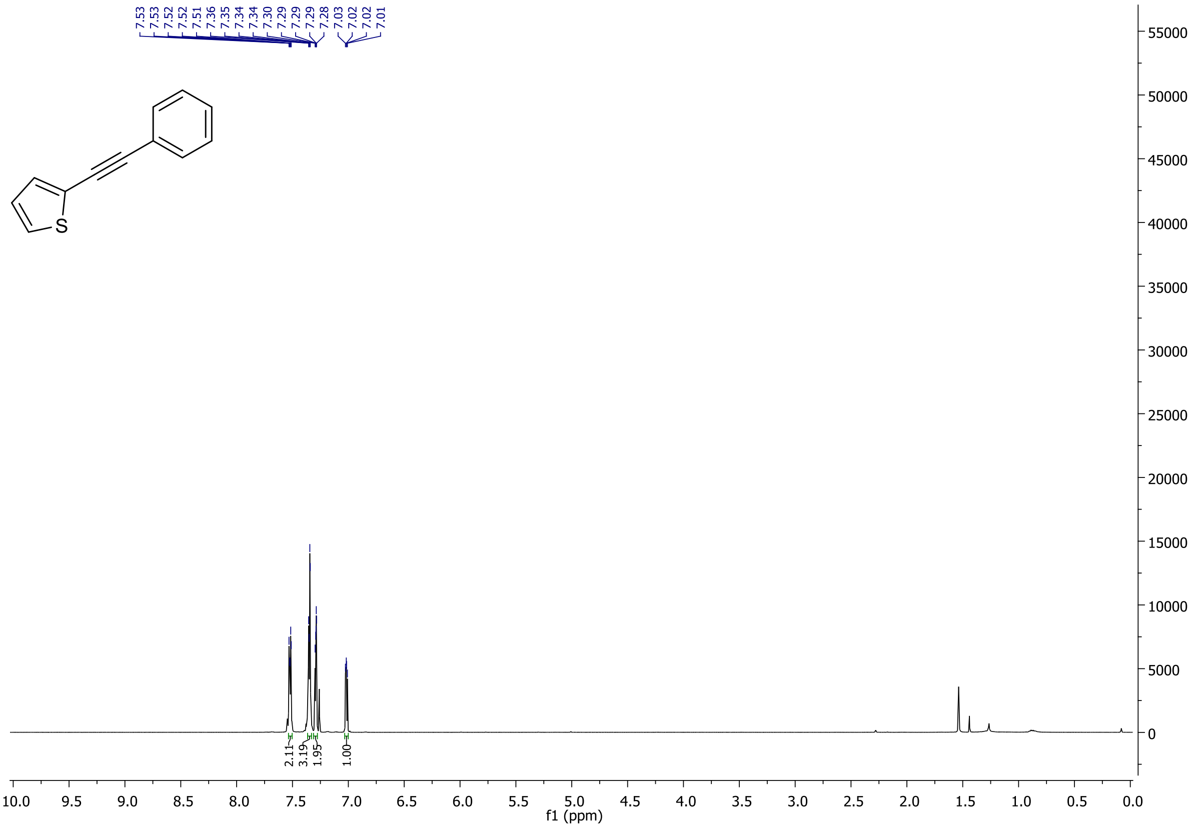

^{13}C NMR 3k

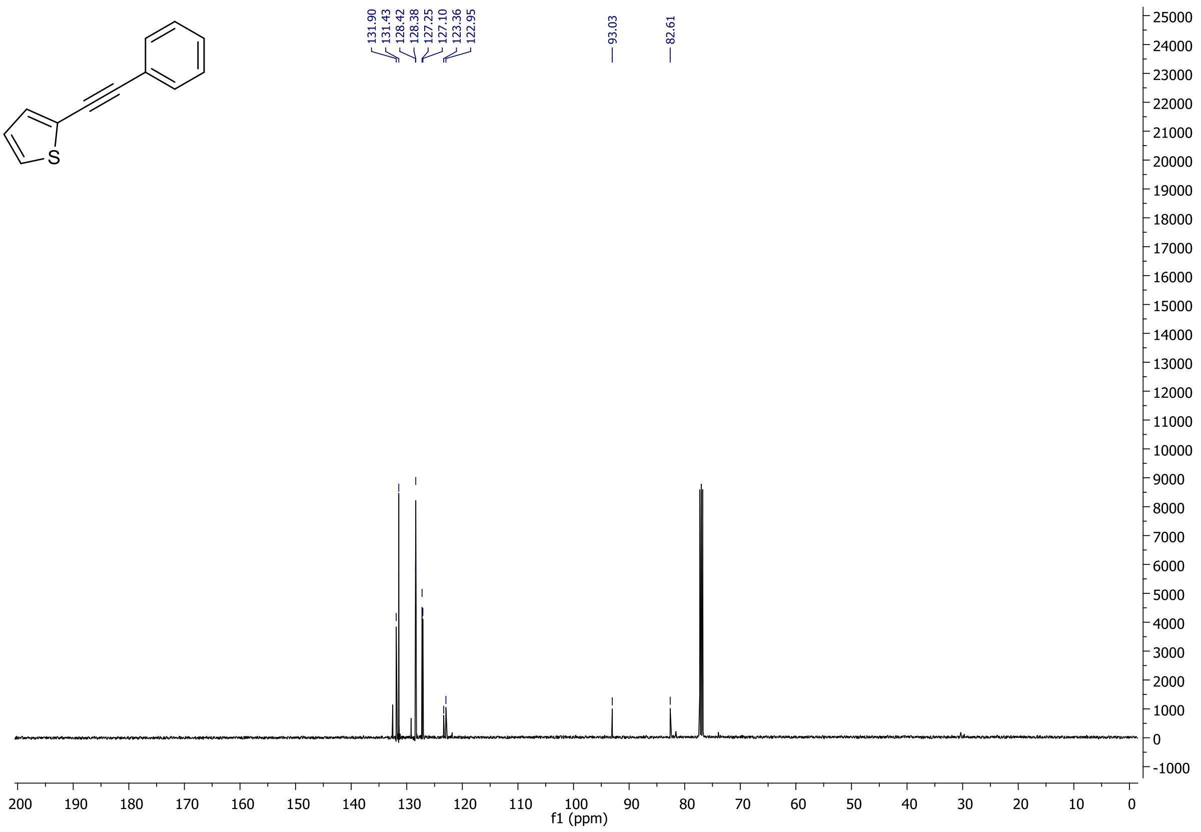

^{1}H NMR 3l

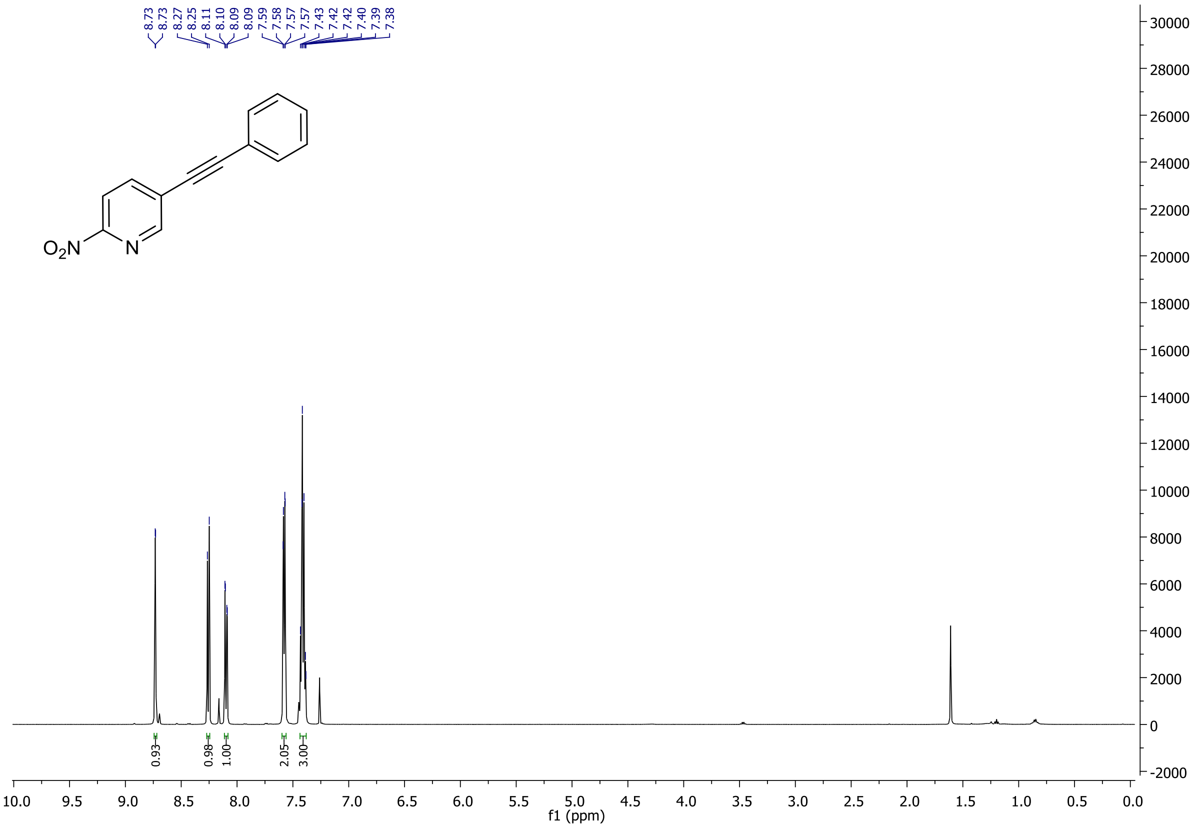

^{13}C NMR 3l

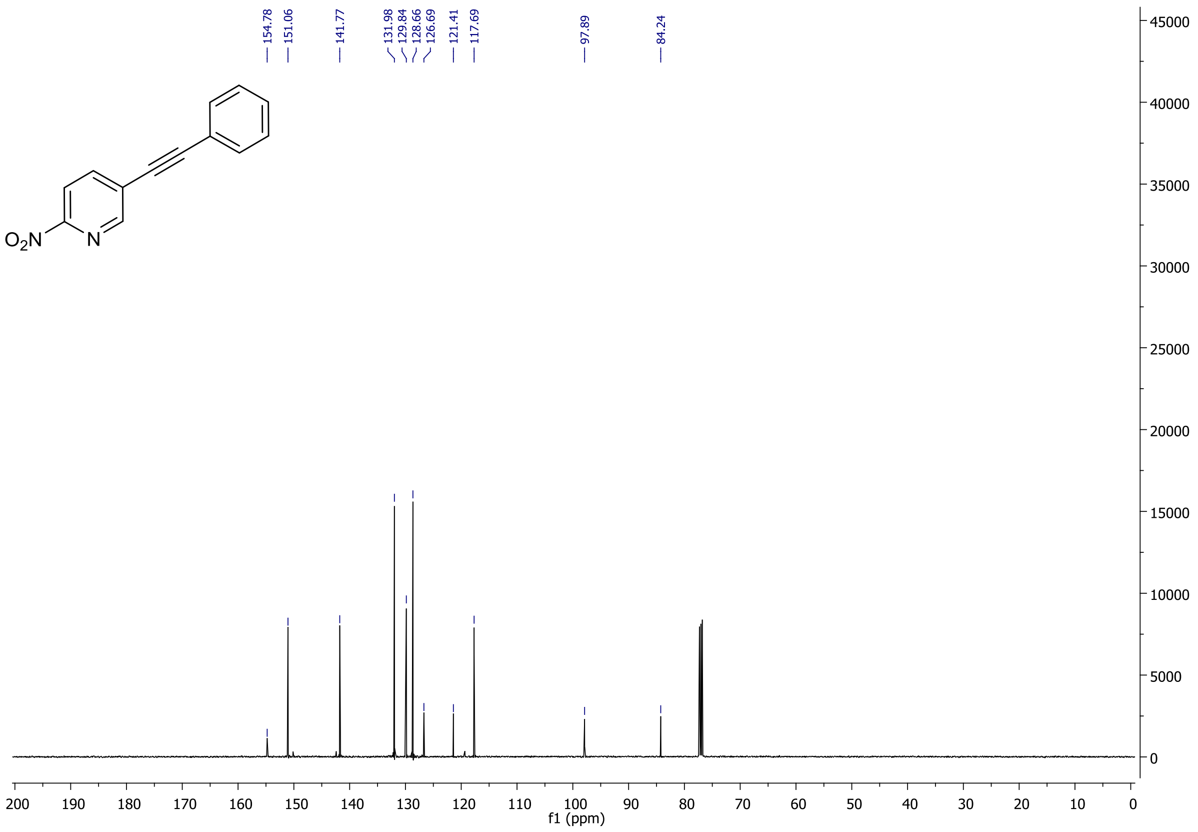

^{1}H NMR 3m

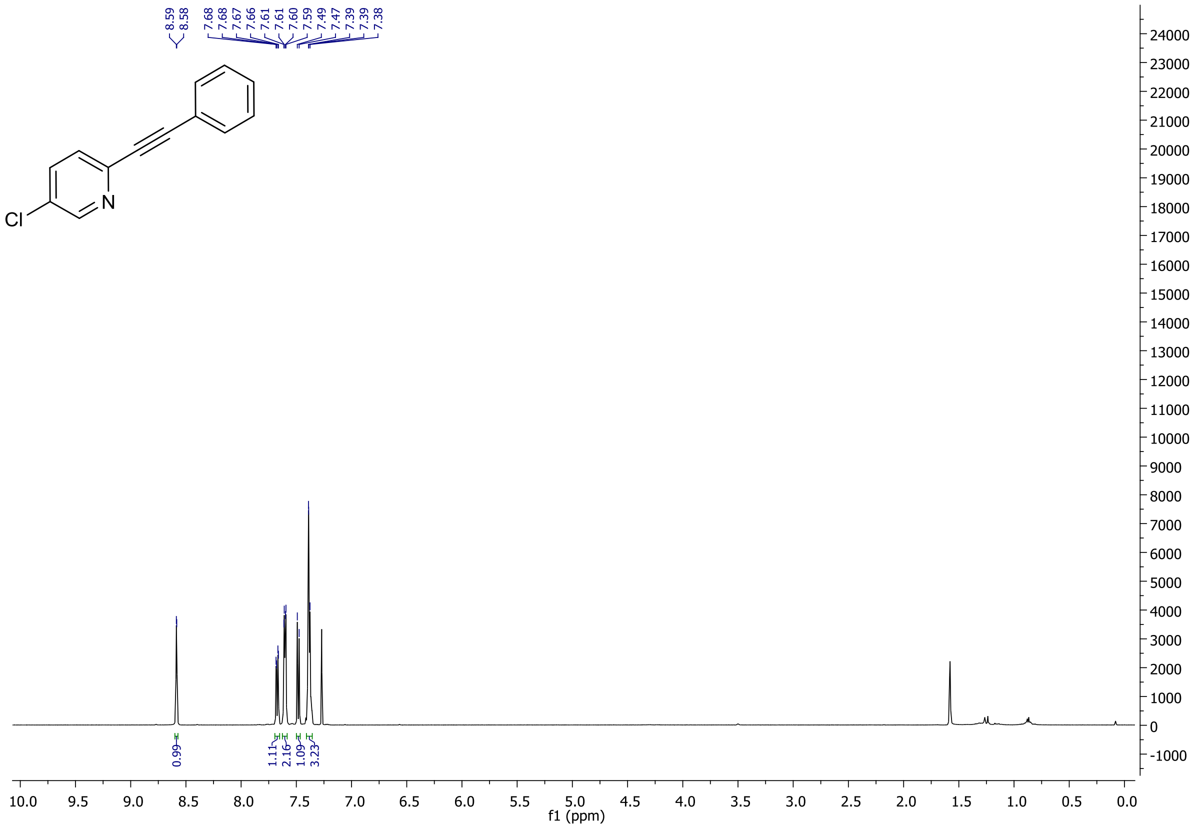

^{13}C NMR 3m

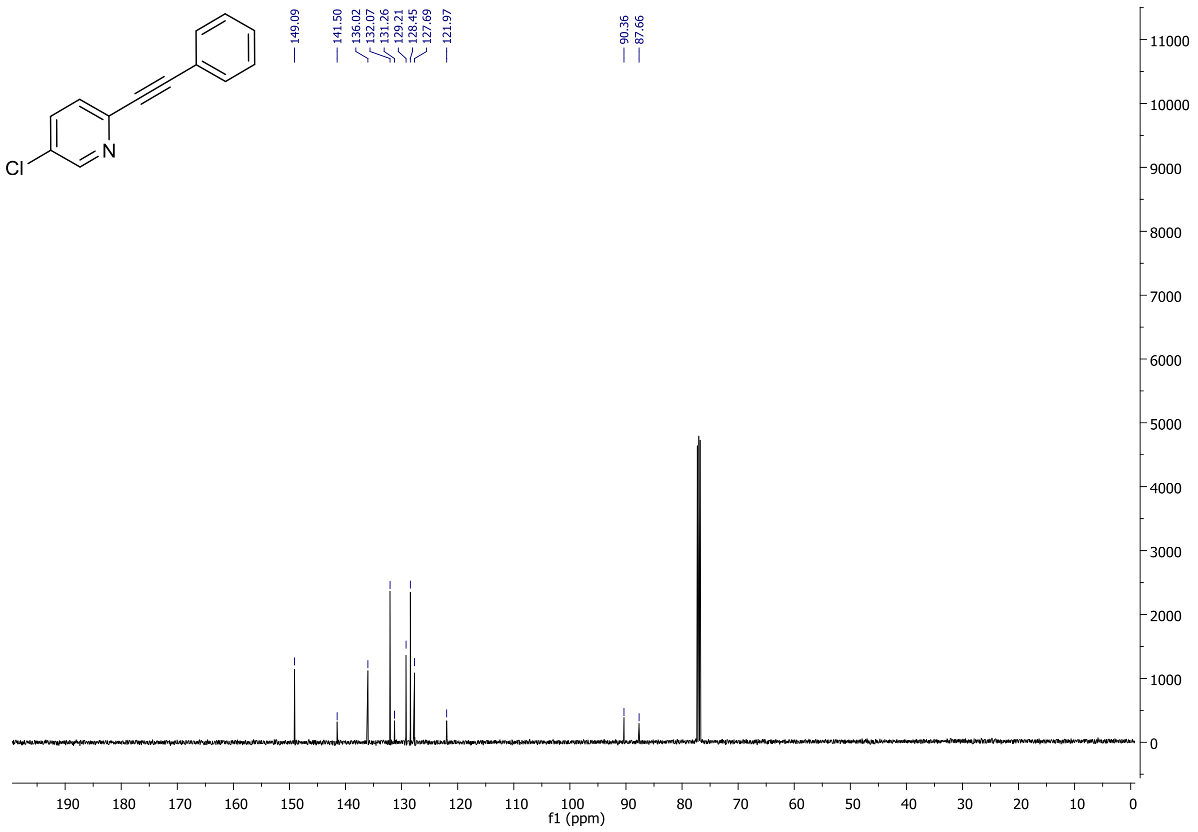

^{1}H NMR 3n

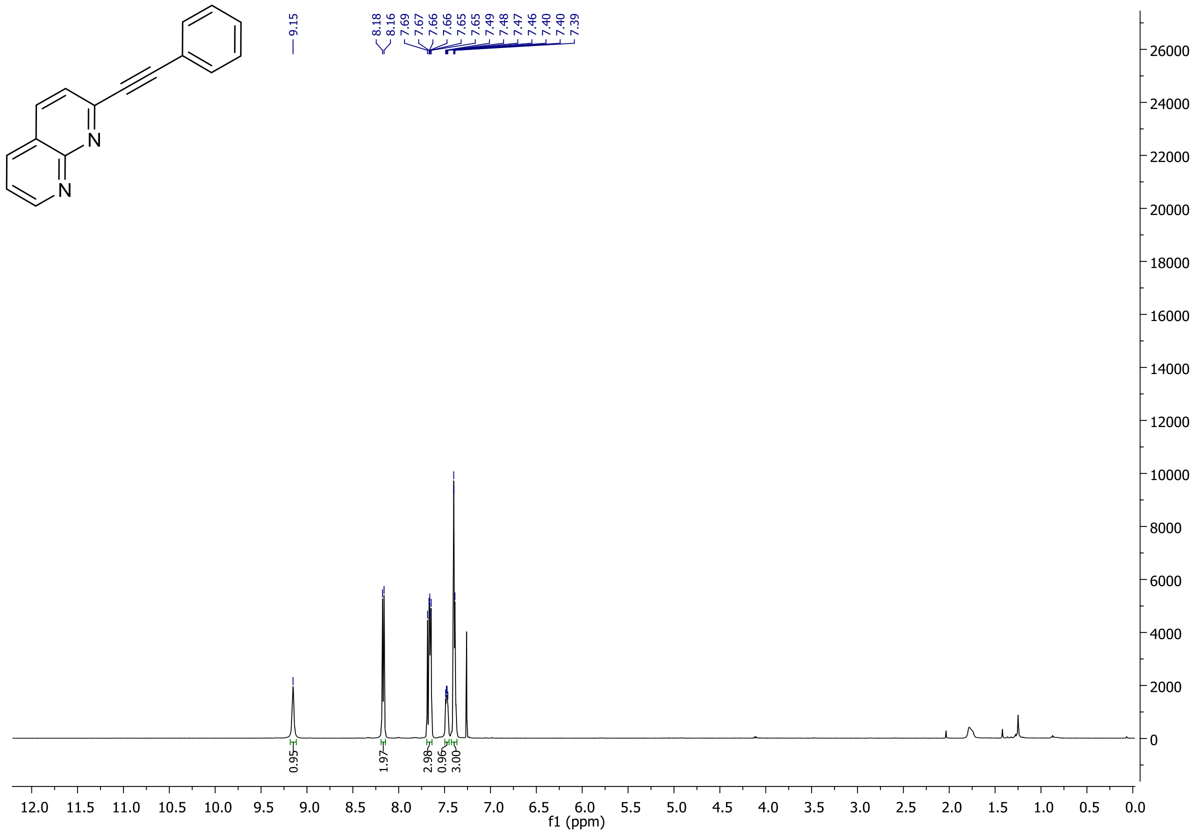

^{13}C NMR 3n

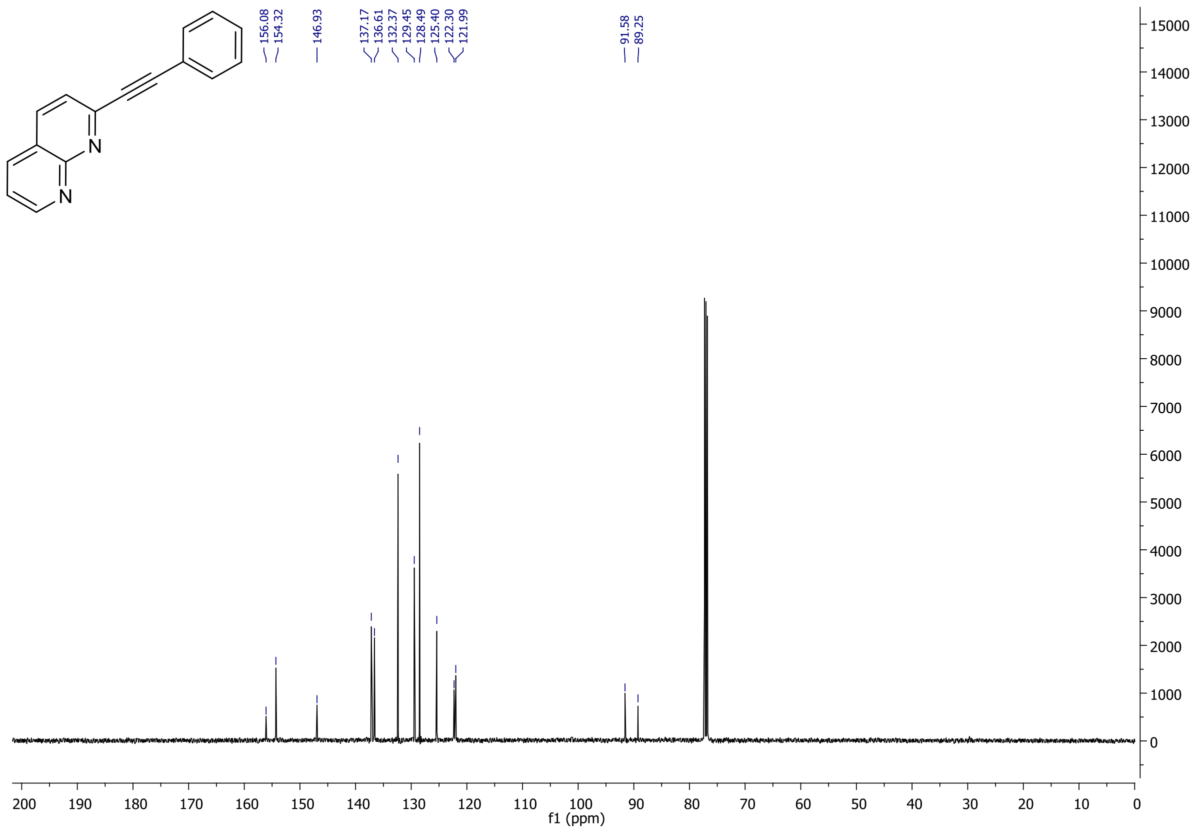

^{1}H NMR 3o

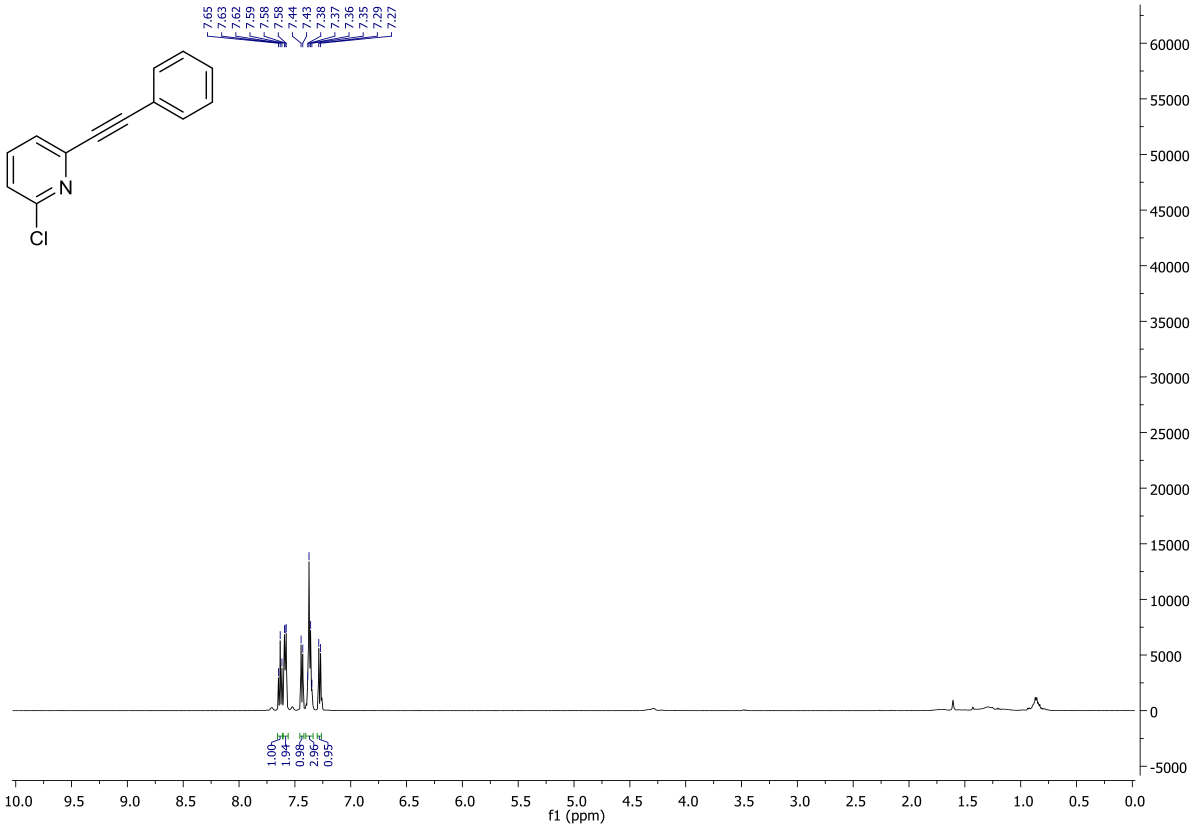

^{13}C NMR 3o

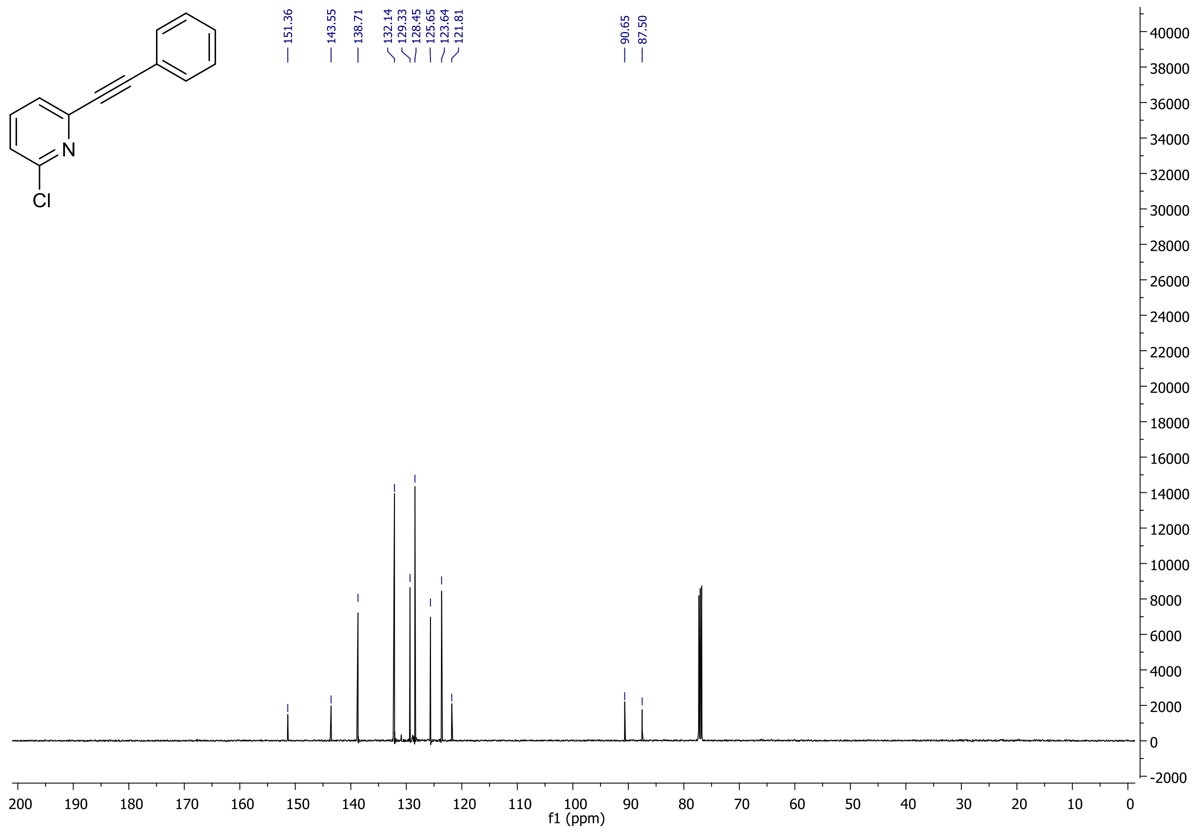

^{1}H NMR 3p

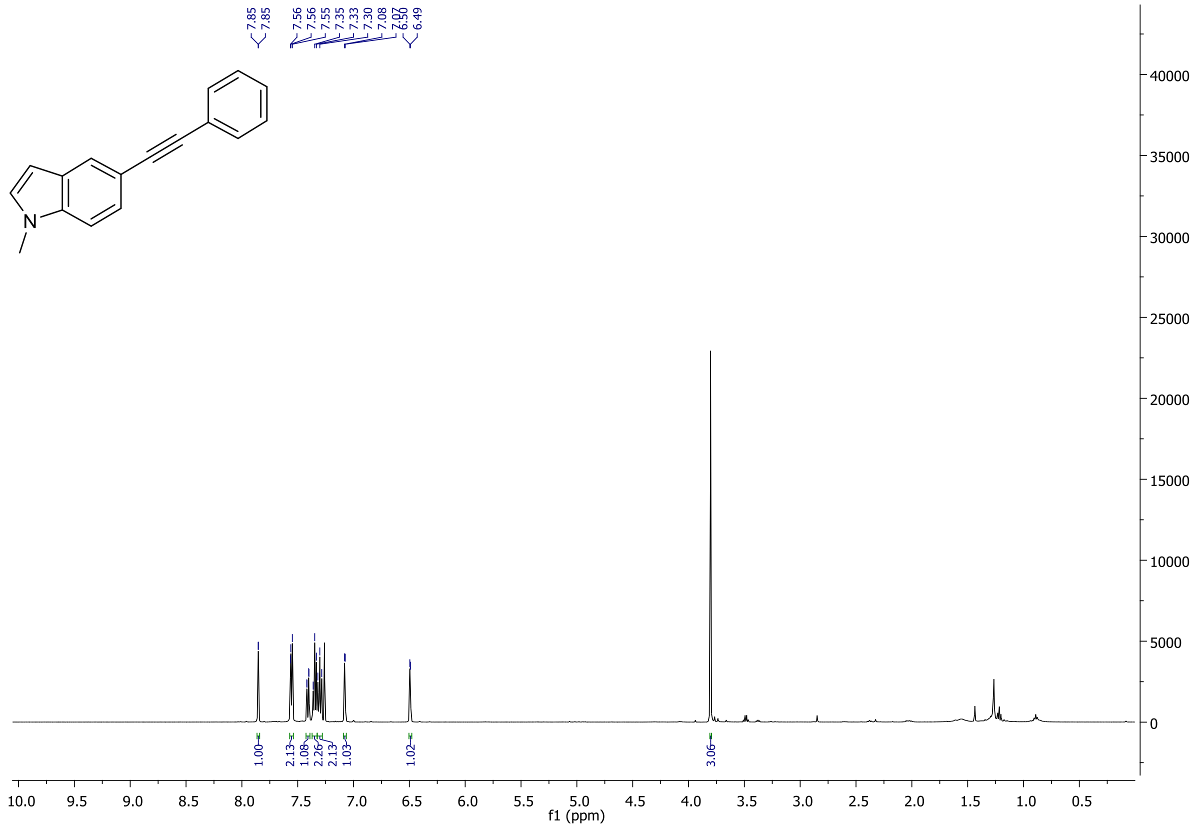

^{13}C NMR 3p

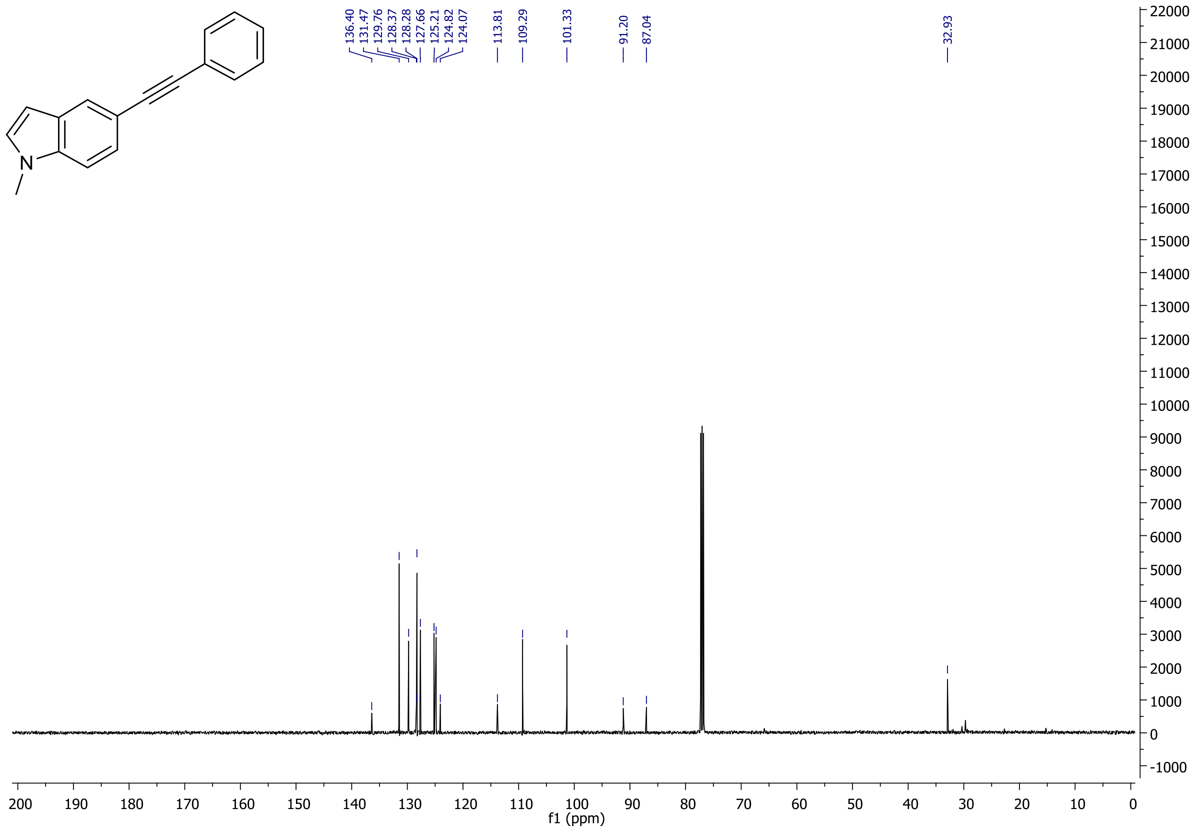

^{1}H NMR 3q

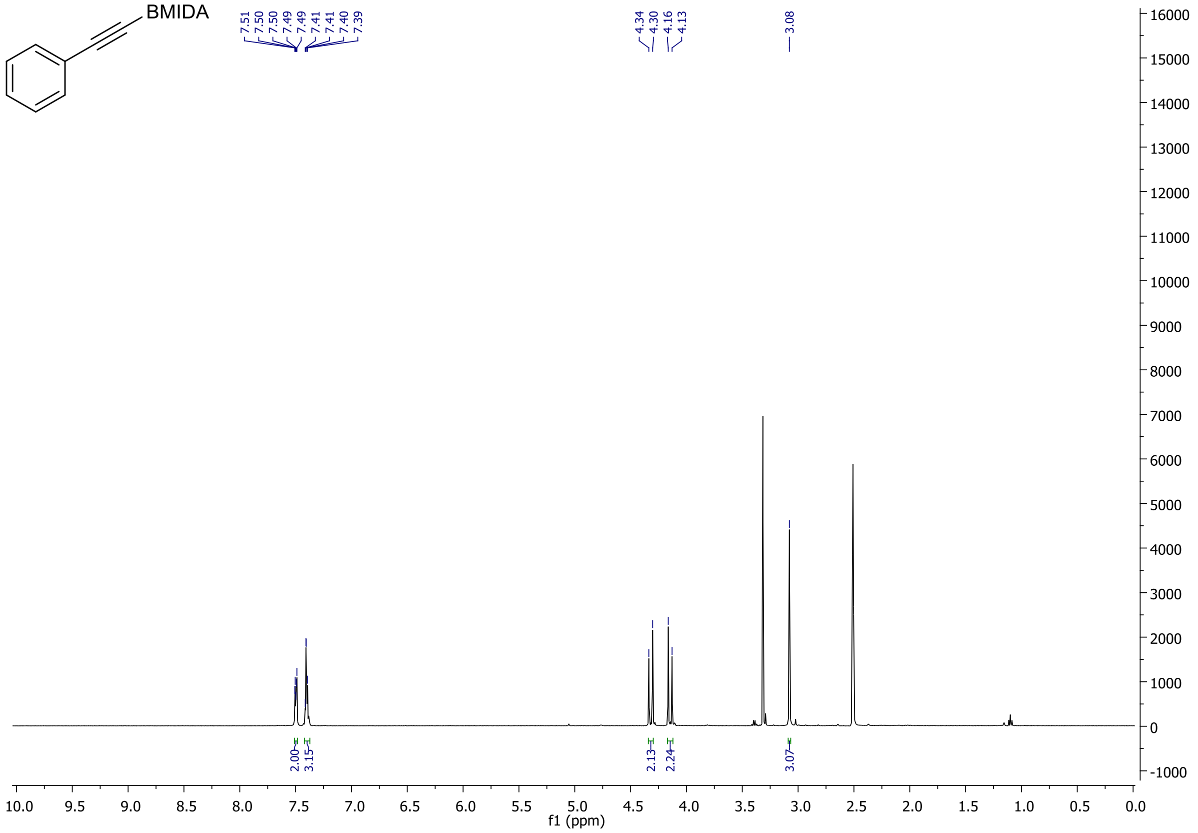

^{13}C NMR 3q

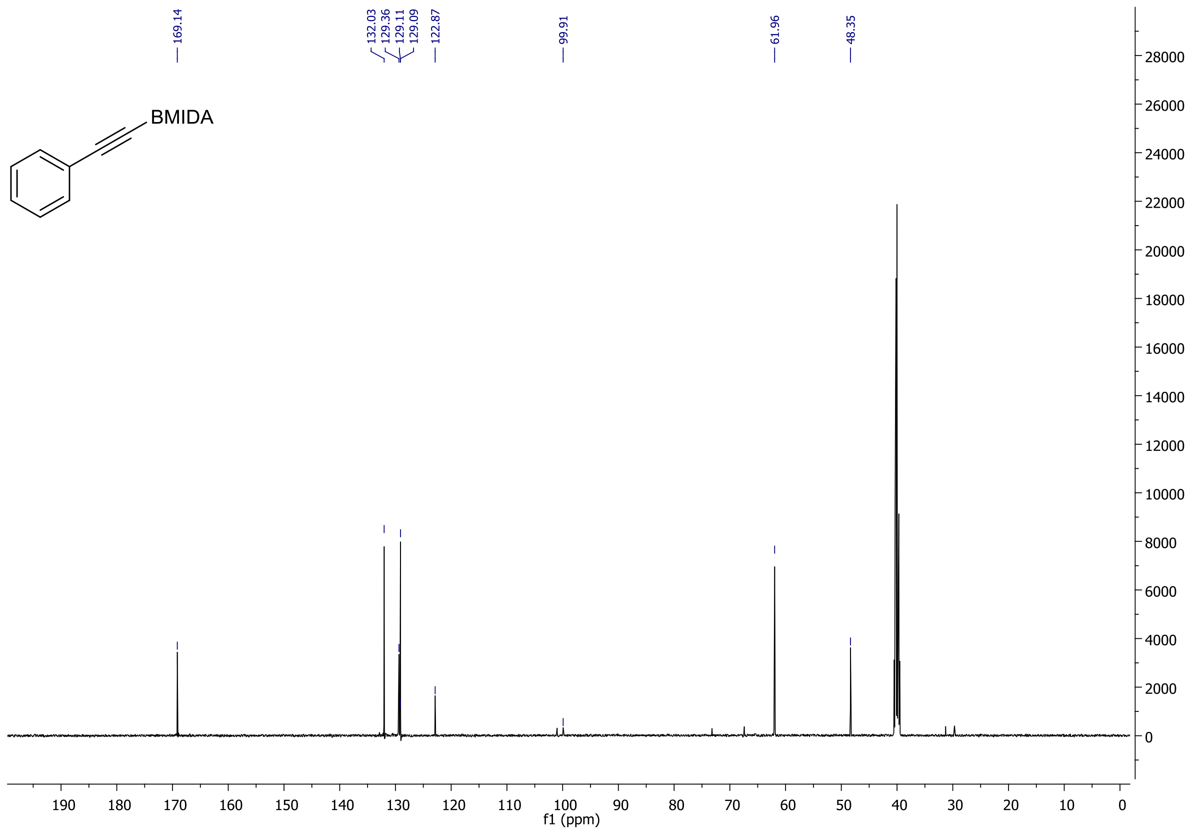

^{11}B NMR 3q

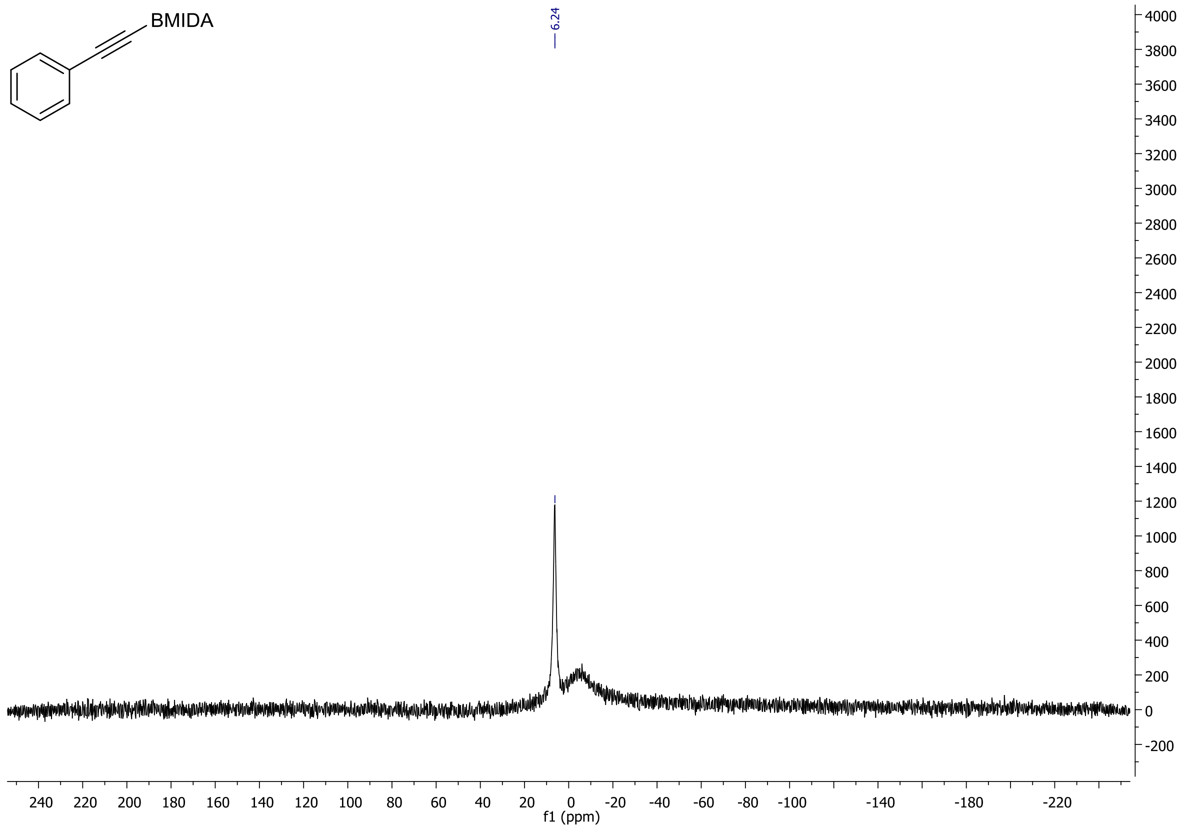

^{1}H NMR 3r

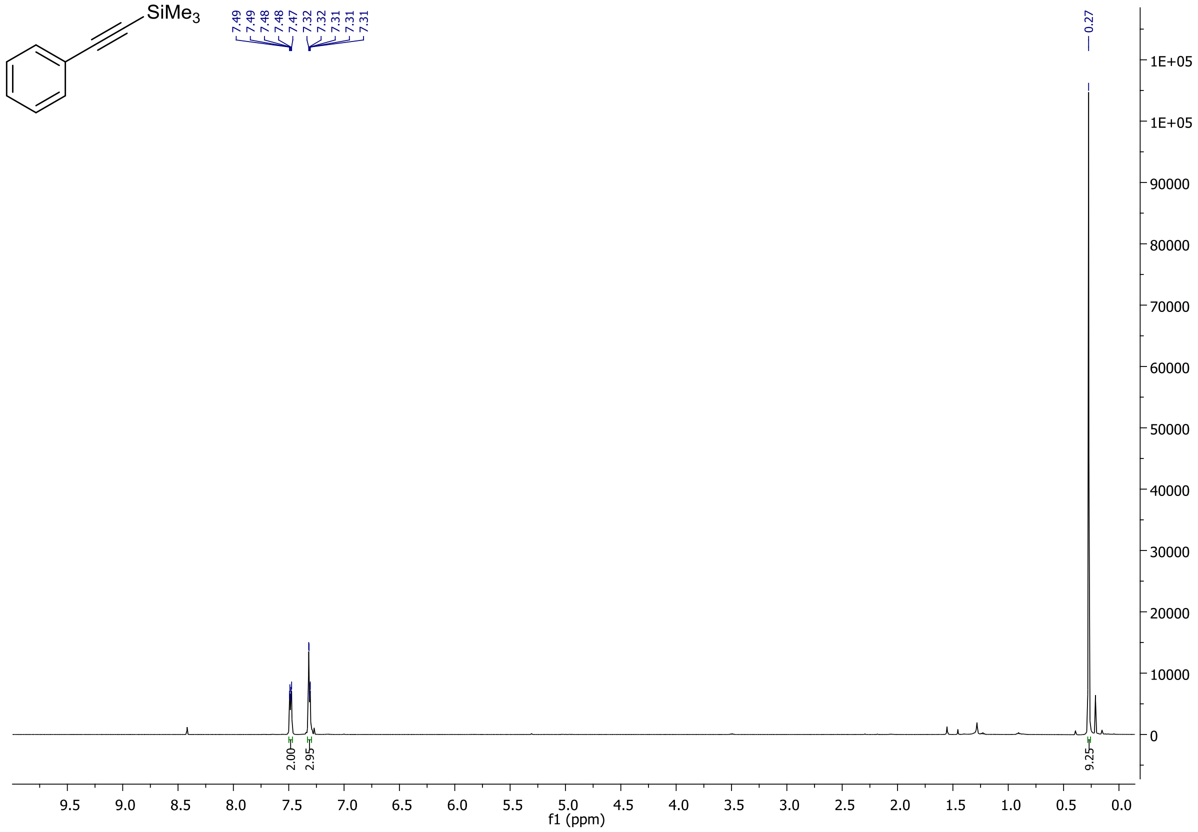

^{13}C NMR 3r

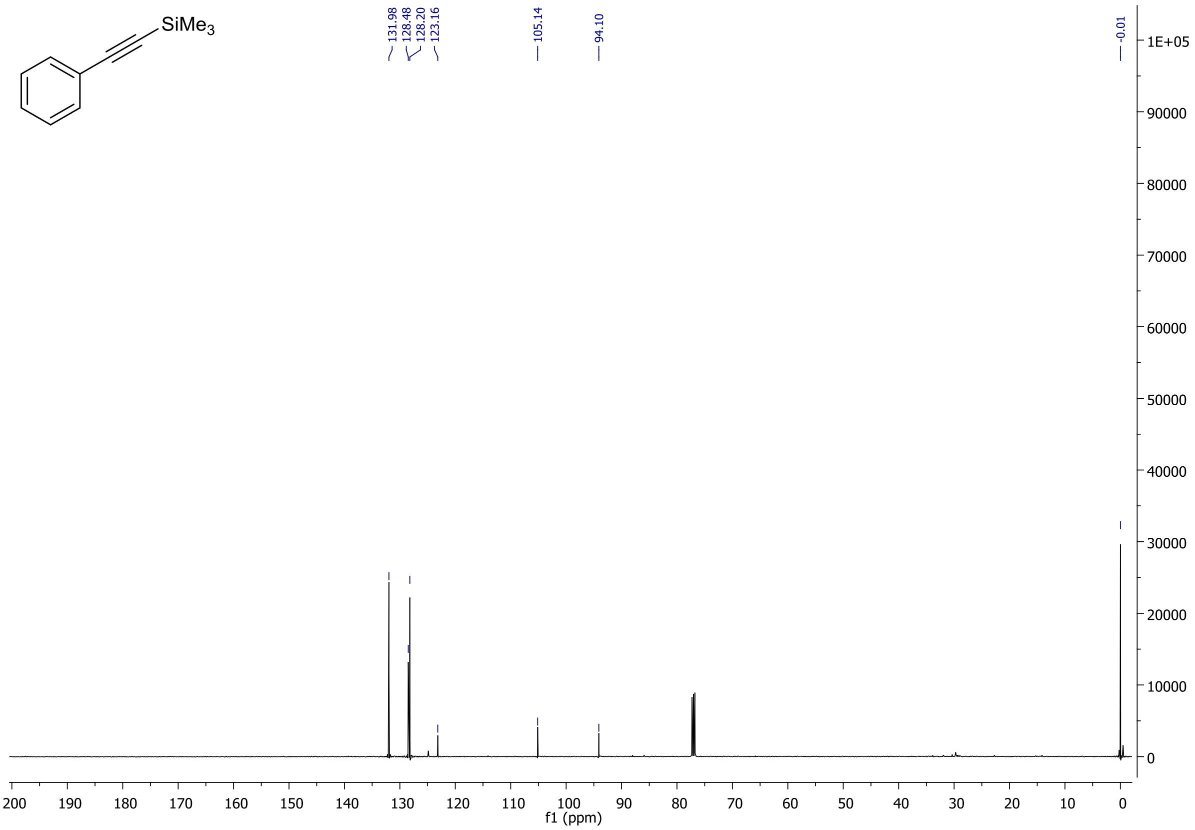

^{1}H NMR 3s

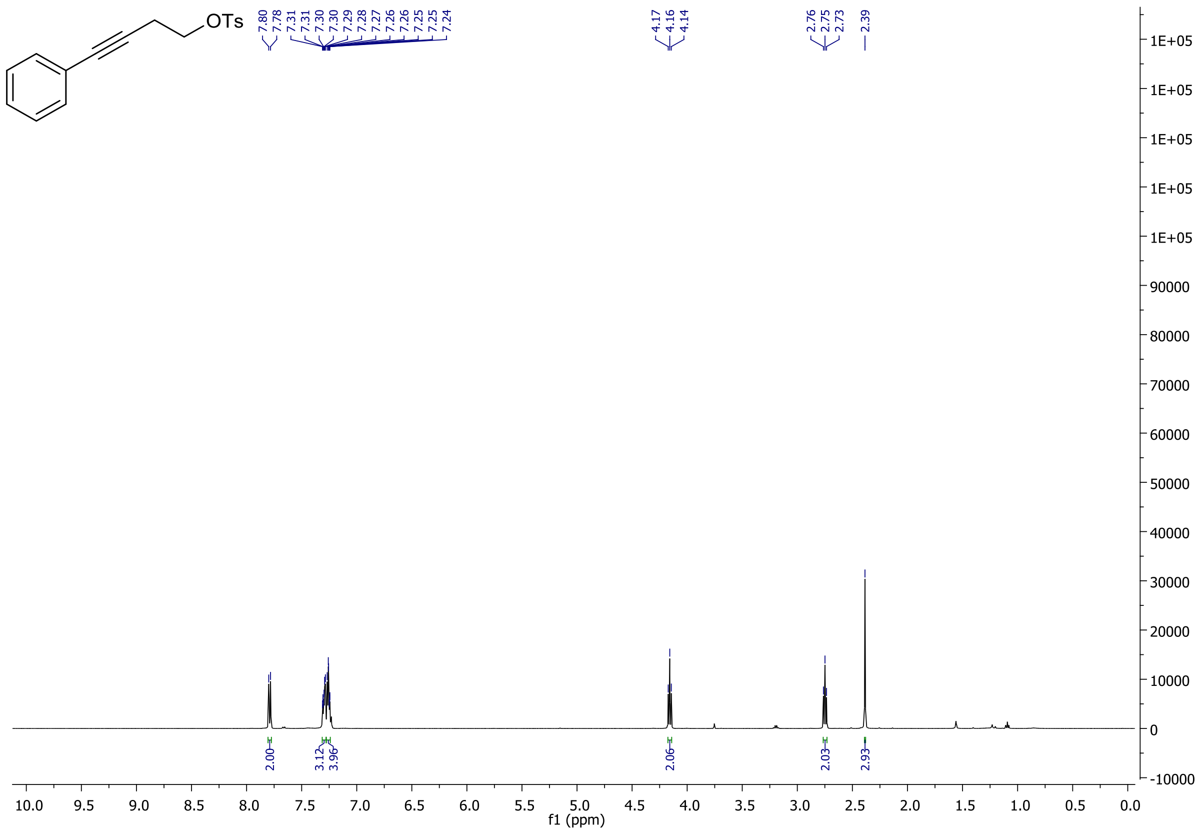

^{13}C NMR 3s

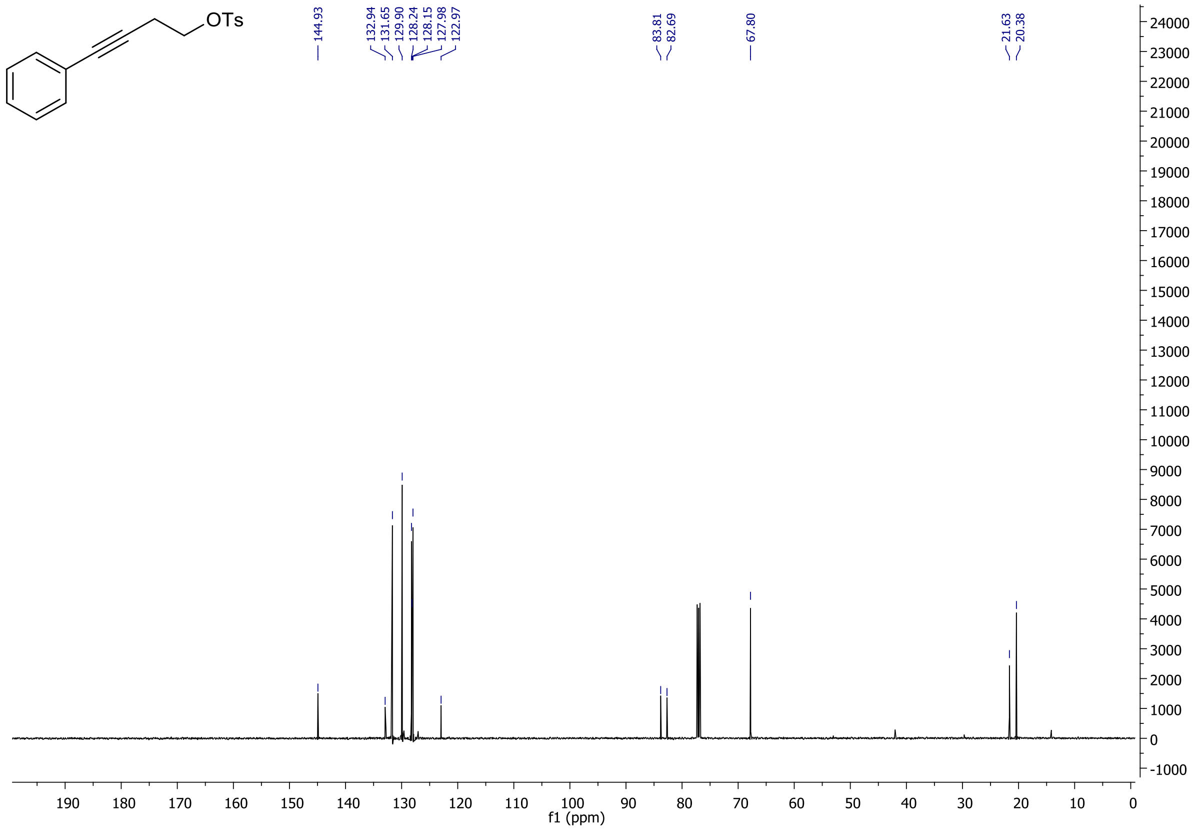

^{1}H NMR 3t

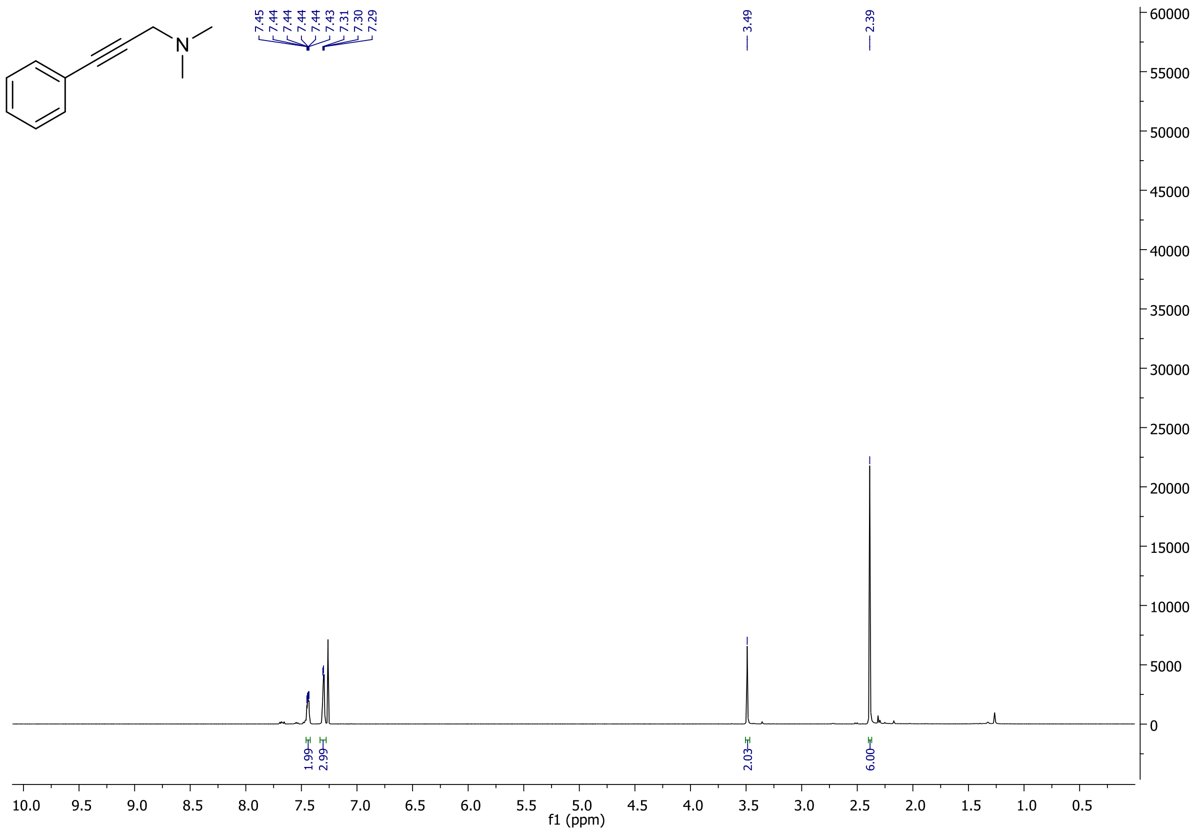

^{13}C NMR 3t

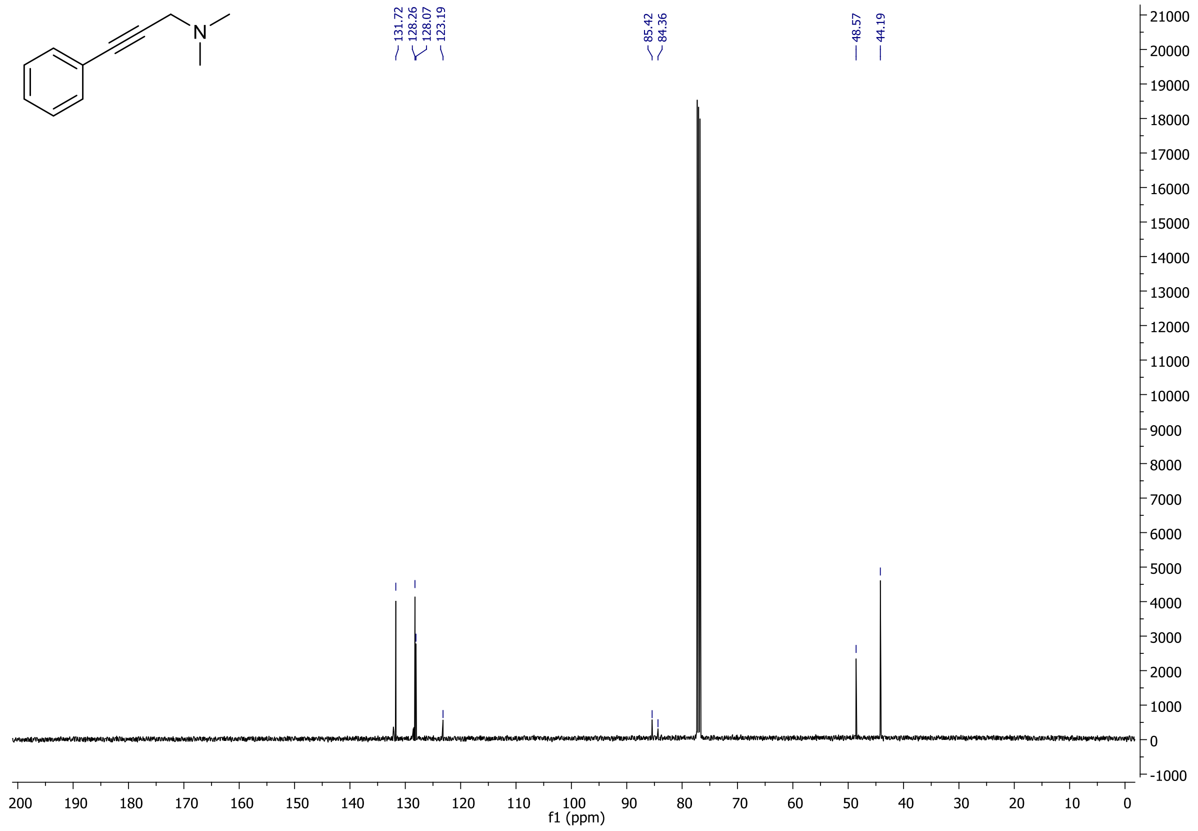

^{1}H NMR 3u

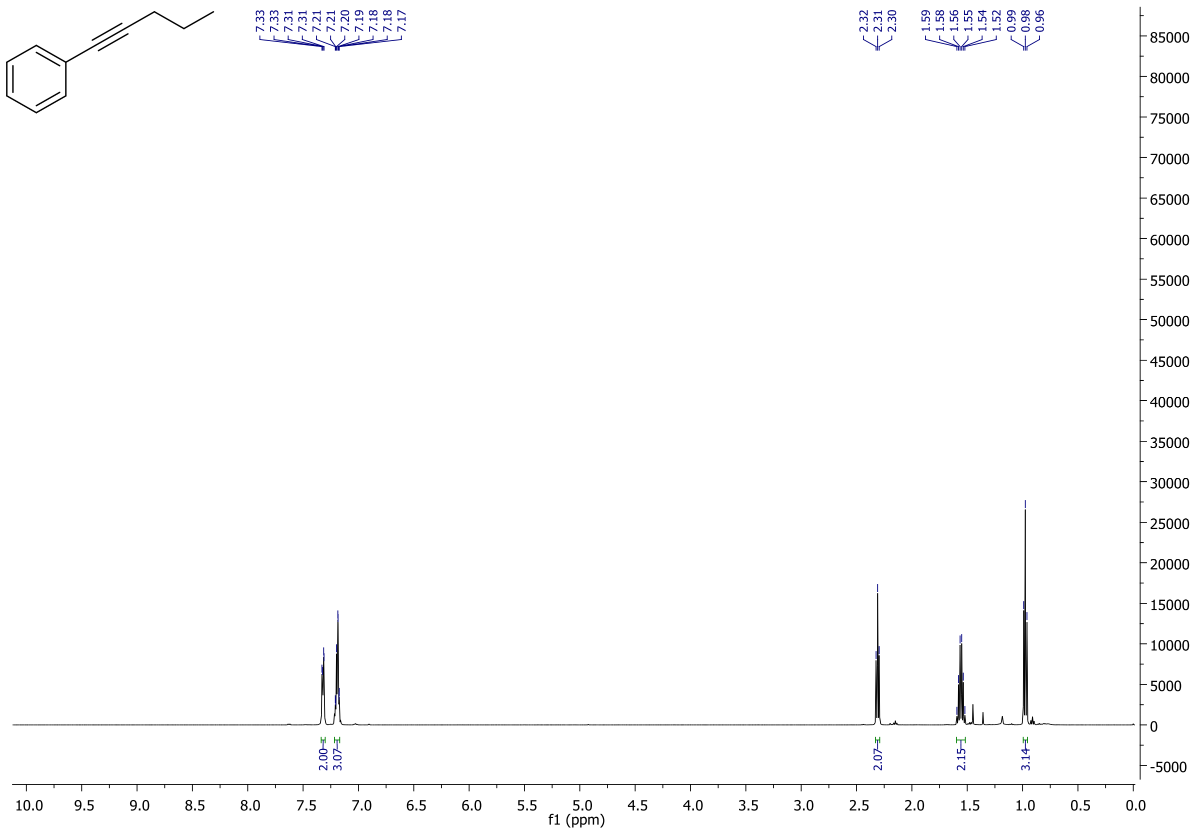

^{13}C NMR 3u

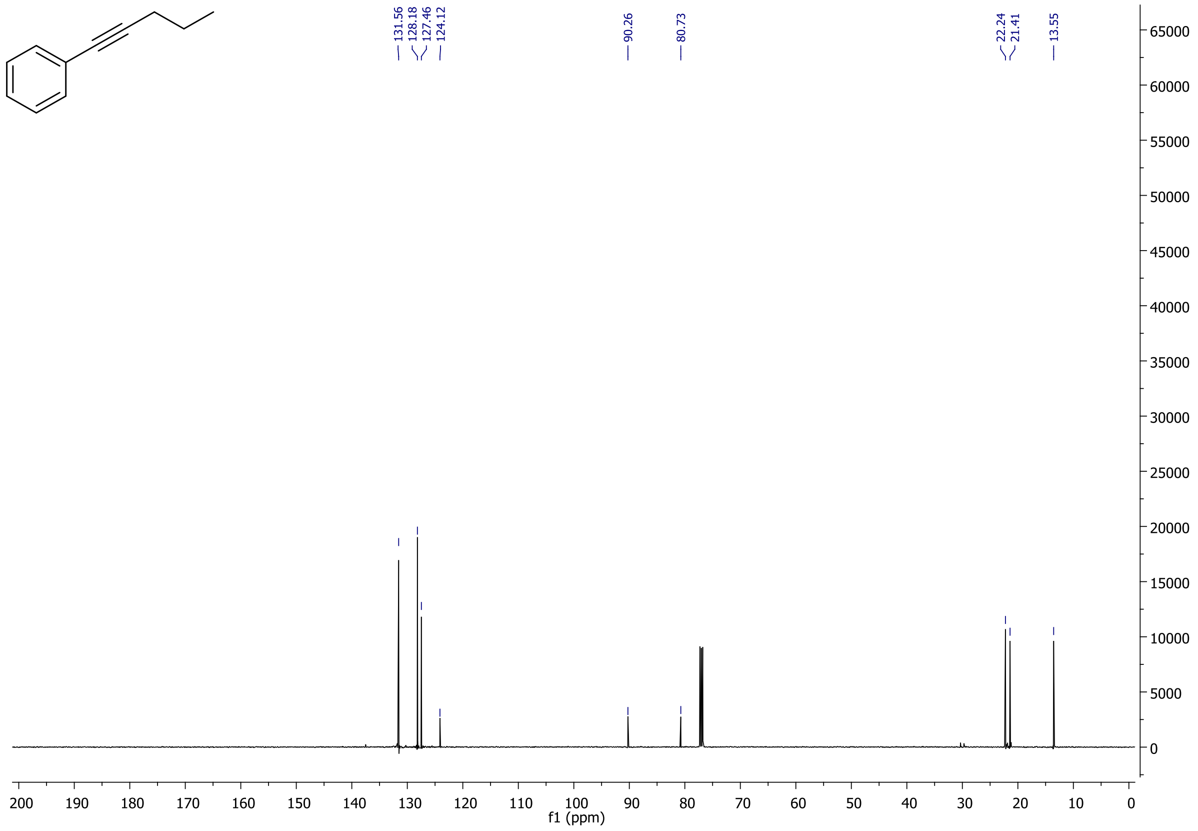

^1H NMR 3v

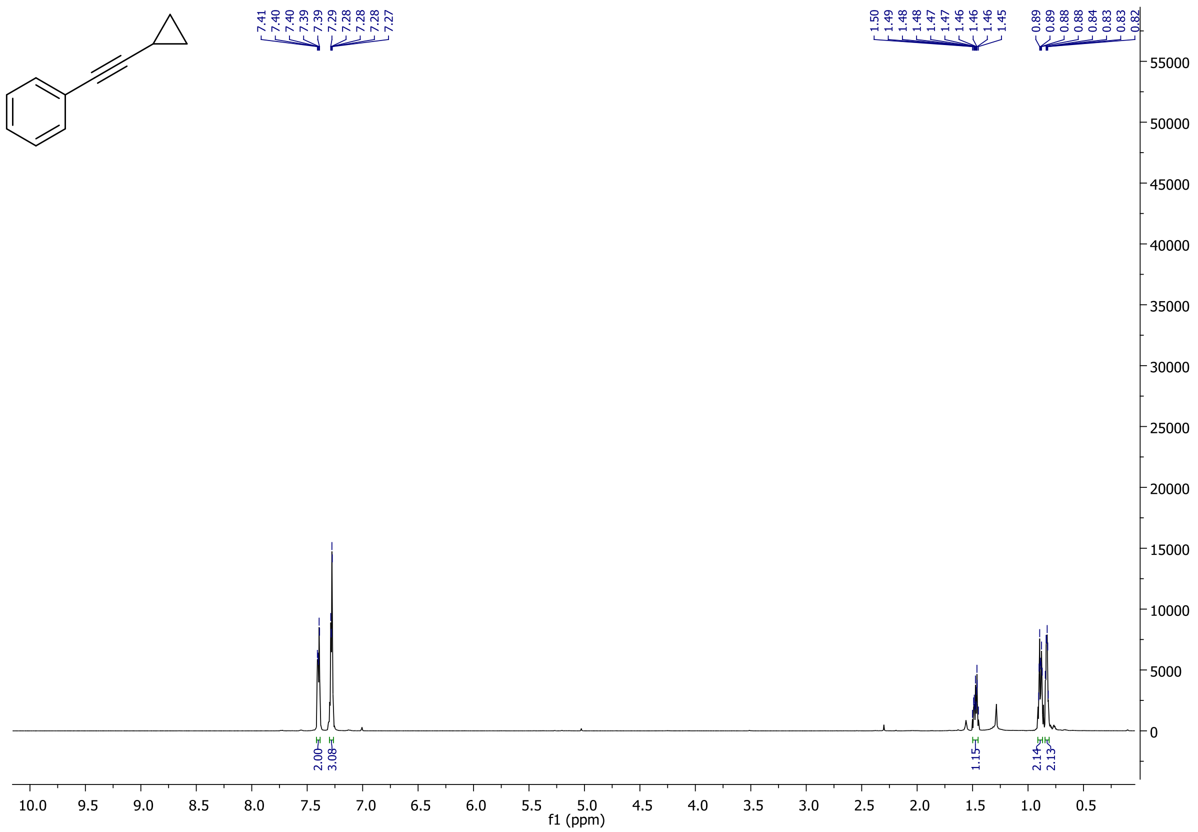

^{13}C NMR 3v

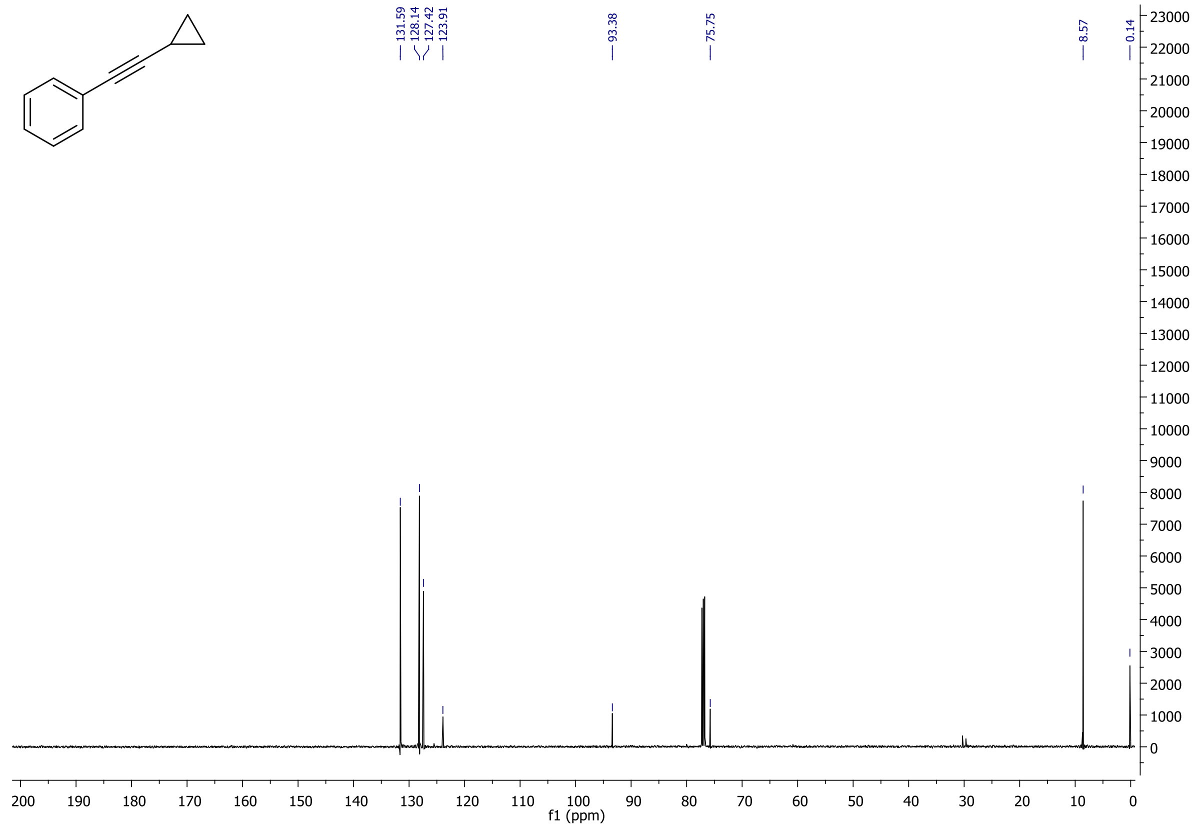

^{1}H NMR 3w

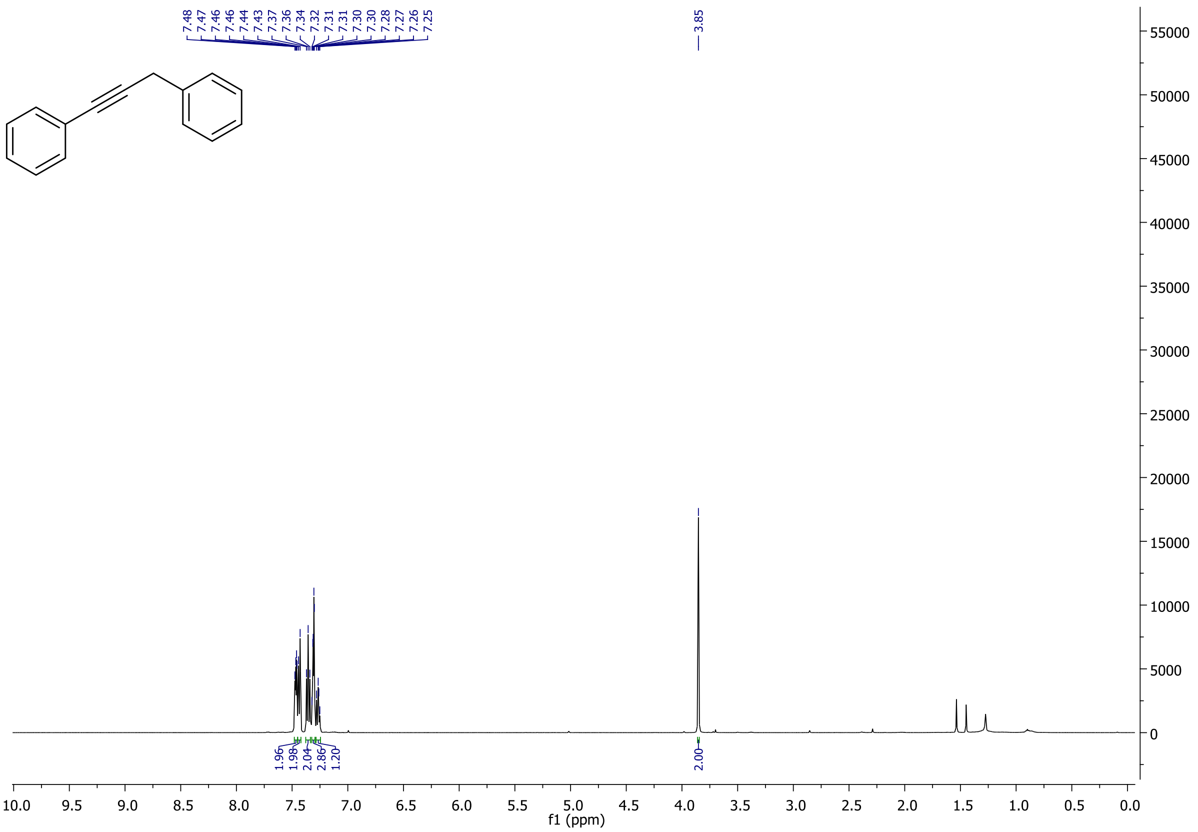

^{13}C NMR 3w

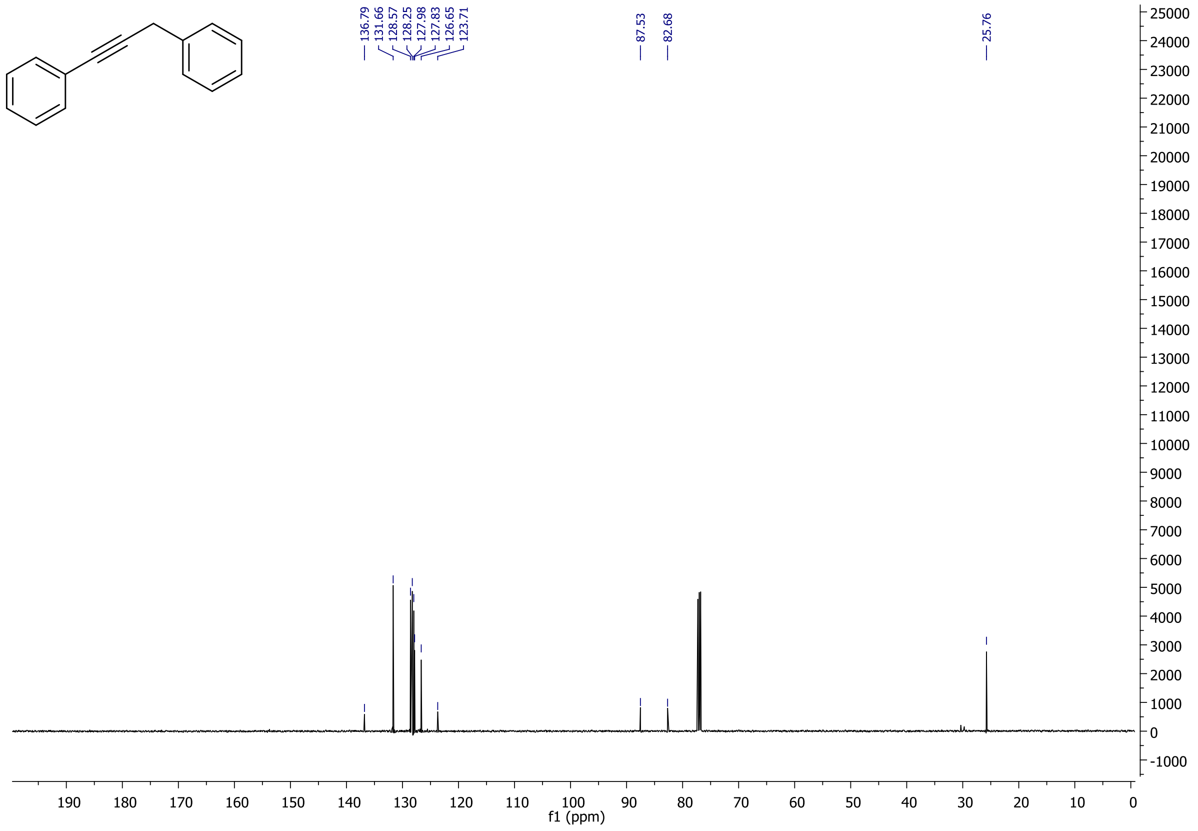

^{1}H NMR 3x

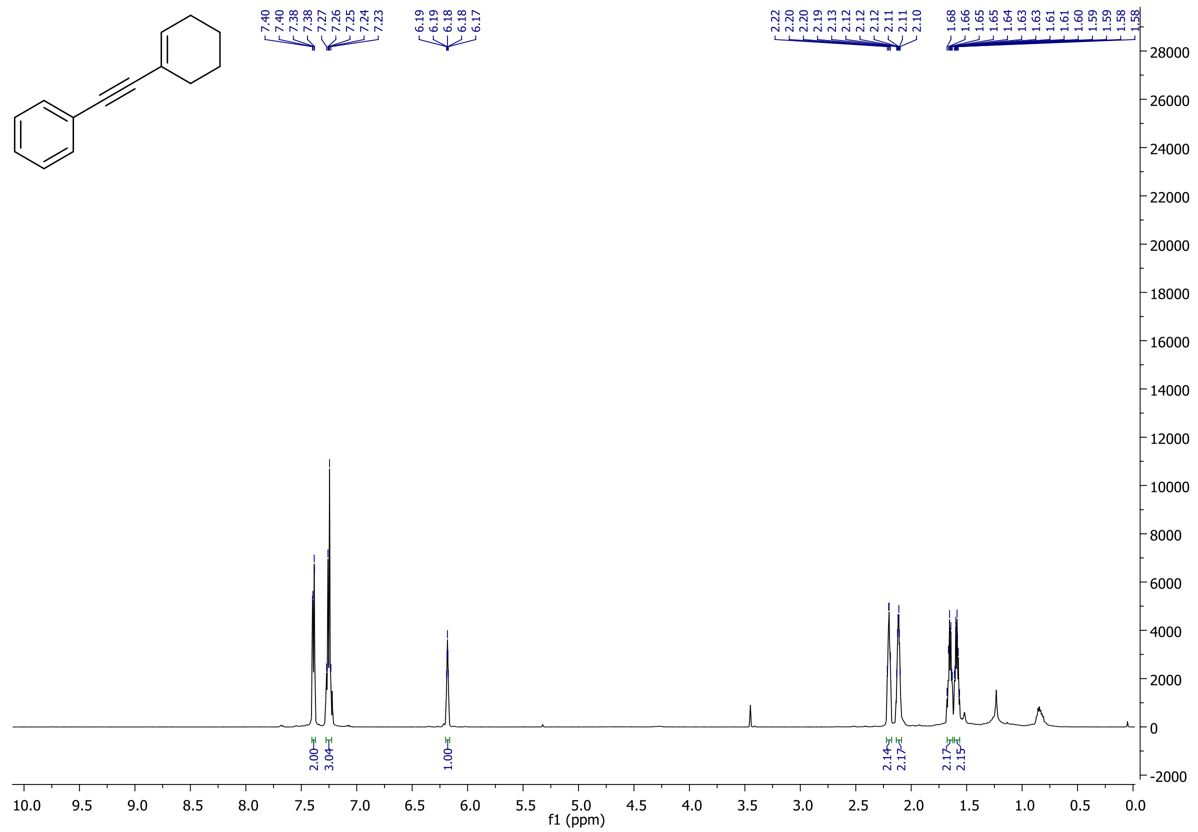

^{13}C NMR 3x

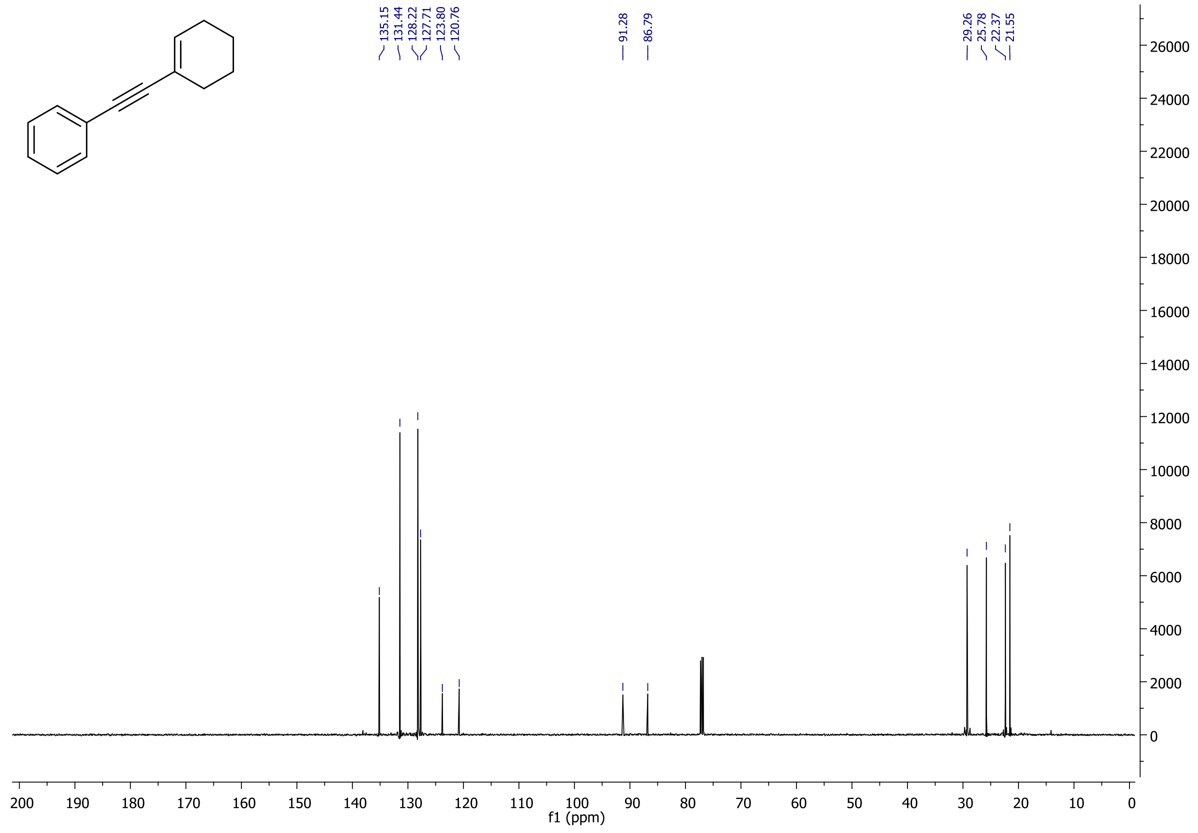

^{1}H NMR 3y

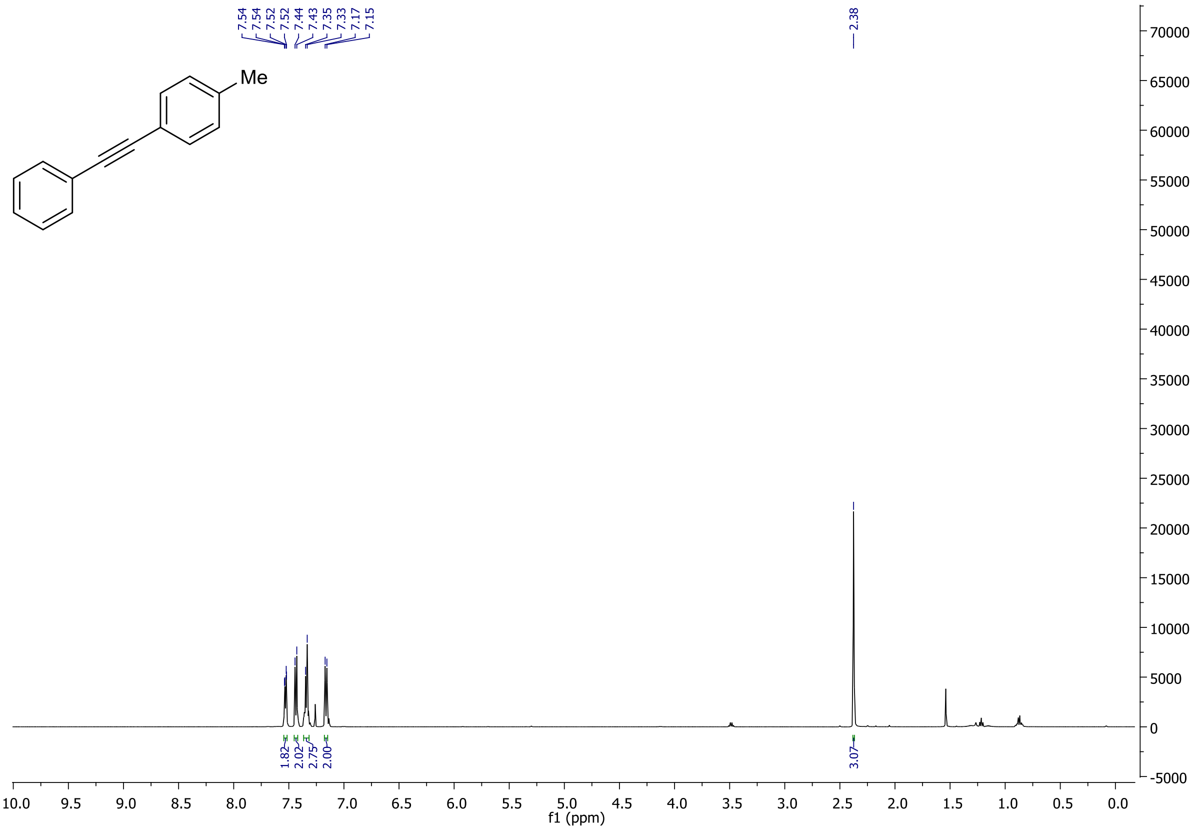

^{13}C NMR 3y

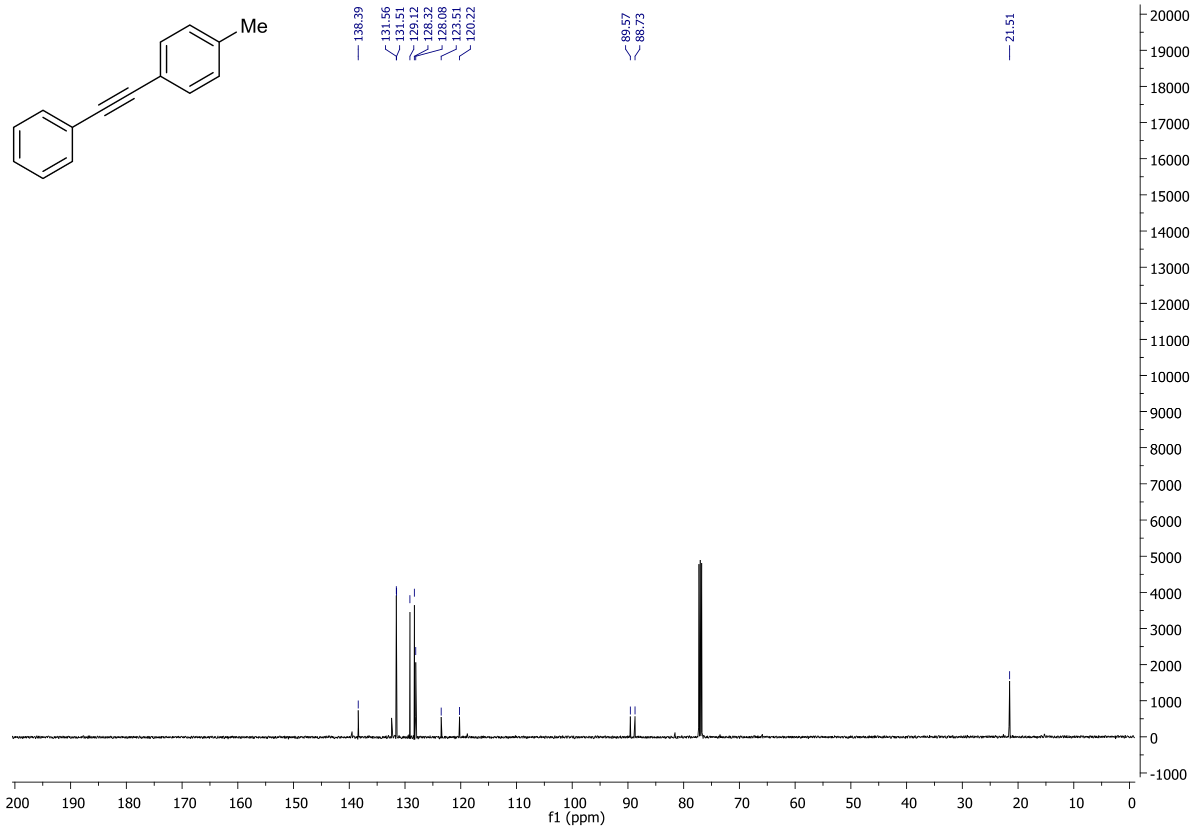

^{1}H NMR 3z

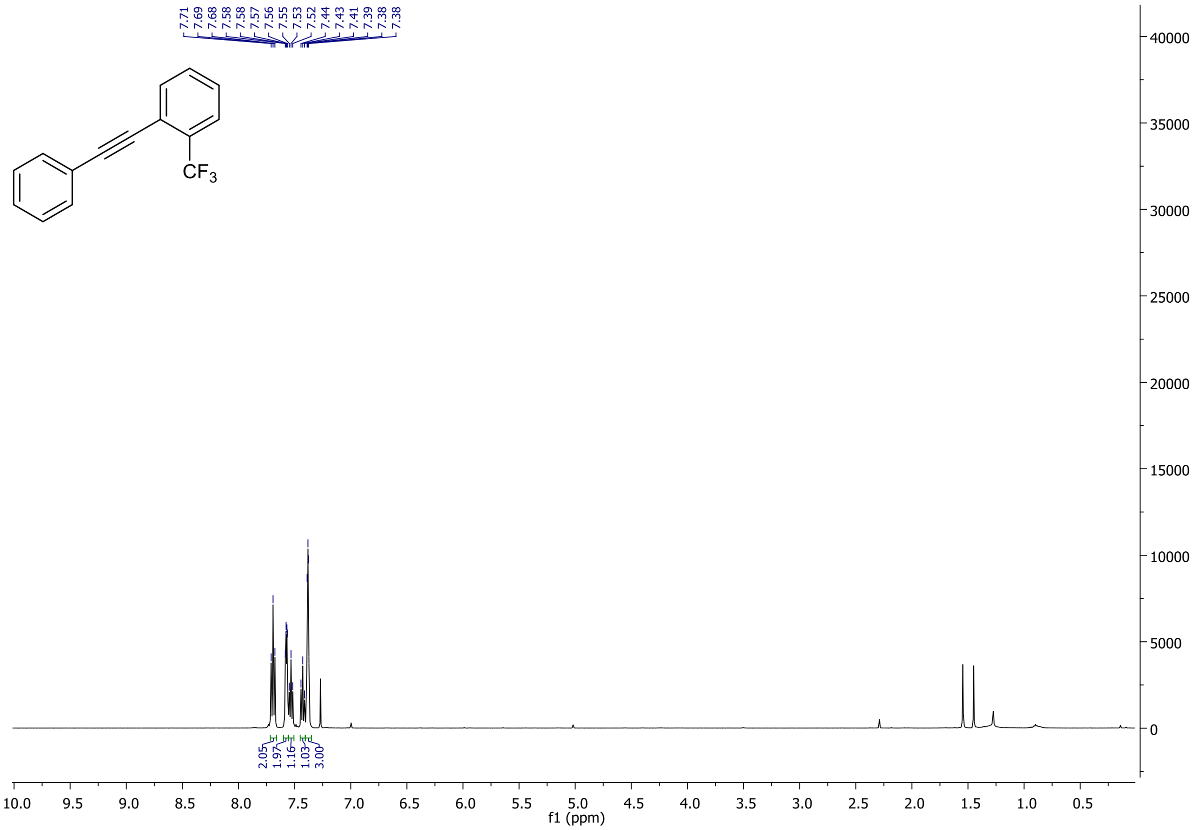

^{13}C NMR 3z

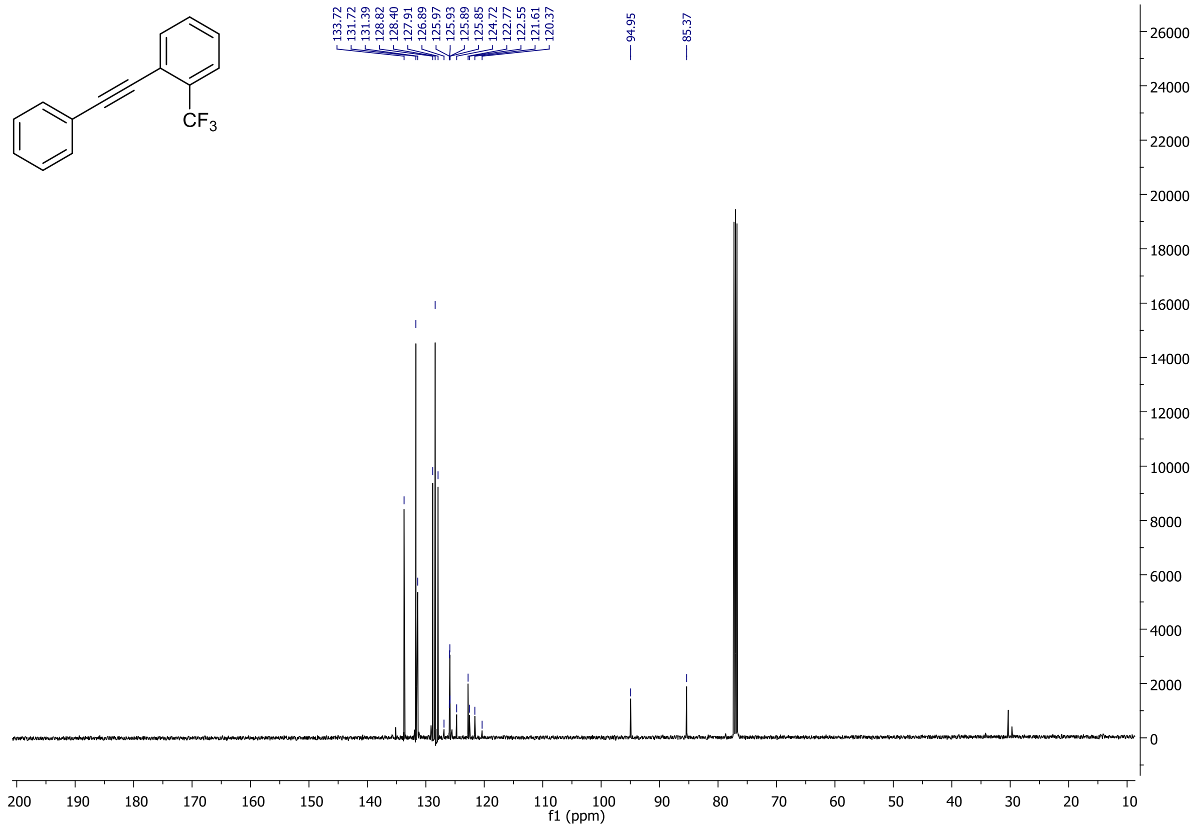

^{19}F NMR 3z

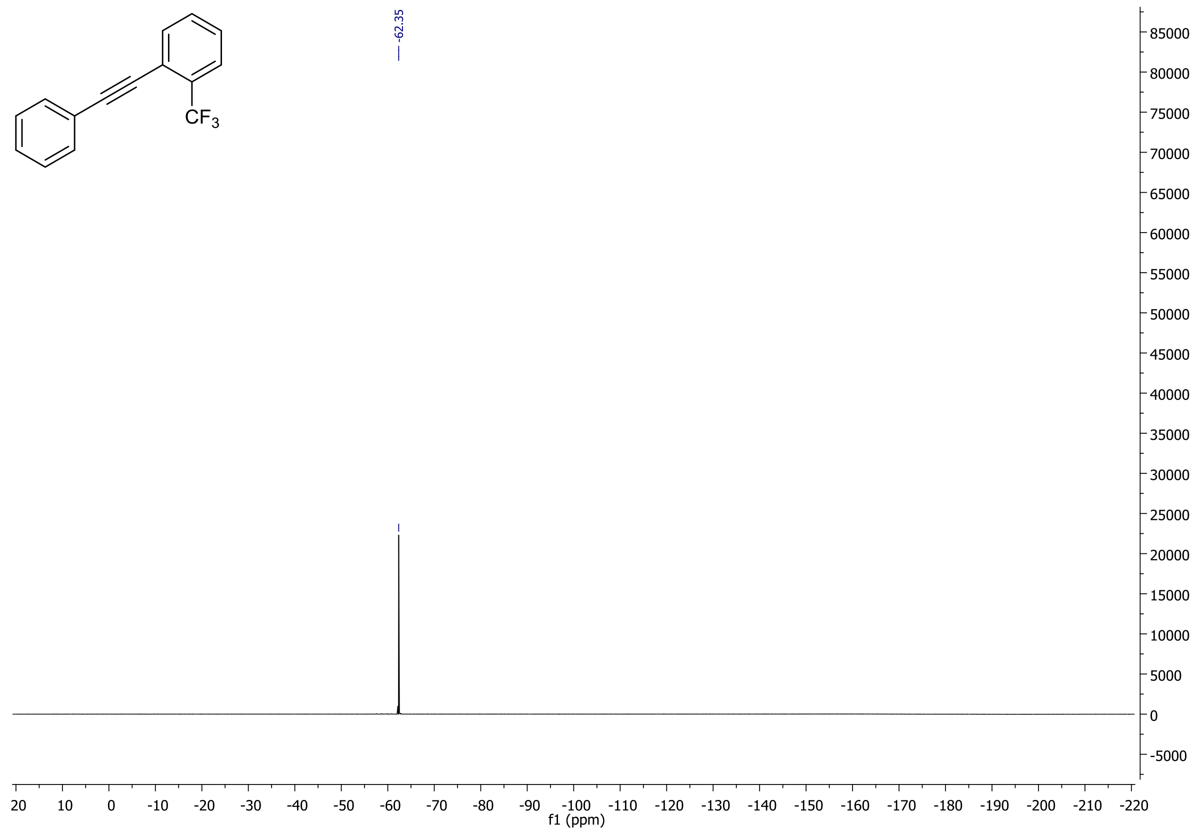

^{1}H NMR 3aa

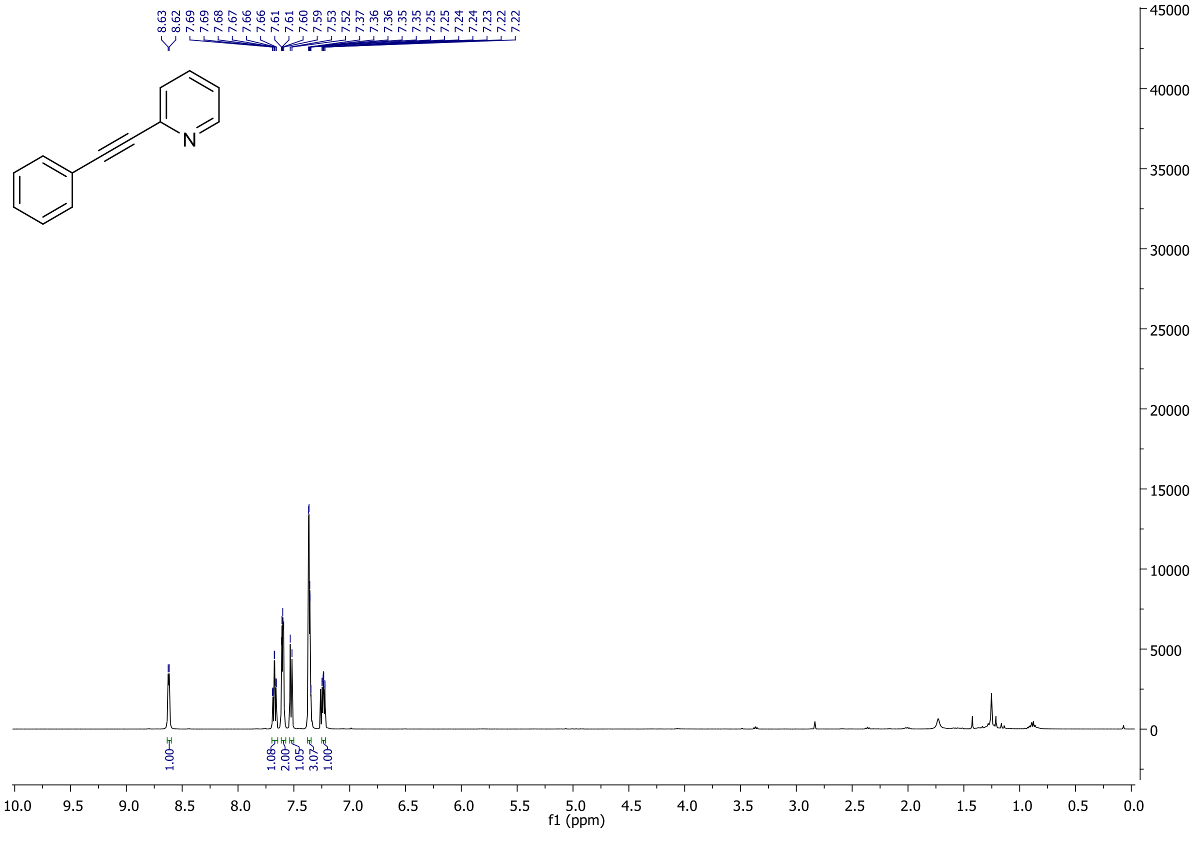

^{13}C NMR 3aa

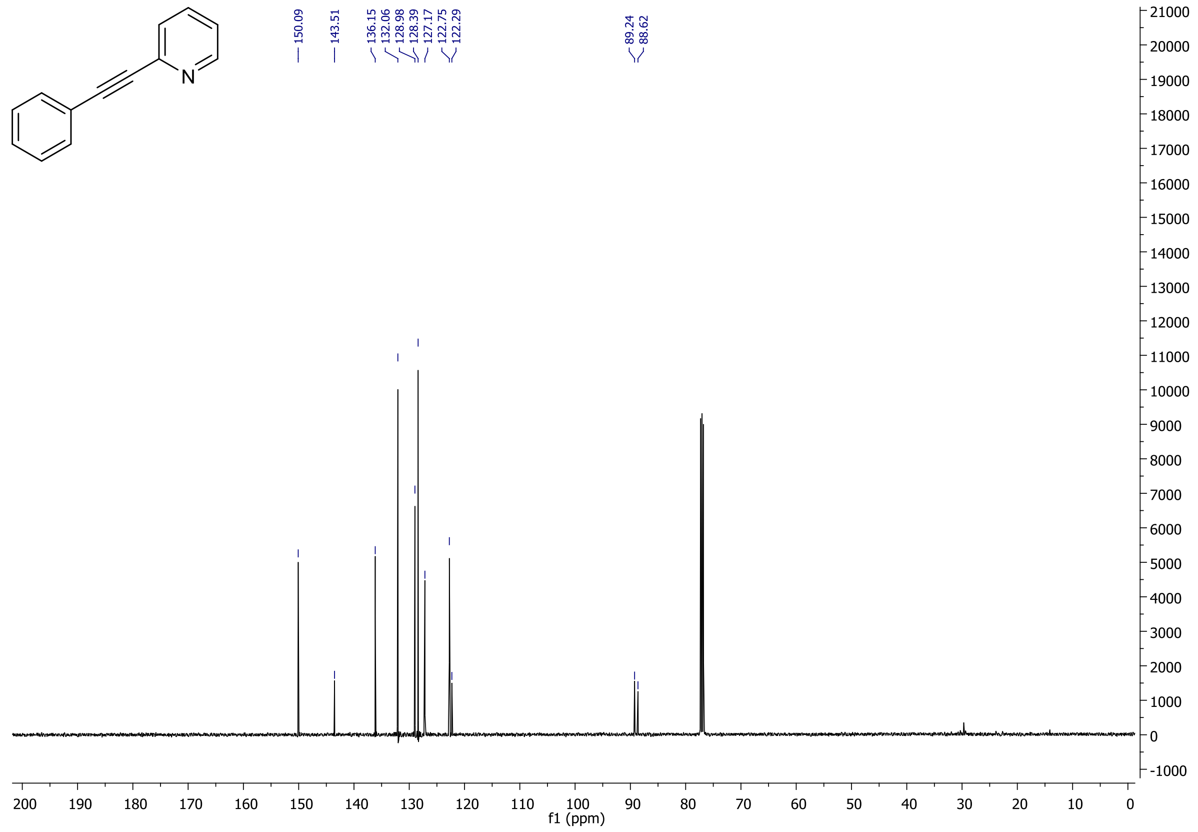

^{1}H NMR 3ab

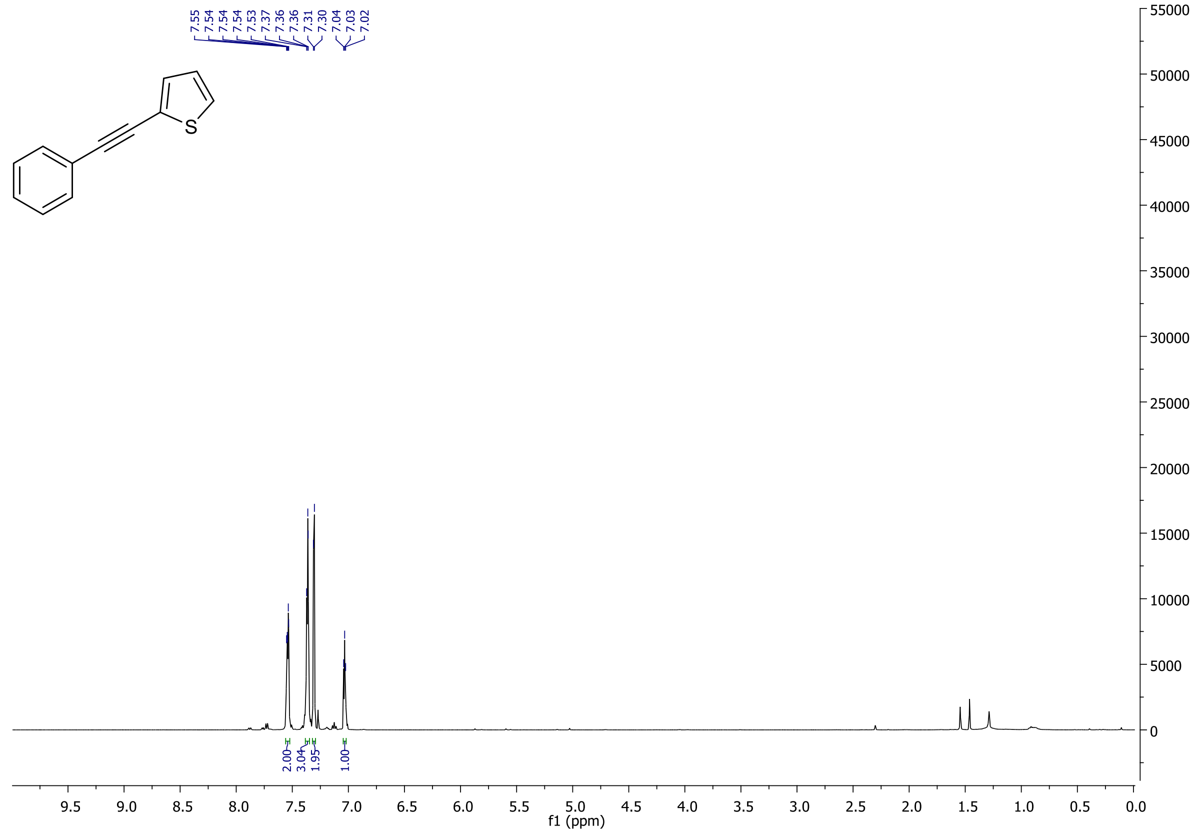

^{13}C NMR 3ab

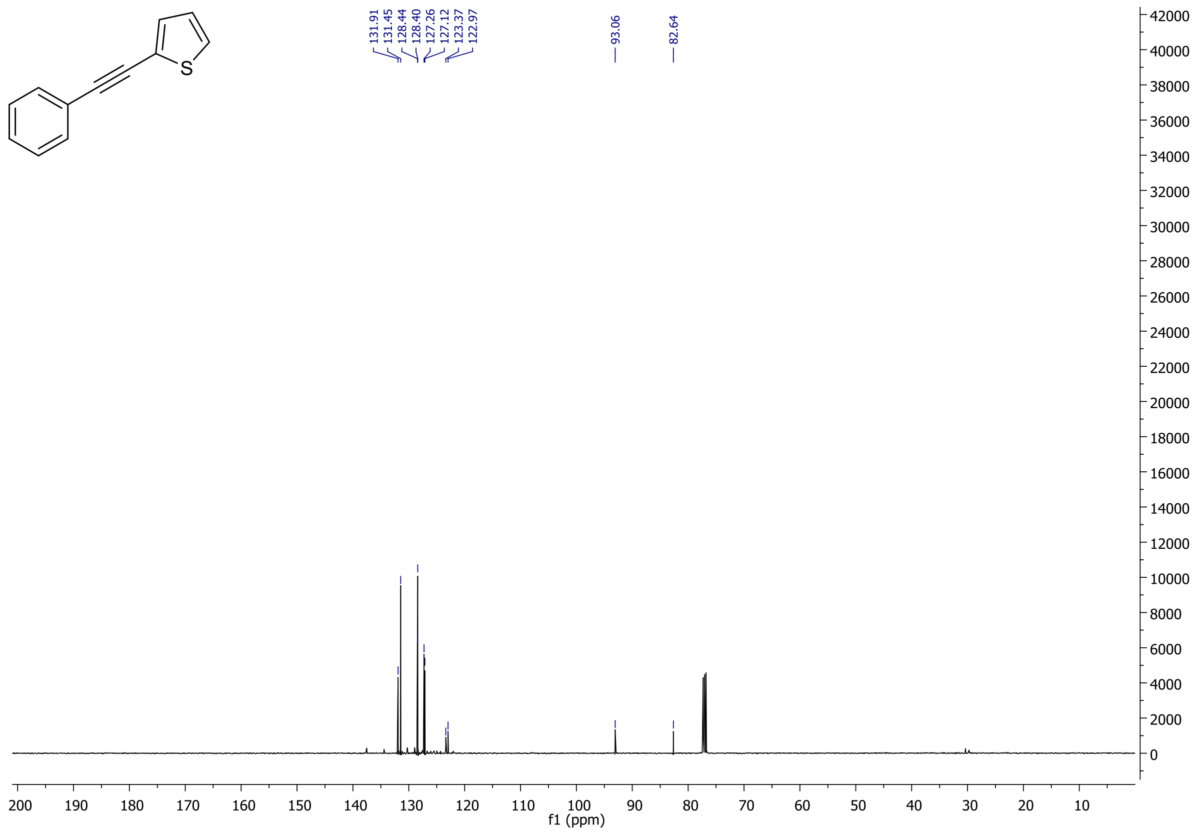

^{1}H NMR 3ac

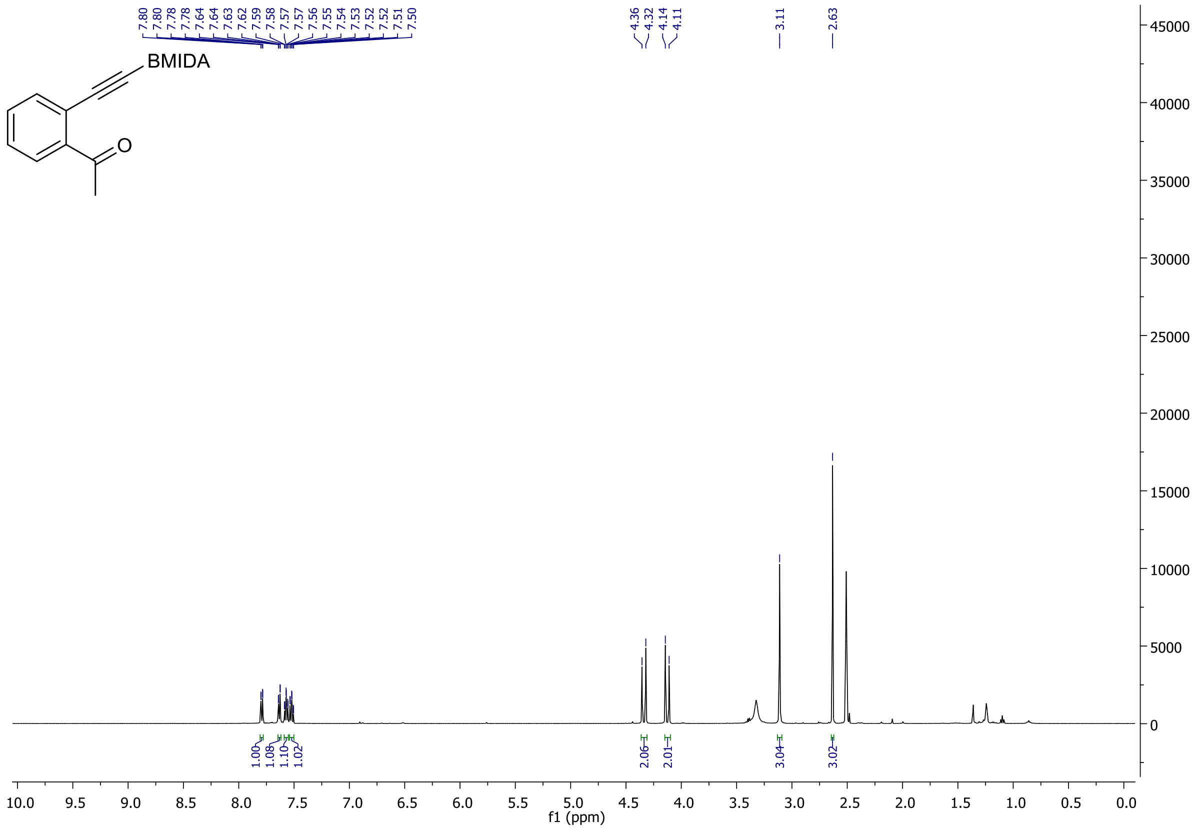

^{13}C NMR 3ac

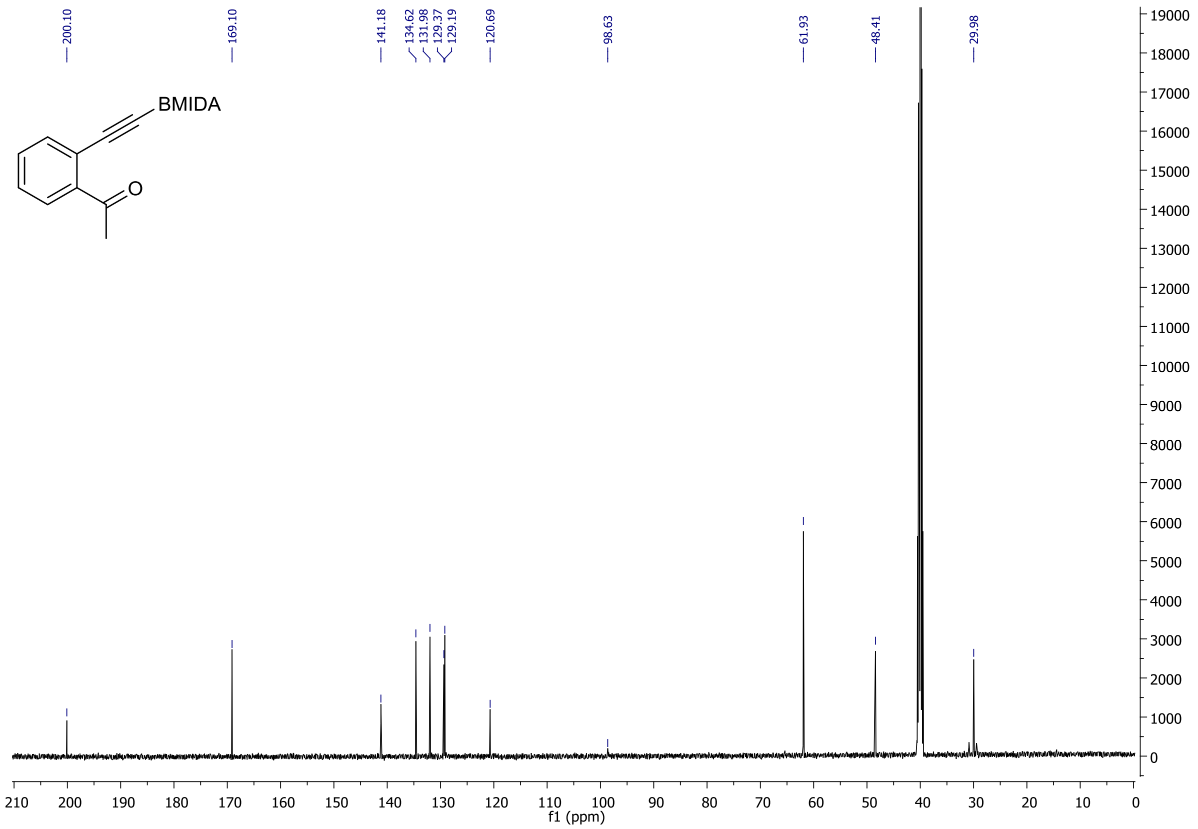

^{11}B NMR 3ac

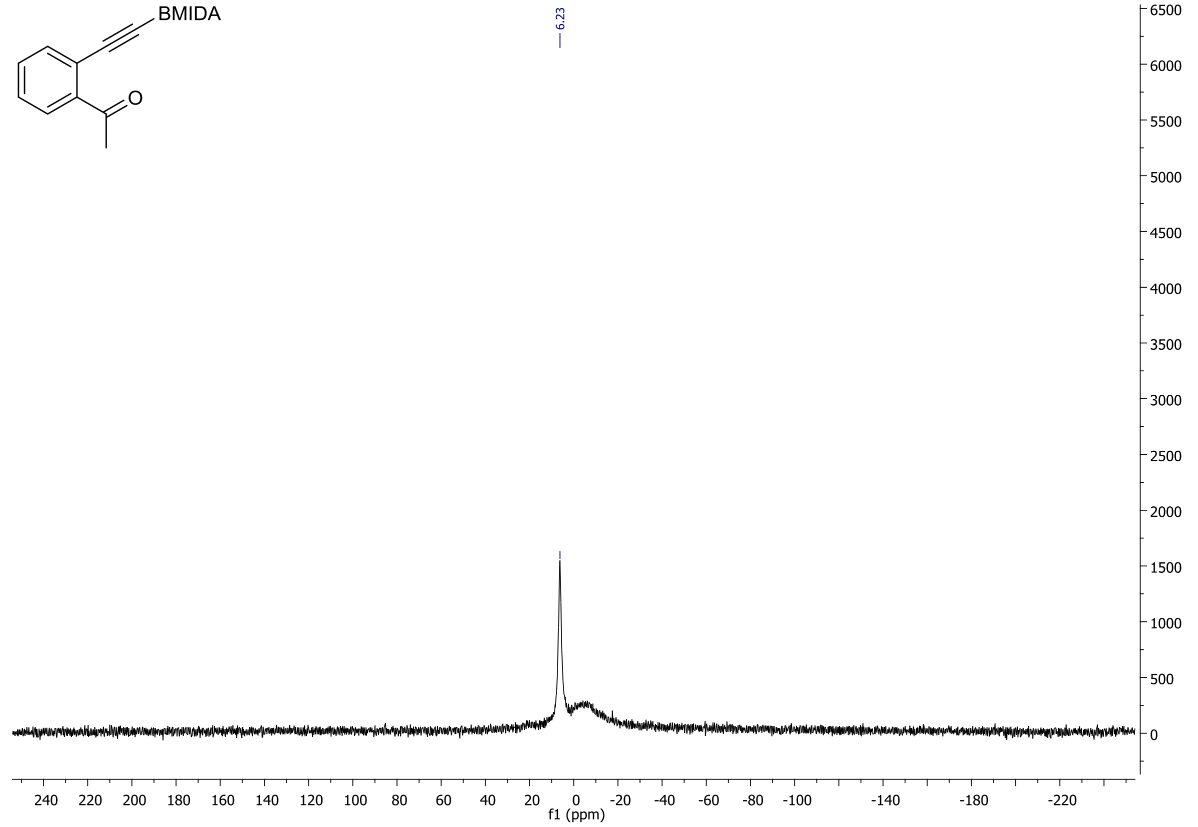

^{1}H NMR 3ad

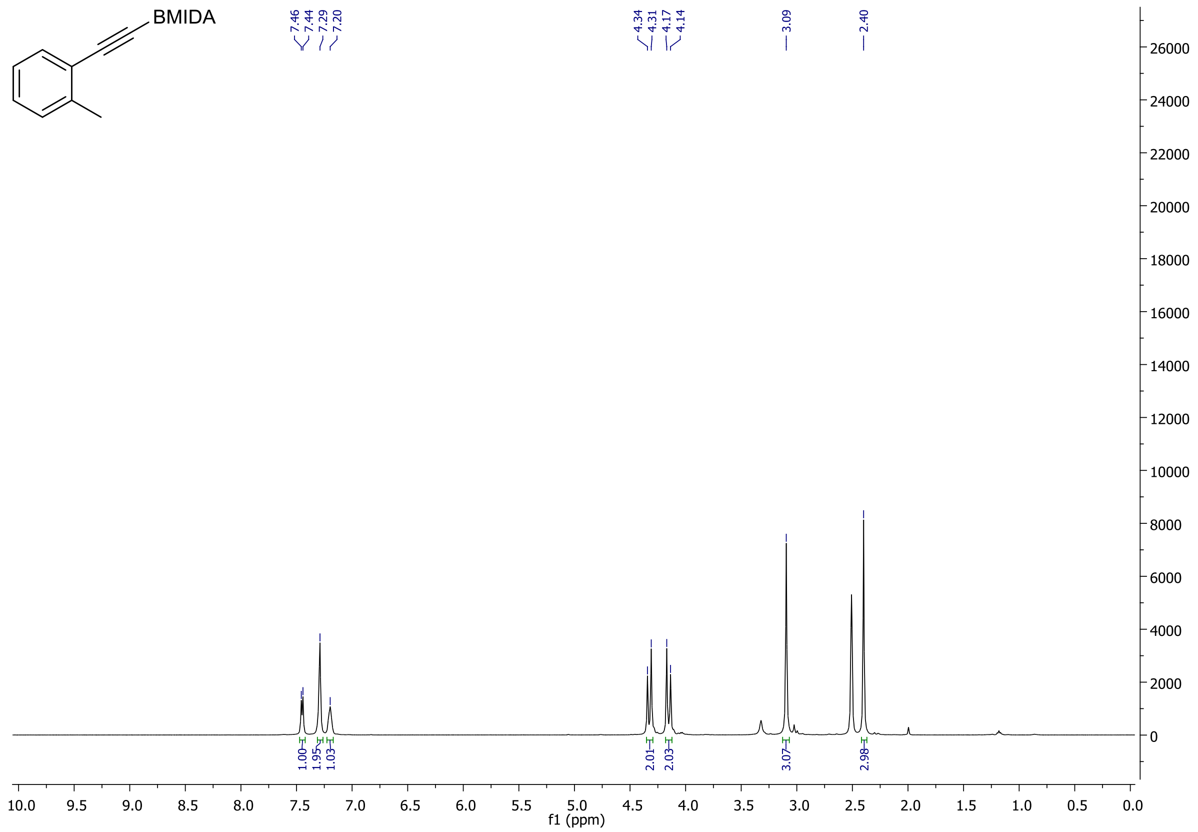

^{13}C NMR 3ad

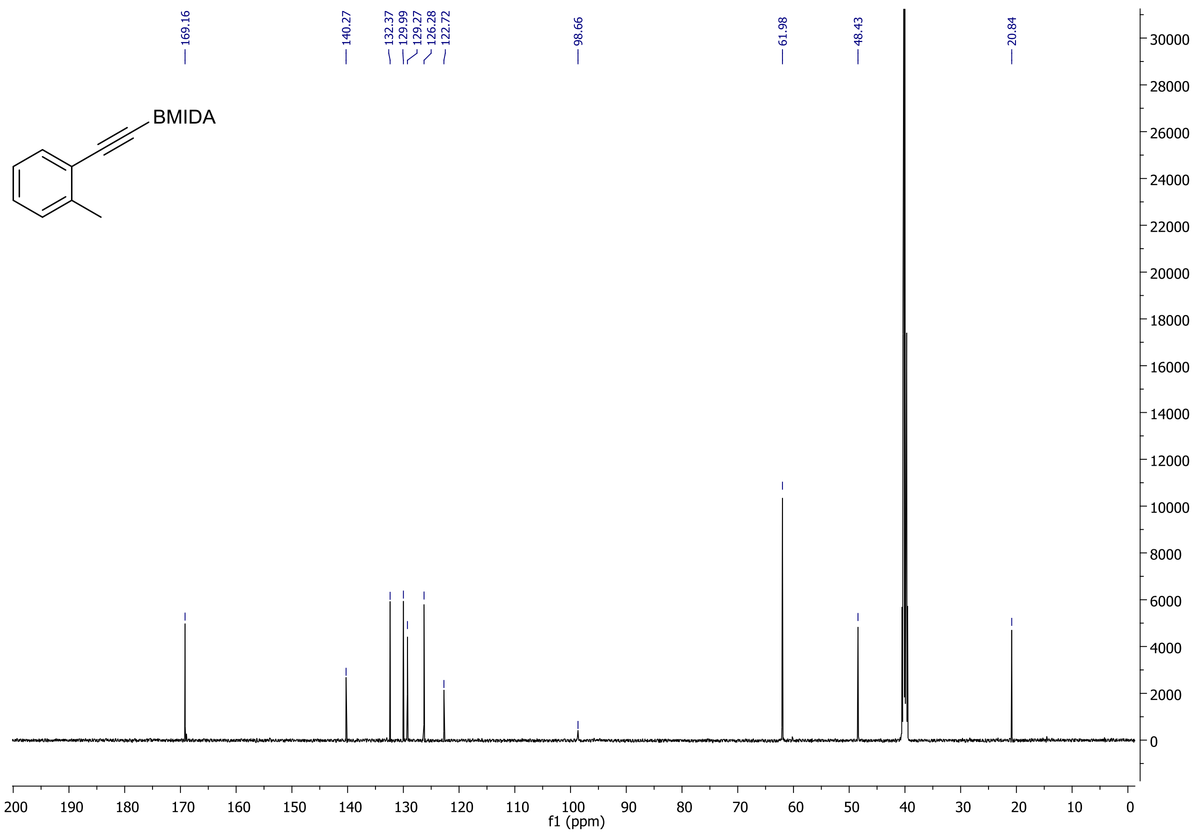

^{11}B NMR 3ad

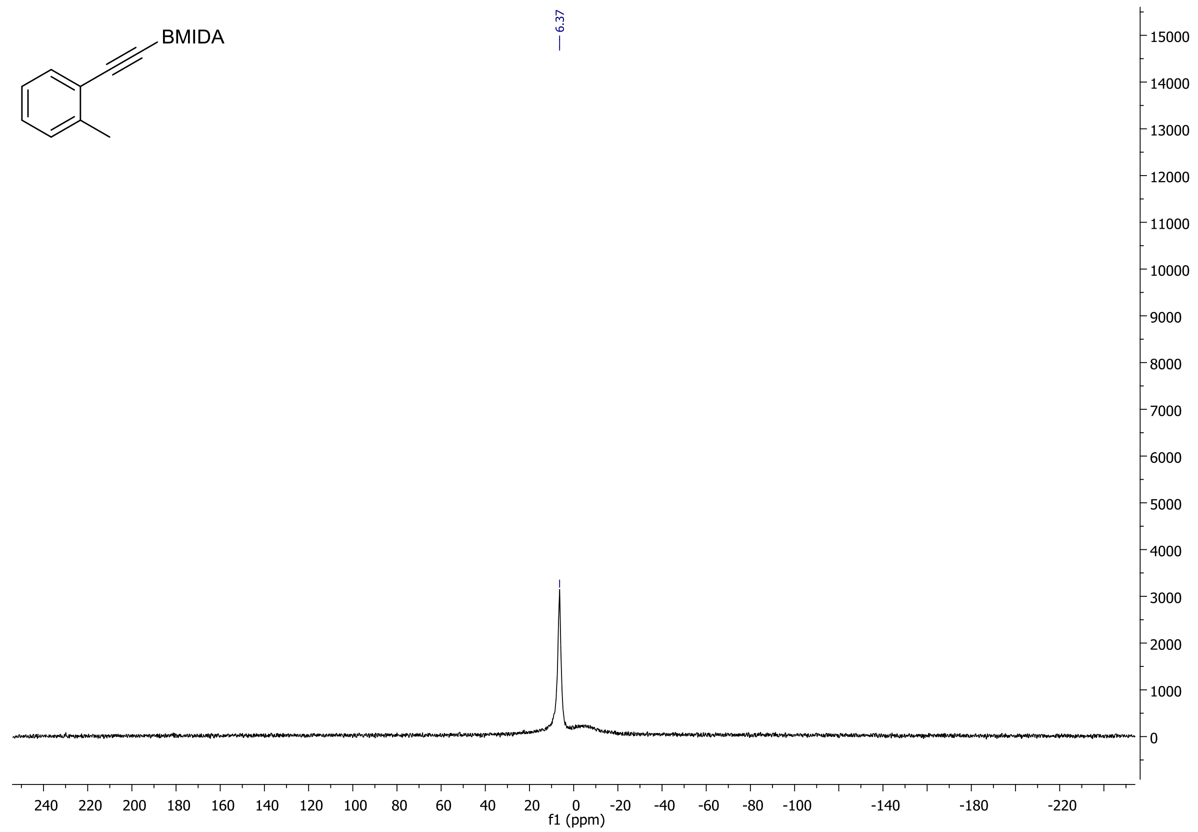

^{1}H NMR 3ae

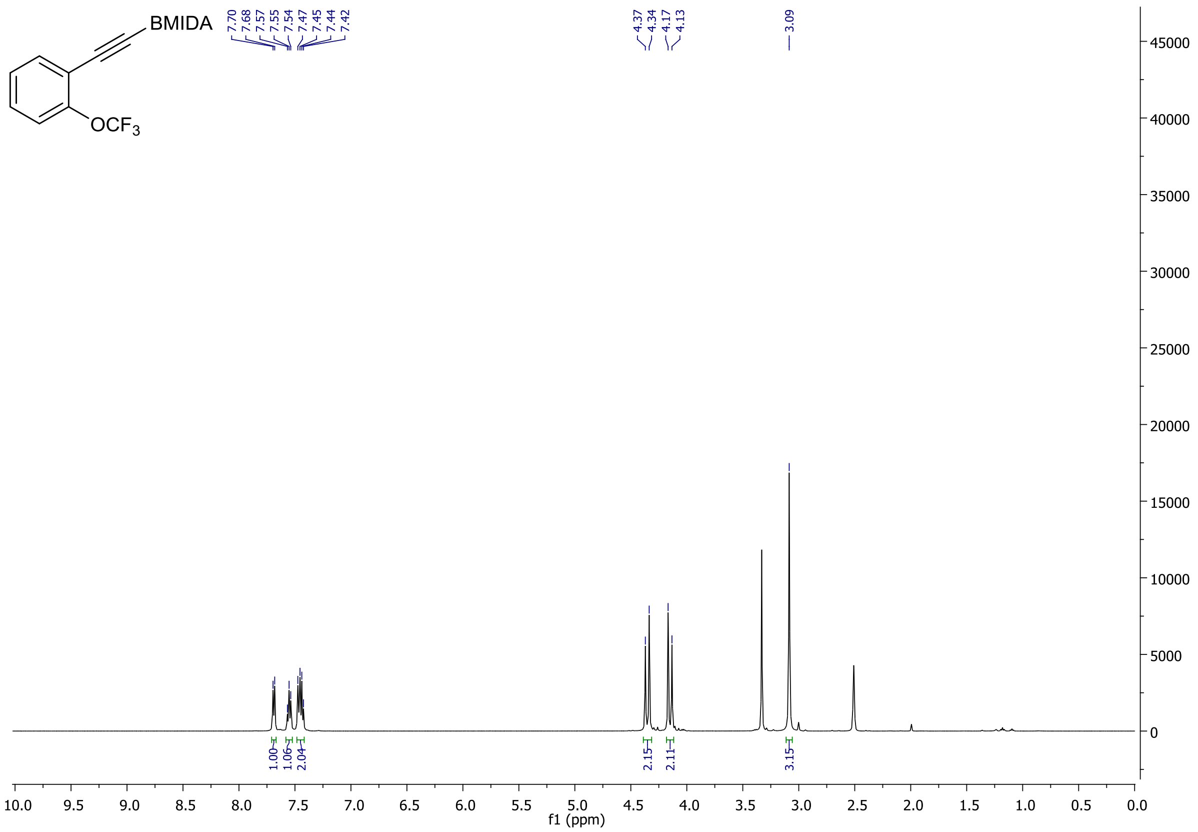

^{13}C NMR 3ae

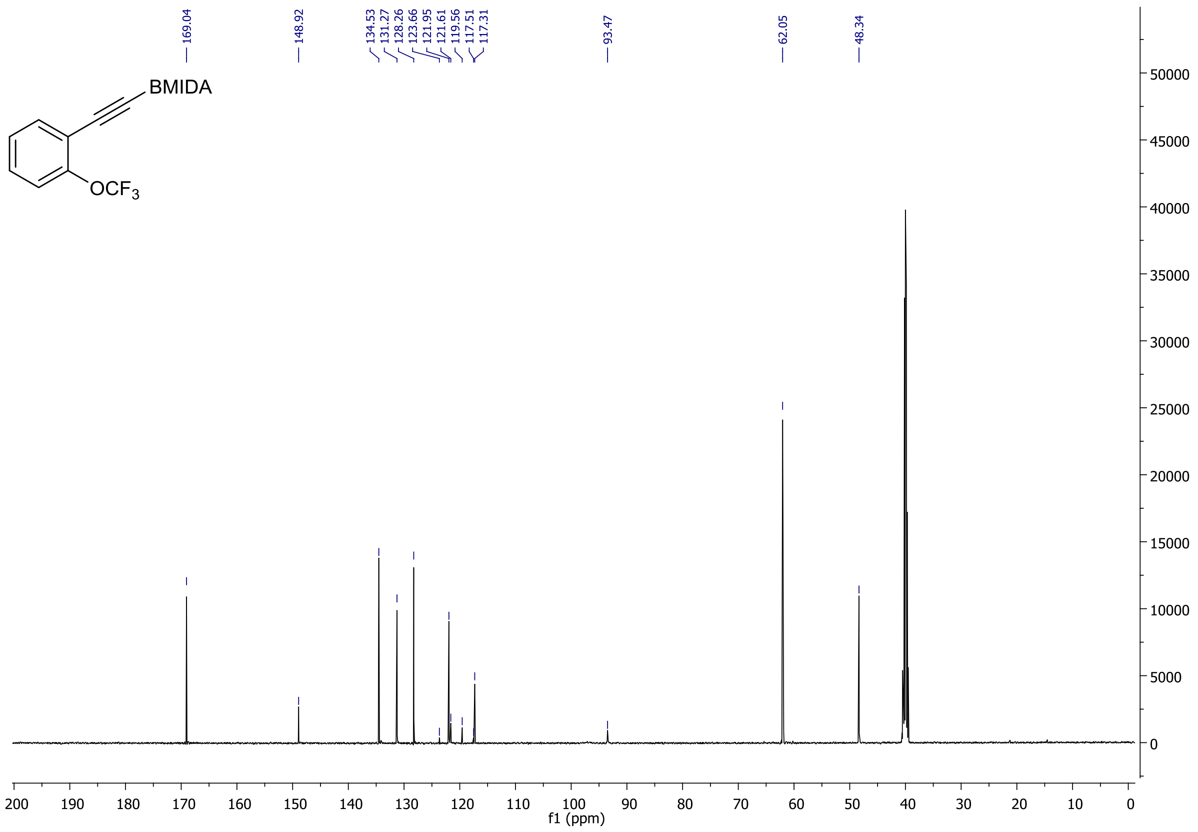

^{11}B NMR 3ae

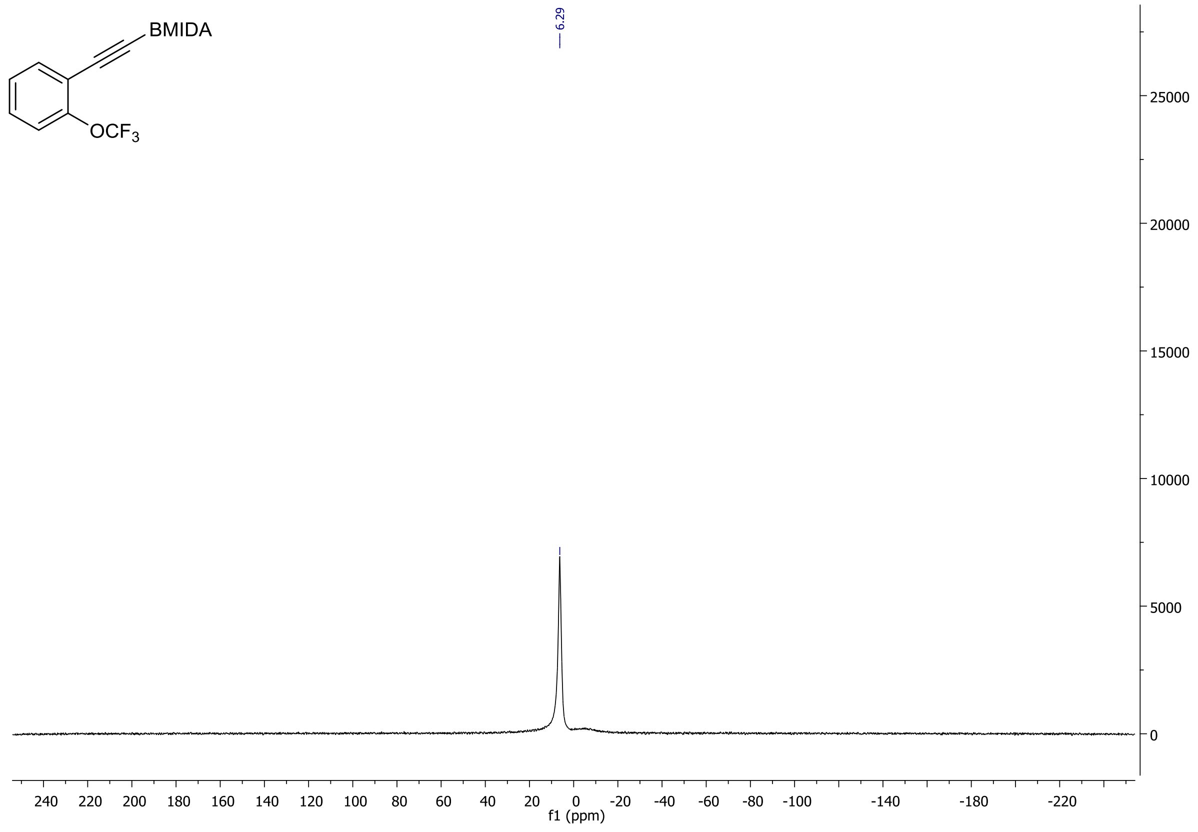

^{19}F NMR 3ae

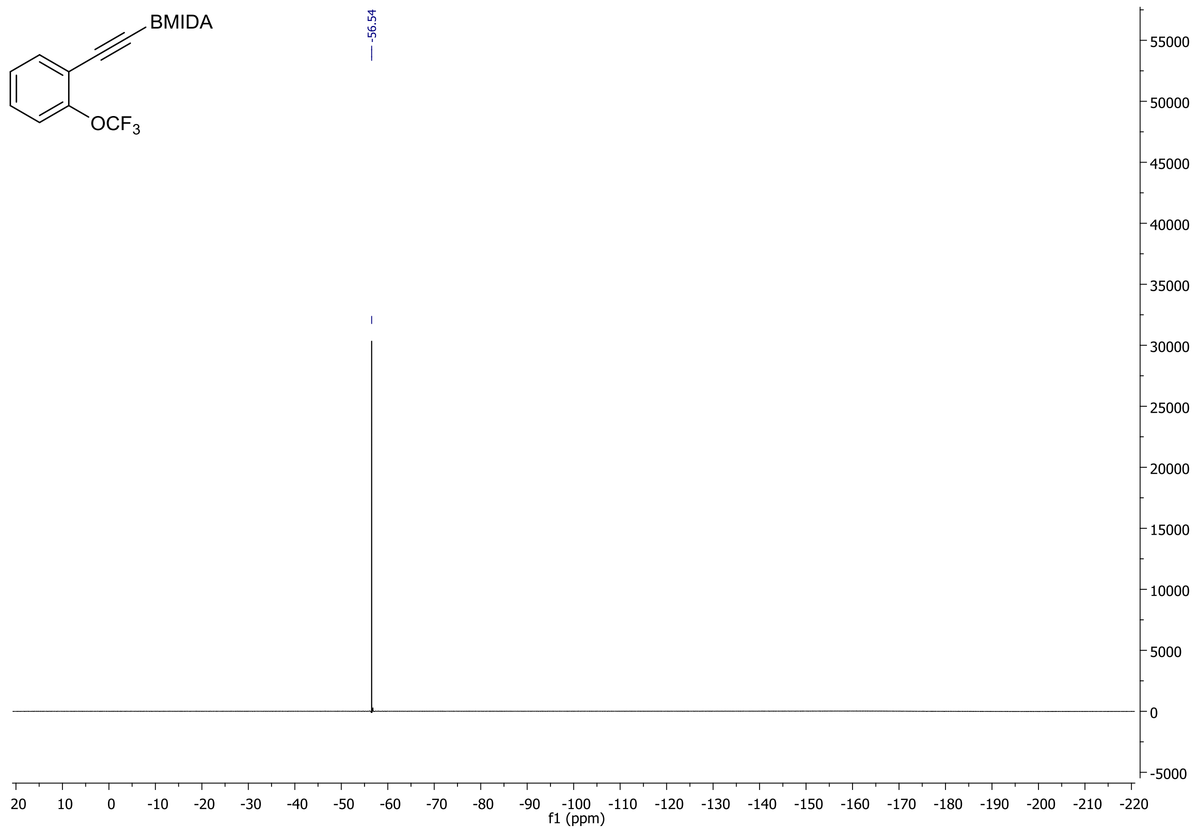

^{1}H NMR 3af

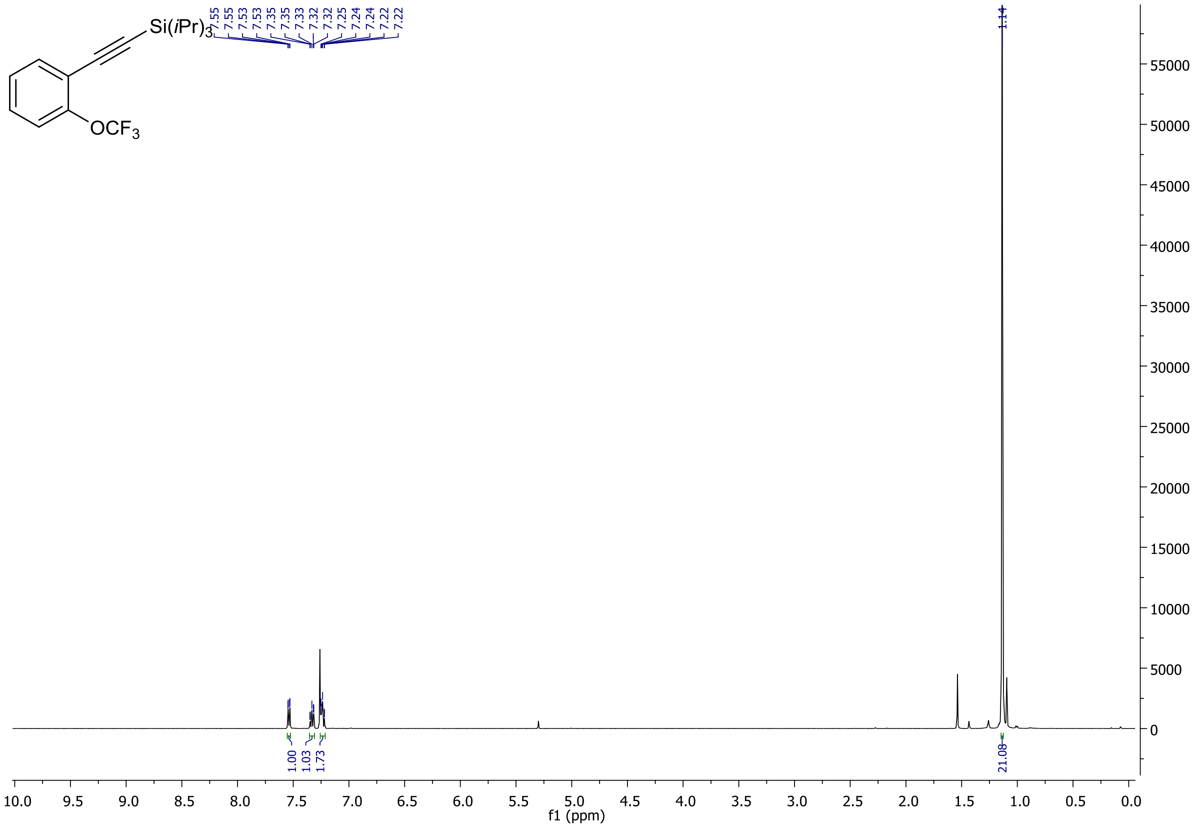

^{13}C NMR 3af

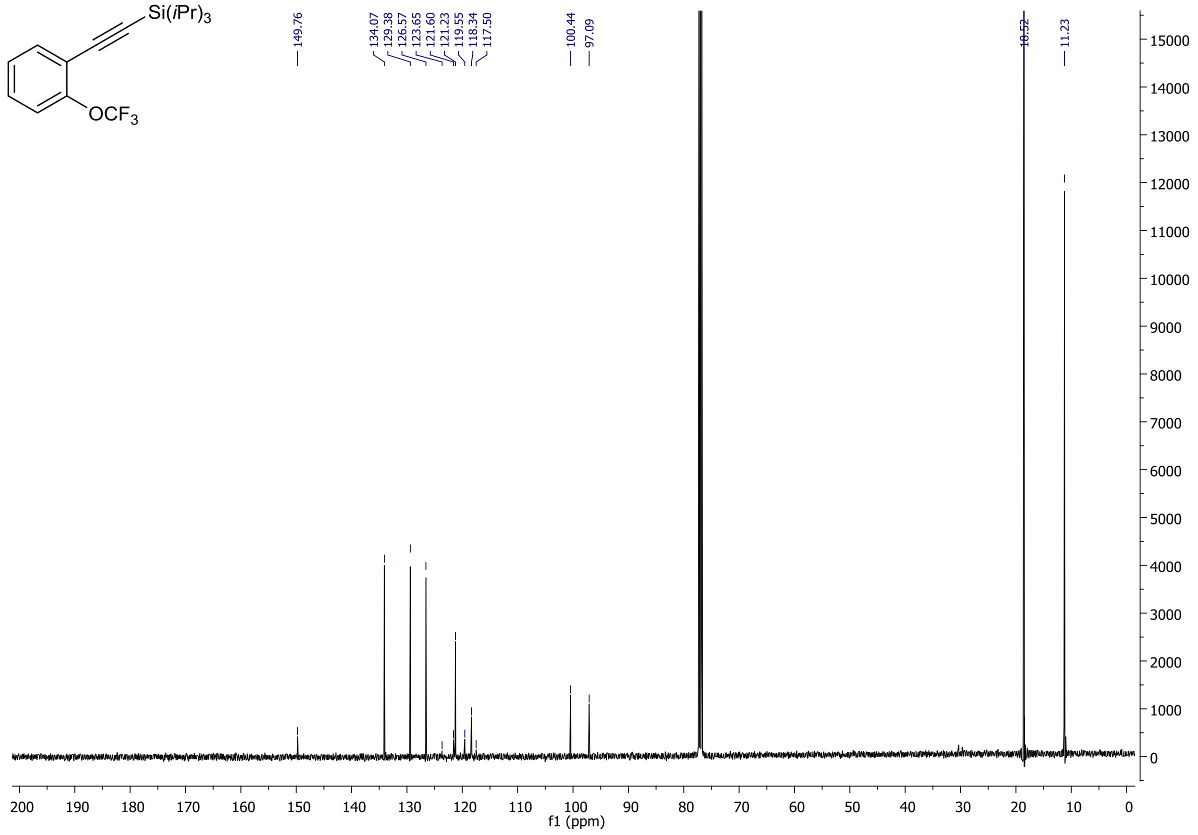

^{19}F NMR 3af

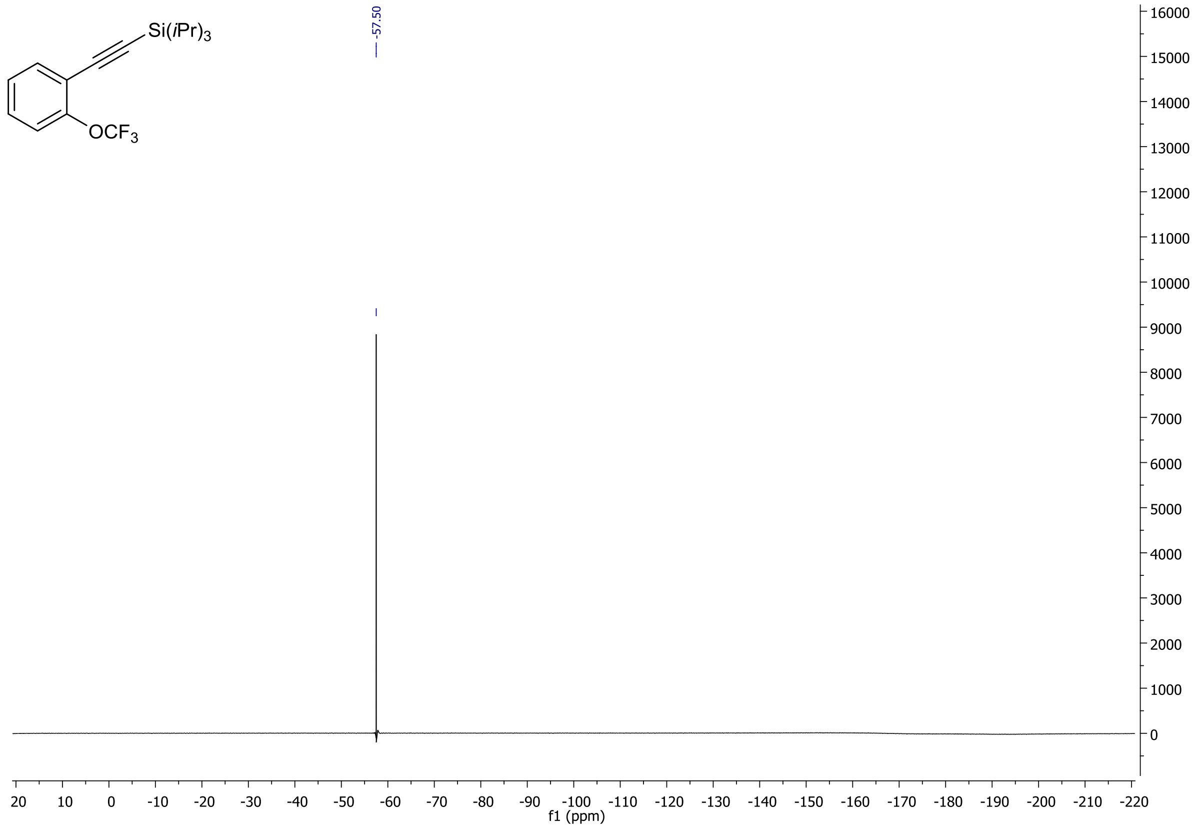

^{1}H NMR 3ag

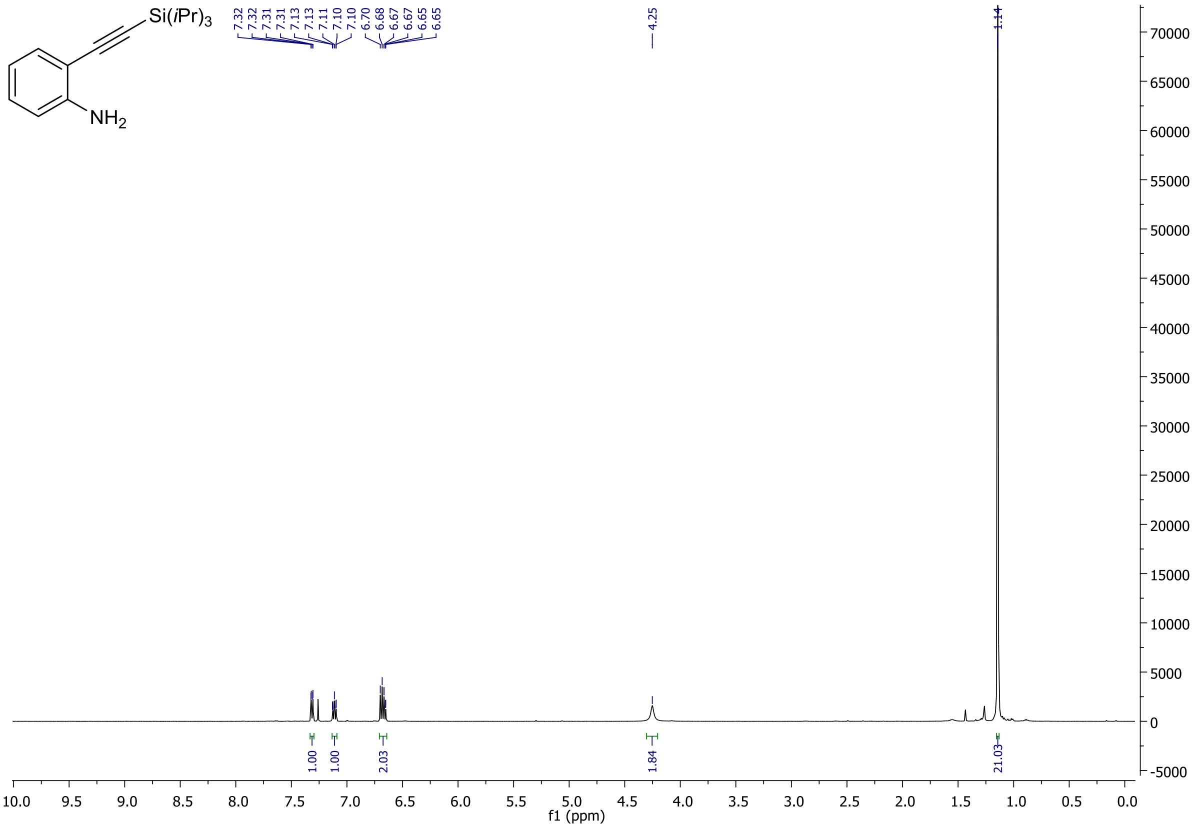

^{13}C NMR 3ag

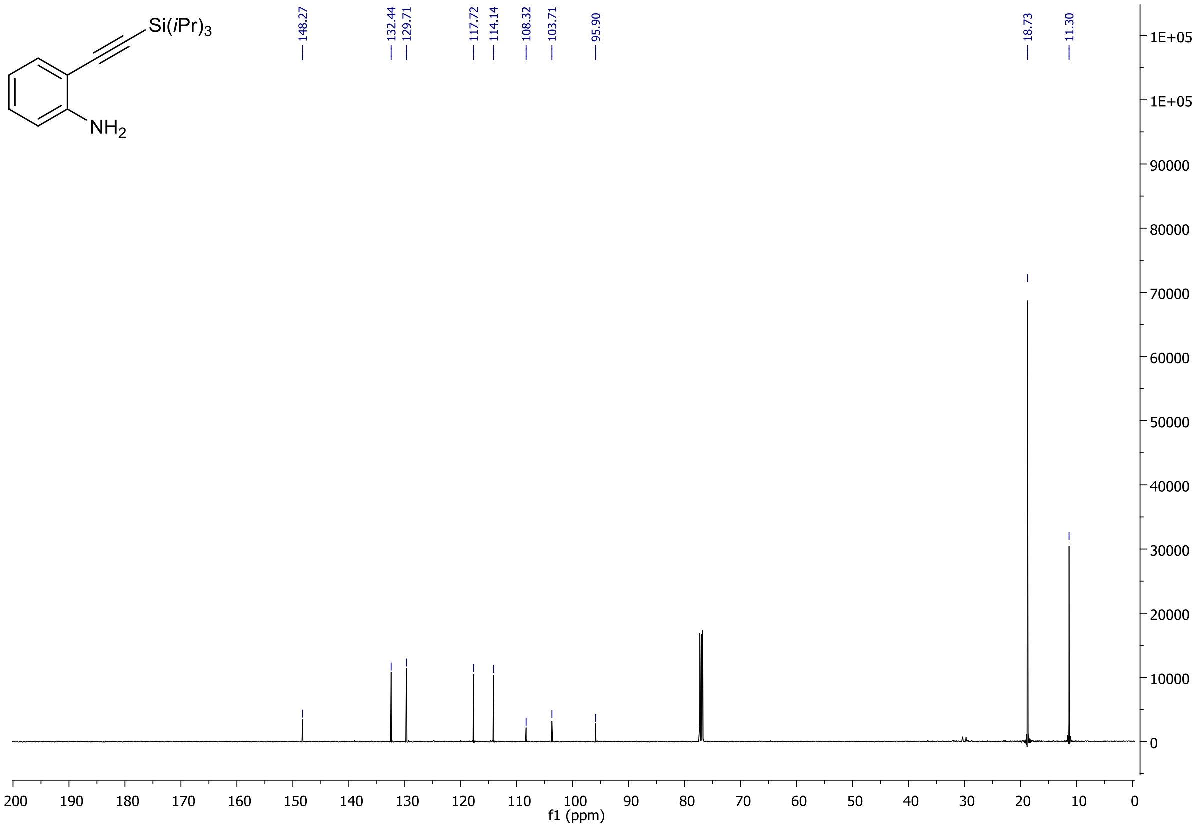

^{1}H NMR 3ah

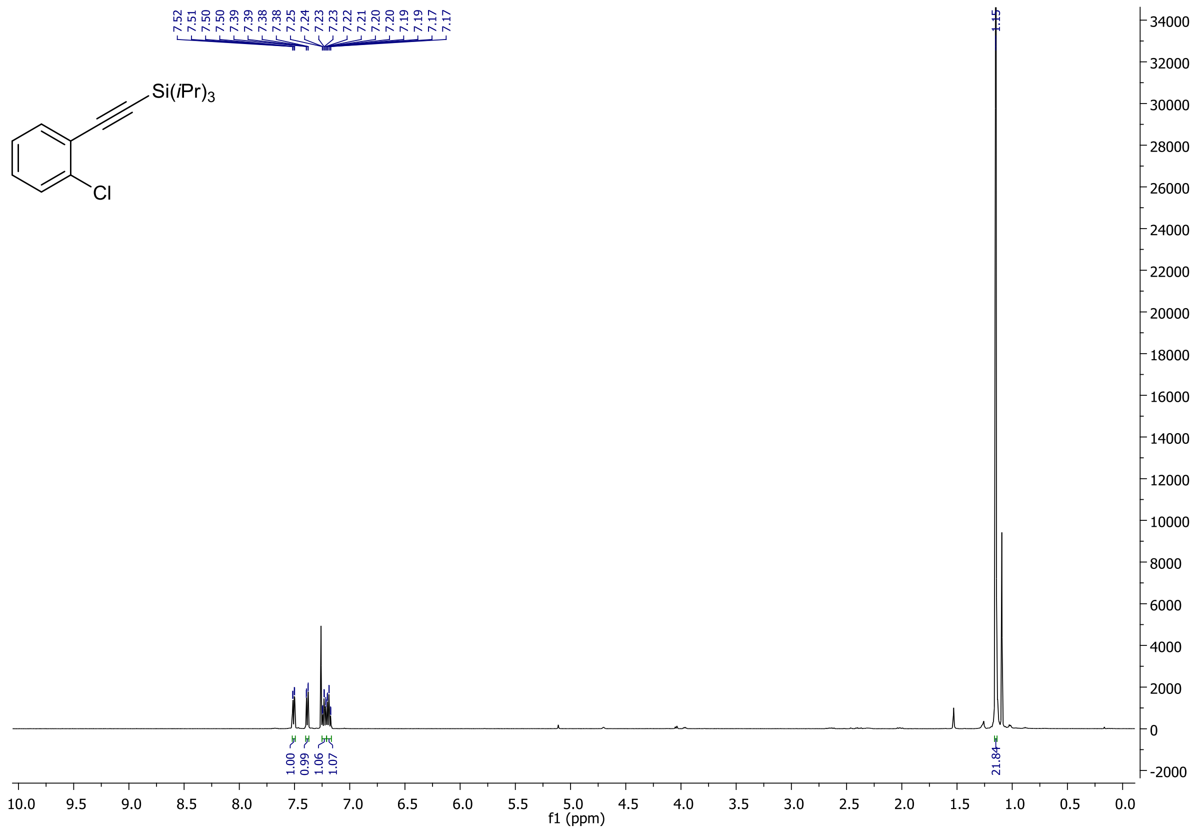

^{13}C NMR 3ah

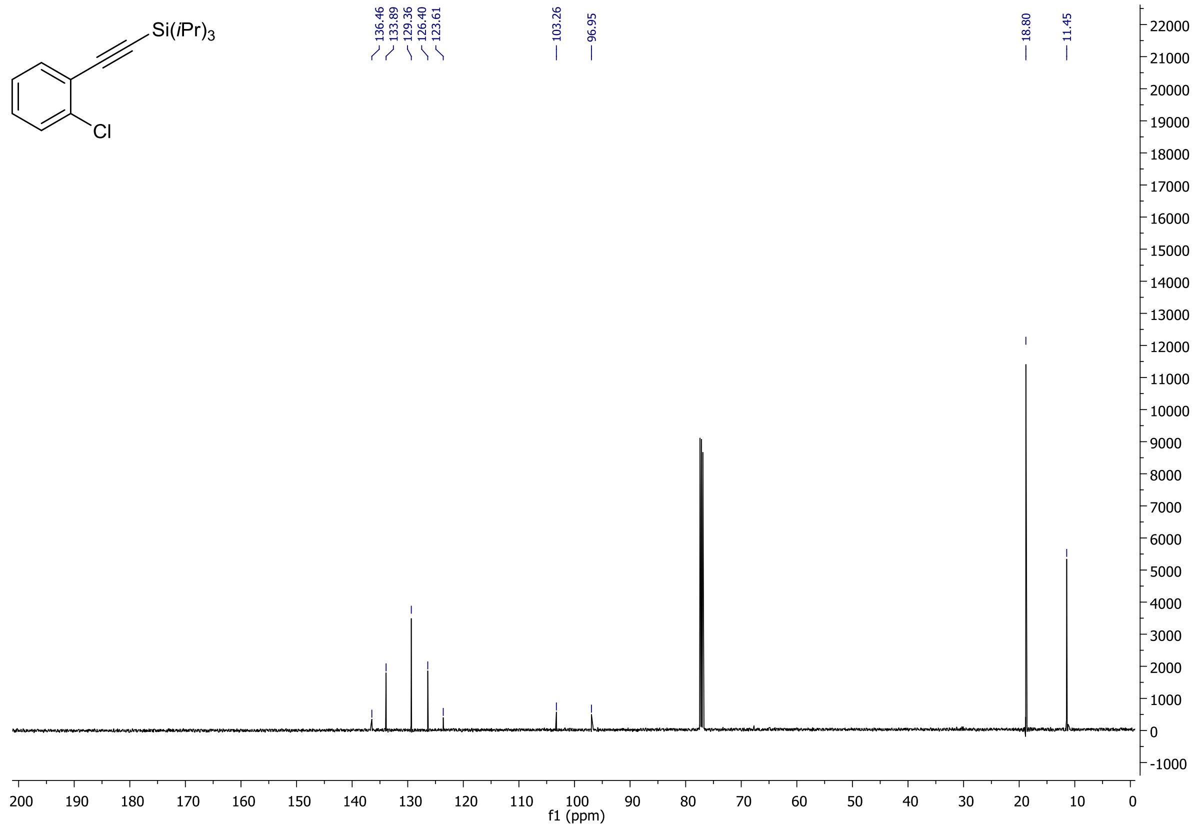

^{1}H NMR of 4a

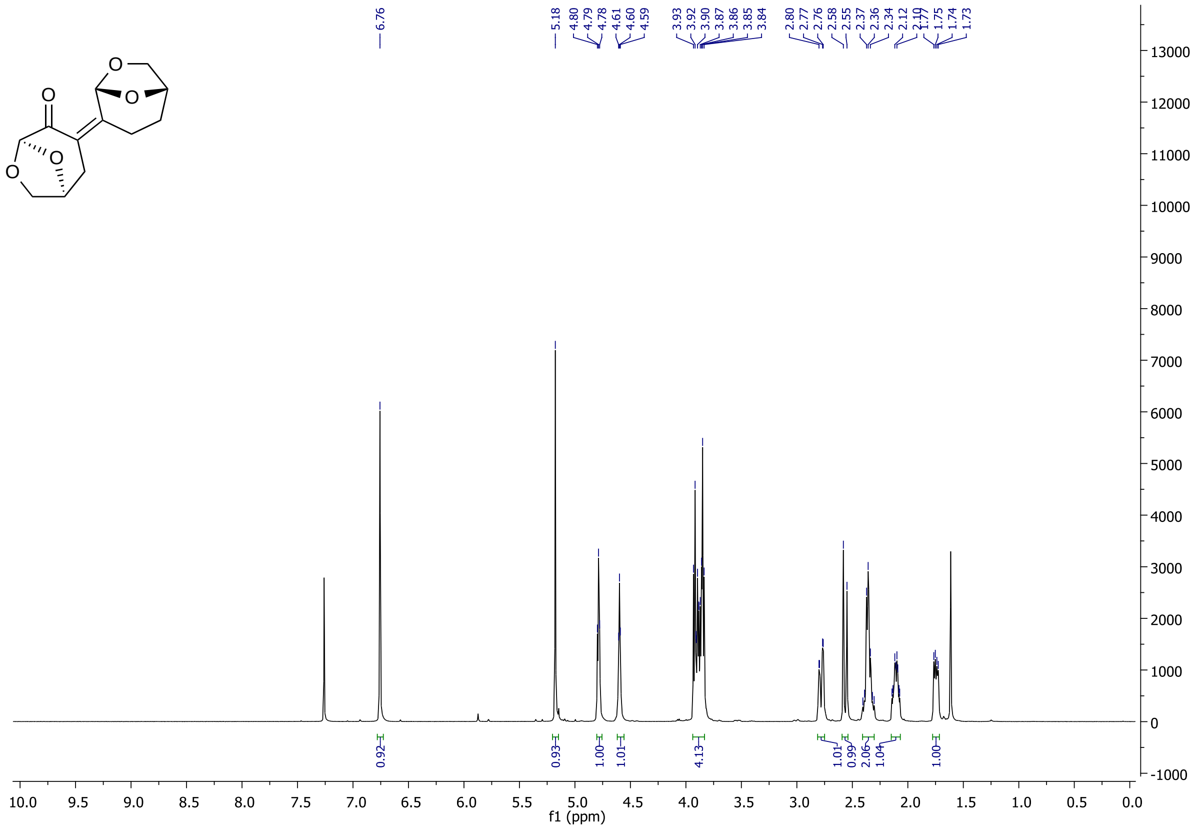

^{13}C NMR of 4a

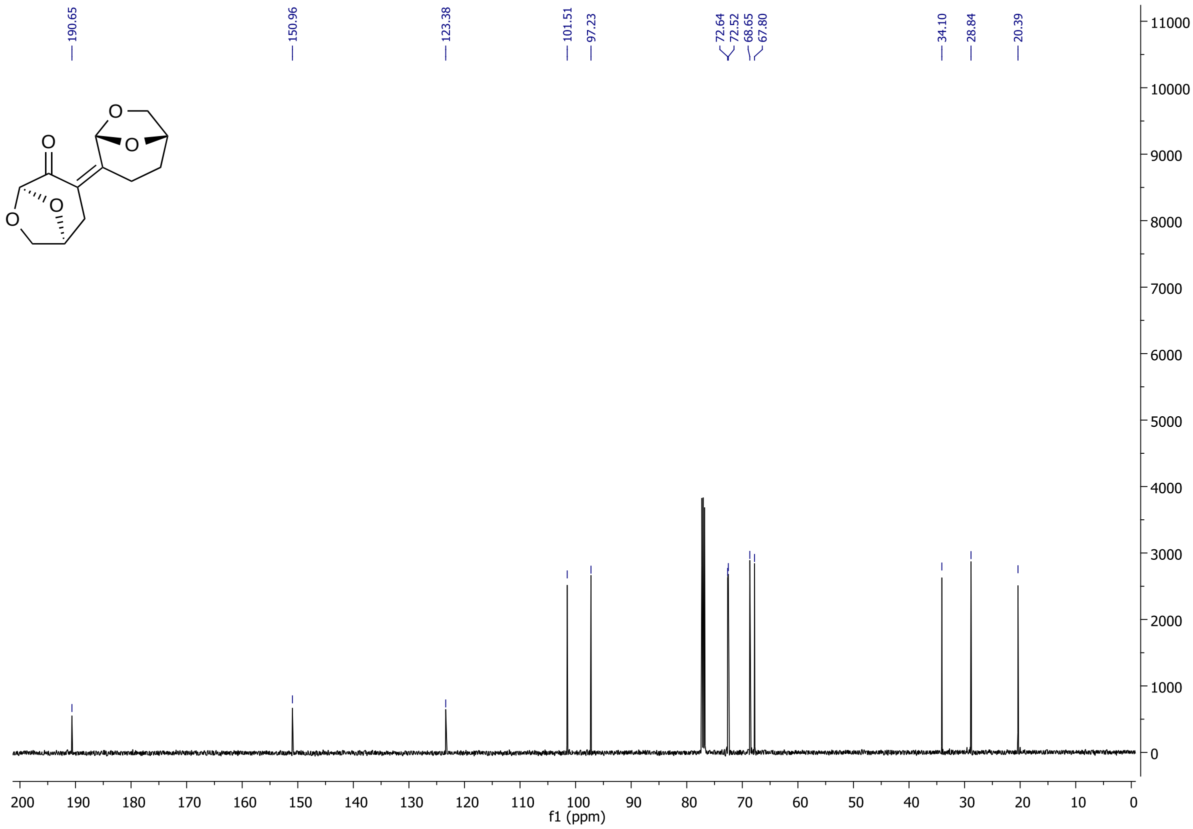

^{1}H NMR 7a

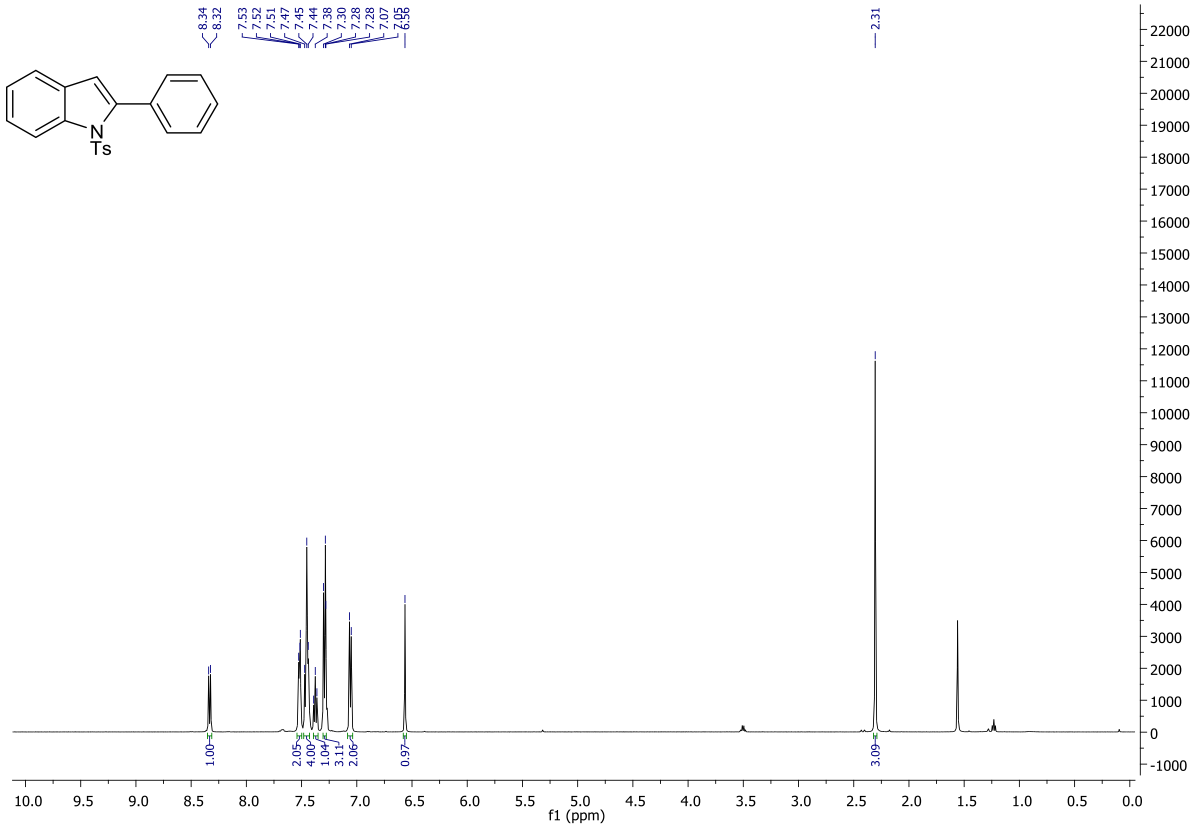

^{13}C NMR 7a

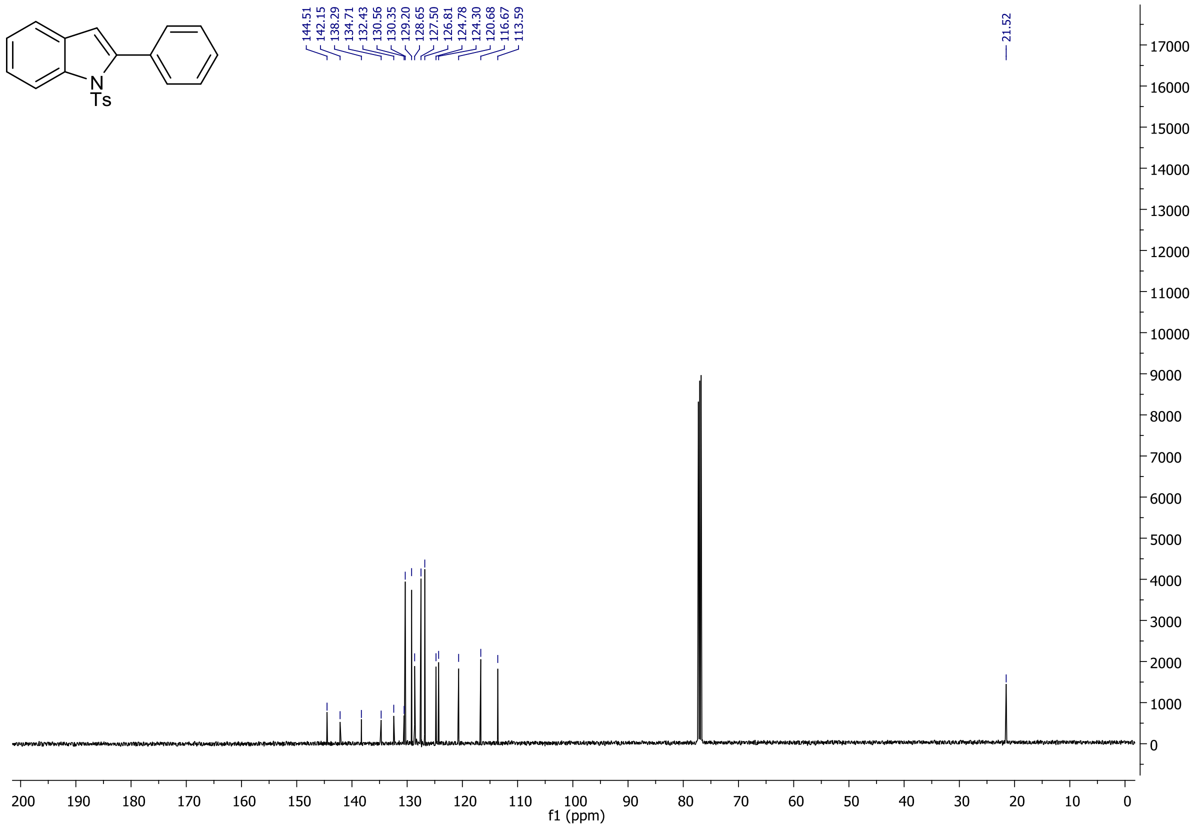

^{1}H NMR 7b

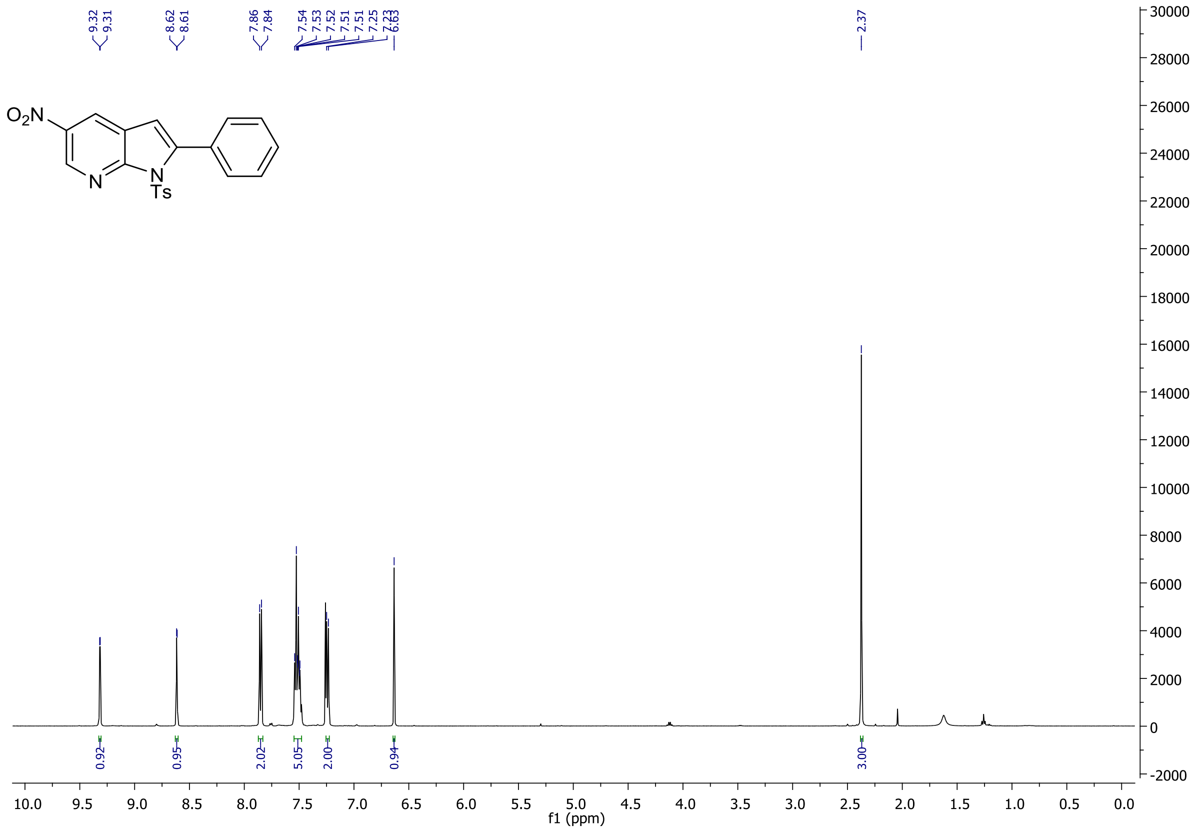

^{13}C NMR 7b

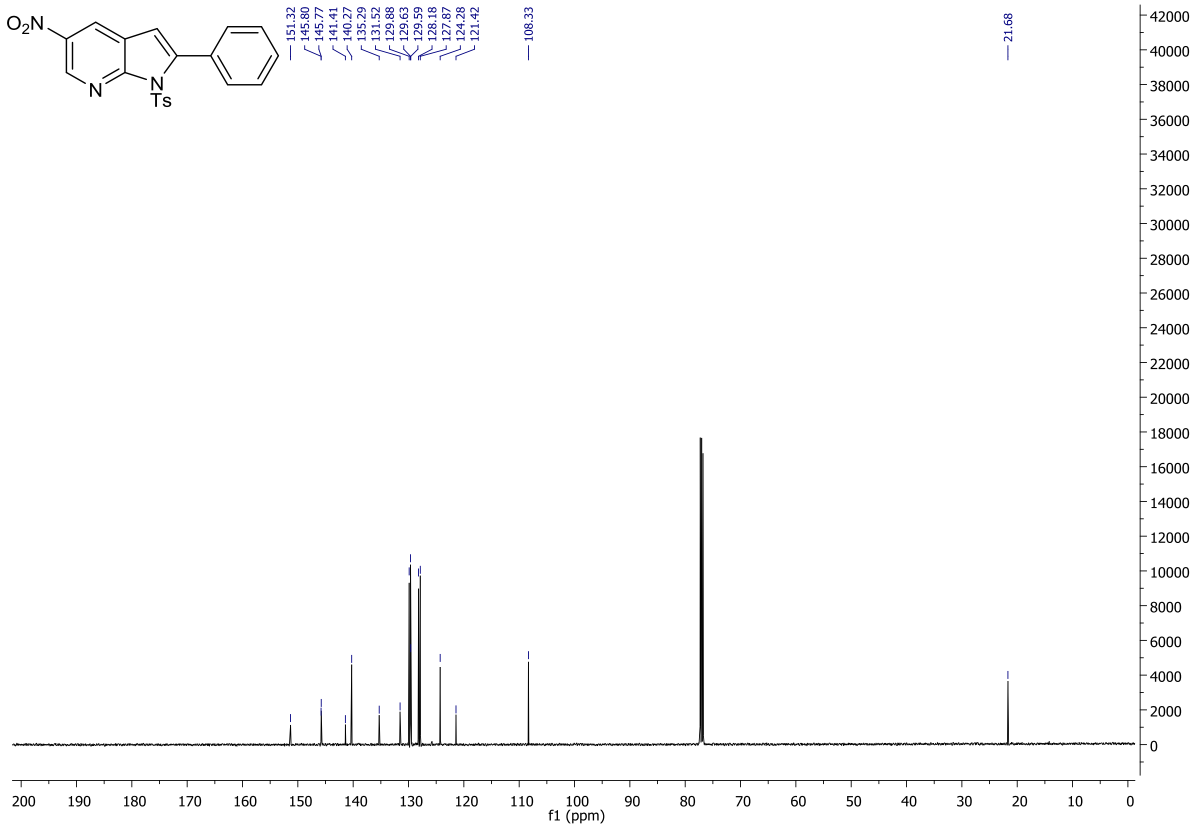

^{1}H NMR 7c

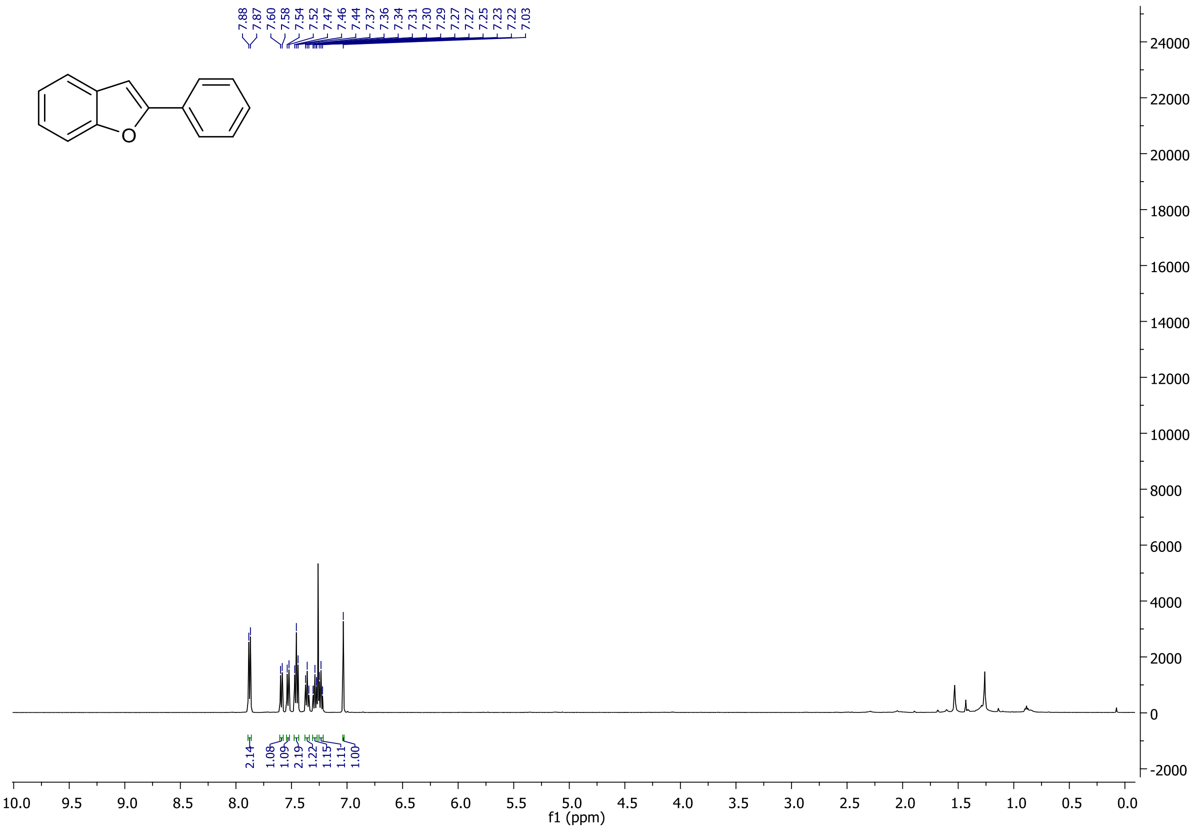

^{13}C NMR 7c

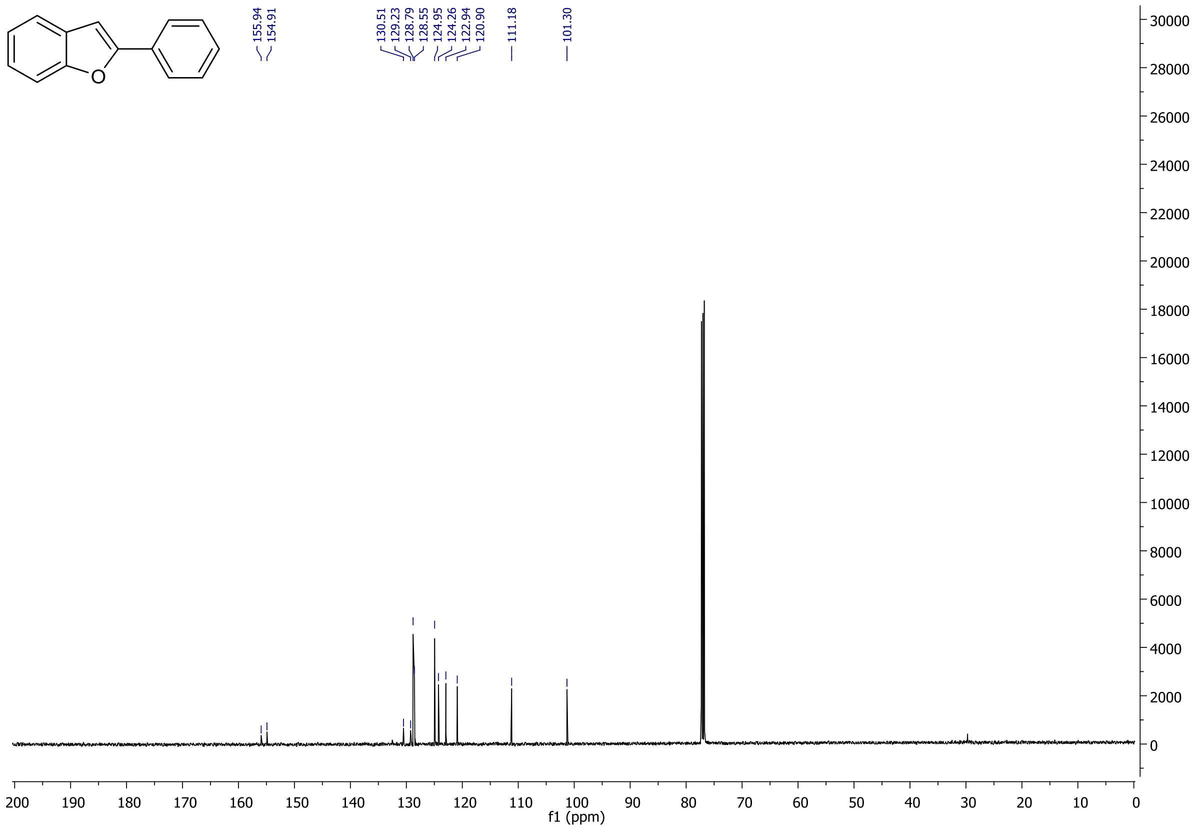

^{1}H NMR 7d

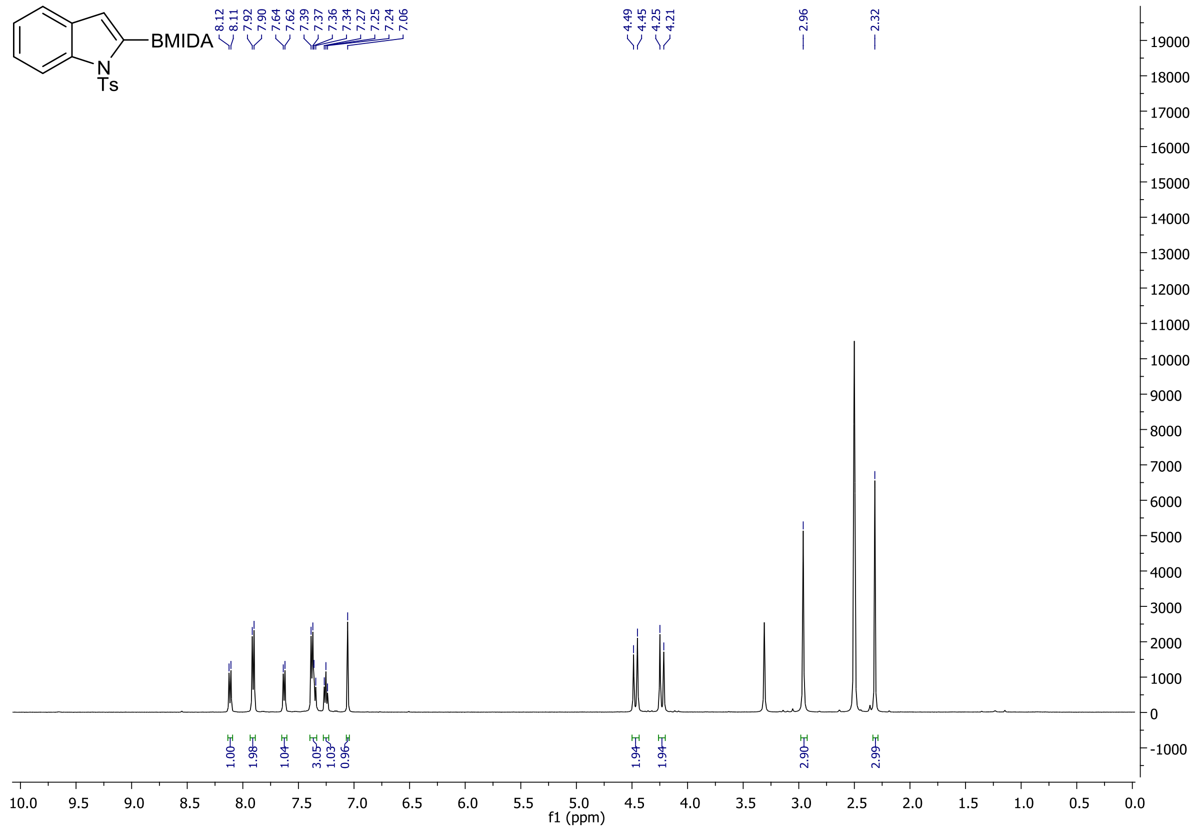

^{13}C NMR 7d

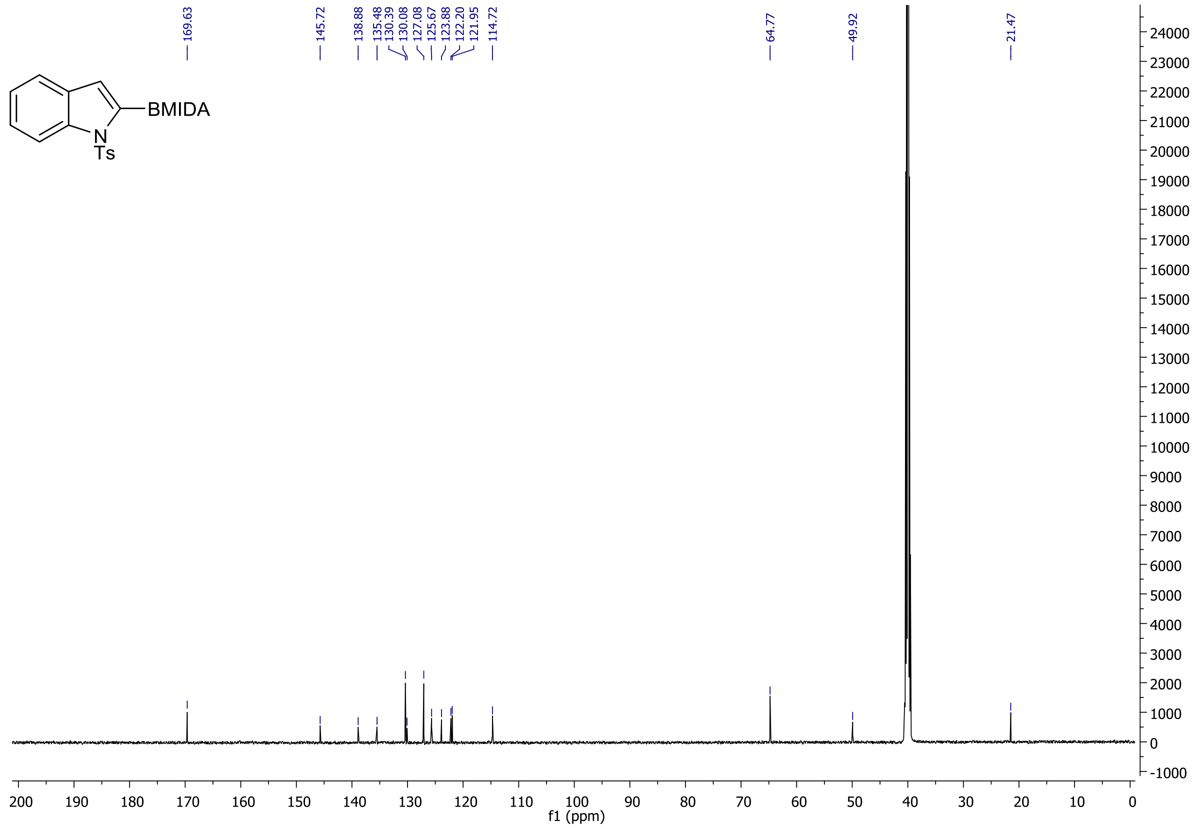

^{11}B NMR 7d

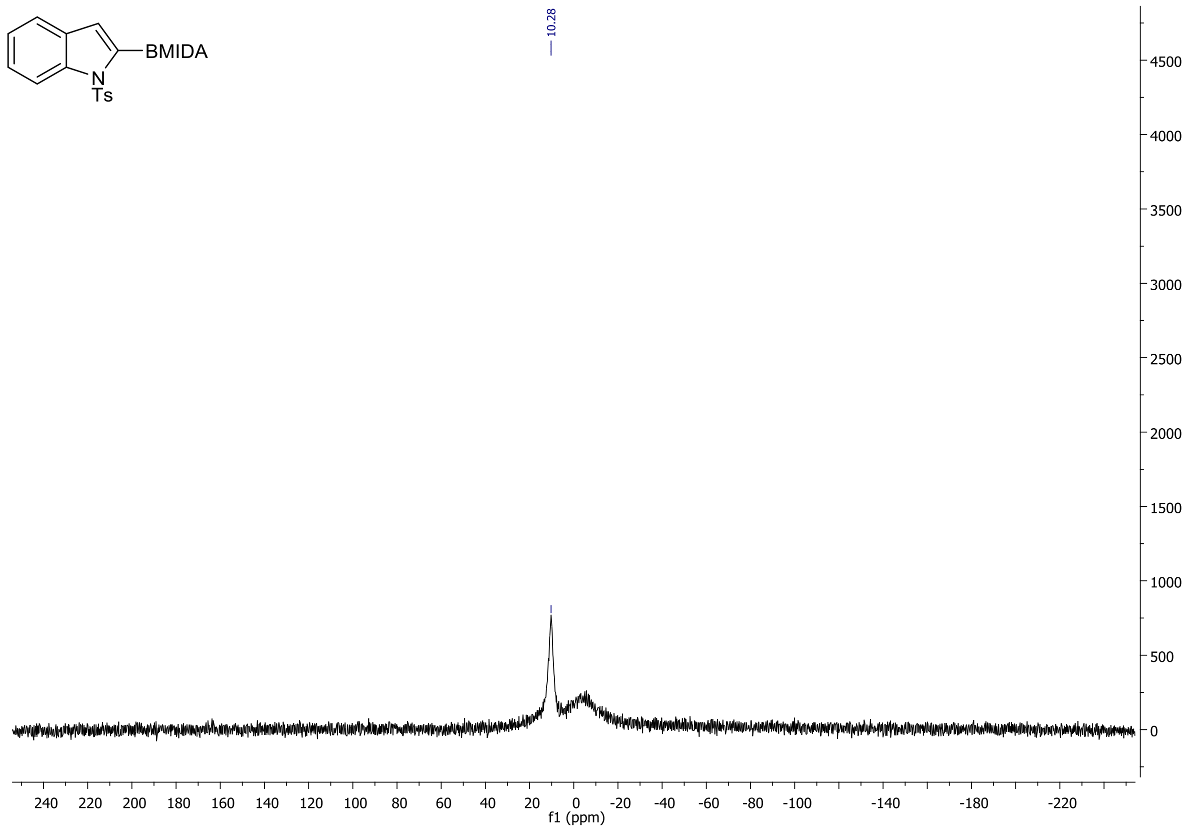

^{1}H NMR 7e

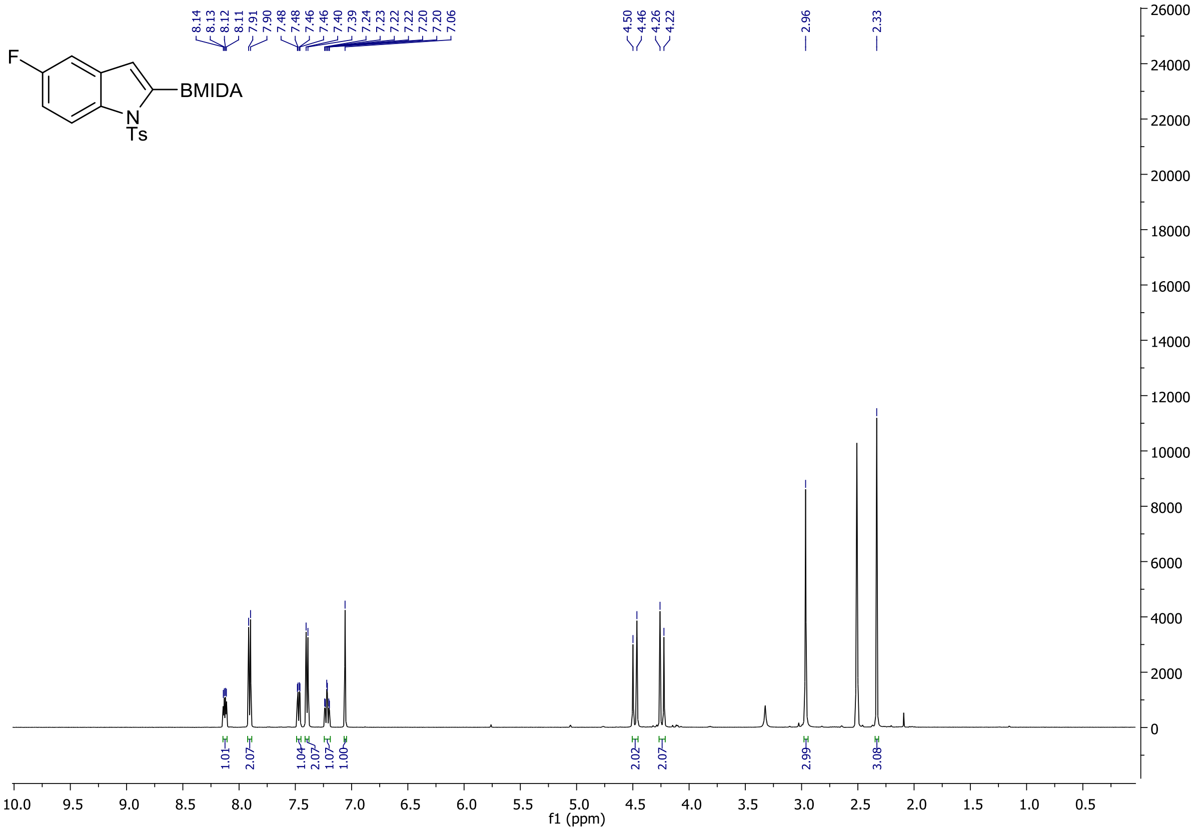

^{13}C NMR 7e

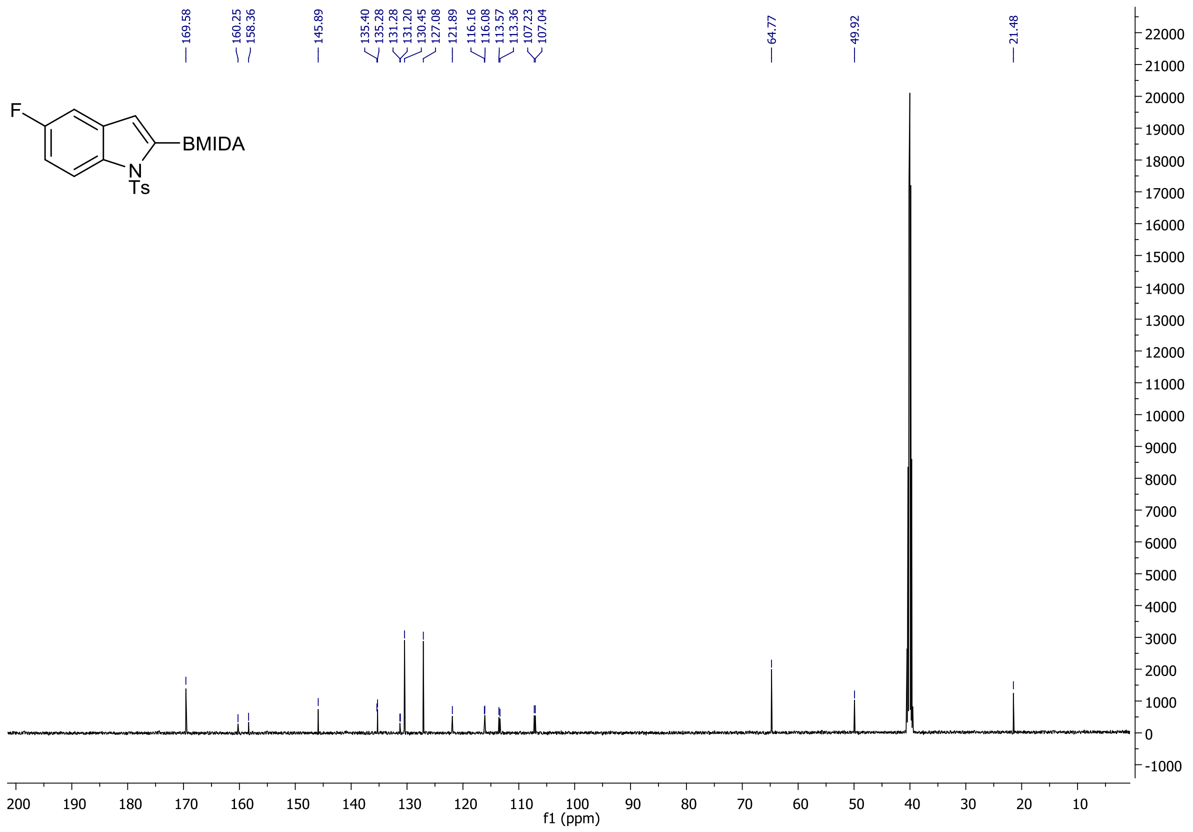

^{11}B NMR 7e

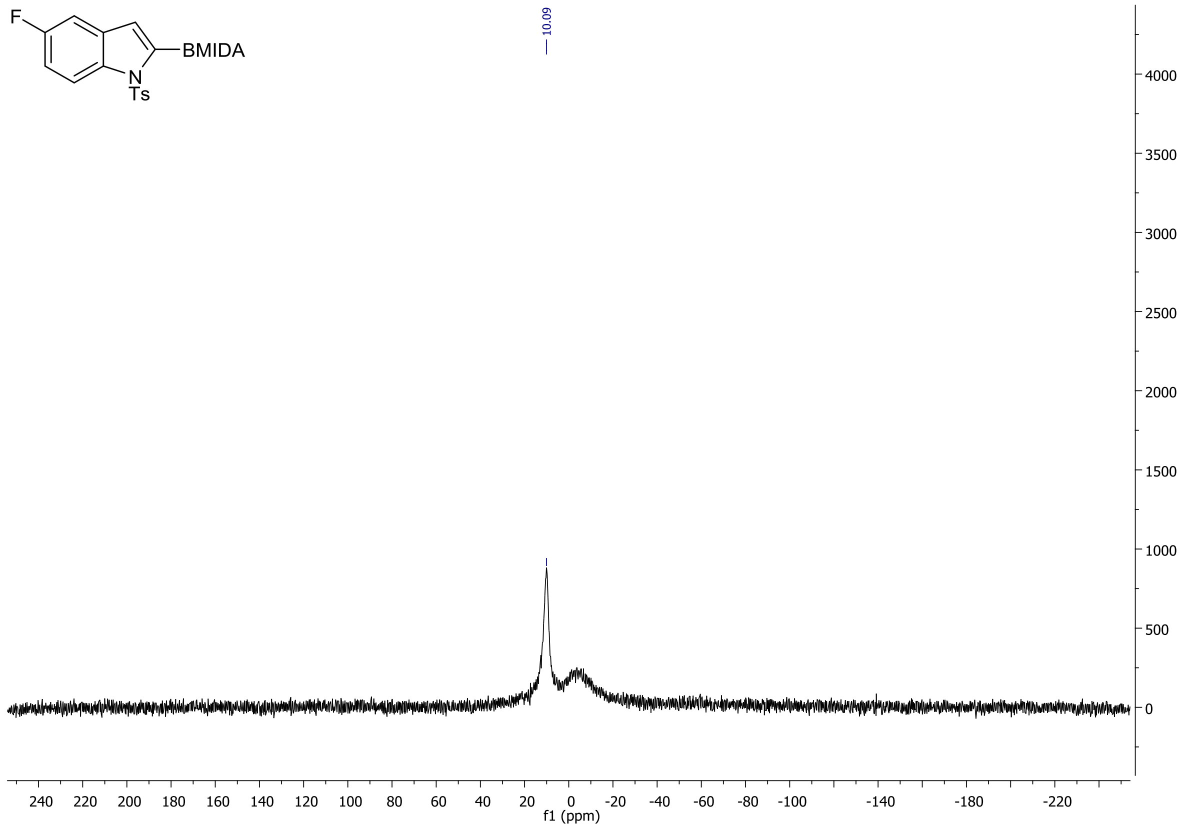

^{19}F NMR 7e

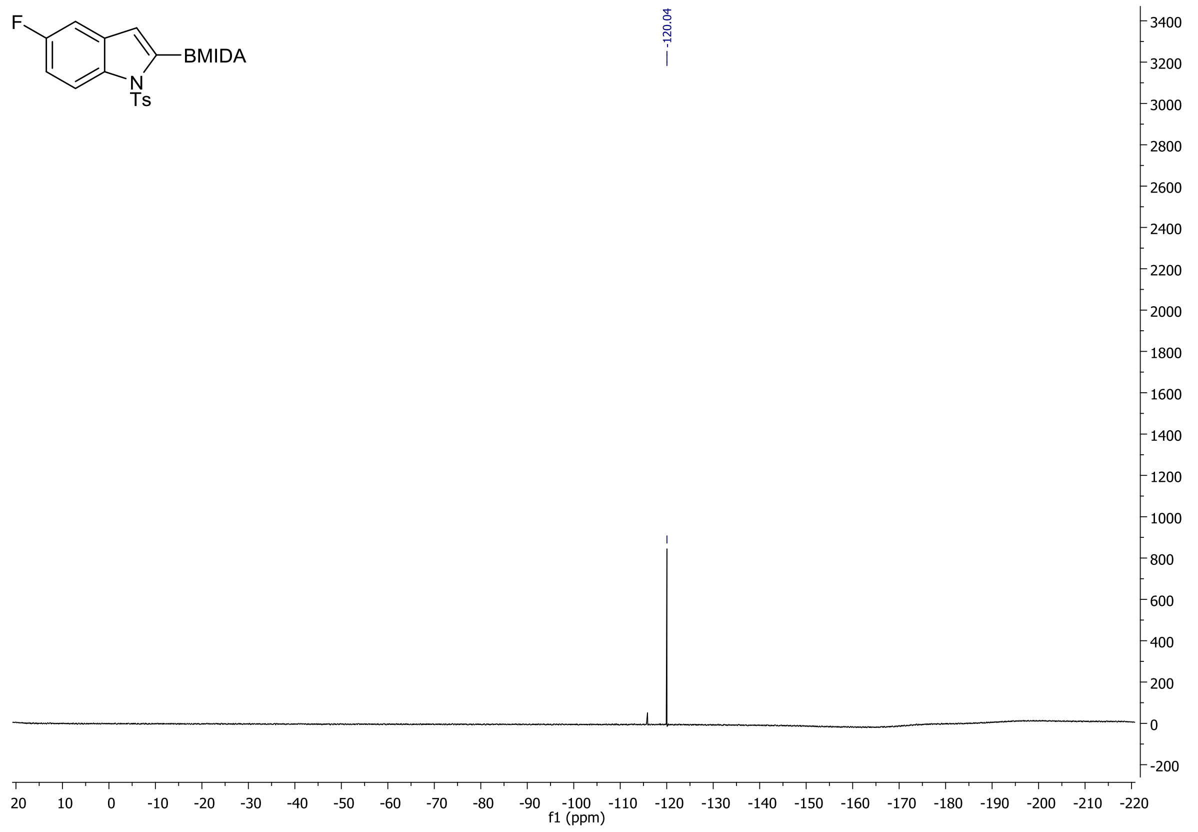

^{1}H NMR 7f

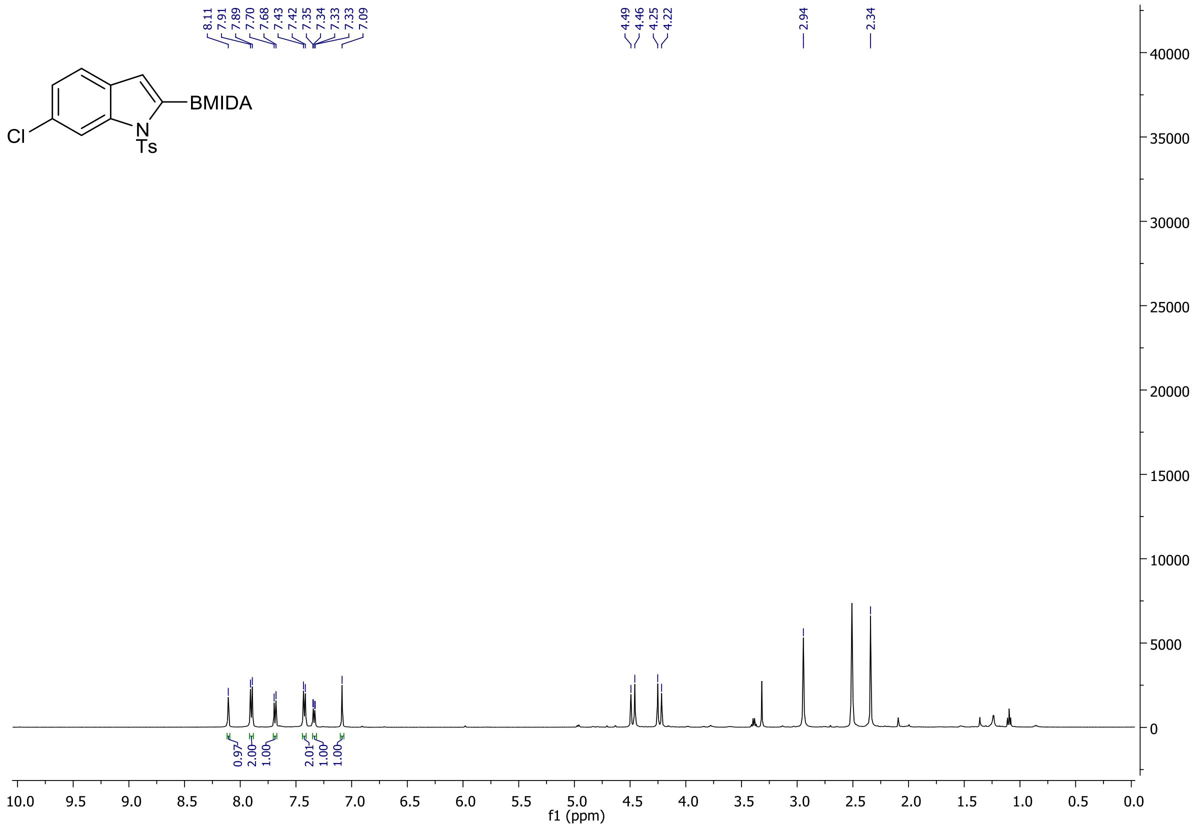

^{13}C NMR 7f

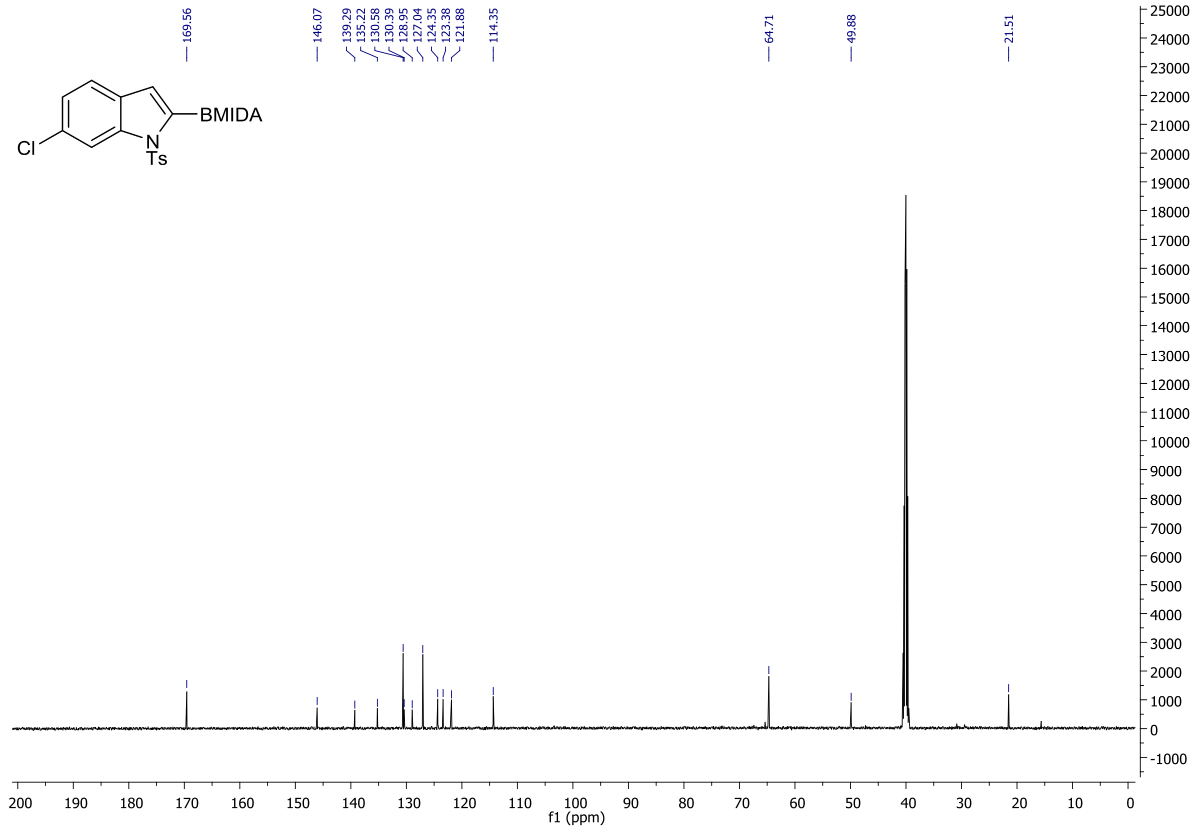

^{11}B NMR 7f

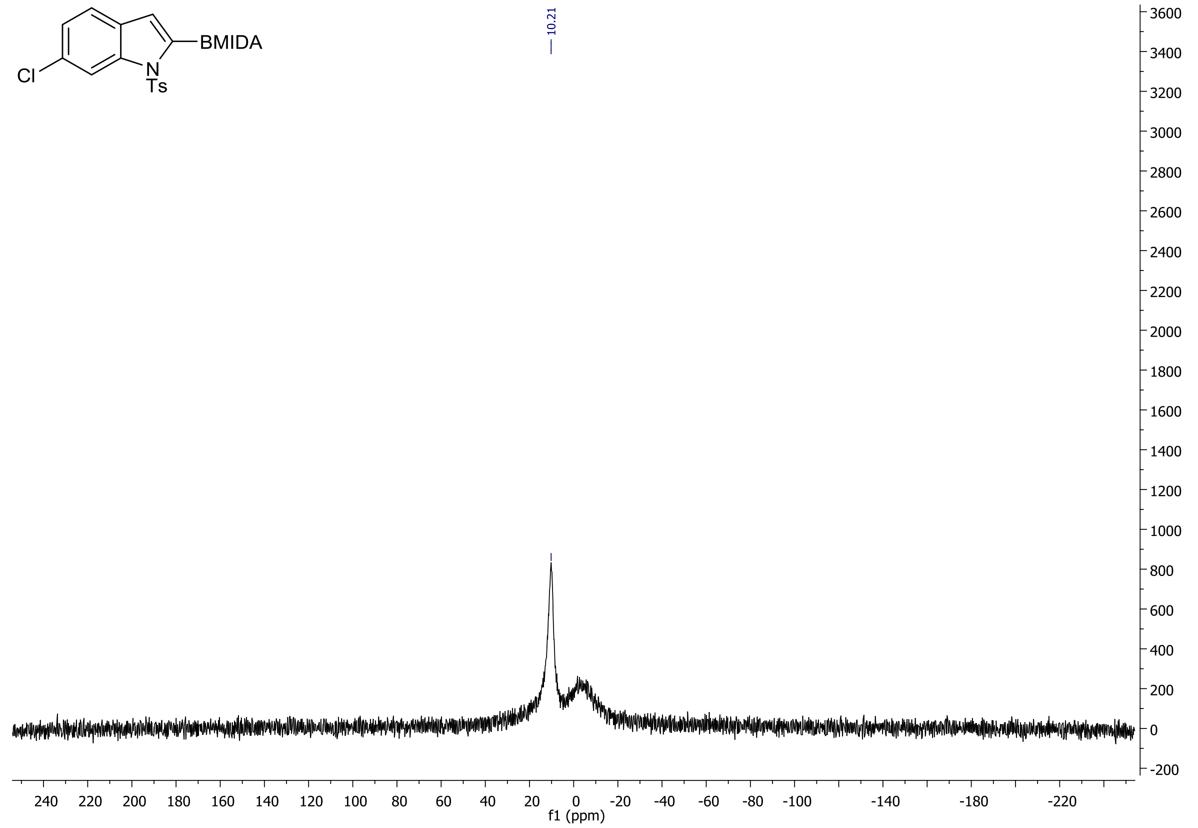

9. ^{1}H NMR Evidence for the Evaluation of the Base Sensitivity

Cyrene ^{1}H NMR

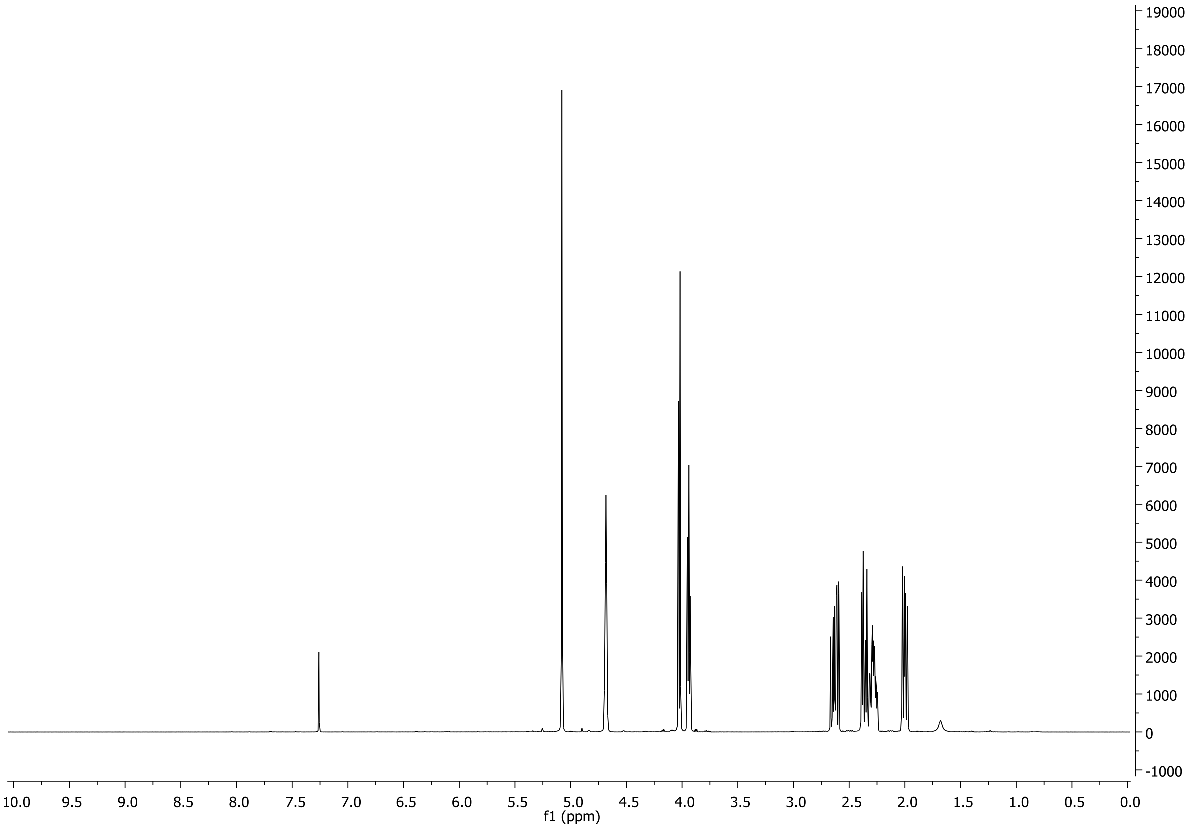

KOAc 25 °C

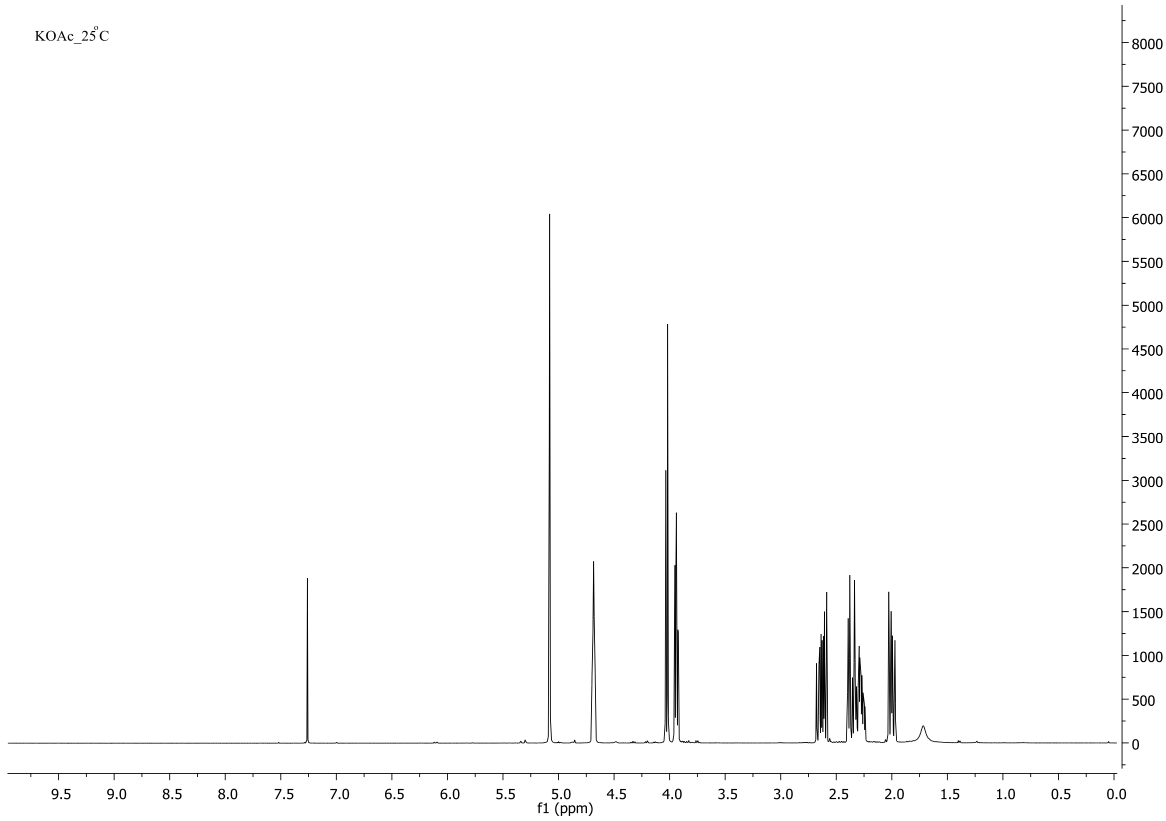

KOAc 50 °C

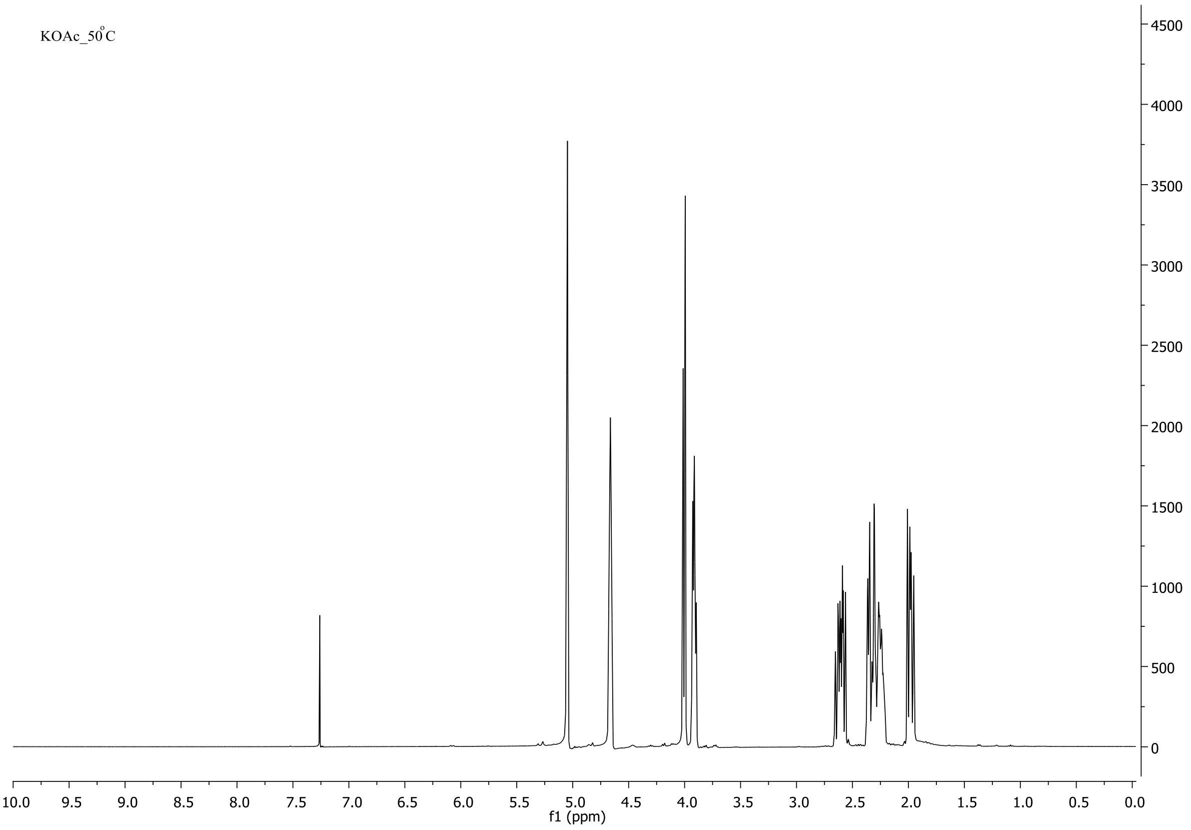

KOAc 100 °C

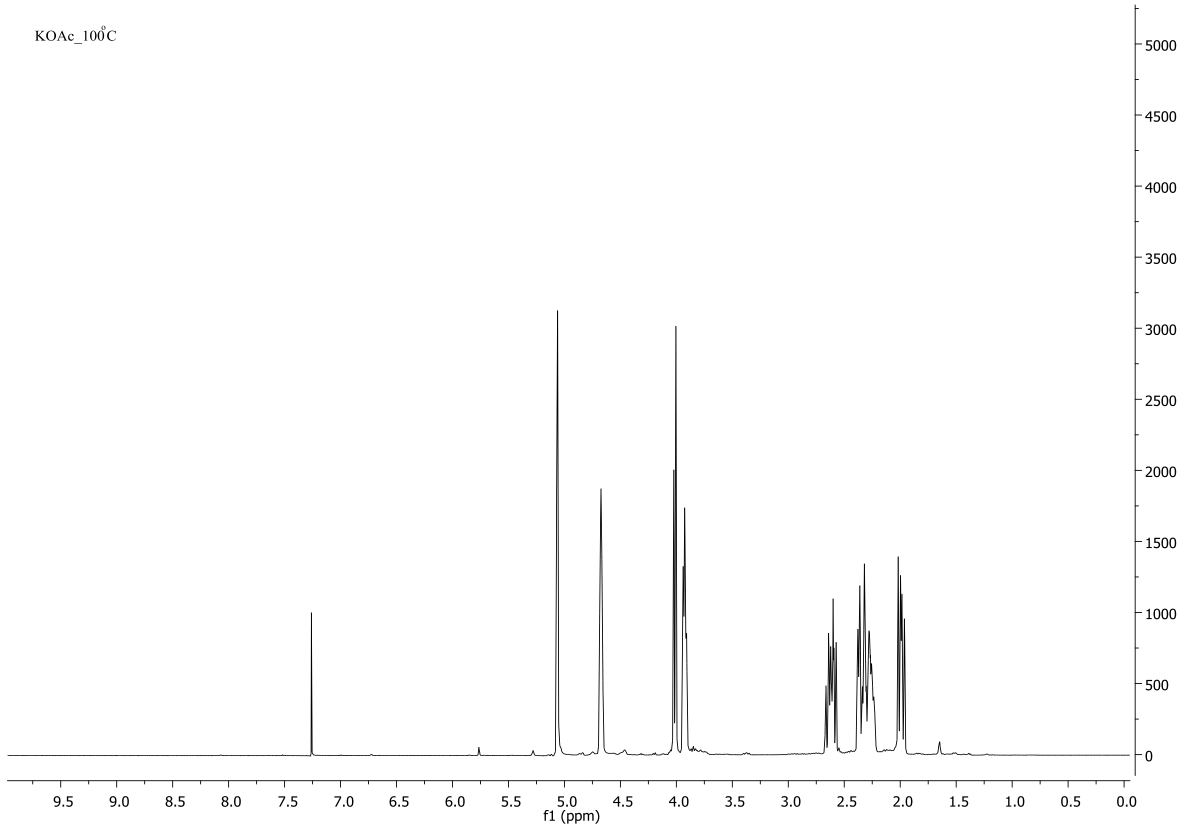

Pyridine 25 °C

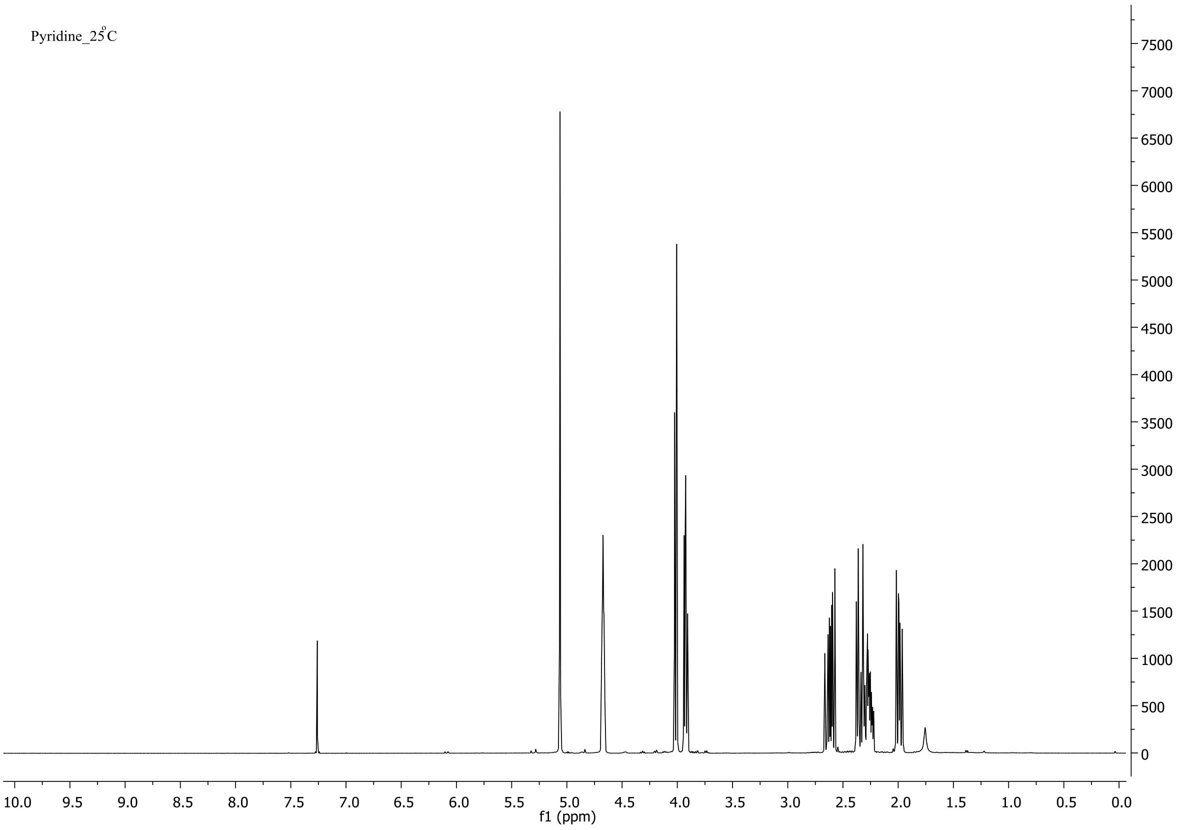

Pyridine 50 °C

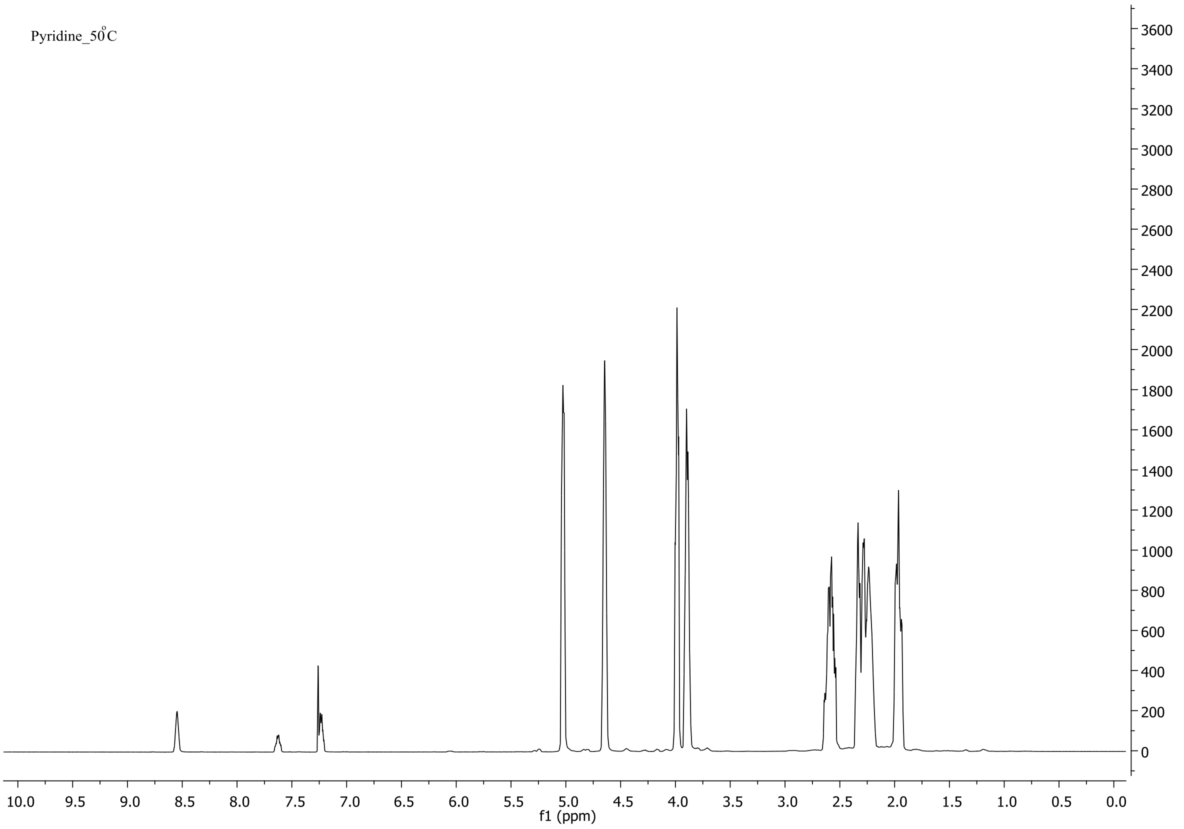

Pyridine 100 °C

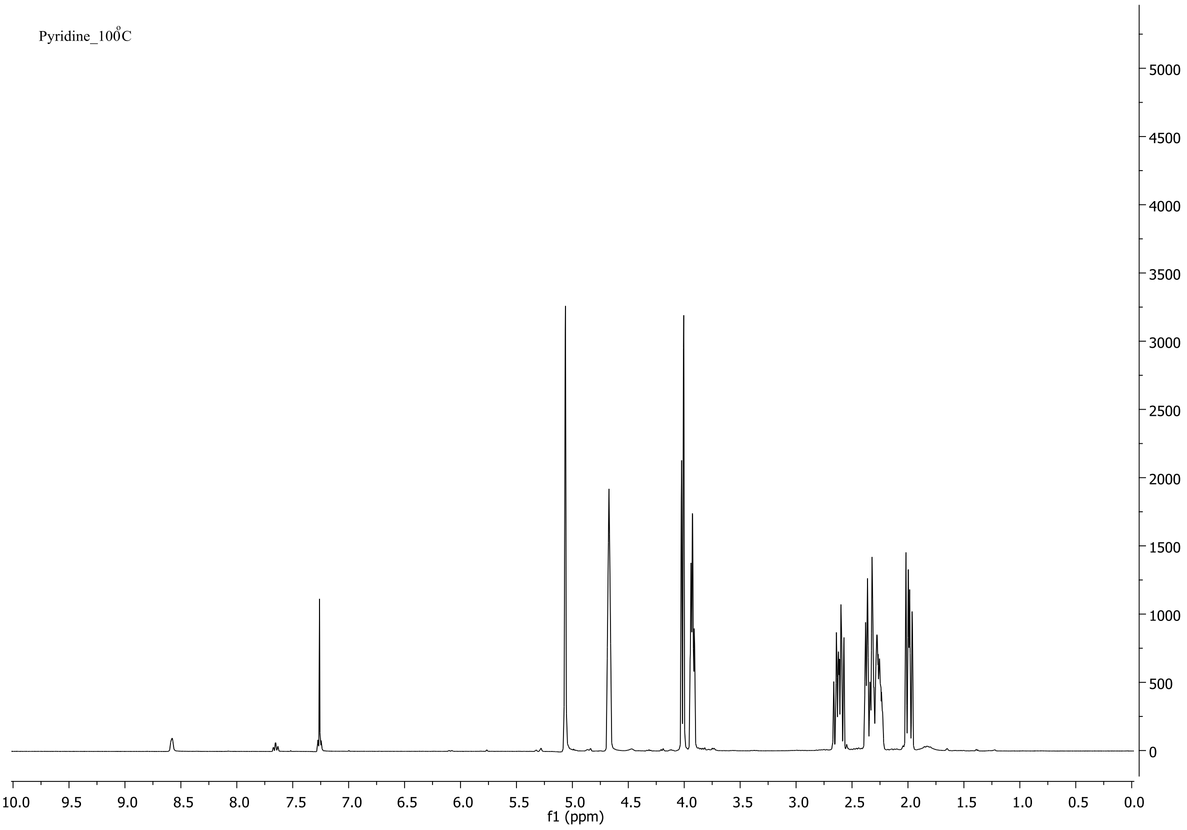

K_2CO_3 25 °C

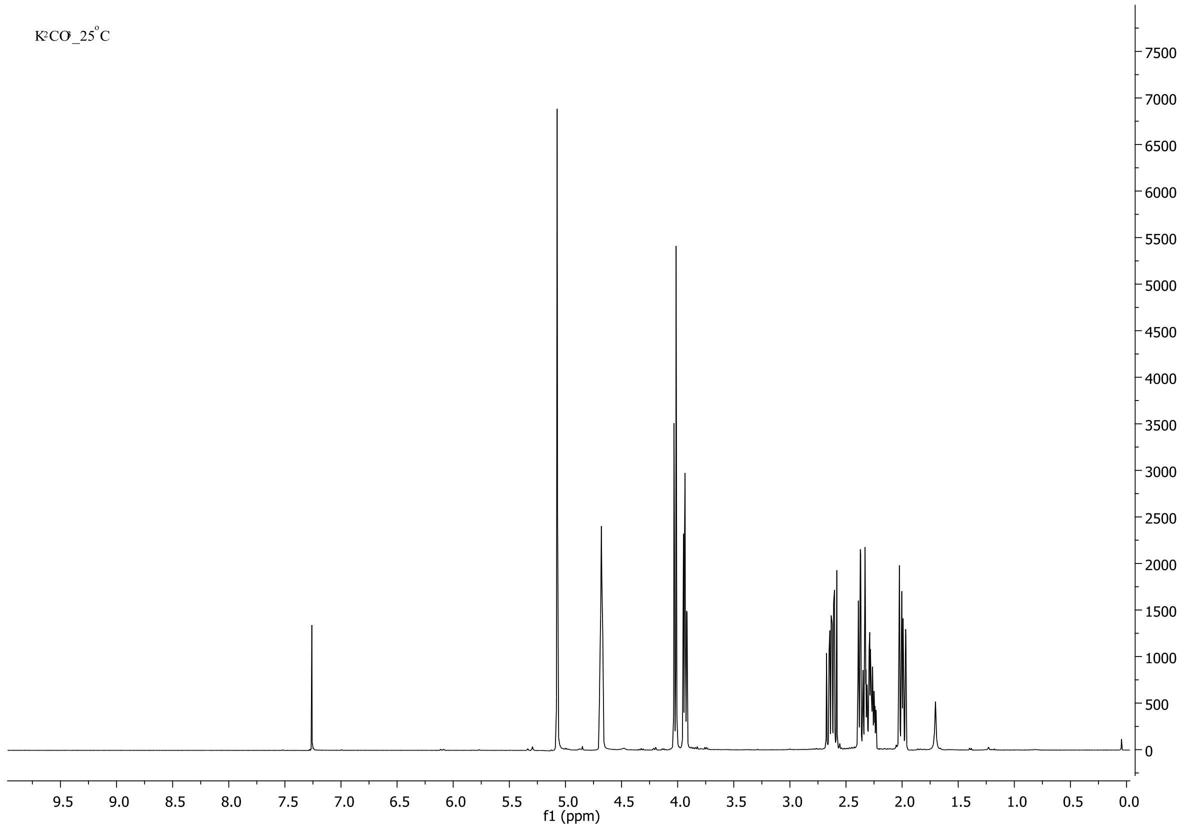

K_2CO_3 50 °C

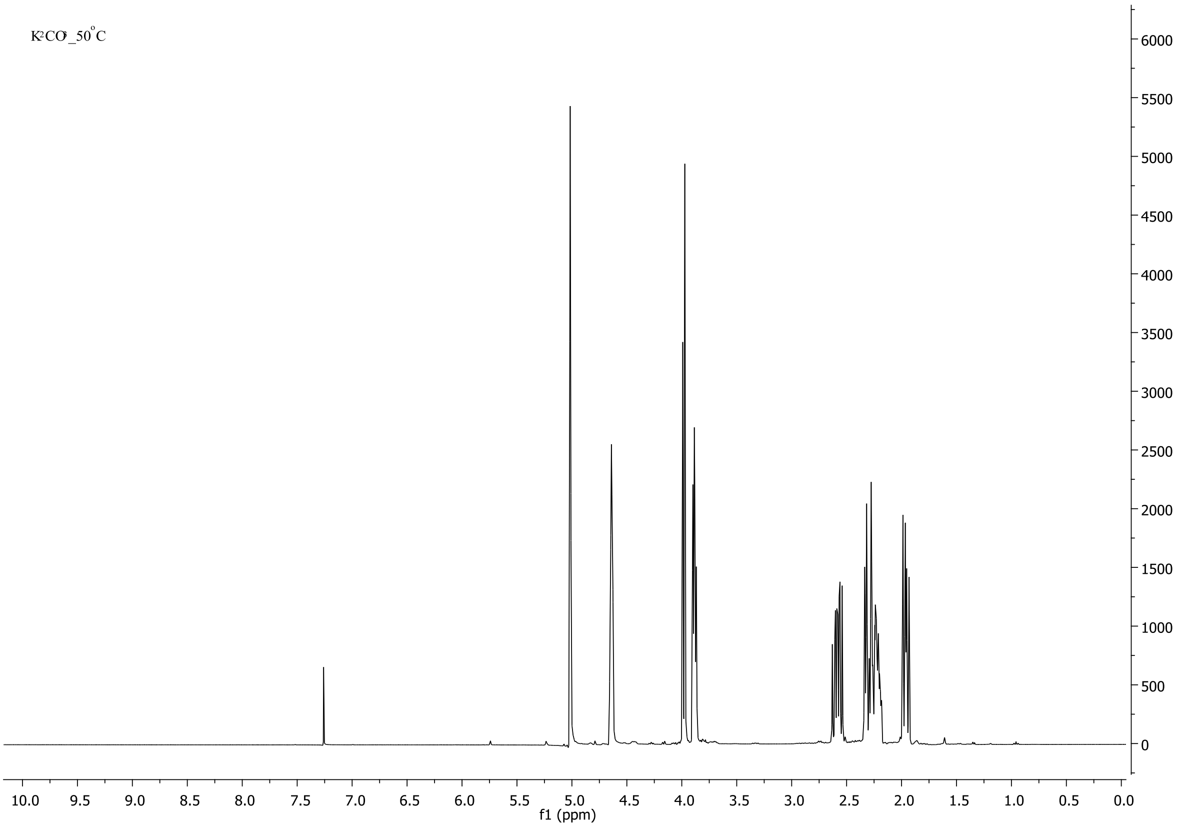

K_2CO_3 100 °C

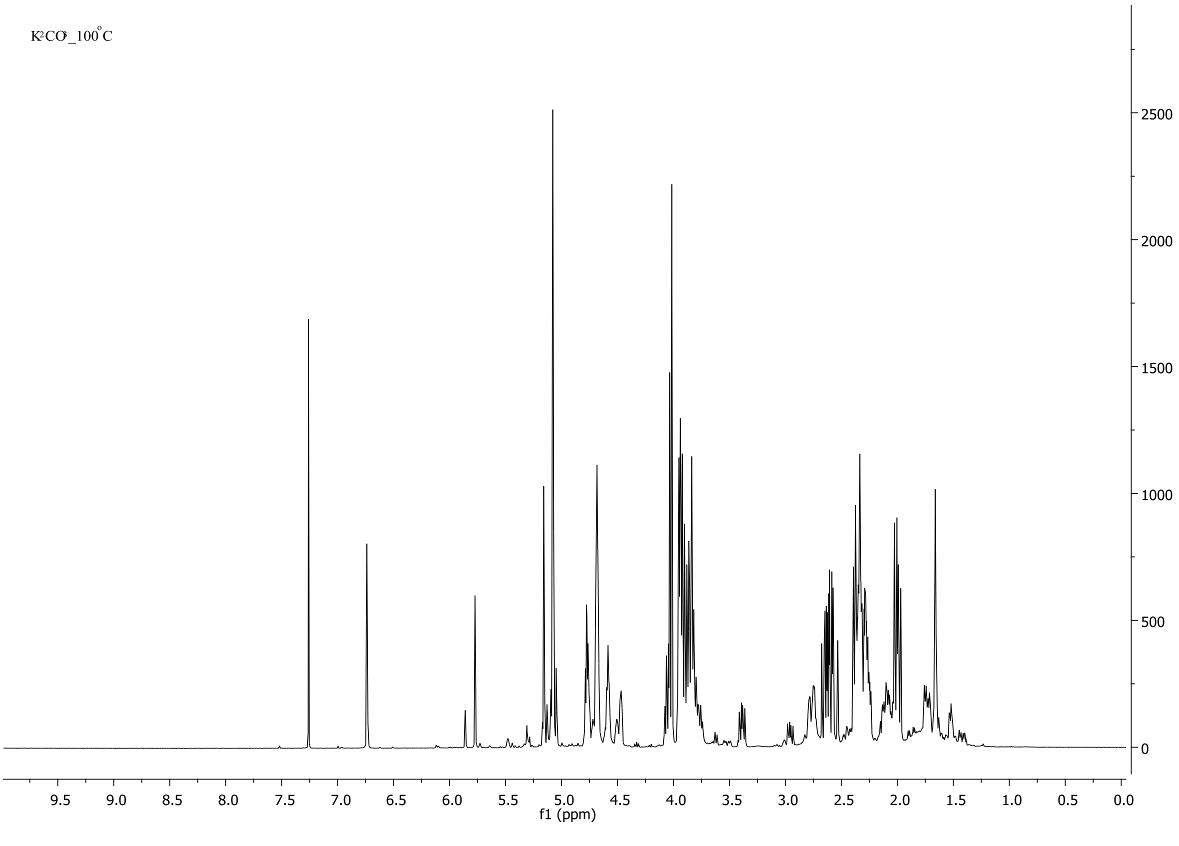

DIPEA 25 °C

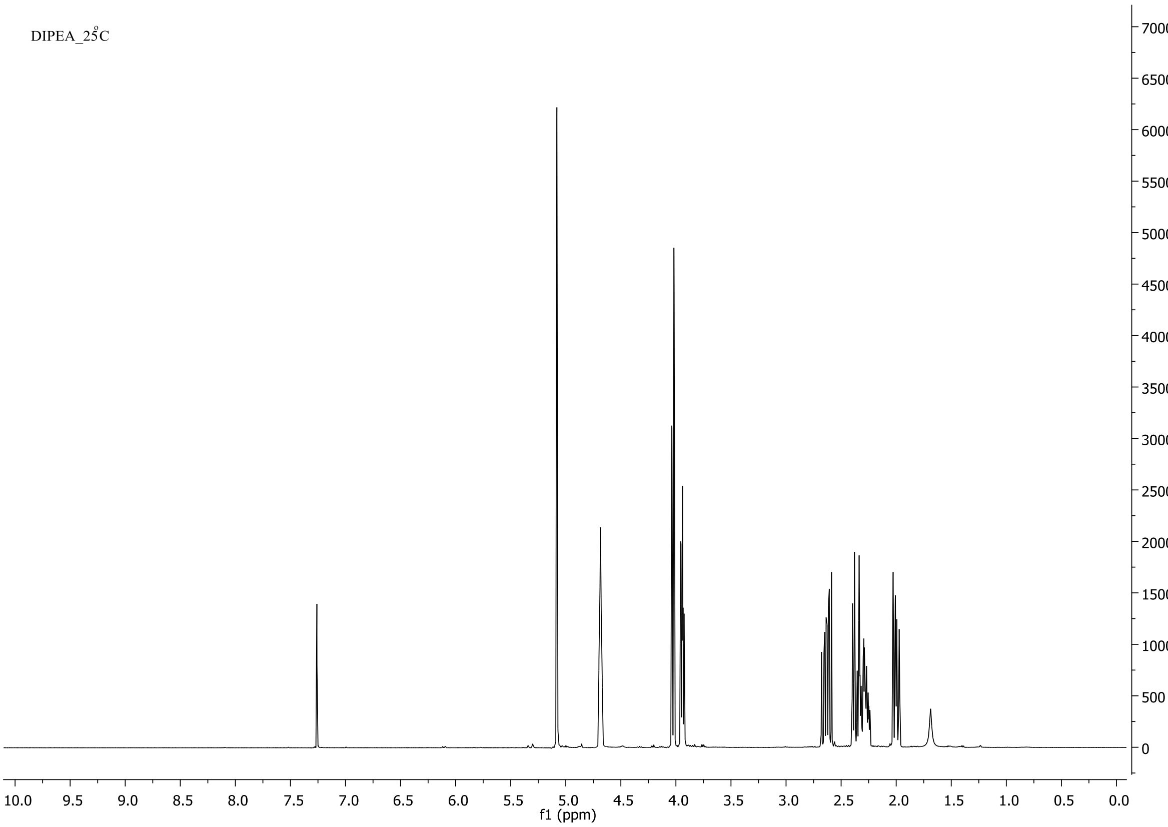

DIPEA 50 °C

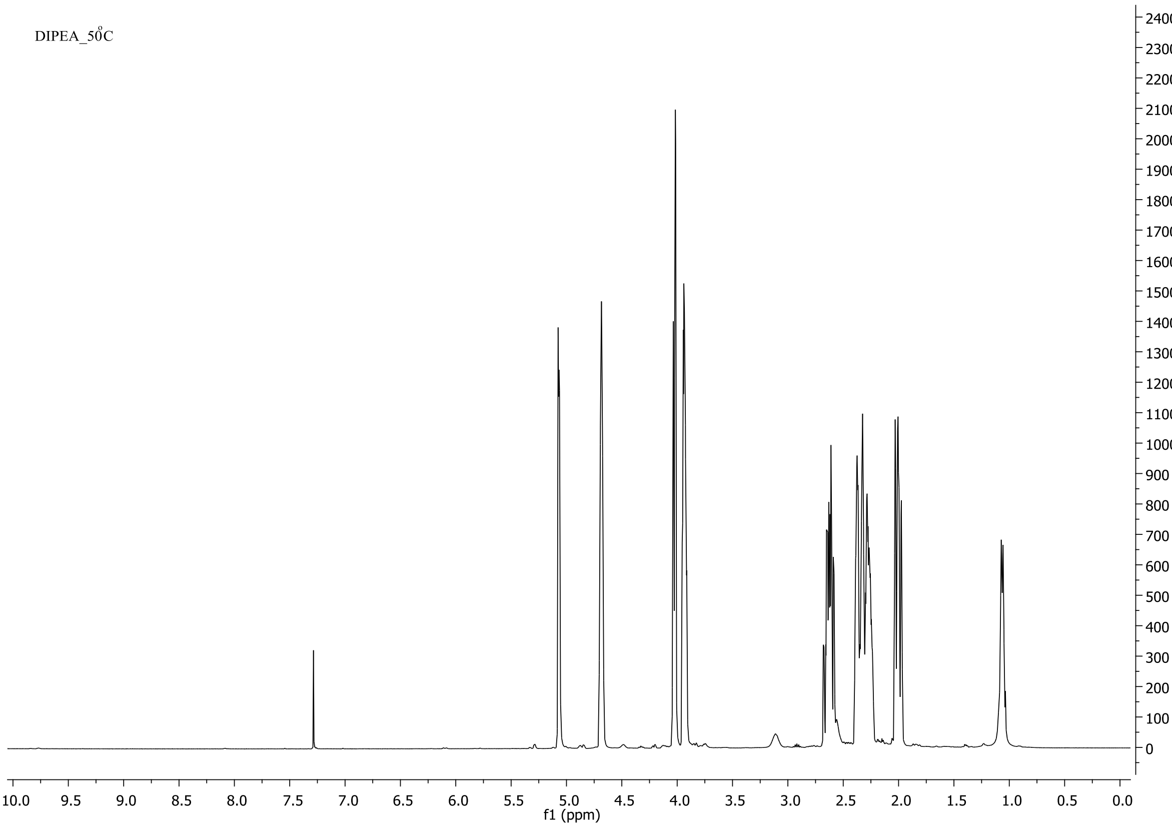

DIPEA 100 °C

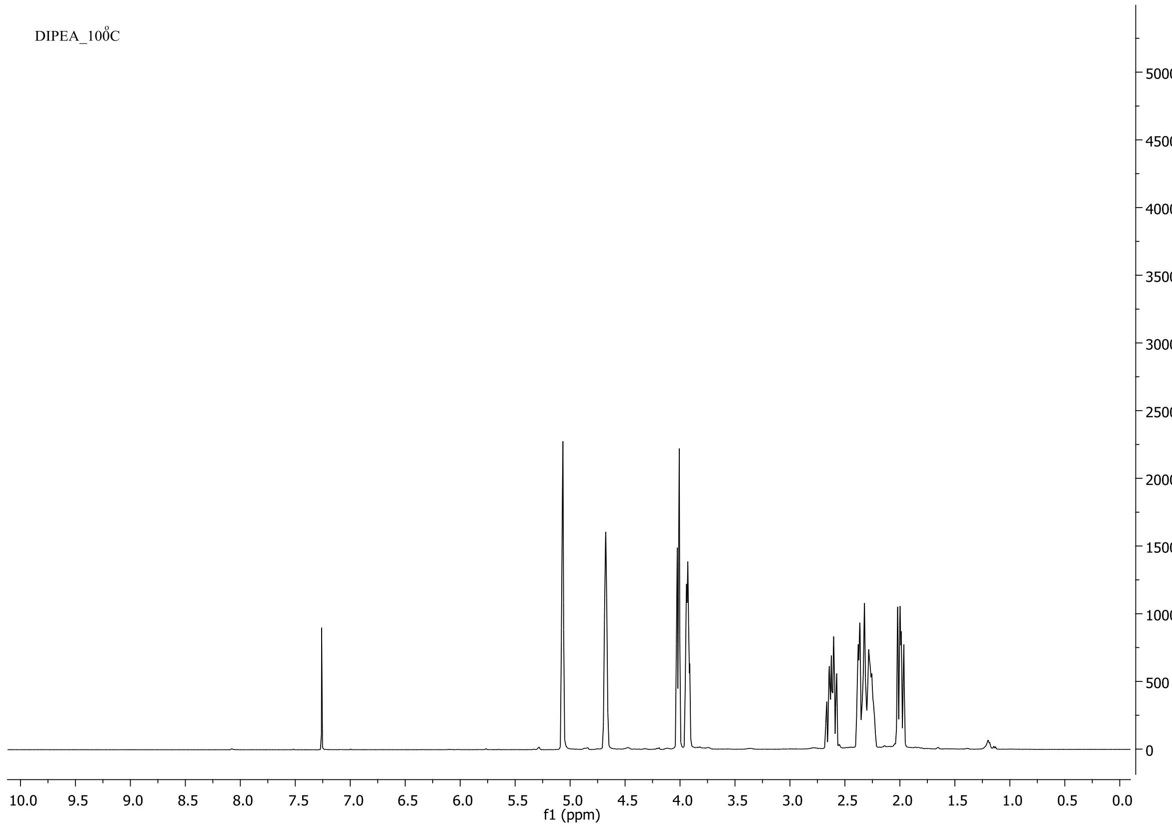

Cs_2CO_3 25 °C

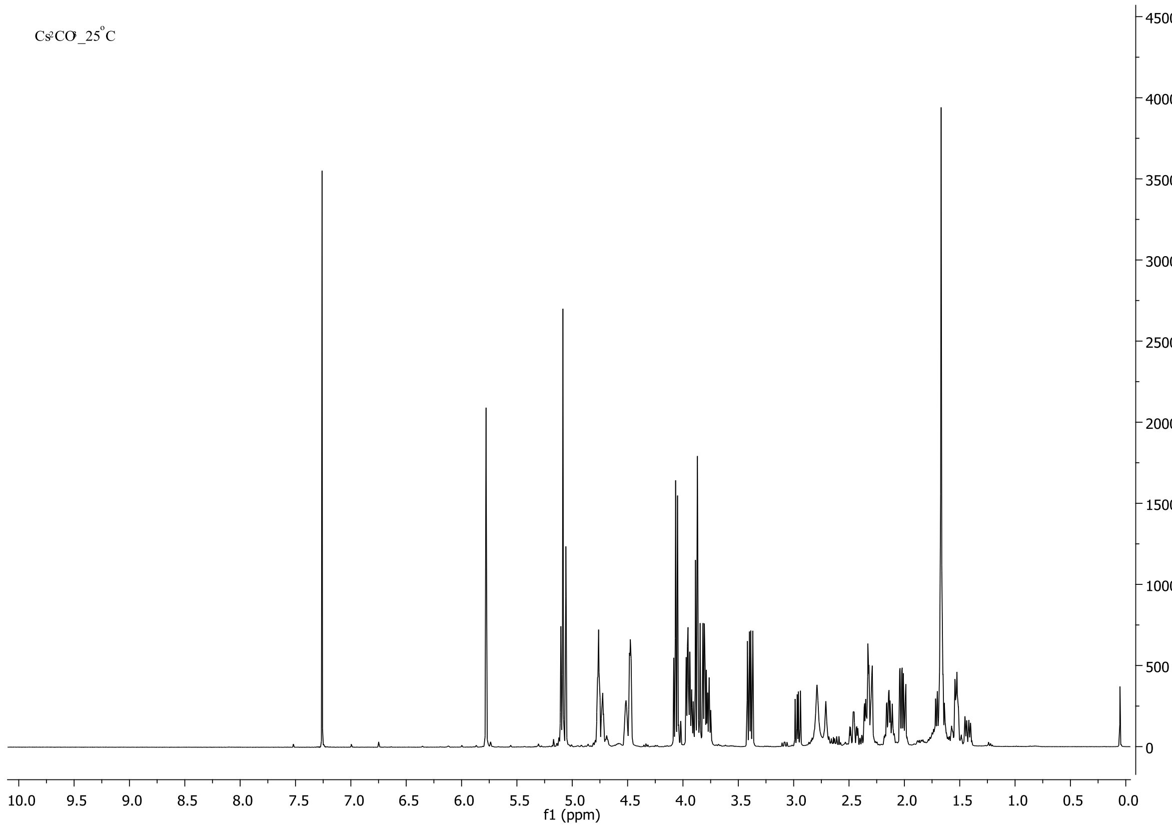

Cs_2CO_3 50 °C

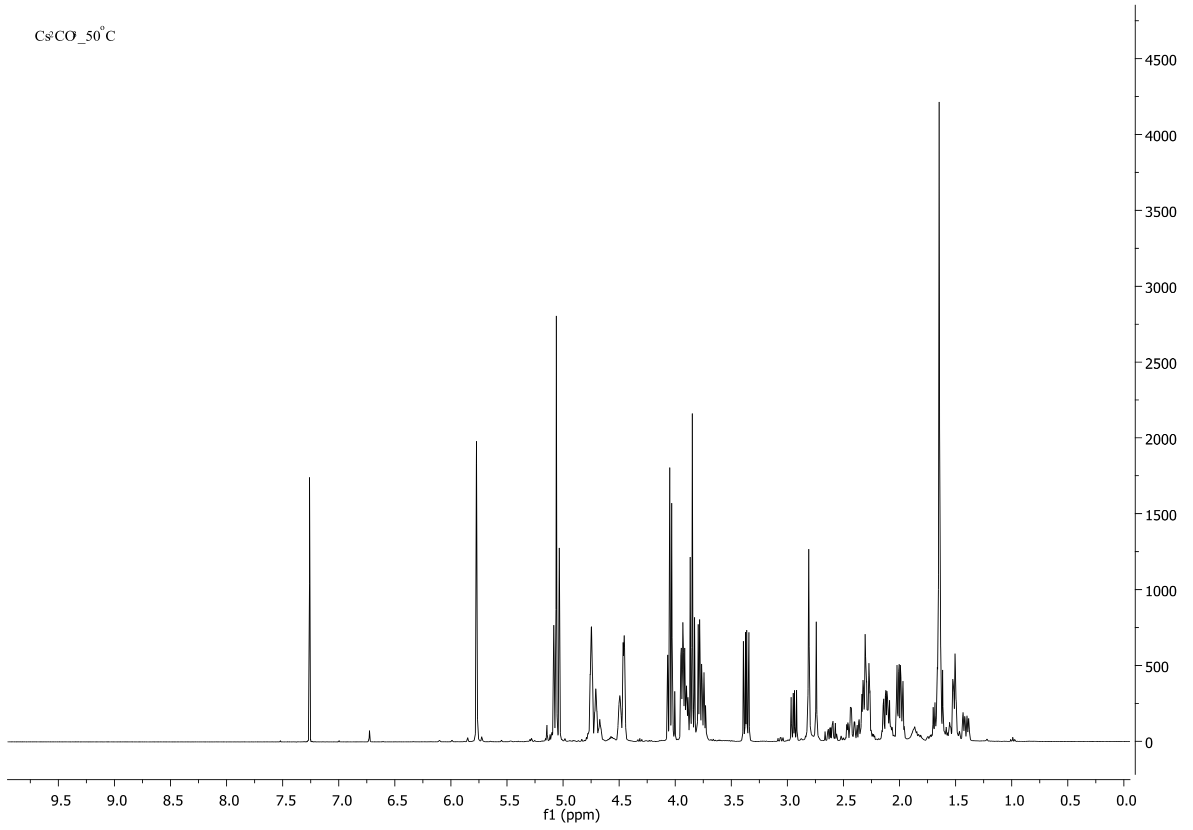

Cs_2CO_3 100 °C

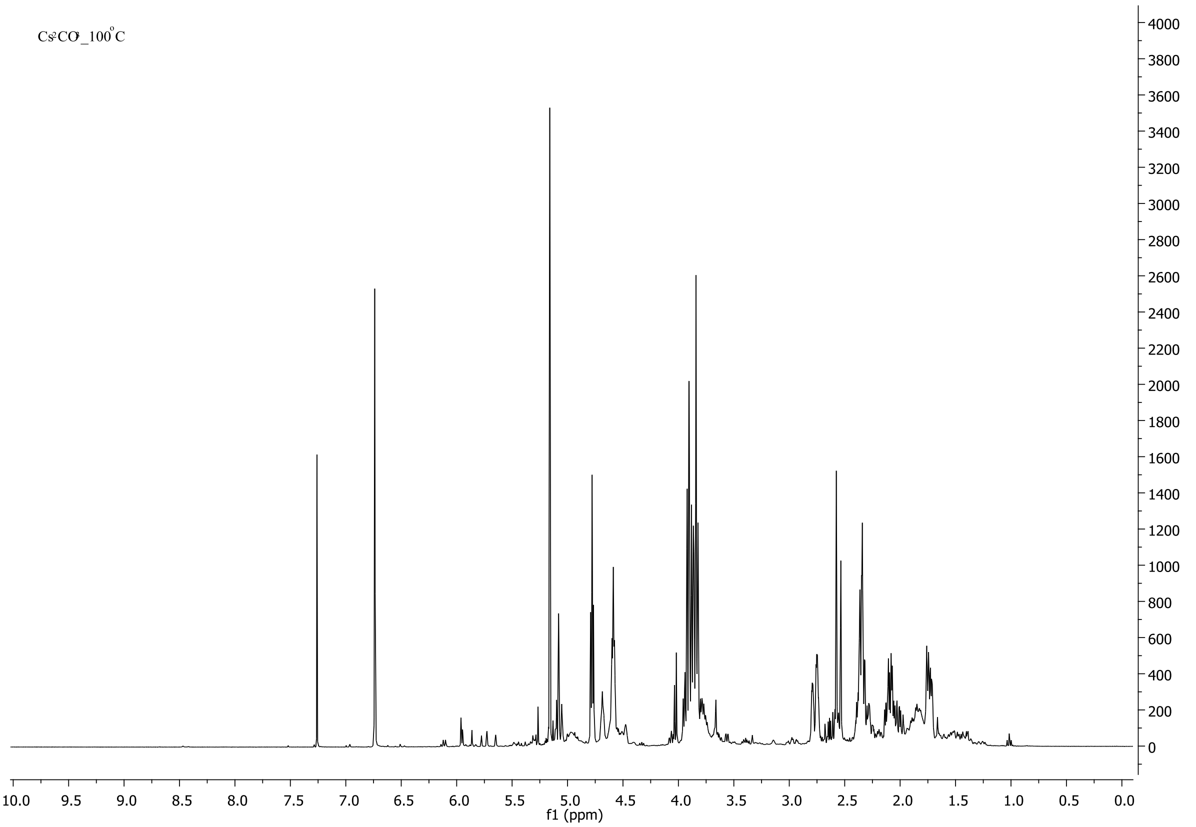

Et_3N 25 °C

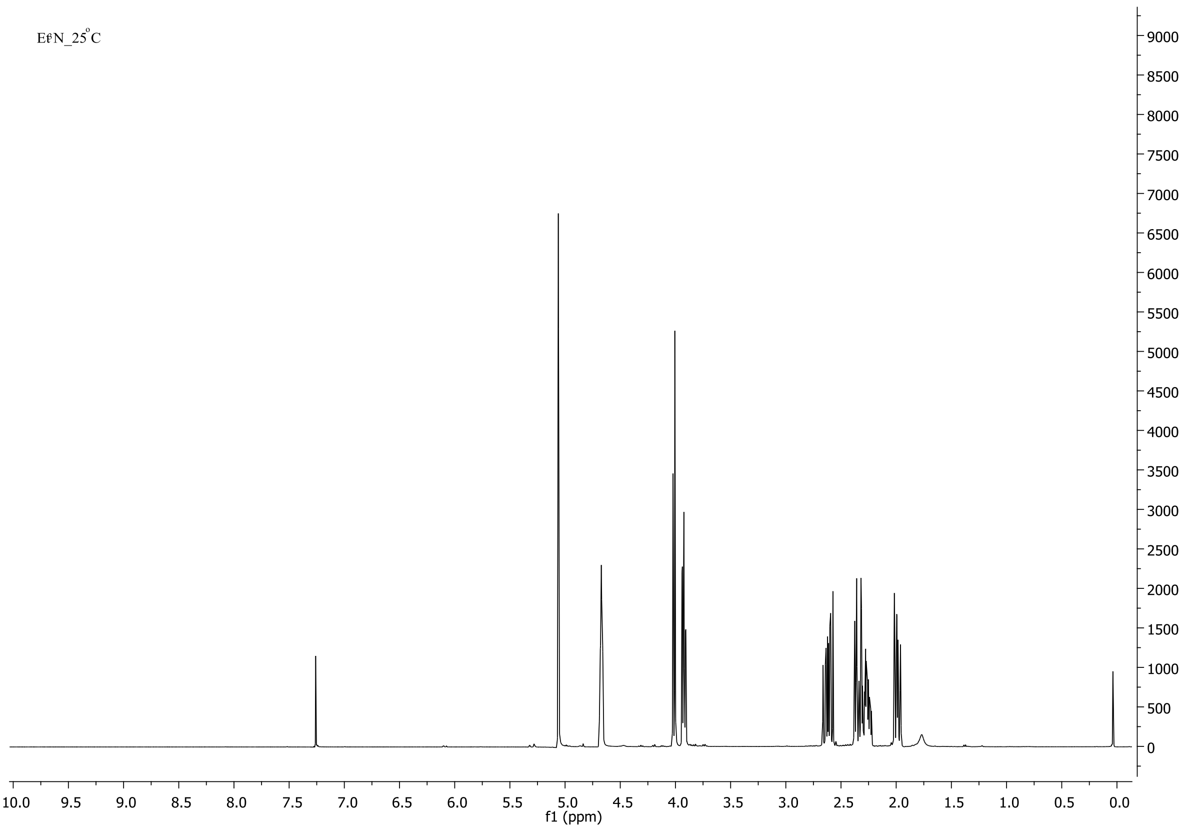

Et_3N 50 °C

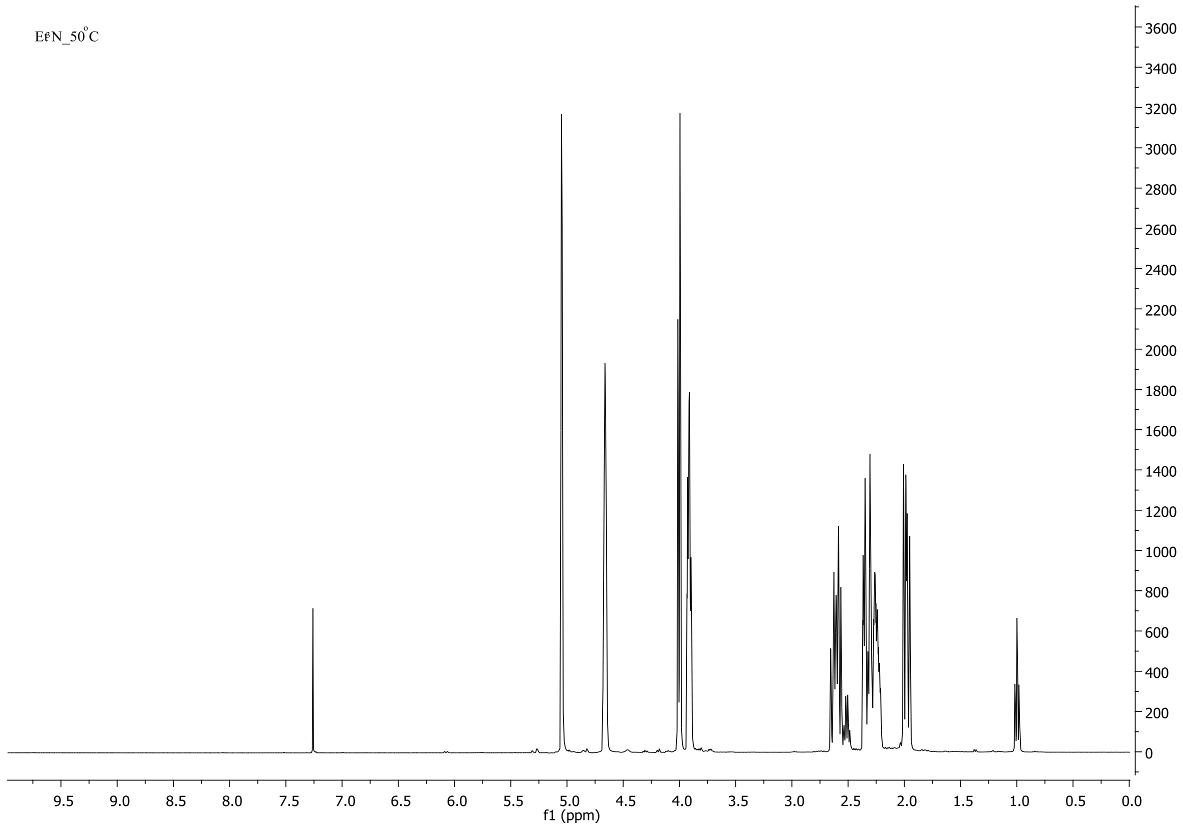

Et_3N 100 °C

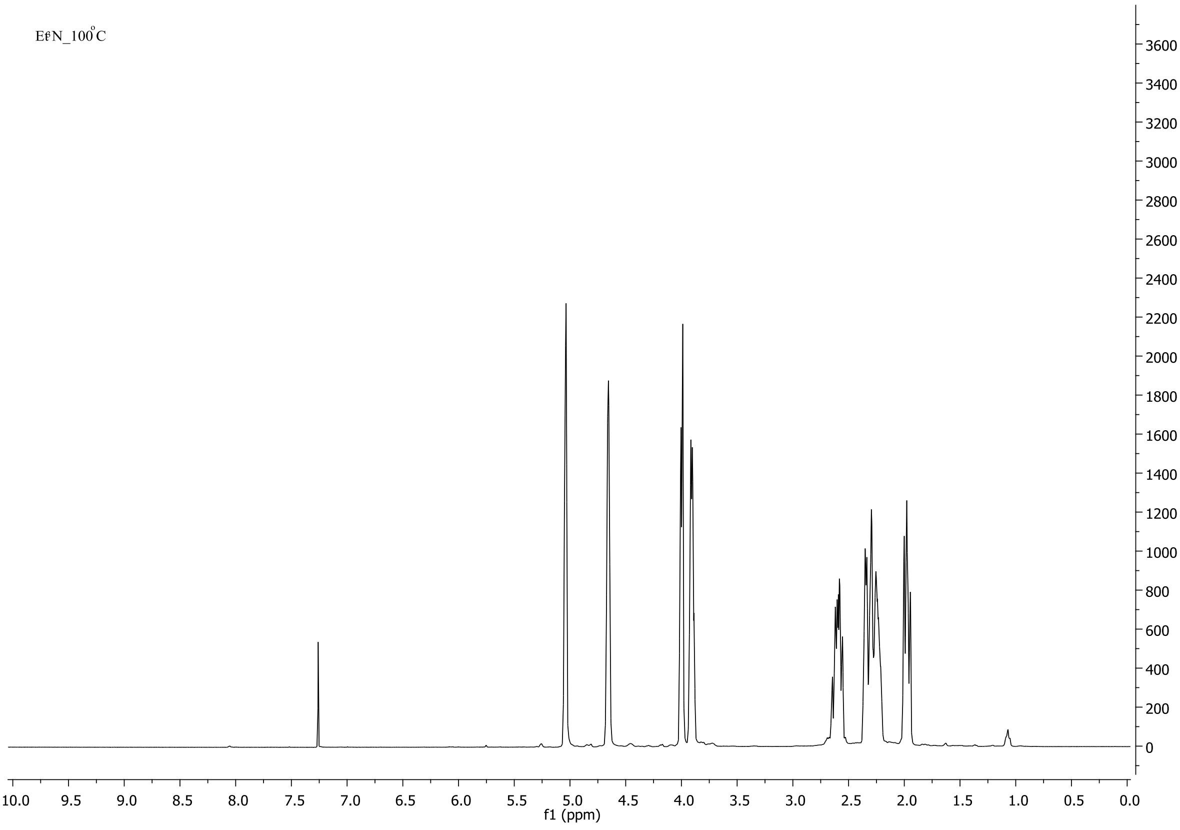

K_3PO_4 25 °C

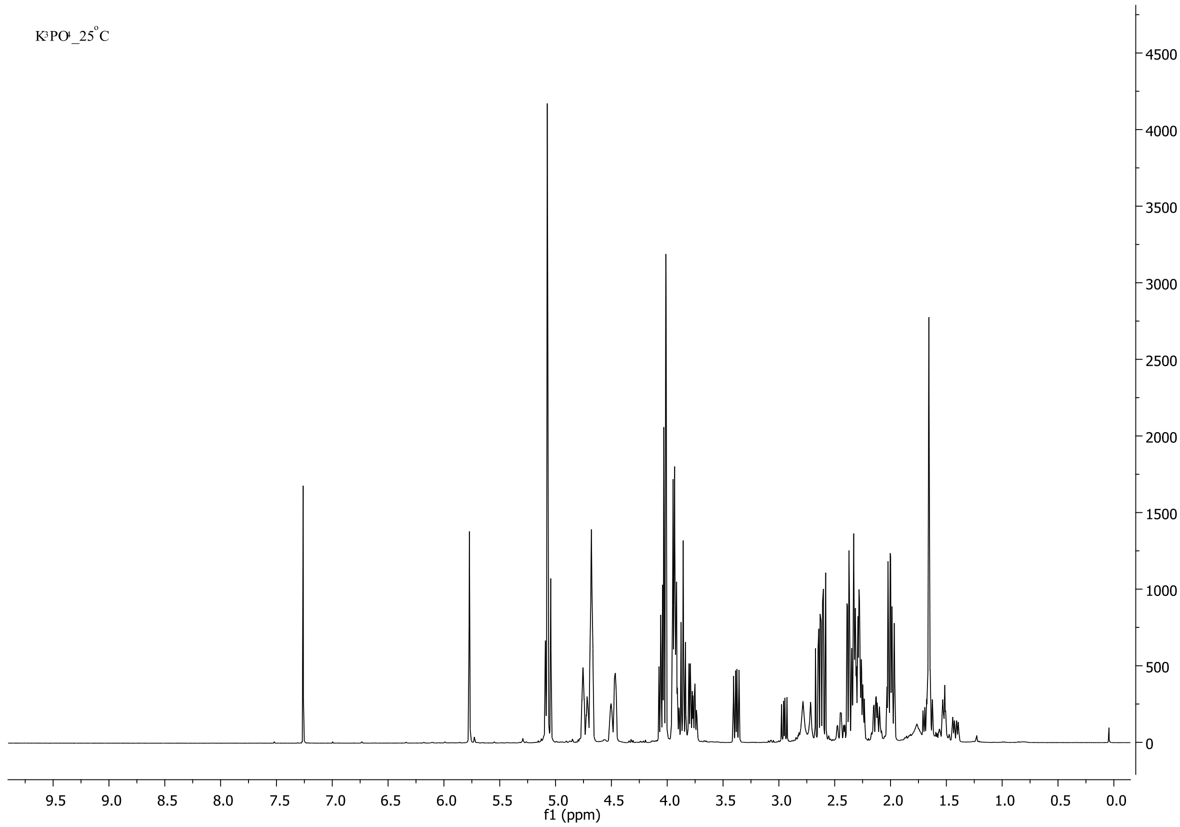

K_3PO_4 50 °C

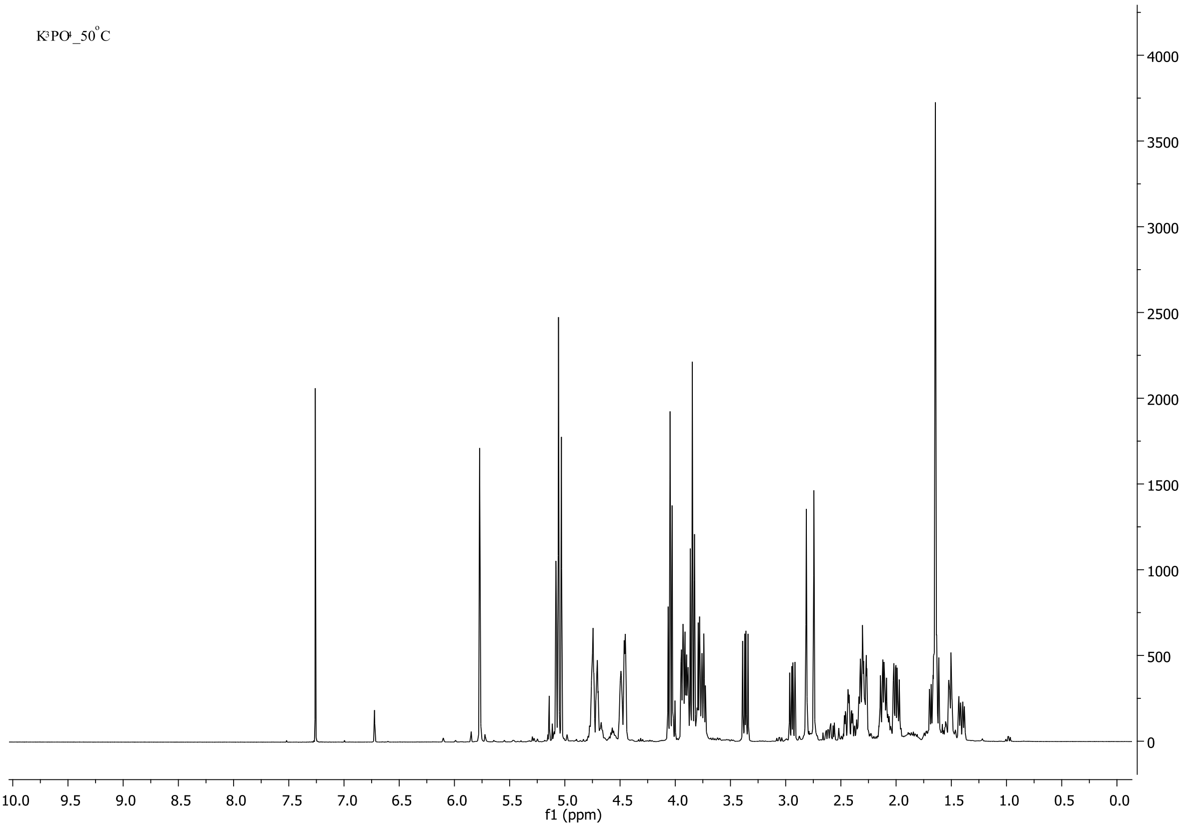

K_3PO_4 100 °C

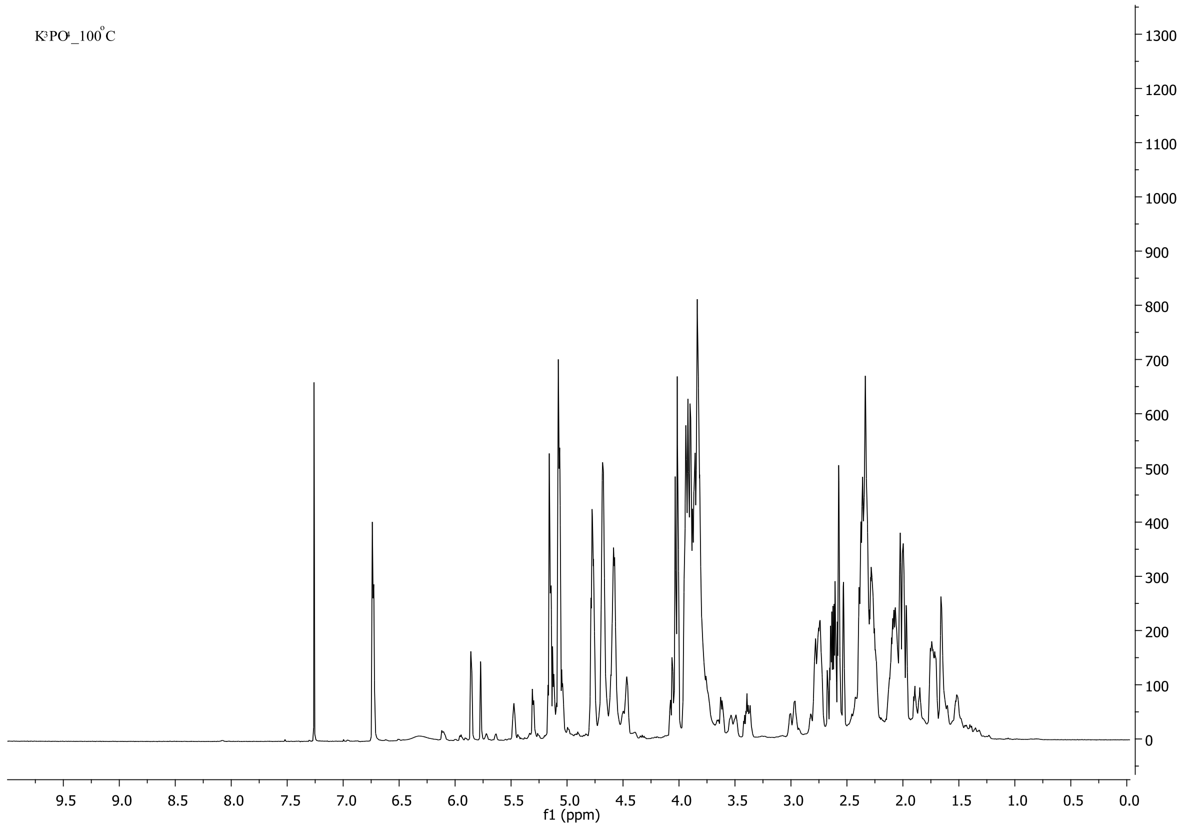

DBU 25 °C

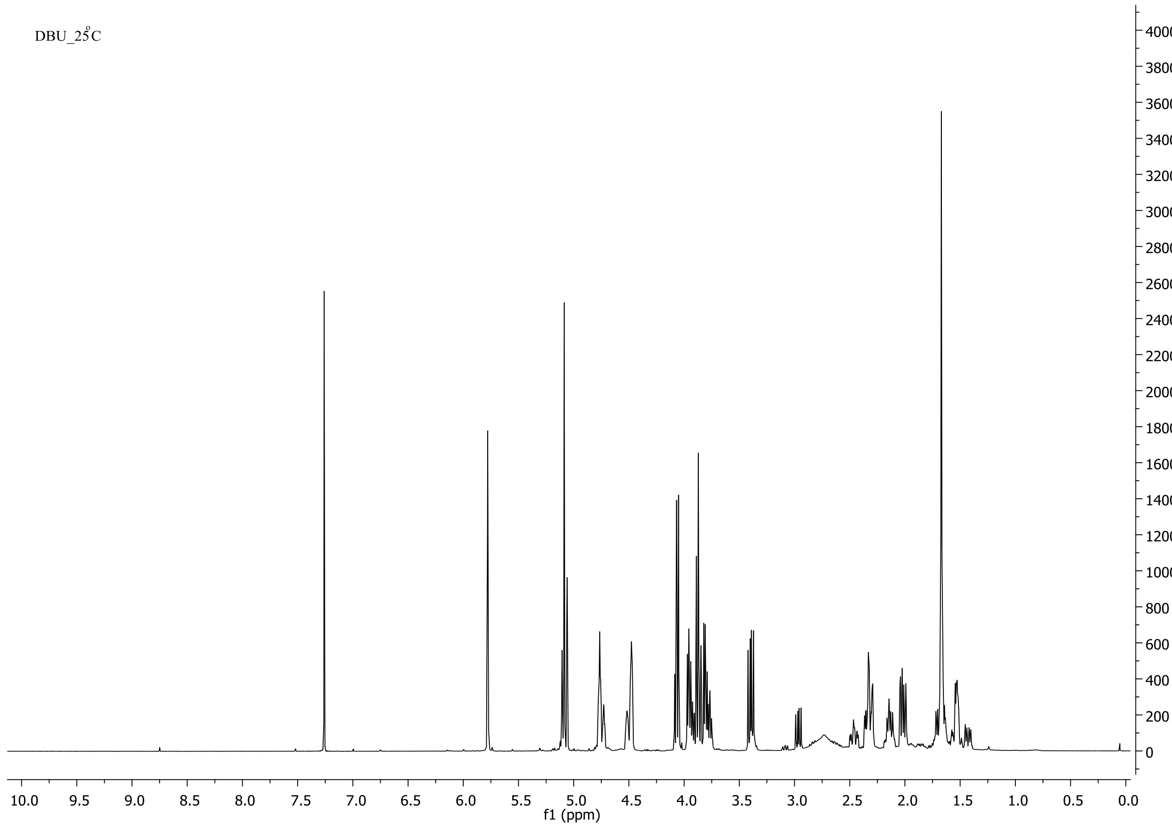

DBU 50 °C

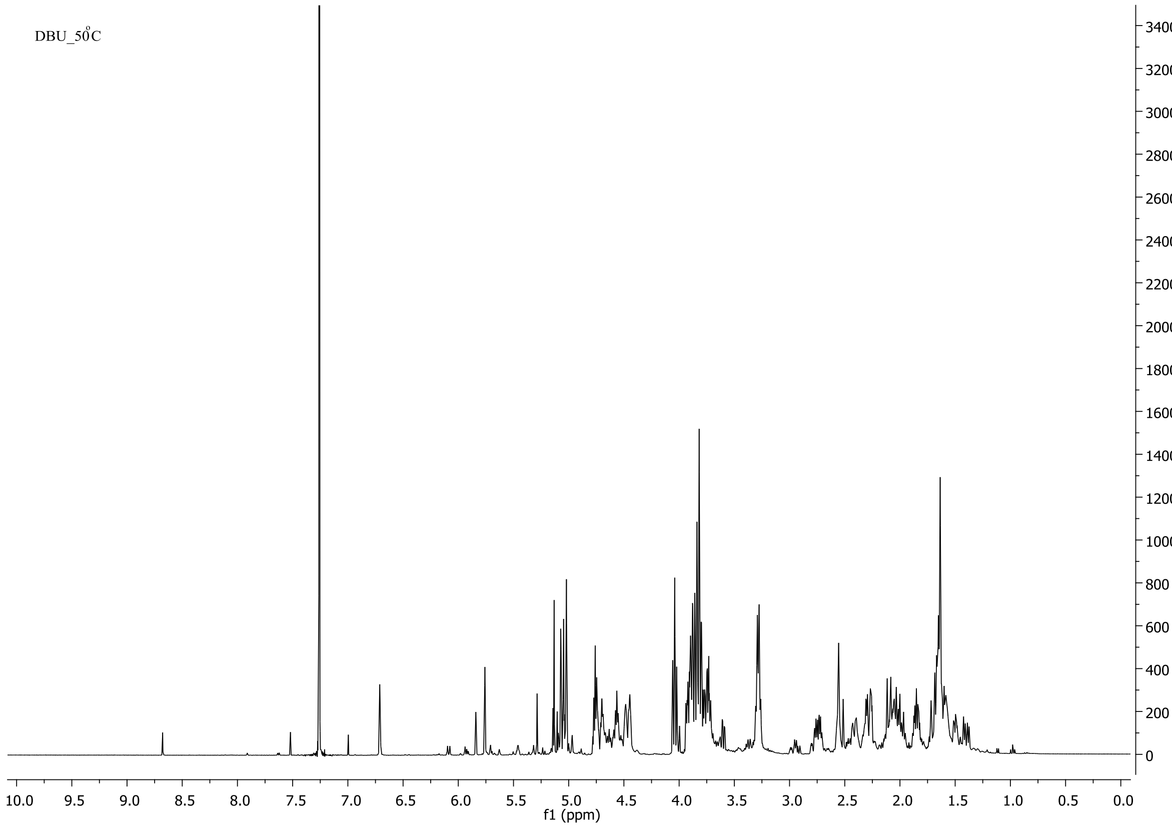

DBU 100 °C

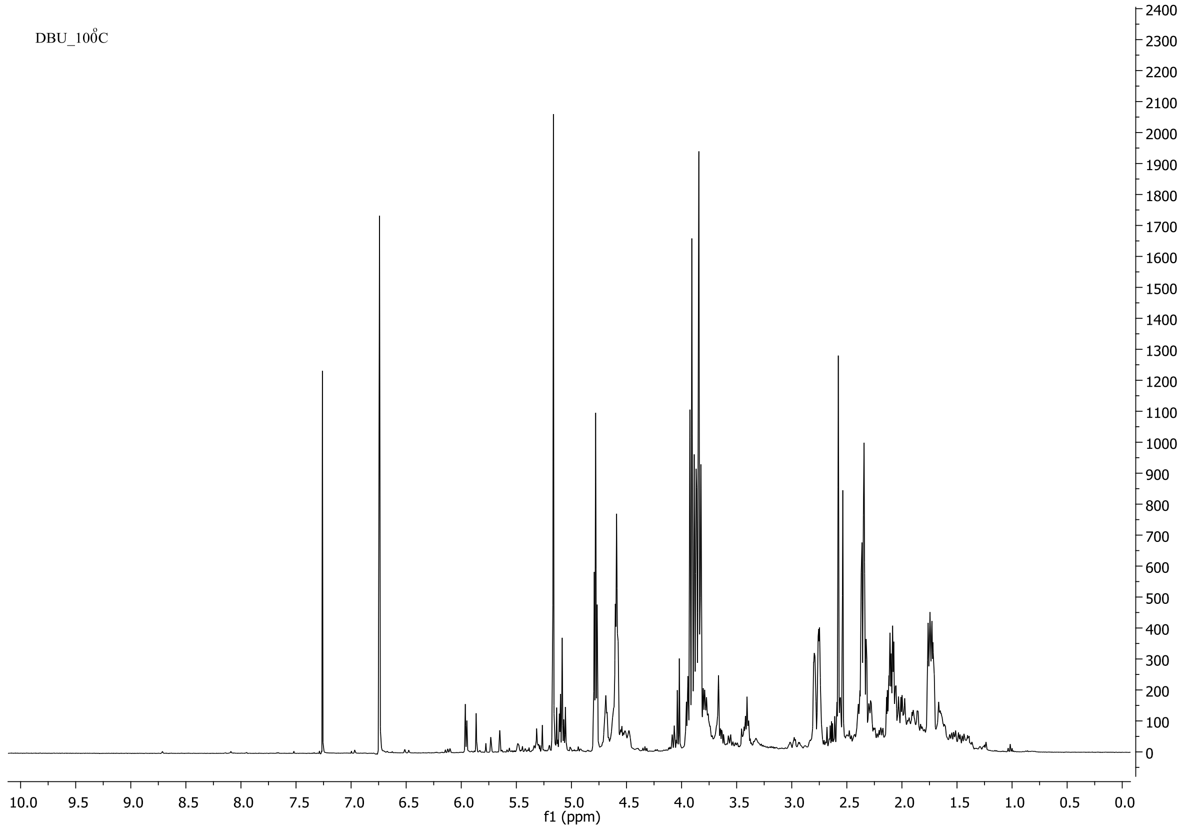

KOH 25 °C

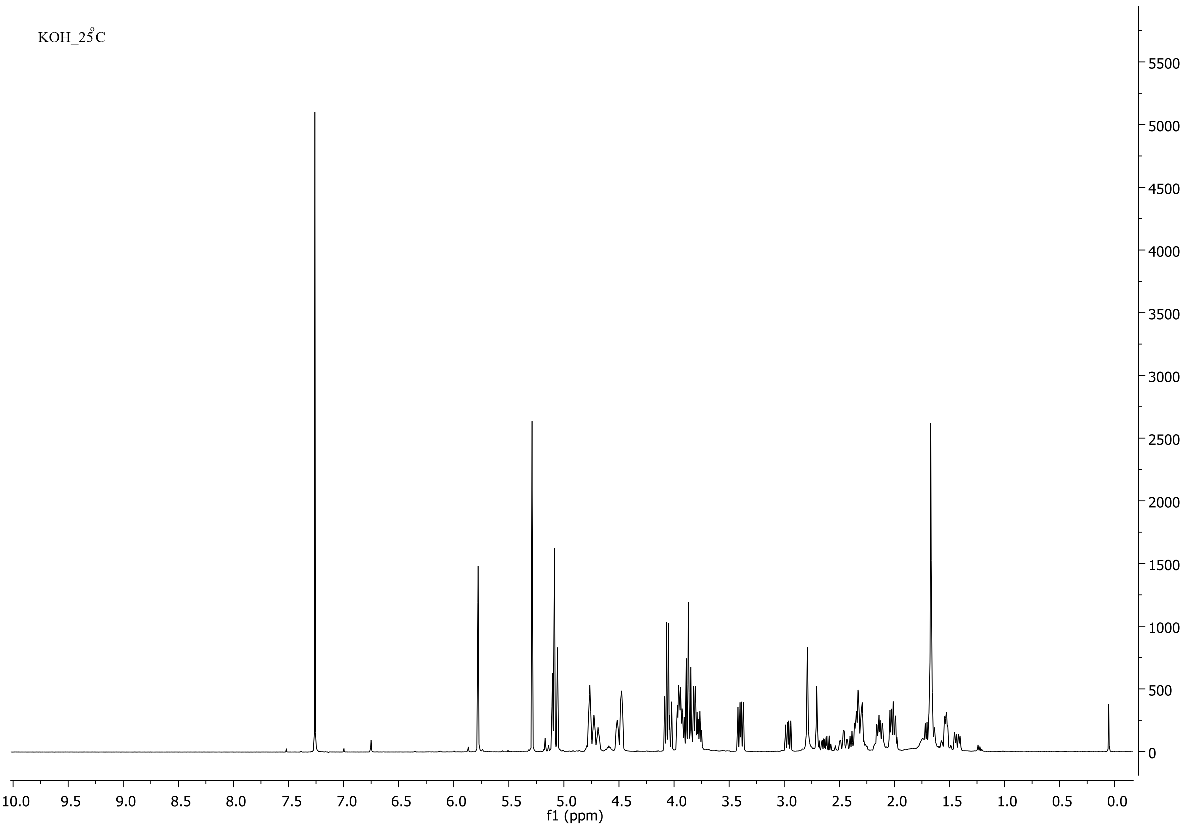

KOH 50 °C

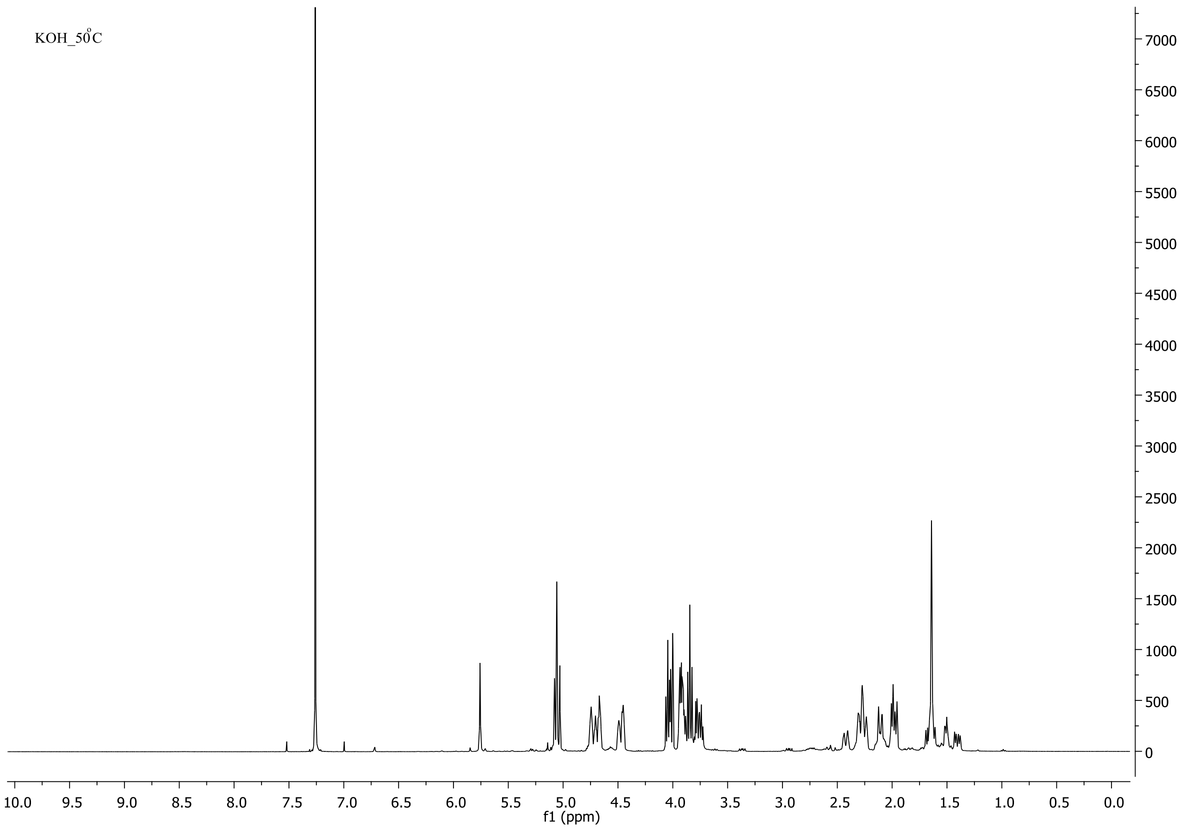

KOH 100 °C

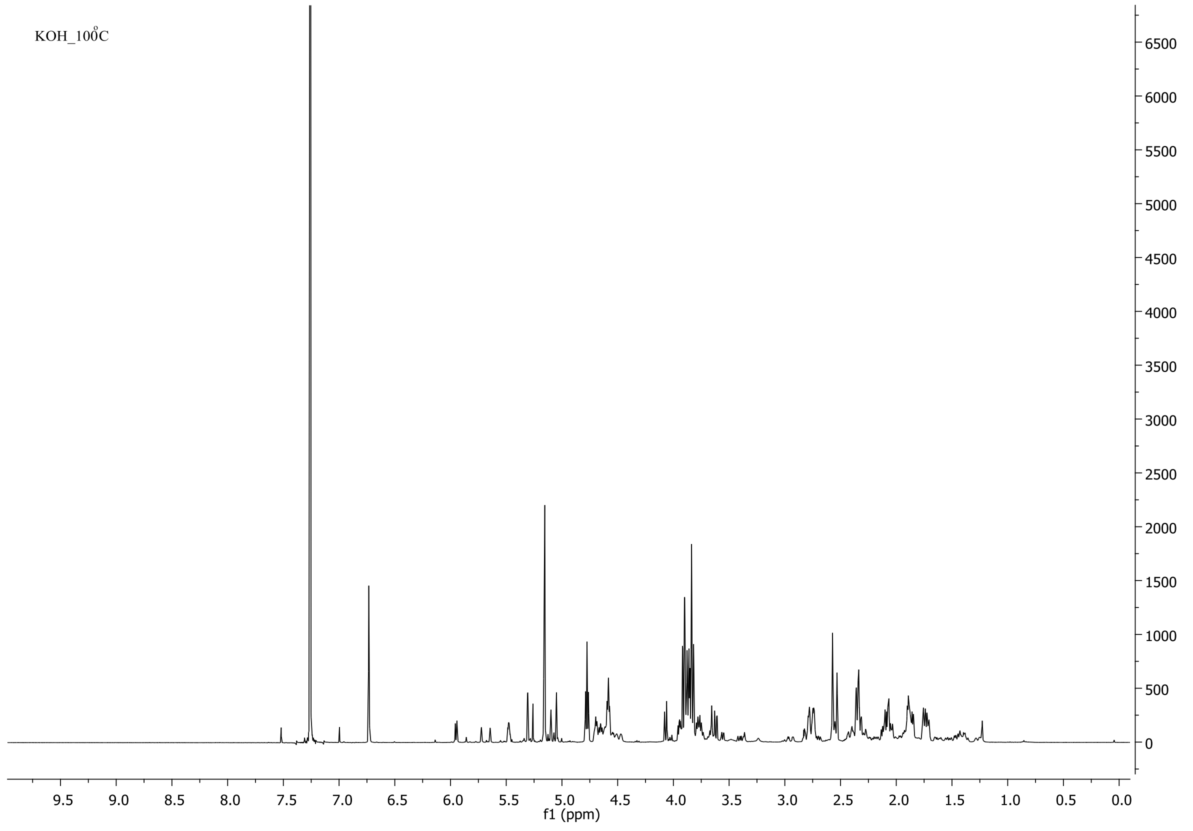

t-BuOK 25 °C

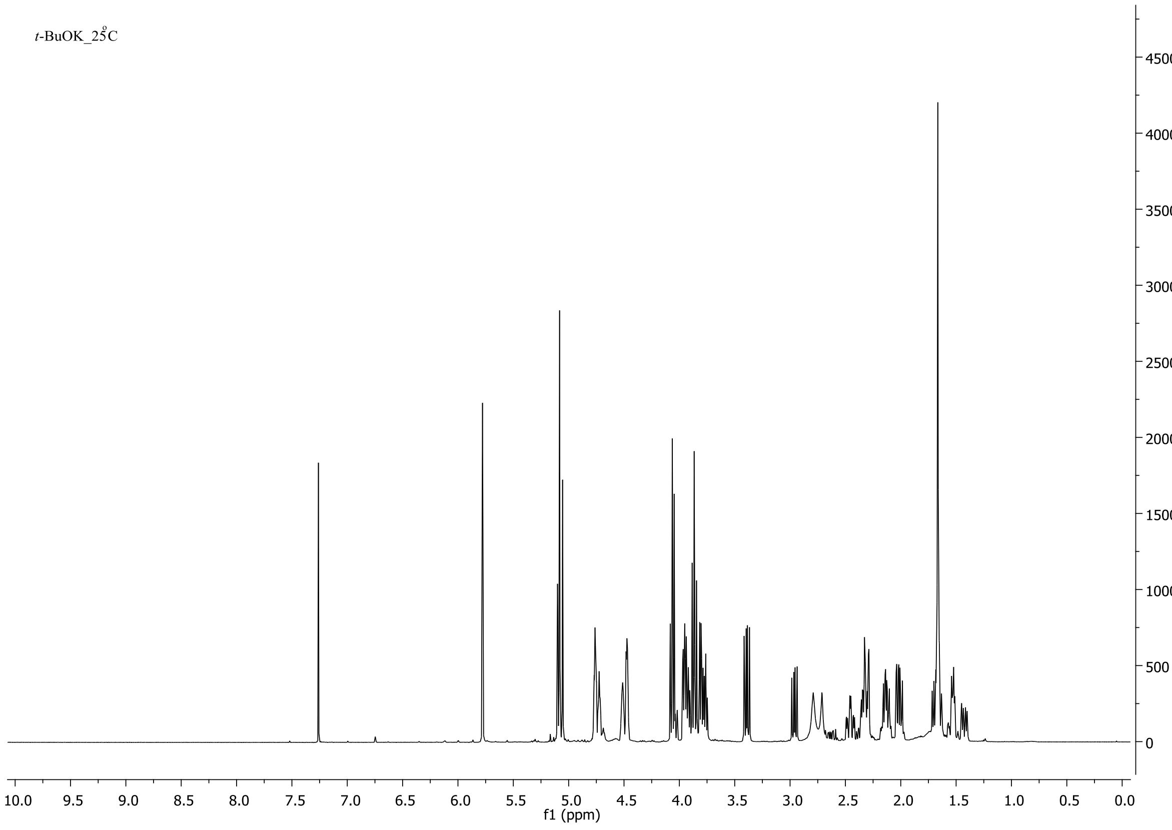

t-BuOK 50 °C

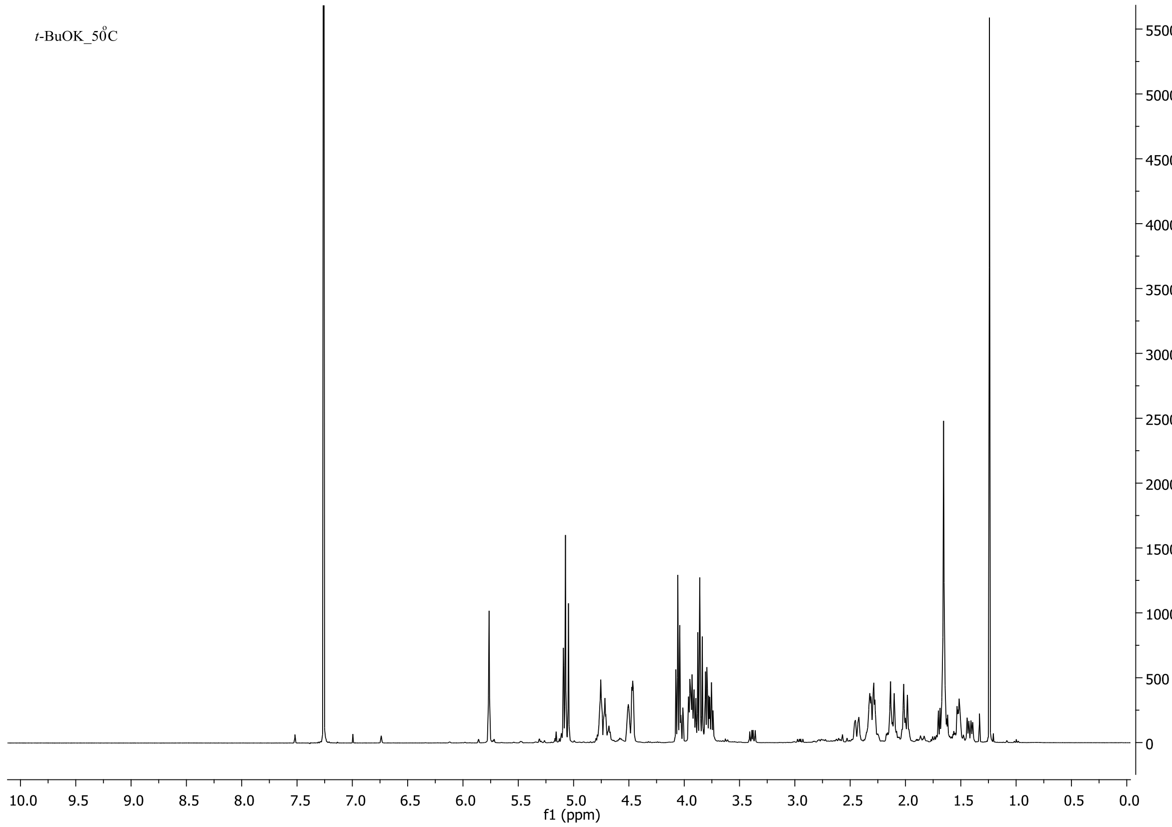

***t*-BuOK 100 °C**

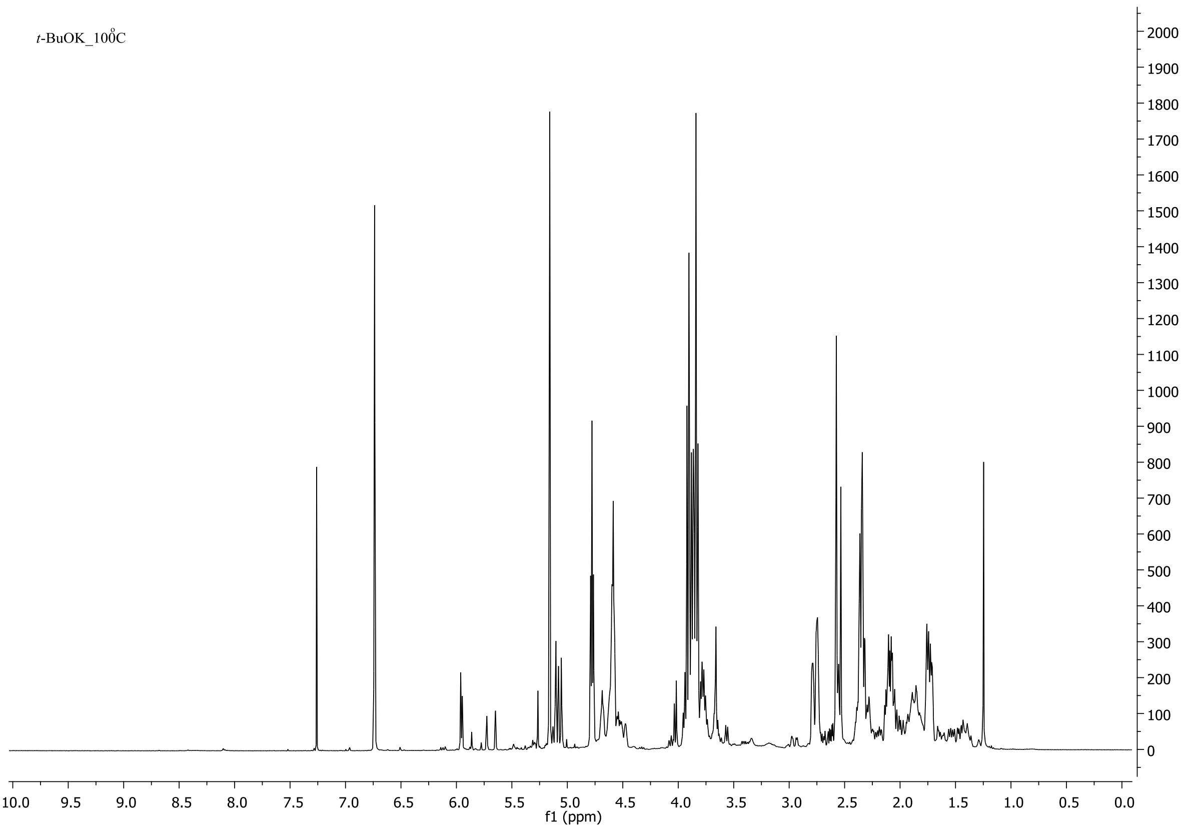

NaH 25 °C

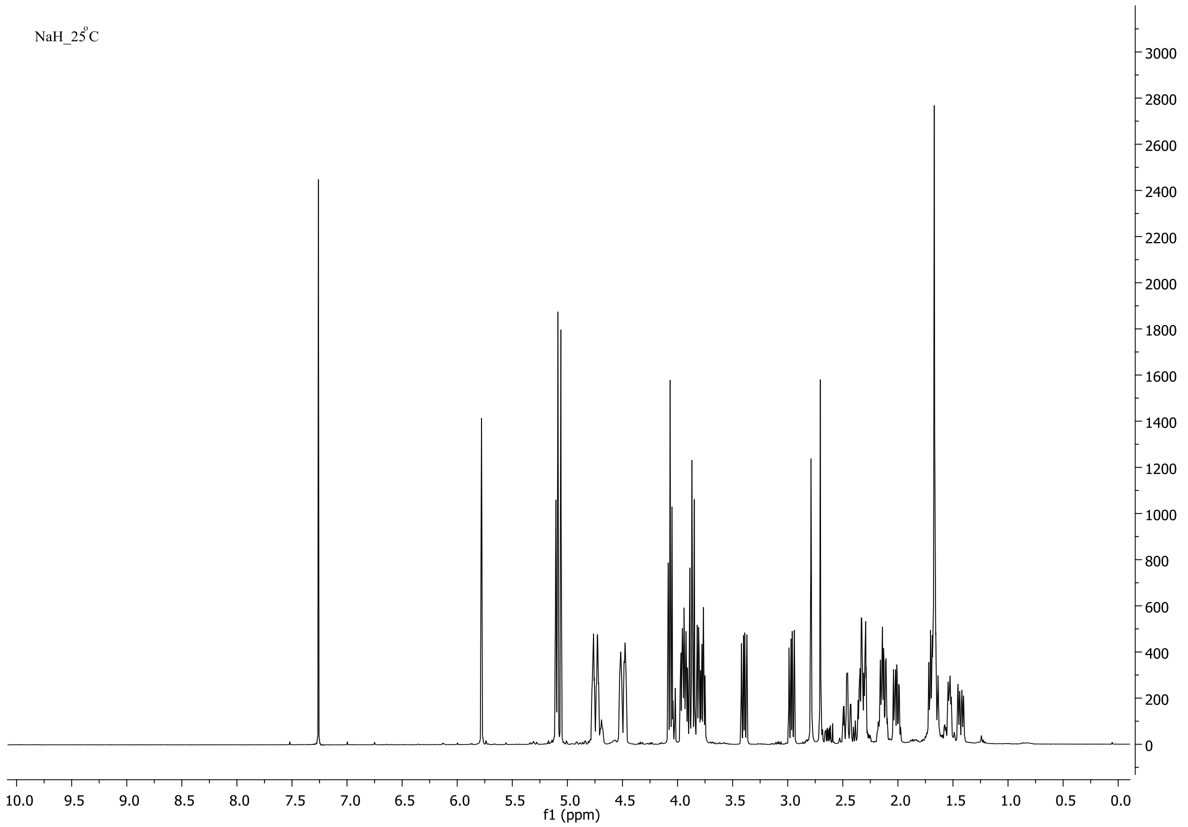

NaH 50 °C

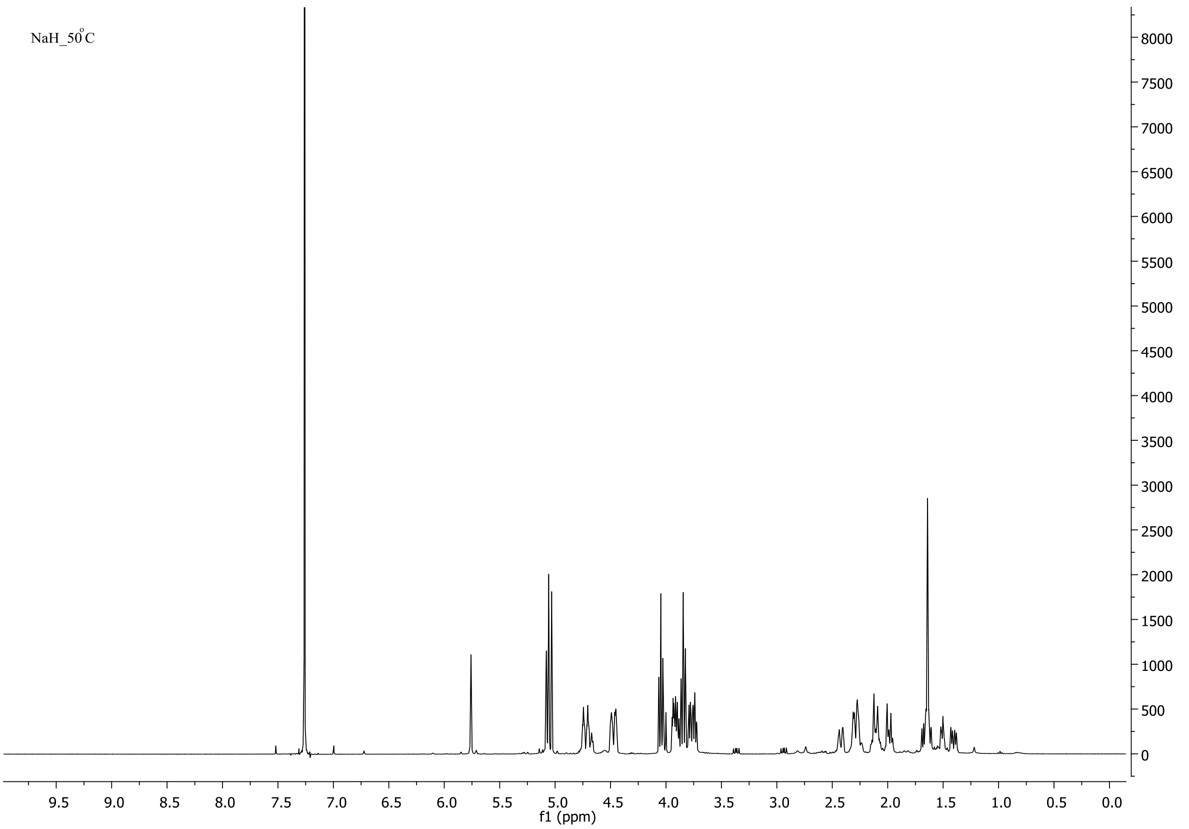

NaH 100 °C

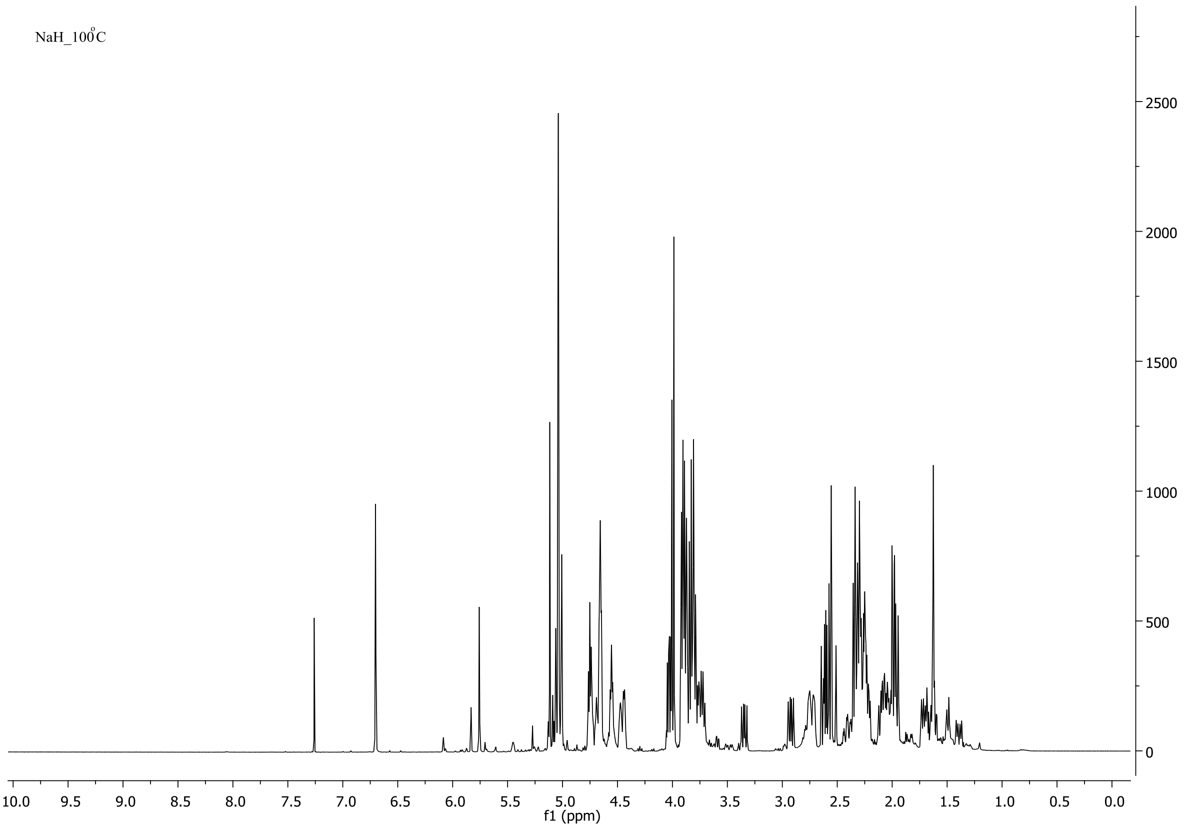

Supplement: File 1 — Experimental procedures, analytical data, copies of NMR spectra, and single X-ray crystal diffraction data of 4b. [file Beilstein_J_Org_Chem-12-2005-s001.pdf]
